# Linking solver characteristics, solving processes and solution attributes: A data explainer for an open innovation generated robotic design dataset

**DOI:** 10.1016/j.dib.2023.109547

**Published:** 2023-09-06

**Authors:** Zoe Szajnfarber, Anthony Hennig, Suparna Mukherjee, Steven Rader, Jason Crusan

**Affiliations:** a The George Washington University, 800 22^nd^ St NW, Washington DC 20052, USA; b NASA, Johnson Space Center, Mail Code AA212 2101 E NASA Pkwy, Houston, TX 77058, USA

**Keywords:** Design data, Solver attributes, Crowdsourcing, Novelty, Feasibility, Robotics

## Abstract

Between 2017 and 2020, a team of researchers from the George Washington University collaborated with NASA and Freelancer.com to design and launch the “Astrobee Challenge Series,” a large-scale field experiment that aimed to generate data to characterize the relationship among how a technical problem is formulated and who is able and willing to solve, and the quality of solutions they generate. The core experimental manipulation was of the architecture of the problem posed; the typical open innovation process was instrumented to collect unusually rich data but otherwise untouched. In all, 17 individual contests were run over a period of 12 months. Over the course of the challenge series, we tracked a population of 16,249 potential solvers, of which 6,219 initiated solving, and a subset of 147 unique solvers submitted 263 judgeable solutions. The resultant dataset is unique because it captures demographic and expertise data on the full population of potential solvers and links their activity to their solving processes and solution outcomes. Moreover, in addition to winning designs (the typical basis of analysis), it captures design outcomes for all submitted design artifacts allowing analysis of the variety of solutions to the same problem. This data explainer documents the research design and implementation process and provides a detailed explanation of each data record, carefully characterizing potential limitations associated with research design choices. This data should be useful for researchers interested in studying the design and innovation process, particularly those focused on novelty, variety, feasibility of solutions or expertise, diversity and capability of solvers.

Specifications Table

**Table:** 

Subject	Engineering

Specific subject area	Engineering design, space robotics, open innovation methods
Data format	Raw, Analyzed
Type of data	Table, Figure
Data collection	Data were collected by instrumenting the Astrobee Challenge Series. The series included 17 prize competitions, each belonging to one of five autonomous manipulator architectures. The five architectures corresponded to different strategies for decomposing the reference problem, for example, by technical discipline (electrical, mechanical, software) or functional component (gripper, arm). Each module of the architecture was run as a separate challenge. The modules varied in their disciplinary emphasis, scope and complexity. Solvers were permitted to participate in as many challenges as they wished. As a field experiment, this approach prioritized observing the participants’ preferences for different challenges over balancing attempts from solvers with different backgrounds. Data were collected for each solver's participation in challenge(s), tracking their Registration.csv, initiating a solution, the design artifacts associated with any submitted solutions and their exit impressions. Human subjects data were collected and stored in accordance with IRB# 031559
Data source location	The challenges were run on Freelancer.com, an online task and open innovation platform. Freelancer.com created a challenge ecosystem for the research challenges which required participants to complete a Registration.csv survey before gaining access to the competitions. All participant interaction with the platform (e.g., downloading problem descriptions) were recorded and final submissions were made through the platform. The platform provided the collected data to NASA who removed individual identifiers (necessary for awarding prizes) before sharing with the George Washington University team, who stored it.
Data accessibility	Repository name: Mendeley Data Data identification number: doi:10.17632/79xc6bkgjt.1 Direct URL to data: https://data.mendeley.com/datasets/79xc6bkgjt/1

## 1 Value of the Data


 

• These data are important to engineering design, systems engineering, open and distributed innovation and expertise research because they provide a cross-section of data previously unavailable. This responds directly to calls for better and more comparable design and solving data. Previously, challenge generated data provides information about the winners and winning solutions; this dataset captures the population of potential solvers (necessary for studying selection mechanisms and understanding broadcast reach) and the population of solutions (giving insight into lower quality solutions and their nature). This makes it possible to link solver characteristics to solution features. It also provides a provides a population of independently generated solutions to the same problem, providing the empirical data necessary to improve measures of novelty, feasibility, variety etc.
• Researchers in engineering design and innovation are the primary anticipated users of this data because they provide a unique basis for studying questions related to design and solver attributes. Systems engineers and scholars of expertise may also find it useful; in the former case to study the impact of architecture on design and solving outcomes; in the latter case they provide micro data to understand the facets of expertise that are linked to solution attributes.
• The dataset includes both raw data, useful in addressing multiple open questions related to the above-described constructs, and some analyzed data in the form of Design Structure Matrices (DSMs) and Functional Hierarchies, commonly used as an input to systems engineering and design research.

## 2 Data Description

**Fig. 1:**
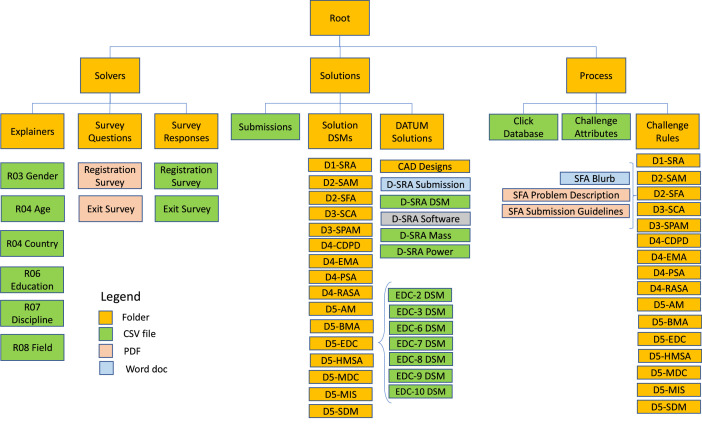
Overview of data folder structure.

The datasets collected from the field experiment are included in the supplementary material as a ZIP file and are also available in the Mendeley Data database: DOI:10.17632/79xc6bkgjt.1. An overview of the data structure is shown in Fig. 1. The main folder had three subfolders “Solvers,” “Solutions” and “Process” corresponding the three macro categories of information we collected. Each record contains either a User_ID or a Solution_ID which can be linked through the “Click Database” in the “Process folder.” An individual user may submit more than one solution, but each solution is associated with only one user.

### 2.1 Solver data

The “Solvers” subfolder contains data about individual solvers’ demographics, knowledge, experience and skills. The subfolder contains three sub-subfolders. The first sub-subfolder, named “Explainers” includes keys for each of the numerical scales in the survey results. Each is in a separate CSV file, for example, there is a file for country codes that match the numerical values to particular countries. The second subsubfolder, named “Survey Questions” contains copies of the Registration.csv and Exit surveys. The Registration.csv survey was administered before solvers were permitted to participate and asks general background questions. The Exit survey was administered after solving, as part of the submission process. This made it possible to ask more specific questions about, for example learning while solving a particular problem. The two surveys are included in the ZIP file. Response to open text answers are not included in to the public repository because too many solvers included personal identifiers in their responses.

The third subsubfolder “Survey Responses” contains two CSV files (Tables 1 and 2). The first CSV file, named “Registration.csv” contains records for the more than 28k potential solvers who began the survey. Many of them stopped before completion. Only those who completed the full survey were given access to the challenges, but many of the partially complete records may be useful for broader population analysis. In the CSV file, column B indicates the completion %. There are 9,641 unique registrants with 100% completion. We did not include the last survey task because of PII concerns, so anyone with 73% or more will have complete demographic records included here. That adds an extra 6,608 solvers. We also included partial records above 40% completion, which include most demographic attributes. After running the experiment, we manually added one record to the file to represent the “user” who generated the NASA reference solutions. This record is coded as User_ID 999999 and is based on an aggregate of the survey responses provided by the NASA design team. The column headers for the CSV file are provided in Table 1.

**Table 1:** Description of columns in CSV file named “Registration.csv”.

Column Header	Description
User_ID	A unique user ID for each user. This is not the same as the alphanumeric code assigned to each user on the Freelancer.com platform and was generated to deidentify the records.
Progress	0-100% Score tracking percentage of survey completed
R03_Gender	Biological sex of respondent [male, female, prefer not to say]
R04_Age	Categorical value denoting age range
R05_Country	Categorical value selected from a drop-down of country codes
R06_Education_X	Multiple column response grid. The first block records a yes/no response to each educational level X (where X is e.g., high school, college) and the second block records an open-text response to the subject of that degree, with a column for each X.
R07_Experience_X	Multiple column response where each column records a yes/no response to each domain of experience X (where X is e.g., medicine, aerospace and defense)
R08_Specialty_X	Multiple column response grid. The first block is a yes/no response to question “have you worked or volunteered in field X” (where X is e.g., robotics, project management) and the second block records the self-reported years in field X.
R10_SpaceRoboticsSimiliarity	Likert Scale, asking “A space robotics problem is…” where response are on a 7-point scale ranging from “inside my field of expertise” to “outside my field of expertise” (= 7).

**Table 2:** Description of columns in CSV file named “exit”.

Header	Description
Solution_ID	A unique identifier for each solution submitted. For a solution, SRA 10, the letters (e.g., SRA) refer to the challenge problem solved and the numbers (e.g., 10) correspond to a specific solution to that challenge
Q4_Problem Similarity	Response to the question “How similar was this contest to projects/problems you normally work on?” on a 5-point Likert scale (where lower is closer).
Q6_EquipmentAcquisition	A yes/no response to the question: “Did you acquire any new equipment or learn any new skills to participate in this contest?”
Q9_DifficultyComparison	Response to the question: “Compared to what you expected, how difficult was this contest?” on a 7-point Likert scale (where lower is easier)
Q10_SimiliarityComparison	Response to the question: “Please choose the level that best fills in the blank. I am ____ with similar problems” on a 7-point Likert scale (where lower is less experienced)
Q15_SelfReport	Numerical response to the question: “How long did you spend solving this problem?” Mixed responses included hours, days, semesters. Q15_Hours converts all units to hours.
Q15_Hours	Processed data converting Q15 response to hours

The second CSV file named “Exit.csv” contains records for the 201 exit surveys that correspond to a unique submitted solution. Although Exit surveys were required for a solution to be judged, not all submitters followed the instructions correctly creating several data validation issues. A small number of solvers circumvented our system in two specific ways: First, some solvers who submitted multiple solutions used the same exit code for all of them. This is a problem because the exit surveys ask questions that are intended to focus on a particular solving experience. There are only a small number of such instances since a relatively small number of solvers submitted multiple solutions, but we are not able to fix this issue. Second, we received multiple solutions without an exit code at all. NASA had some success encouraging solvers to complete the survey so that their submissions could be judged. In some cases, the issue was not recording the exit survey correctly, but using time stamps we were able to reconcile most of those records. Although we applied a strict standard for judging (no code, no judging) we retained the solution record, but there is no corresponding exit survey. Users of the database may still find the solution useful but will be unable to do the full linking for the 18 solution records (out of 219). The column headers for the CSV file are provided in Table 2.

### 2.2 Solution data

The “Solutions” subfolder contains data about each solution that was submitted. The raw solutions were submitted as PDFed design documentation, including CAD drawings, narrative descriptions, and engineering analysis. Since these materials are proprietary and owned by the solvers unless they win or sell their solutions to the platform, we only include extracted performance characteristics and some processed representations useful for research, per our agreement. There are three sections in the Solutions subfolder. First, a CSV file, named “Submissions.csv,” which summarizes the solutions received (described in Table 4). Second, a folder of “Solution DSMs” which contains subfolders for each challenge (e.g., D5-EDC) and then within each of those a Design Structure Matrix (DSM) representation for each solution, organized by challenge. Below we describe the structure of each DSM CSV file in Table 5. Finally, a “Datum Solutions” folder which contains the researcher-generated documents for the NASA reference system.

**Table 4:** Description of column headers in CSV file named “submissions”.

Header	Description
User_ID	A unique numerical identifier for each solver
Solution ID	A unique alphanumerical identifier for each solution
FOM_Units	Units of the Fig. of Merit (FOM) used to evaluate winners
FOM_SelfReported	Value submitted by the user as their estimate of the FOM
Completedness_ Rating	Researcher-evaluated rating of the submission (incomplete, conceptually complete, mixed detail, detailed)

**Table 5:** Illustration of different DSM views based on SRA 15 submission.

The CSV file named “Submissions.csv” includes two measures of effectiveness for each submission. First, a self-reported Figure of Merit (FOM) for each solution that was sufficiently detailed. We only included FOMs for mixed and detailed solutions because we found high variation in the quality of FOM estimates for less complete solutions. Second, a researcher-evaluated completeness level. The procedure for coding this scale (from incomplete to detailed) is detailed in the methods section. The column headers for the CSV file are provided in Table 4.

The DSM folder contains 17 subfolders, one for each challenge. Within each folder are excel files corresponding to each solution of a given challenge that was sufficiently detailed to support the production of a Design Structure Matrix (DSM). Table 3 summarizes the number of DSMs created for each challenge. In all, DSMs were created for 112 unique solutions with five representations of each. DSMs are one of the most popular representational frameworks for designs within Systems Engineering and are the input for many of the lifecycle property measures used in that domain. A detailed description of how the DSMs were created is provided in ref [1]. The files are named consistently with Solution ID used throughout the database. Each CSV file contains five unique DSMs. The first corresponds to the module-level view. The second includes the most detail because it contains all disciplines and detailed components. The third includes all disciplines but removes fasteners. The fourth adopts assumptions stated in the standard Tilstra method [2]. The fifth leverages Tilstra but with fasteners removed. We included all 5 representations because each might be useful for different research questions. Table 5 illustrates the five views present in the “DSM” folder.

**Table 3:** Overview of solution data included.

	Challenge	Submitted	Judged (Detailed, Mixed)	System Architecture Representations (DSM) Made
D1	SRA	27	18 (11, 7)	10
D2	SFA	10	8 (3, 3)	4
	SAM	16	14 (5, 6)	8
D3	SCA	10	9 (3, 3)	3
	SPAM	20	15 (6, 5)	5
D4	EMA	23	17 (12, 1)	9
	CDPD	10	5 (1, 3)	4
	RASA	6	6 (4, 1)	4
	PSA	8	7 (3, 2)	3
D5	AM	21	19 (13, 4)	13
	EDC	13	11 (9, 2)	7
	MDC	18	18 (14, 1)	14
	MIS	16	15 (14, 0)	N/A
	HMSA	8	7 (5, 0)	5
	SDM	25	18 (8, 5)	13
	EBD	24	20 (14, 2)	10
	BMA	8	7 (3, 1)	N/A

Total	17 Challenges	263	211	112

The Datum folder contains a submission file that simulates what the internal NASA solution would look like if it were responding to the Astrobee Challenge Series challenges as posed, subject to the same submission guidelines. It was created by the second author using the same design principles and materials included in the NASA documentation. It includes all the views and diagrams that are in other crowd-generated solutions. The DATUM SRA solution was created first and then modified to generate DATUM solutions to most of the challenges. Some challenges were not created when there was insufficient relevant documentation from the NASA team, for example for many of the D5 discipline-focused challenges. In all of the files and representations below, any solution with the ID of all 9s refers to the DATUM or its 13 subDATUMs.

### 2.3 Process data

The “Process” subfolder contains data about administering the challenges and stages of solving. There are three subsections: A CSV file named “Click database.csv,” another CSV named “Challenges.csv” and a folder that includes all the Problem Description and Submission Guideline documents (i.e., the rules) for each challenge.

**Fig. 2:**
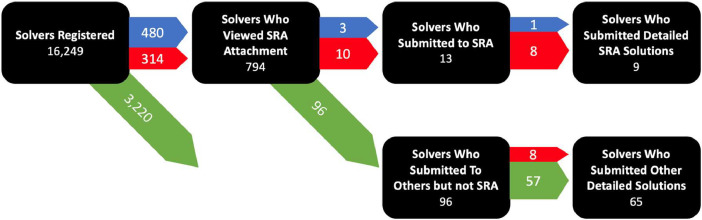
Flowthrough of SRA participant engagement (Blue lines represent only SRA, Red lines represent SRA and Others, Green Lines Represent Only Others from SRA).

Table 6 describes the headers for the “Click database” CSV file. The challenge series was instrumented so we could track level of engagement on the platform by each of the participants. When potential participants registered, Freelancer.com assigned a token to their profile and recorded each time they took an action in the challenge ecosystem. Actions progressed from registering to downloading detailed information about one (or many) challenges, to submitting a solution to a subset of those challenges. In the aggregate this makes it possible to view flow through the system. An example is shown below in Fig. 2.

**Table 6:** Description of column headers in CSV file named “click database”.

Header	Description
User_ID	A unique identifier for each solver
Challenge	The abbreviation of the relevant challenge (e.g., SRA)
Viewed_Attachment	A yes/no binary of whether the user downloaded the attachments necessary to begin a submission to the relevant challenge.
Submitted	A yes/no binary of whether the solver submitted a solution to the challenge
Solution_ID	A unique identifier for each solution, if submitted

Minimal processing was performed on the Freelancer.com record. To fully remove Identifying information, the Freelancer.com token was replaced by an alphanumeric User ID. Otherwise, the data was cleaned to remove obvious duplicates. When we first received the dataset we noticed that there were instances when multiple records were created by a single action (for example, 10 entries when someone clicked on the description for a particular challenge, likely because they didn't save the file and instead revisited the web version). We chose to include one click per solver (the first) on a given challenge. We performed several consistency checks on the data. We checked to make sure every document flowed from Registration.csv to download to submission. Among the 11,541 clicks, the Freelancer platform only logged four unknown accesses (i.e., action without Registration.csv) to the EBD challenge series (0.03%). We did not find any submissions without clicks. We chose not to insert DATUM records into the click database. If a researcher wants to consider user 99999 (the NASA solutions), it would be appropriate to consider that they downloaded all 13 (including SRA) challenges to which they submitted and no others.

Table 7 describes the headers for CSV file “Challenge.csv.” It contains a record of the timelines on which challenges were administered. The final folder contains subfolders for each of the 17 challenges. Each includes two files: 1) the corresponding problem descriptions 2) and submission guidelines that were generated as part of the challenge. This provides useful context for researchers wishing to make an independent assessment of the difficulty and complexity of the challenge problems.

**Table 7:** Description of column headers in CSV file named “challenge”.

Header	Description
Challenge	Abbreviation of the relevant challenge (e.g., SRA)
Architecture	The alternative architecture it belongs to (e.g., a module in D1)
Start_Date	Start date of the competition XX/XX/XXXX
End_Date	End date of the competition XX/XX/XXXX
Prize_level	Prize level in dollars
Quality_Type	Winning criteria (reported as mass as Kg or lines of code as LOC depending on the challenge)
Duration	Duration in days

## 3 Experimental Design, Materials and Methods

**Fig. 3:**
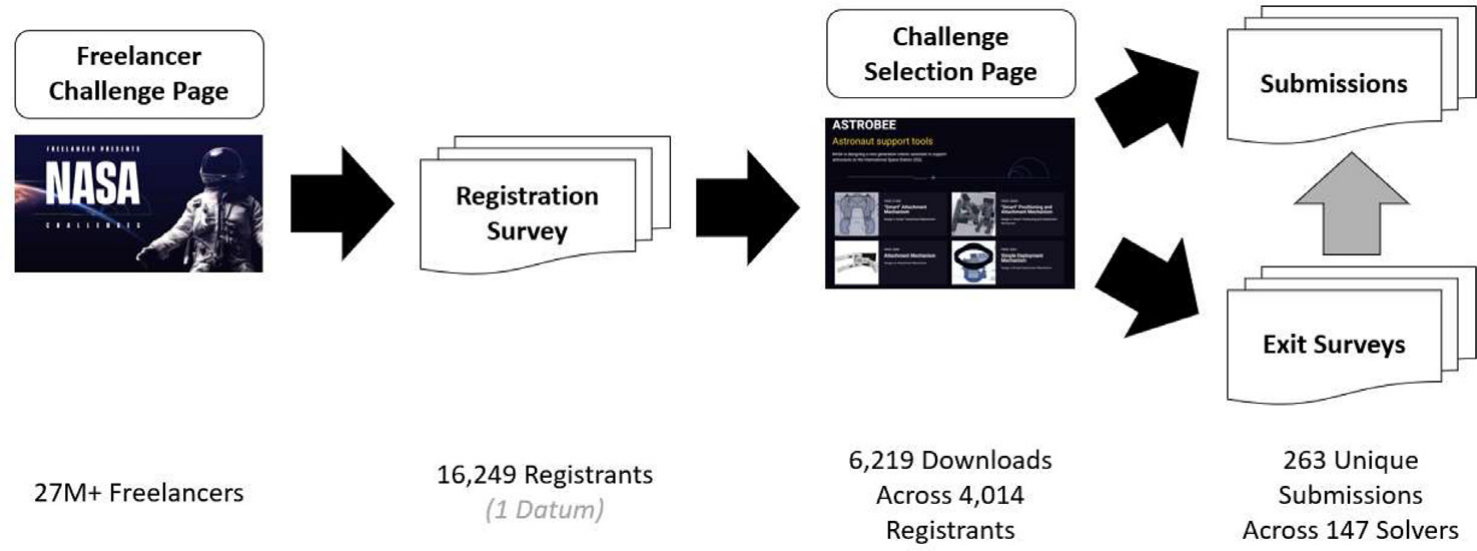
Challenge administration and data collection process.

This section describes the experimental procedures used to collect the data contained in the repository. The experiment was structured around a technical reference problem that involved the design of an autonomous robotic manipulator for use on the International Space Station. This was a problem that NASA was already working on internally. The experiment manipulated how the problem was *formulated*, introducing four alternative system architectures and framing innovation contests around the associated modules or subproblems. For example, one problem was presented as the full autonomous manipulator, where another isolated the mechanical aspects of the gripping mechanism. In all, 17 individual contests were run over a period of 12 months. We chose to run contests in part because of our general interest in Open Innovation as a tool, but also because it is a means to generate multiple independent design solutions to a reference problem. Additionally, because of the nature of an online innovation platform, it lends itself to non-invasive observation of solver engagement in the work. Other than the problem framing manipulation, the contests were run consistent with the norms of the Freelancer.com platform. The overall process flow is shown in Fig. 3.

This section is organized as follows: First, we describe the reference space robotics system problem and explain why it was selected. Second, we describe how the problem was re-formulated to support an open innovation experiment and summarize the resultant 17 subproblems that were run as contests (the detailed subproblem specifications are contained in the repository). Third, we describe how the overall challenge series was administered, in terms of what data was collected and when. It is worth noting that we collected more data than can be shared in a public repository due to the proprietary nature of technical designs. To maximize what can be shared about the solutions, where feasible, we are sharing processed versions of the reserved raw data. That processing is described in detail in the data records section.

### 3.1 Reference design problem: NASA's Astrobee perching arm

Each contest related to the design of a moderately complex robotic system, capable of autonomously attaching to an International Space Station (ISS) handrail and executing positioning commands. This particular robotics problem was chosen because NASA was also solving it internally and it matched the research need of being representative of real-world complex design problems. Additionally, since this system featured mechanical, electronic, and software design features, it could be decomposed in several potential ways (i.e., both hierarchically and disciplinarily).

**Fig. 4:**
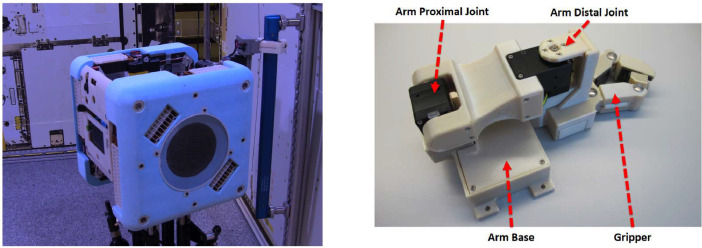
Astrobee Robotic Freeflyer (Left), Perched on an ISS Handrail (Middle), and Detailed View (Right) From [4].

The internal-to-NASA project is the Astrobee Perching Arm System. It is a robotic manipulator designed to support on-orbit Astronaut operations inside the International Space Station (ISS). The perching arm works with the Astrobee Free Flyer and is stowable in its payload bay [3], [4], [5]. The perching arm augments Astrobee operations by providing a stable “perch” on ISS handrails (see Fig. 4), which reduces power consumption by the system and supports ideal camera viewing angles [6,7].

The perching arm was designed by a team of roboticists at the NASA Ames Research Centre and is currently flying on the ISS. The arm system as flown featured a two degrees of freedom manipulator and passive clamping claw with compliance so it could detach easily in an emergency. The arm provides panning and tilting functionality to the Astrobee Free Flyer and attached cameras. The NASA design is captured in multiple papers [3], [4], [5], [6], [7].

### 3.2 Alternative problem formulations

Problem formulation is the main manipulation in this field experiment. We wanted to understand if the scope and framing of the problem impacted who solved and how they solved. To test the impact of this manipulation, we needed a selection of alternative formulations, which were variations on an “open” version of the reference problem.

#### 3.2.1 D1: the open version of the Astrobee reference problem

In this context, opening the original problem of how to attach the Astrobee Free Flyer to an ISS Handrail primarily involved re-writing NASA's internal requirements in a way that could be accessible to non-NASA and non-robotics solvers. This primarily involved changes like simplifying and making explicit environmental requirements, such as the amount of debris a space robotic system can make, or the material selection to prevent off-gassing, which are sometimes taken for granted within a domain. It also involved simplifying the geometric complexity of the physical interface to the Astrobee Platform to reduce the need for Computer Aided Design (CAD) tools to produce a compliant design solution. CAD tools, in addition to requiring specialized knowledge, are expensive, so requiring them might have unnecessarily limited who could solve. Finally, we defined a workspace for the manipulator so as to eliminate its reliance on the Astrobee free flyer for coarse positioning and other related capabilities.

This explicit allocation of functions to the robotic manipulator means that one needs to be careful comparing the open version of the arm subsystem to the internal problem, since it is part of a larger arm-free flyer system. While the problems are similar, to create a fair reference, we created a DATUM version of the Astrobee perching arm which adopts the same design principles and materials used by the NASA Ames team but is designed to solve the exact challenge as broadcast. The detailed design for this DATUM is included in the “Solutions” folder in the repository in the “DATUM Solutions” subfolder.

#### 3.2.2 Challenge problems

**Fig. 5:**
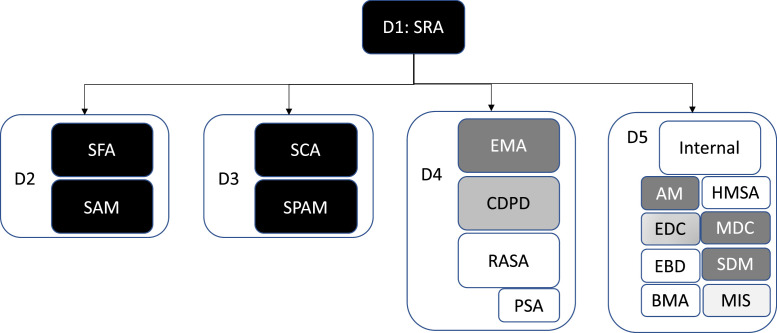
Alternative decompositions. Black shading denotes interdisciplinary challenge, dark grey is mechanical only, middle grey is electrical only, white is computational only, gradient is a defined specialty.

Once the reference system (D1) had been created, we developed four alternative system architectures (D2-5). First, we created the most standard modularization from industry: D2 separates the perching arm into a manipulator and a gripper, essentially separating the “hand” (gripper) from the rest of the “arm” by breaking the system at the wrist. This required a formal requirements allocation across the two modules and a new interface plate to be designed, featuring power and data connectors as well as a decomposed concept of operations. Second, we created another two-module decomposition which instead breaks the system below the “shoulder” joint (D3). This results in a simpler arm and assigns more functionality to the gripper. Third, we performed a disciplinary decomposition which aimed to isolate the mechanical from the power electronics from the software and control elements of the system (D4). Finally, we introduced a decomposition that prioritized isolating subfunctions that might be particularly amenable to external solving (D5). For example, we created a challenge focused on reducing slipping on the gripper surface that might reasonably be solved by a material scientist, and a health monitoring challenge that would be familiar to hardware-focused programmers from any discipline. These alternatives are summarized in Fig. 5. In all, 17 unique challenges were administered across five architectures. In the remainder of the document, we will be referring to the challenges by their acronyms, summarized in Table 8.

**Table 8:** Challenge acronyms.

Acronym	Title	Acronym	Title
SRA	Smart Robotic Arm	AM	Attachment Mechanism
SFA	Smart Fine-positioning Arm	MDC	Mechanically Driven Clamp
SAM	Smart Attachment Mechanism	EDC	Electrically Driven Clamp
SCA	Smart Coarse-positioning Arm	HMSA	Health Monitoring SA
SPAM	Smart Positioning Attachment Mechanism	MIS	Material Interface System
EMA	Electro-Mechanical Arm	EBD	Electronic Box Design
CDPD	Command, Data and Power Distribution System	BMA	Box Mechanical Analysis
RASA	Robotic Arm Software Architecture	SDM	Simple Deployment Mechanism
PSA	Positioning Software Architecture		

#### 3.2.3 Problem descriptions

**Fig. 6:**
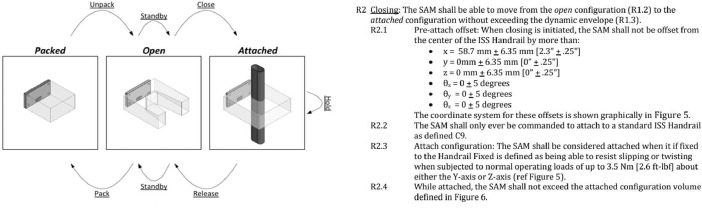
Right, image from SAMs problem specification illustrating the sequence of functions specified by the requirements (Left).

For each module we generated an associated problem specification and solution guideline. Each of these documents are included in the repository. The aim with the problem specification was to define solution-agnostic requirements and write them in a language that was accessible to potential solvers without a background in the space sector and ideally without expecting an engineering background. An example of the type of language is provided below in Fig. 6. Note that the writing is still *technical*, though not specific to a particular domain. Where feasible, pictures were used to clarify concepts and referenced by specific requirements.

**Fig. 7:**
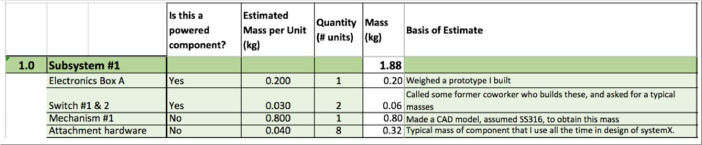
Excerpt from SAM's submission guidelines the mass template and guidance.

The aim with the solution guideline was to provide a standard format for communicating design information without over-constraining the type of analysis that could support the solution. For example, we provided a template in both excel and google sheets for calculating a power budget to guide solvers in providing the requisite information. An example is shown below in Fig. 7. The full submission guidelines for each challenge are included in the repository.

### 3.3 Administering the challenge series

Other than manipulation of the challenge problem, we ran the challenge series as would otherwise be done on the Freelancer.com platform. Freelancer.com is a web-based platform that connects “freelancers” (i.e., people willing to work) to tasks and includes a capability to host prize-based contests. Freelancer.com is one of the largest open innovation platforms in the world, boasting an active solver base of more than 27 million active users at the time of the competition (as of 2022 they have over 60 million). It is free to join which meant that NASA could market to the general public to attract a broader potential audience. We chose Freelancer.com because it has a more diverse solver base than other platforms,^1^ which enabled us to run all mechanical, electrical, computational and interdisciplinary challenges in one ecosystem.

**Fig. 8:**
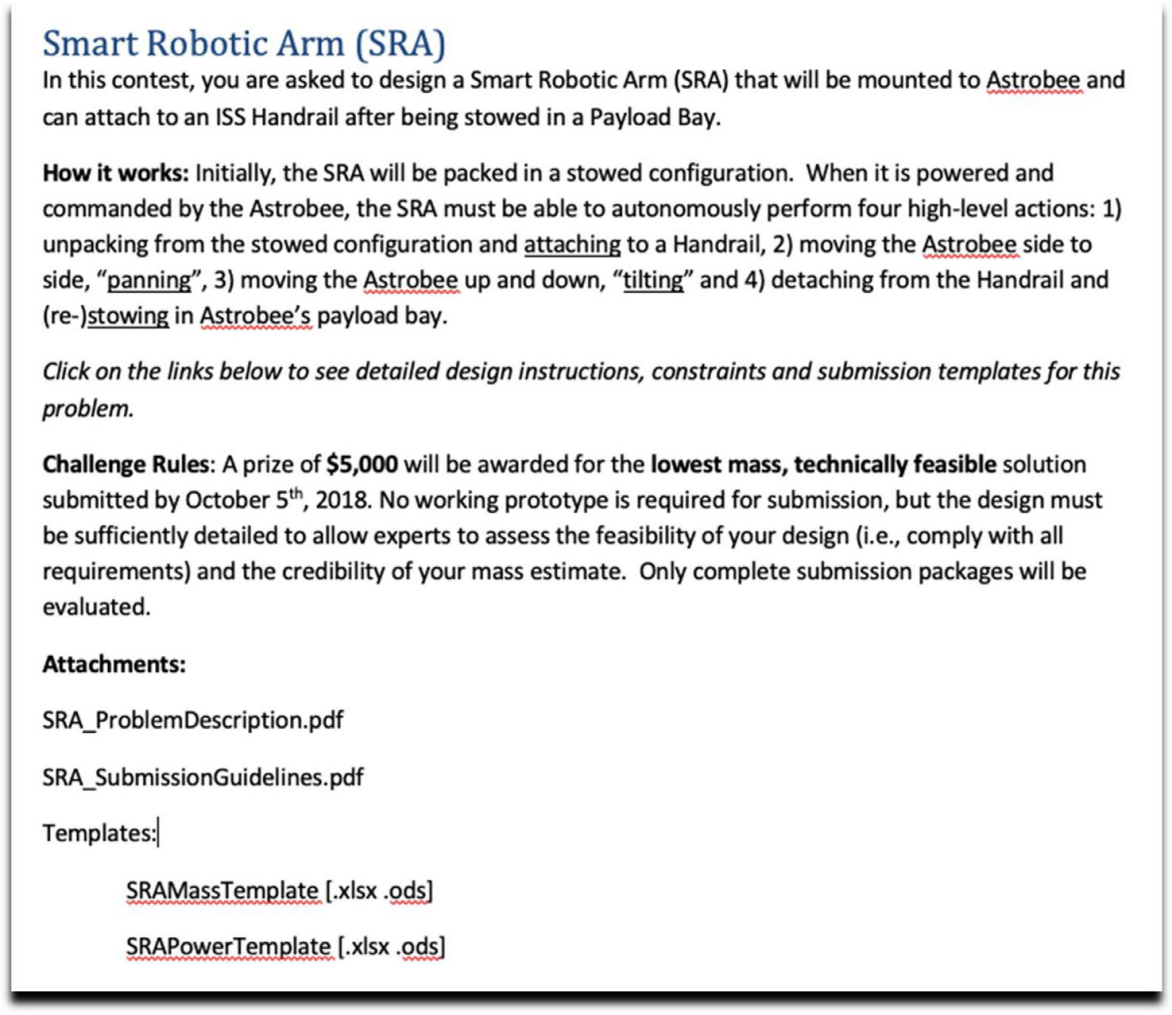
Example of a brief challenge description.

In a typical contest, a seeking organization (the one with the problem) posts a short, one paragraph description of their need and offer on the platform's website and advertises it to the crowd. In our case, NASA was presented as the seeking organization, and we advertised through Freelancer's listserv and NASA's “solve” twitter account. An example of a short description is shown in Fig. 8. Each includes a brief statement of the required functionality, how the challenge will be evaluated (i.e., the figure of merit (FOM)) and the prize level.

The challenge page was set up so that any potentially interested Freelancer could signal their interest by registering for the challenge series. This would give them access to view all of the related challenges and give us a means to track their participation in our ecosystem. The Registration.csv process is described in more detail below. Once they registered, they could peruse the brief descriptions and “click” for more detail on any challenge that piqued their interest. Clicking brought up a page with links to the detailed problem description, solution guidelines and associated templates (described above).

Consistent with platform norms, all challenges had a prescribed time limit ranging from two weeks to two months and a chat/forum feature where participants could ask clarifying questions and request more time. As is typical on this platform, challenge deadlines were extended by one to two weeks when enough requests came in from solvers asking for an extension.

Participants were able to submit to as many challenges as they wished and multiple times for each challenge. Submissions took the form of a PDF document that responded to the submission guidelines and were required to include a completion code from an exit survey, which captured more detailed information about their solving experience (e.g., did they need to learn anything new to submit). A detailed description of the exit survey is provided below.

#### 3.3.1 Timing of the contests

Ideally, we would have wanted to run all contests simultaneously so that participants could pick directly among all the options. However, this wasn't feasible due to limited evaluation capacity. Per Freelancer guidelines, all prizes must be awarded within two to three weeks of contest close. Given the technical nature of these paper studies, evaluating the quality and feasibility of the solutions was quite labor-intensive – the research team spent an average of a day on each solution, sometimes much longer. Out of necessity, we staged the contest end dates to limit periods where we would be evaluating solutions from more than one contest at any given time. To mitigate the impact of this constraint, we launched challenges in waves so that participants would still have multiple challenge options at any given period. The sequence of challenges mixed problem scope and disciplinarity in every wave. For example, a wave might include an autonomous electromechanical component (multi-disciplinary, high scope) a higher complexity, but a mechanical only challenge (single-discipline, high scope) and a distant discipline focused challenge (other-discipline, low scope) so that participants would still be prioritizing what to work on.

**Fig. 9:**
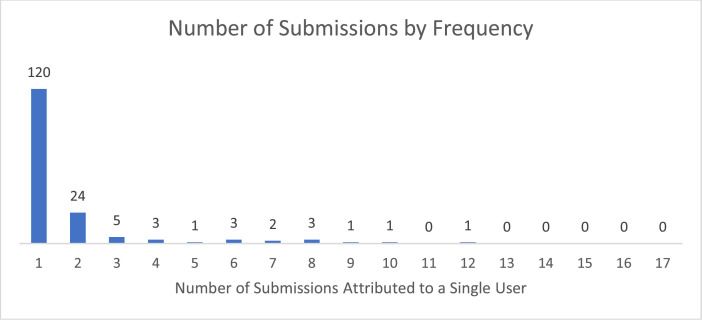
Count of submissions by user. the vast majority of participants submitted no solutions (not shown) and of those who submitted something, over 70% submitted only submitted one.

**Fig. 10:**
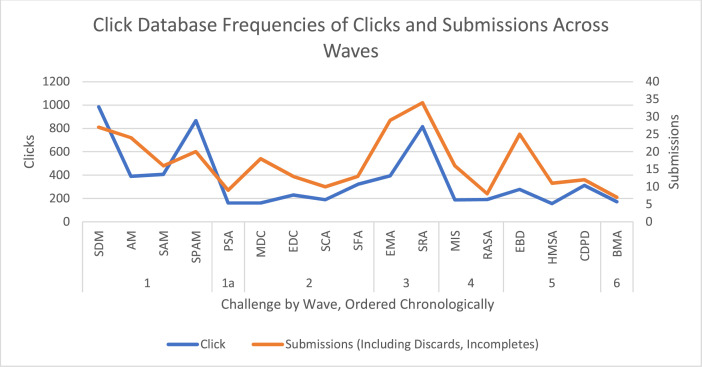
Count of clicks (left axis-bar plot) and submissions (right axis-line plot) ordered chronologically by competition wave.

All of the challenges were completed over a period of 12 months between May 2018 to May 2019. Table 9 shows the schedule of which contests were launched when. There are two important methodological concerns with launching the challenges in this way. First, we might see significant re-use from one challenge to another since all challenges are variants of a reference problem. We did see this to some extent, with some participants reusing their solutions to earlier challenges. However, while a small subset of solvers participated in multiple challenges, the modal number of contributions was one (see Fig. 9). Second, we were concerned that there would be a drop-off in participation over the course of the challenge series. This was not evident in the data (see Fig. 10). While there was variation in participation across challenges, there is no apparent temporal trend.

**Table 9:** Schedule of challenges.

Wave	Challenge Name	Prize value	Start Date	End Date	Duration (Days)
Wave 1	SDM	$250	5/31/2018	6/18/2018	18
	AM	$500	5/31/2018	6/25/2018	25
	SAM	$1500	5/31/2018	7/2/2018	32
	SPAM	$4000	5/31/2018	7/19/2018	49
Wave 1a	PSA	$500	6/28/2018	7/26/2018	28
Wave 2	MDC	$250	7/18/2018	8/6/2018	19
	EDC	$250	7/18/2018	8/13/2018	26
	SCA	$1500	7/18/2018	9/1/2018	45
	SFA	$4000	7/18/2018	9/12/2018	56
Wave 3	EMA	$4000	8/10/2018	9/24/2018	45
	SRA	$5000	8/10/2018	10/31/2018	82
Wave 4	MIS	$500	10/17/2018	11/8/2018	22
	RASA	$1500	10/17/2018	11/14/2018	28
Wave 5	EBD	$250	2/8/2019	3/4/2019	24
	HMSA	$250	2/8/2019	3/4/2019	24
	CDPD	$1500	2/8/2019	3/28/2019	48
Wave 6	BMA	$250	4/29/2019	5/16/2019	17

#### 3.3.2 Setting prize levels and contest length

The prize levels were set following the standard procedures for the platform. Domain experts – in this case, two members of the research team with combined more than 20 years of space robotics experience – estimated the time it would take an industry professional to respond to the contest. There was a high level of agreement, and we used the average of the two estimates. These hour values were multiplied by a representative US wage and then examined for consistency with other contests being run on the platform.

There is a relatively large literature on prize levels inducing participation and we were initially concerned that varying prize levels would skew results. At the same time, not offering different prize levels for very different levels of effort would also skew the results [8,9]. Our NASA collaborators shared their experience that it was most important to match the norms of the platform – in this case ensuring a minimum prize level ($250) and not exceeding the current max, which could indicate a very difficult problem. Balancing this input, we used the upper and lower bounds of $250 and $5000 and scaled the individual values based on relative effort. Table 9 shows the prize levels for each of the competitions.

#### 3.3.3 Seeker-solver interaction during the competition

Freelancer.com's contest implementation allows solvers to post messages to the seeker during the competition. Questions typically involve technical clarification, request for more information, complaints about the challenge and/or requests for more time. The research team responded promptly to every request through the NASA account and kept a record of the communications. The responses are visible to challenge participants so that no unique information was provided to any particular solver. We were also careful not to suggest solving strategies in the answers. These are not included in the database since many solvers included difficult to de-identify information. Examples are provided below in Table 10.

**Table 10:** Example questions asked.

Challenge	Question	Response
SDM	Hello, can two motors be used? Or are you looking for a mechanism that satisfies the requirement with a just single actuator source?	You may use any number and any combination of mechanisms and motors you desire as long as they meet energy and SDM volume requirements.
SPAM	Can we assume that the SPAM move on only X-Z plane since the handrail is in the direction Y. Or do you expect to consider the movement of the SPAM in y direction within -45 to +100 mm	The SPAM may move in that way so long as it is able to attach to the ISS Handrail. A solution that only moves in a few directions, but can still attach the Handrail when commanded, is acceptable.

#### 3.3.4 Survey design

Two surveys were used to collect information about solver's demographics, background and experience participating in the challenges. The complete Registration.csv survey – taken before participating – and exit survey – taken after completing the challenge as part of the submission process – are included in the database. Most of the questions are standard demographic measures or explicit self-reports (e.g., “did you acquire any new equipment or learn any new skills to participate in this contest?”). The questions focused on measuring the theoretical concept of distance, replicating the scale and phrasing from [9]. The Registration.csv survey also included a multi-dimensional scaling experiment as Q15 and later, which is not included in this data release.

#### 3.3.5 Evaluating solutions and awarding prizes

The challenge description for each contest included an explicit statement of how the prizes would be awarded. In most cases, it took the form of A) meets all the requirements and B) performs best on the stated figure of merit (e.g., lowest mass, lowest Lines of Code (LOC)). Since submissions were received as paper documentation, each of these criteria required expert evaluation. First, when the challenge closed, we received raw submissions, typically in PDF form. Submissions were ordered based on their self-reported figure of merit (FOM). Then, starting from the best FOM, experts performed two separate evaluations. Solutions were assessed for whether the self-reported metric was credible. This is not the same thing as assessing if it is correct, which would have required independent analysis, but rather solutions were judged in terms of the basis of estimate. For example, if a solver reported that the estimate came from a CAD model, we deemed the estimate credible. Similarly, if a detailed bill of materials was included with component estimates provided, it was deemed credible. In some cases, there was no basis provided or there were obvious gaps in what was counted, making it easy to remove the solution from consideration. If a high-ranking estimate (that is, having the lowest mass or Lines of Code) was credible or likely credible, the experts performed the second evaluation of whether the solution met the requirements. This is a difficult assessment for any paper study, but a process leveraging the design of the submission guidelines was adopted. These included several plausibility probes, which were chosen to capture critical requirements and gave confidence in the quality and efficacy of the design. In some cases, external domain experts were consulted for third and fourth opinions.

If the team agreed and was confident that the submission was highly likely to be feasible and had the highest ranking FOM of the set, a prize was awarded. After a prize was awarded, the solution was published to the freelancer site, per their platform norms. This typically included the freelancer profile as well as their submission to the competition. As a result, winning solutions are not associated with any of the records in this databased since the risk of re-identification is high. It is worth noting that the best FOM rarely passed both evaluations, so even though FOMs are reported, users of the database should not assume a simple ranking would reveal the winner.

Since quality is an important attribute to include in the database, a different measure is provided in the database. Multiple measures of quality were explored, settling on a scale that integrates notions of both completeness and performance [10]. Self-reported Figure of Merit (FOM) is also included, but only for those that are sufficiently complete to be likely credible. This is because our review of the solutions revealed inconsistency in the quality of those estimates, correlated to completeness. Specifically, more unusual, and less elaborated solutions also tended to have non-credible FOMs. Therefore, rather than trusting the self-reported FOM as a measure of quality across submitted solutions, we qualitatively coded each solution.

Solutions were coded for quality in terms an expert-rated completeness scale, previously reported in [10]. Although completeness is not a direct proxy for quality, it has previously been used to capture related features [11], demonstrated to be a useful signal of quality in other studies. The research team, in consultation with NASA, performed all coding on the completeness scale. Even among discipline experts, assessing solution quality based on a “paper” study (i.e., a PDF with no physical artifacts) is inherently subjective,^2^ and initial coding efforts revealed this challenge. Therefore, the completeness scale detailed in [10] was defined to shift the assessment from “feasibility” to “is the solution detailed enough that a domain expert could implement it.” Once the standards of completeness were defined, consistency was significantly improved. Each solution was coded as detailed, mixed, conceptual, incomplete, or unsure, as seen in Table 11, by one coder. Then two additional coders were recruited and assigned an overlapping subset of the “unsure” codes and a random selection of the other solutions. Overall, each solution was coded by at least two coders. Across 263 solutions (and the associated 3000 pages of technical documentation) there were substantive disagreement on fewer than 20 solutions. All disagreements were satisfactorily resolved through team discussions.

**Table 11:** Levels of quality for submissions.

Quality	Description	Example
Detailed	The design of the entire system has been specified (i.e., all components have been selected or sized and their relationships defined)	The designer had a full specification and answered all of our submissions guidelines
Mixed	Detailed design work has been completed in at least one aspect of the design, but other aspects remain at the conceptual level.	The designer included images of their Computer Aided Design (CAD) files, but had no specifications for electronics for a challenge that required it.
Conceptual	The conceptual design is complete; with additional design work it could become a detailed design.	The designer provided us an image of a robotic arm that they sketched that could meet the competition and answered a few questions.
Incomplete	Solution has design content, but it is not responsive to the problem as posed.	The designer uploaded a picture of a robotic system.

## 4 Limitations

Specific limitations related to data collection and curation were described above as they relate to specific data records.

## Ethics Statement

The data collection protocol used in this field experiment received an excempt Registration.csv from the George Washington University (IRB # 031559) under DHHS regulatory Category 5. The authors declare that the protocol was followed as approved.

## CRediT authorship contribution statement

**Zoe Szajnfarber:** Conceptualization, Methodology, Writing – original draft, Writing – review & editing, Supervision, Funding acquisition. **Anthony Hennig:** Methodology, Investigation, Data curation, Writing – original draft, Visualization. **Suparna Mukherjee:** Investigation, Data curation. **Steven Rader:** Resources, Project administration. **Jason Crusan:** Conceptualization, Resources.

## Declaration of Competing Interest

The authors declare that they have no known competing financial interests or personal relationships that could have appeared to influence the work reported in this paper.

## Data Availability

Open Innovation Generated Robotic Design and Solver Characteristics Dataset (Original data) (Mendeley Data).

## Appendix Supplementary materials

Image, application 1

## Footnotes

1 For example, GrabCAD has a strong mechanical design community but few solvers capable of tackling algorithm challenges. Conversely, TopCoder has curated a community of highly capable programmers, but has limited experience with mechanism design. MTurk is diverse, but tends to attract contributors with less technical training.

2 See for example, [12], where coders showed strong agreement on all measures except quality.
