# Supplementary material for: Linking solver characteristics, solving processes and solution attributes: A data explainer for an open innovation generated robotic design dataset
Source: Data Brief. 2023 Sep 6;50:109547. doi: 10.1016/j.dib.2023.109547 (PMC10518673; doi:10.1016/j.dib.2023.109547)
Supplement: Supplementary file 1 [file mmc1.zip › Release/Solutions/Datum Solutions/SRA Datum Submission.docx]

**Submission Guidelines for the Smart Robotic Arm**

In this contest, you were asked to design a “Smart” Robotic Arm that will be mounted to Astrobee and attach to an ISS Handrail

This document provides detailed guidelines on how you must describe and present each aspect of your design in order to be considered for the prize. This document looks long but very little text is required. Your submission document must include each of the sections detailed below and all of the information requested in each. Several templates and examples are provided to clarify what constitutes a complete solution.

**Table of Contents**

1 Submission Summary 2

2 Functional Description 2

2.1 Narrative (word) description of design 2

2.2 Functional Analysis 2

3 Mass Summary and Component List 3

4 System Layout 4

5 Design Drawings 5

6 Software Description 6

7 Power Usage Description 7

8 Exit Survey 8

# Submission Summary

| Mass (kilograms) | 0.47 kg |
| --- | --- |
| Exit Survey Confirmation Code | 32593 |

# Functional Description

## Narrative (word) description of design

1. Attach: How does your SRA attach to the ISS Handrail from a stowed configuration (R1)? Can your SRA attach to the ISS Handrail in all eight corners of the ISS Workspace?
2. *First, the system receives the command from the Astrobee main computer.*
3. *It begins by extending the turret platform directly upward.*
4. *Then, the forearm swings around, and the gripper swings around then to align itself towards the handrail.*
5. *The gripper motor then actuates to open up the gripper unit*
6. *The turret base and the forearm pan motor then work simultaneously to align the gripper with the handrail*
7. *The turret base, forearm gripper, and extension base them move simultaneously to extend the gripper in planar motion towards the handrail*
8. *Once the computer calculates its final position and it’s on the handrail, the gripper motor deenergizes, closing the gripper on the handrail through its naturally closed spring actuation.*
9. Orient - Pan: How does your SRA move Astrobee side to side (R2)?

*The forearm pan motor moves in planar, side to side motion.*


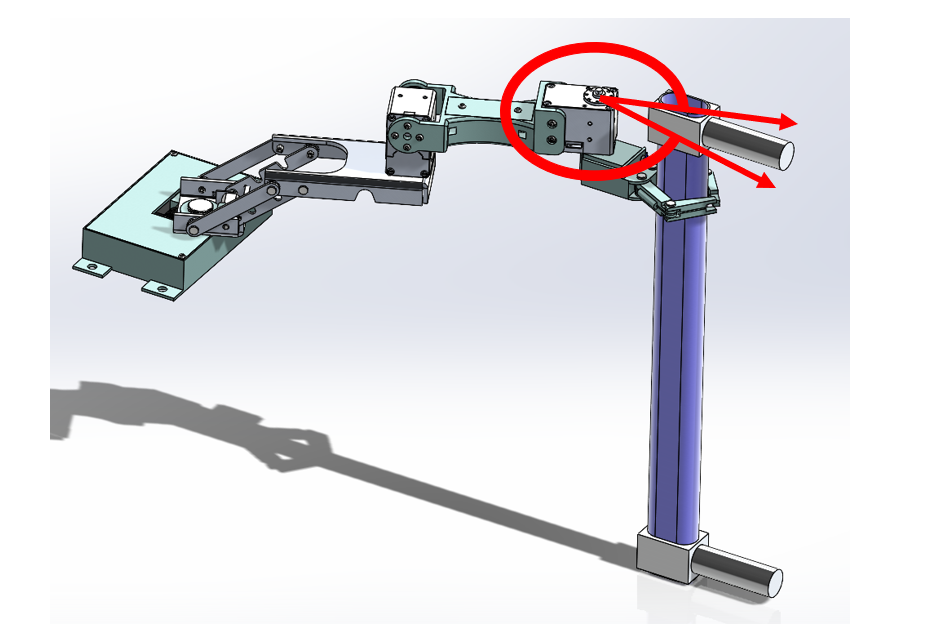


1. Orient - Tilt: How does your SRA move Astrobee up and down (R2)?

*The forearm tilt motors move in up and down motion*


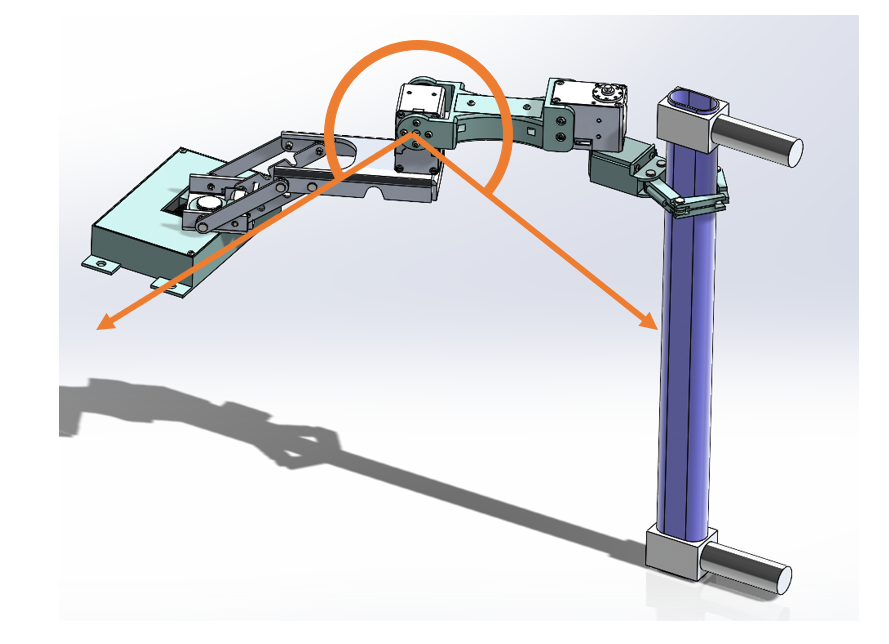


1. Stow: How does your SRA stow itself into the Astrobee payload bay (R3)?
2. *The system receives the command*
3. *It opens up the gripper motor*
4. *It then moves the turret base, forearm gripper, and extension base in a planar motion away from the handrail*
5. *The gripper motor closes*
6. *Then, the forearm folds up, first by rotating the gripper motor around, and then flipping the robotic arm along the tilt motion.*
7. *Then, it realigns the turret base to the stow position*
8. *Then, the platform descends back into the stowage position*
9. How does your SRA release from the ISS Handrail when the astronaut pulls away (R22)?

*The springs in the gripper make gripping passive and spring driven. If an astronaut pulls it away, it opens up.*

1. How does your SRA respond to being commanded to attach at a location, but no ISS Handrail is present (R23)?

*It will just close the gripper and will not damage itself.*

1. How does your SRA power and control its active components (e.g. actuators, sensors)?

*The two high precision actuators, the Dynamixel used for accurate pan and tilt, have internal control circuits. The remaining actuators, the two Polulu gear motors for the platform extension and rotation, as well as the SG-90 servo used, have internal sensors for position and speed control.*

1. If you have moving joints, how are power and control signals passed across each joint’s range of motion?

Cable routing is handled inside of numerous components, and we have a set of power and data lines that go to every component from the control board main bus.

## Functional Analysis

In this section, describe your logic and/or analysis for the following aspects of your SRA design. Including equations and mathematics is acceptable if it helps clarify the logic behind your design, but please ensure that it will be understood by our reviewers by annotating your process or describing the math being done and why.

1. What is the typical force you expect the SRA will exert on the Handrail while *attached* (R1 and R2)?

Torsional springs for the gripper have been selected such that 4Nm of gripping strength will be applied to the handrail.

1. How much force is applied to the ISS Handrail while *attached* and excessive loads are applied (R21)?

Due to the fact that the gripper is passively capturing the handrail with the springs, that grip force will not be greater than 4Nm or 20N

1. What is the location of your Center of Rotation in SRA Reference Frame coordinates for Pan and Tilt operations (R2)?

There are two locations for the center of rotation for pan and tilt. The pan axis is located approximately 35-40mm from the handrail. The tilt axis is approximately 150-155mm from the handrail and is supplied through the forearm tilt joint.

1. How long does it take your SRA to attach (R1)? Or stow (R3)?

In total, we expect 215 seconds, and no greater than 240 seconds.

| Step | Actions | Time |
| --- | --- | --- |
| 1. *First, the system receives the command from the Astrobee main computer.* | Process, path finding, system startup | 30 seconds |
| 1. *It begins by extending the turret platform directly upward.* | Single motion of the extension Polulu | 15 seconds |
| 1. *Then, the forearm swings around, and the gripper swings around then to align itself towards the handrail.* | Motion of the Dynamixel for tilting with concurrent motion of the base turret Polulu | 15 seconds |
| 1. *The gripper motor then actuates to open up the gripper unit* | Simple SG-90 gripper servo movement | 10 seconds |
| 1. *The turret base and the forearm pan motor then work simultaneously to align the gripper with the handrail* | Simultaneous movement of the pan Dynamixel and turret base Polulu | ~30 seconds (depending on the orientation of the handrail to the system, no more than 45 seconds for extreme ranges) |
| 1. *The turret base, forearm gripper, and extension base them move simultaneously to extend the gripper in planar motion towards the handrail* | Simultaneous movement of the pan Dynamixel, turret base Polulu, and extension Polulu | ~30 seconds |
| 1. *Once the computer calculates its final position and it’s on the handrail, the gripper motor deenergizes, closing the gripper on the handrail through its naturally closed spring actuation.* | Deenergize of SG-90 grip servo | 5 seconds |

#

# Mass Summary and Component List

|  |  | **Powered?** | **Estimated Mass per Unit (kg)** | **Qty** | **Mass (kg)** | **Basis of Estimate** |
| --- | --- | --- | --- | --- | --- | --- |
| **1.0** | **Base Module** |  |  |  | **0.1287** |  |
|  | Baseplate |  | 0.039 | 1 | 0.04 | ULTEM-9085 3D printed with 80% infill - CAD Model |
|  | Polulu Gear Motor (Turret) | Yes | 0.010 | 1 | 0.01 | Catalog Lookup |
|  | Polulu Rotation Sensor | Yes | 0.005 | 1 | 0.01 | Catalog Lookup |
|  | McMaster 57545K624 Metal Worm Gear |  | 0.001 | 1 | 0.00 | Catalog Lookup |
|  | McMaster 4037N117 Plastic Worm Gear |  | 0.010 | 1 | 0.01 | Catalog Lookup |
|  | Adapter and Thumbwheel |  | 0.001 | 1 | 0.00 | ULTEM-9085 3D printed with 80% infill - CAD Model |
|  | Electronics Assembly | Yes | 0.049 | 1 | 0.05 | Estimate based on number of components (14 grams for board, 25 grams for components |
|  | Electronics Top Cover |  | 0.013 | 1 | 0.01 | ULTEM-9085 3D printed with 80% infill - CAD Model |
|  | McMaster 97763A414-18-8 SS Hex Drive Screws |  | 0.000 | 3 | 0.00 | Catalog Lookup |
| **2.0** | **Turret and Extensions** |  |  |  | **0.1116** |  |
|  | McMaster 5909K110 Thrust Needle Roller Bearing |  | 0.000 | 2 | 0.00 | Catalog Lookup |
|  | Cradle |  | 0.027 | 1 | 0.03 | Folded 2 mm Aluminum 6061-T6 - CAD Model |
|  | McMaster 92552A434 Knurled Hand Knob |  | 0.001 | 1 | 0.00 | Catalog Lookup |
|  | Thrust Washer Capture |  | 0.001 | 1 | 0.00 | ULTEM-9085 3D printed with 80% infill - CAD Model |
|  | Carrier Plate |  | 0.049 | 1 | 0.05 | Folded 2 mm Aluminum 6061-T6 - CAD Model |
|  | Straight Linkage |  | 0.003 | 2 | 0.01 | 2 mm Aluminum 6061-T6 - CAD Model |
|  | Straight Linkage with cutout |  | 0.004 | 2 | 0.01 | 2 mm Aluminum 6061-T6 - CAD Model |
|  | Polulu Gear Motor (Extension) | Yes | 0.010 | 1 | 0.01 | Catalog Lookup |
|  | Polulu Rotation Sensor | Yes | 0.005 | 1 | 0.01 | Catalog Lookup |
|  | Polulu Horn |  | 0.000 | 1 | 0.00 | ULTEM-9085 3D printed with 80% infill - CAD Model |
|  | Carrier Polulu Holder |  | 0.001 | 1 | 0.00 | ULTEM-9085 3D printed with 80% infill - CAD Model |
|  | Carrier Pin |  | 0.000 | 1 | 0.00 | ULTEM-9085 3D printed with 80% infill - CAD Model |
|  | 4mm washer |  | 0.000 | 12 | 0.00 | 2 mm Aluminum 6061-T6 - CAD Model |
|  | McMaster 95648A703 Captive Pin |  | 0.000 | 8 | 0.00 | Catalog Lookup |
|  | McMaster 97763A414-18-8 SS Hex Drive Screws |  | 0.000 | 2 | 0.00 | Catalog Lookup |
| **3.0** | **Forearm** |  |  |  | **0.204** |  |
|  | Dynamixel XM430 | Yes | 0.082 | 2 | 0.16 | Catalog Lookup |
|  | Arm Extension |  | 0.035 | 1 | 0.04 | ULTEM-9085 3D printed with 80% infill - CAD Model |
|  | Arm Cover |  | 0.001 | 1 | 0.00 | ULTEM-9085 3D printed with 80% infill - CAD Model |
|  | Gripper Adapter |  | 0.002 | 1 | 0.00 | ULTEM-9085 3D printed with 80% infill - CAD Model |
|  | Servo Horn |  | 0.000 | 2 | 0.00 | ULTEM-9085 3D printed with 80% infill - CAD Model |
|  | McMaster 92095A115-18-8 SS Hex Drive Screws |  | 0.000 | 2 | 0.00 | Catalog Lookup |
|  | McMaster 97763A414-18-8 SS Hex Drive Screws |  | 0.000 | 16 | 0.00 | Catalog Lookup |
| **4.0** | **Gripper and Wrist** |  |  |  | **0.0291** |  |
|  | Gripper Frame |  | 0.009 | 1 | 0.01 | ULTEM-9085 3D printed with 80% infill - CAD Model |
|  | Cover |  | 0.000 | 1 | 0.00 | ULTEM-9085 3D printed with 80% infill - CAD Model |
|  | Finger Middle Link |  | 0.003 | 2 | 0.01 | ULTEM-9085 3D printed with 80% infill - CAD Model |
|  | Finger-Tip Outside |  | 0.001 | 1 | 0.00 | ULTEM-9085 3D printed with 80% infill - CAD Model |
|  | Finger-Tip Inside |  | 0.001 | 1 | 0.00 | ULTEM-9085 3D printed with 80% infill - CAD Model |
|  | SG-90 Servo | Yes | 0.005 | 1 | 0.01 | Catalog Lookup |
|  | Pulley |  | 0.000 | 1 | 0.00 | ULTEM-9085 3D printed with 80% infill - CAD Model |
|  | Large Pad |  | 0.000 | 2 | 0.00 | Custom Rubber Pad - CAD Model |
|  | Small Pad |  | 0.000 | 2 | 0.00 | Custom Rubber Pad - CAD Model |
|  | Small Torsional Spring |  | 0.001 | 4 | 0.00 | Aluminum 6061-T6 Custom Design - CAD Model |
|  | McMaster 95648A703 Captive Pin |  | 0.000 | 2 | 0.00 | Catalog Lookup |
|  | McMaster 95648A704 Captive Pin |  | 0.000 | 2 | 0.00 | Catalog Lookup |
|  | McMaster 97763A414-18-8 SS Hex Drive Screws |  | 0.000 | 8 | 0.00 | Catalog Lookup |
|  |  |  |  | **Total** | **0.47** | kg |

# System Layout

## Electronics System


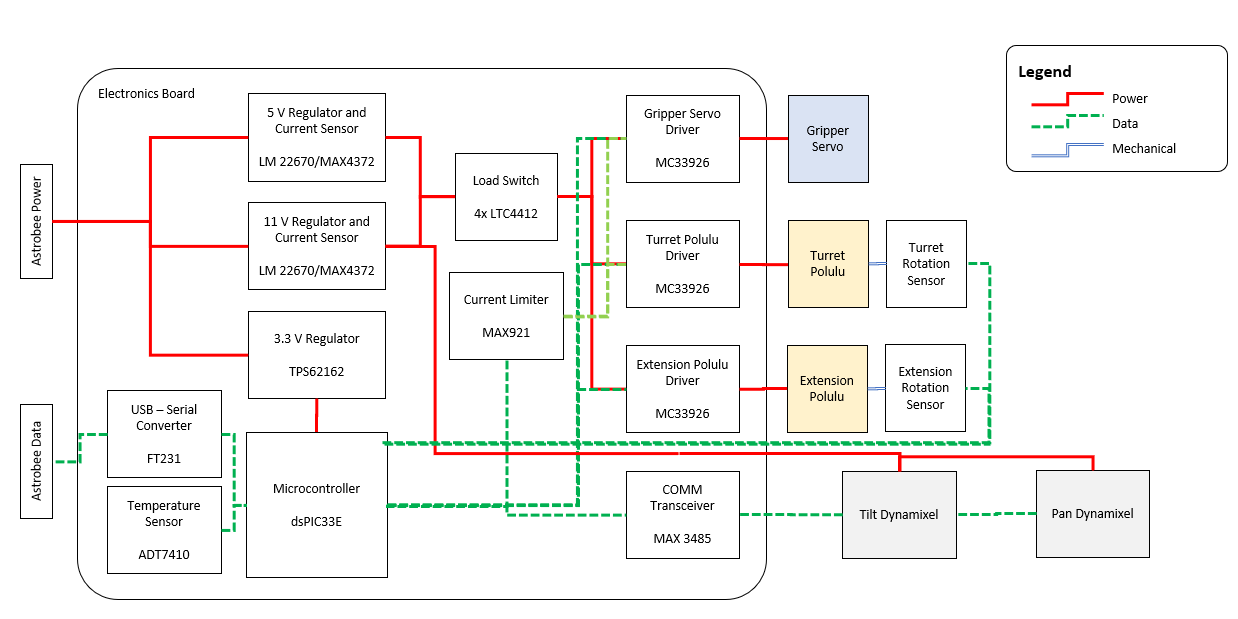


## Mechanical Design


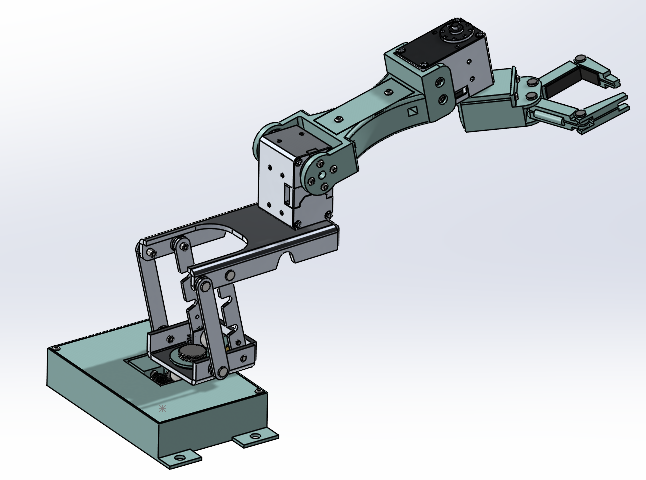


## Mechanical Subsystems

| **Base** | **Turret** |
| --- | --- |
| 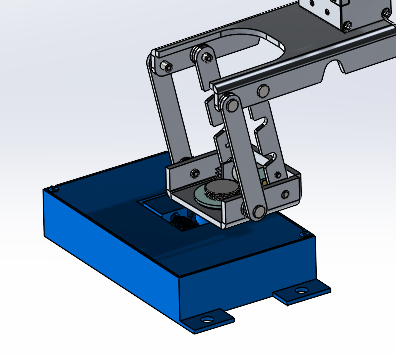  Responsible for electronics control. Also rotates the base of the robot side to side and helps secure it to the Astrobee Platform. | 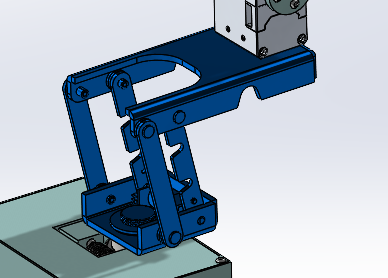  Responsible for parallel extension of the robotic arm forearm and gripper. Designed to help the system fold up into a compact form |
| **Forearm** | **Gripper** |
| 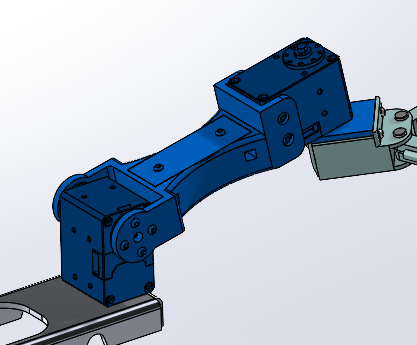  Responsible for the pan and tilt motion of the robotic system. Attaches on one end to the turret and has an adapter on the other end, which allows for connection to the gripper. | 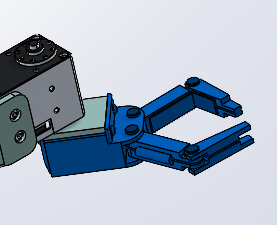  Responsible for capturing the handrail. Designed with internal springs to help keep it naturally closed. A small motor helps keep it open for grasping and releasing and passively holds onto the ISS Handrail. |

## Base Exploded View


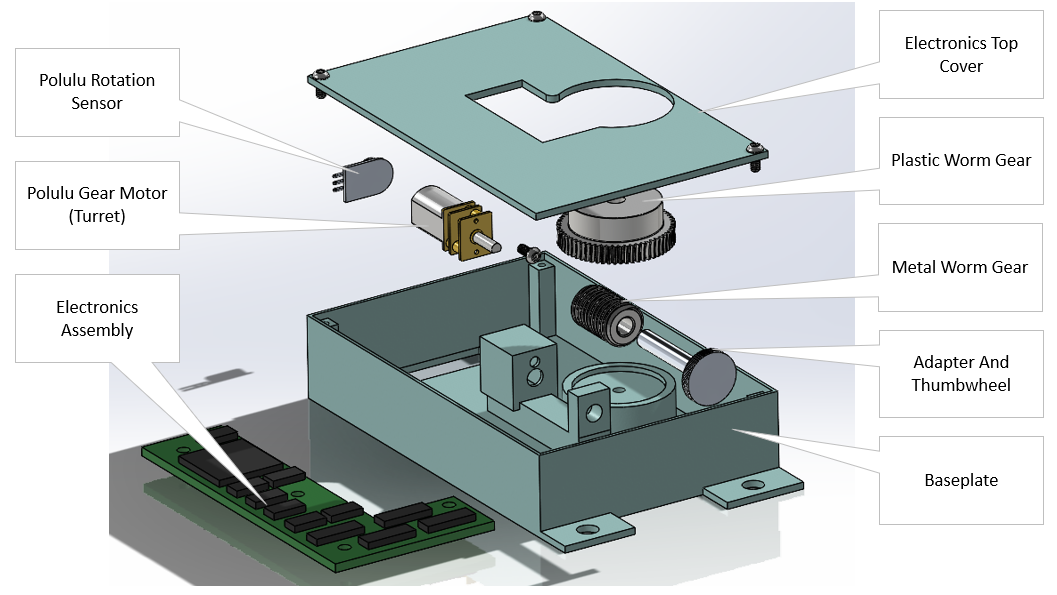


## Turret Exploded View


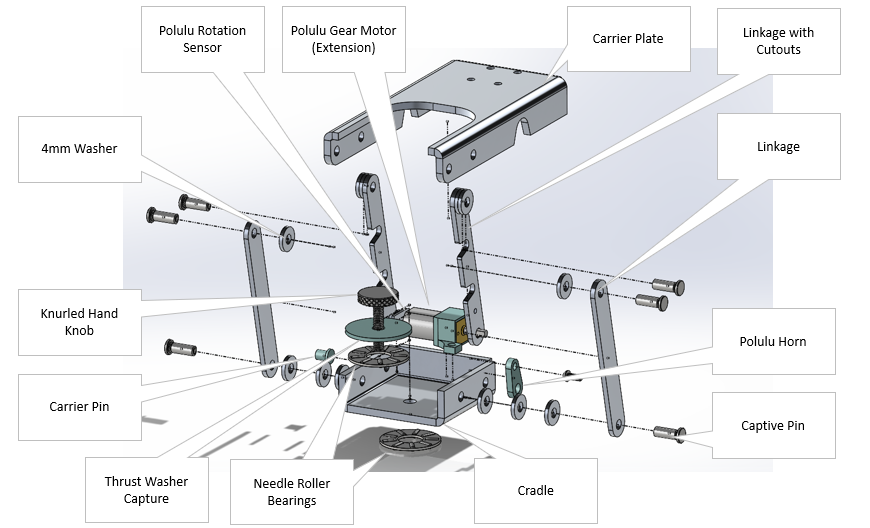


## Forearm Exploded View


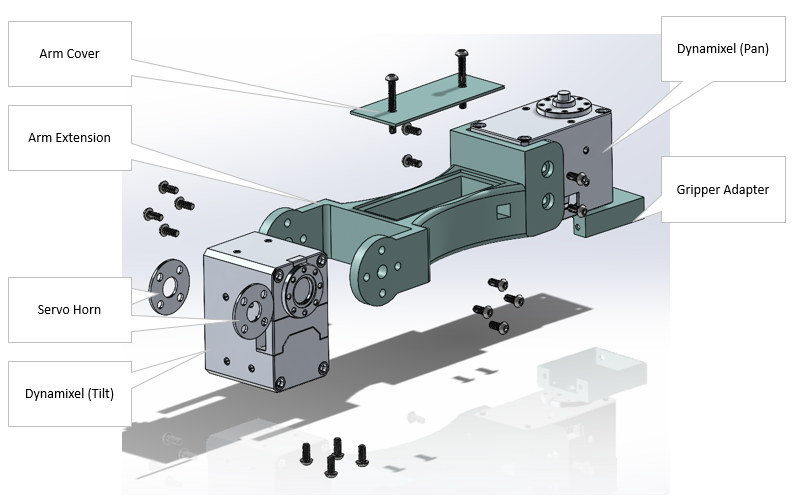


## Gripper Exploded View


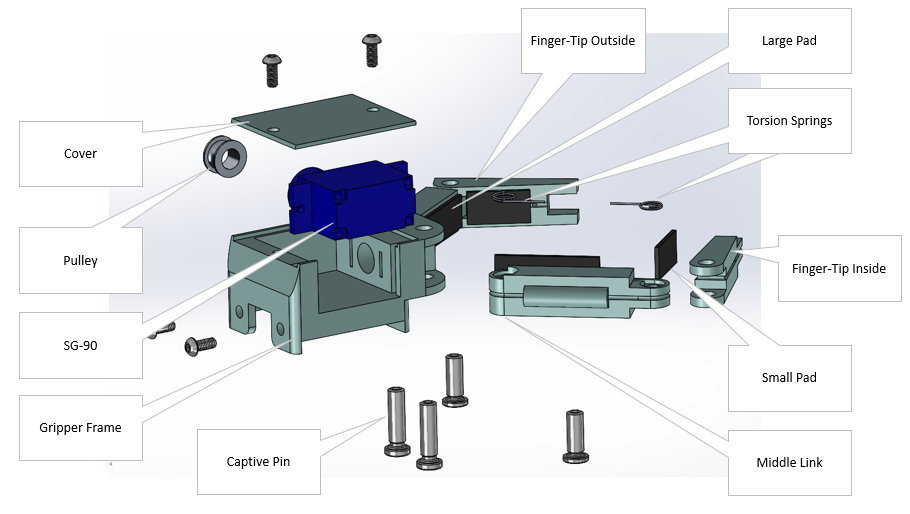


## Turret Base Detailed View


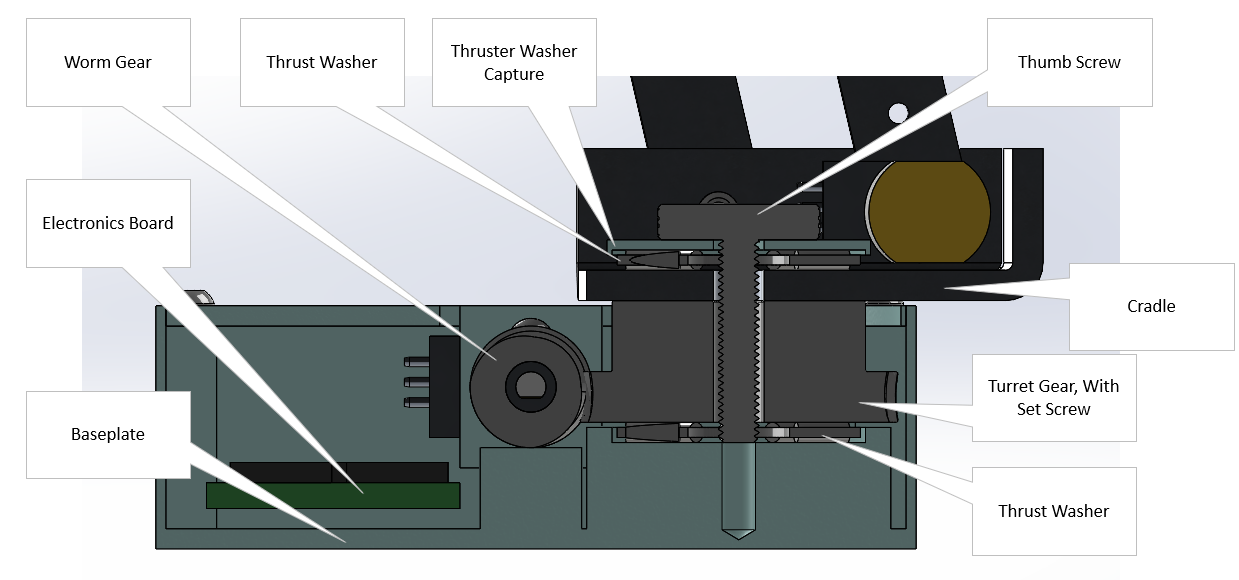


## Gripper Detailed View


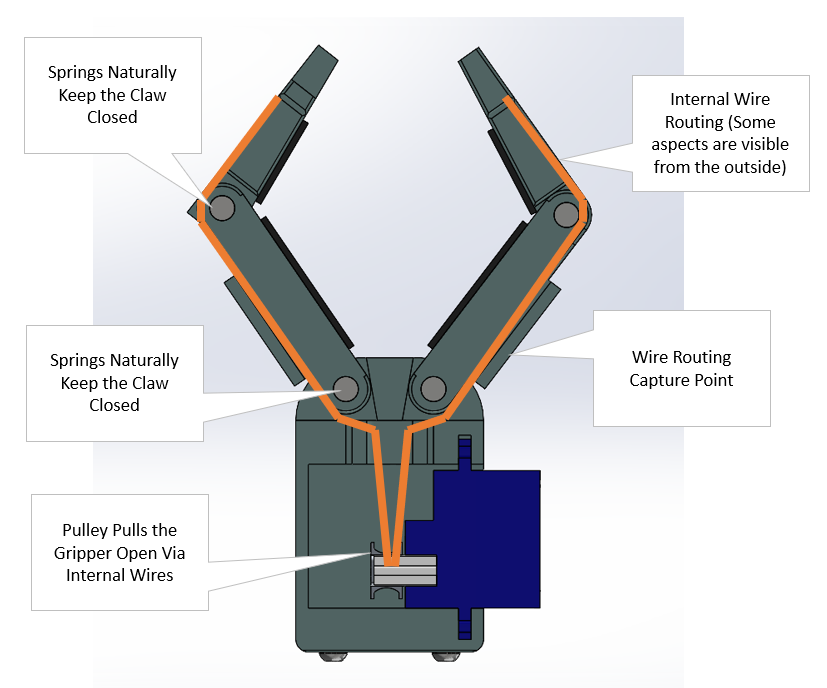


## Wire Routing View

##
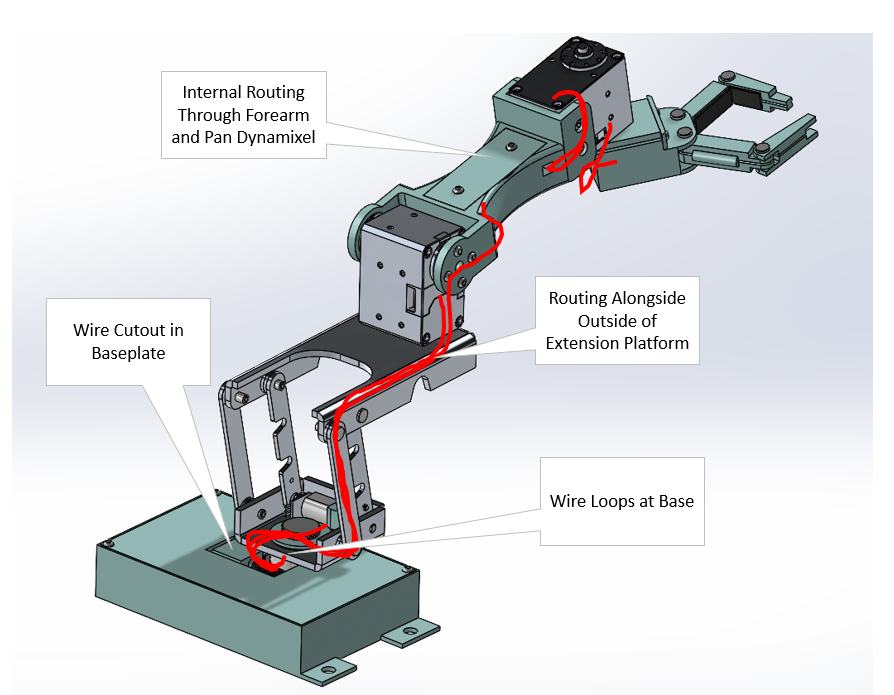


## Extreme Conditions

## We found that because of the motion of our gripper, we can deal with a handrail of any height. However, because there could be a strike between the gripper and upper part of the Handrail, the worse possible condition involves the gripper centrum on the bottom plane of the grasping envelope. In the below pictures, we show that the gripper can work through the entire range of this motion.

|  | **Front Extreme** | **Back Extreme** |
| --- | --- | --- |
| **Left Extreme** | **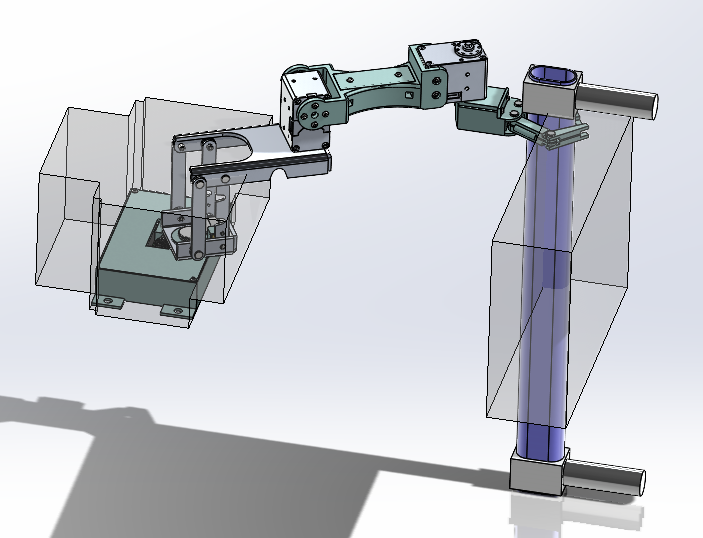** | **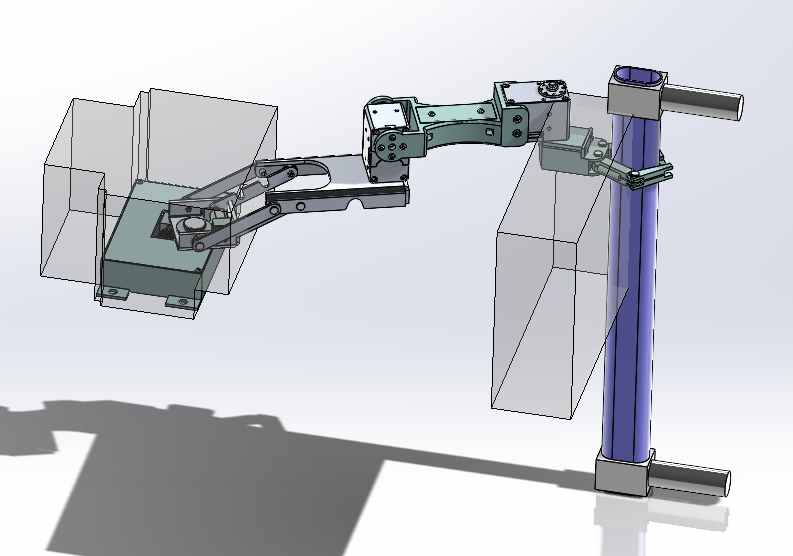** |
| **Middle Extreme** | **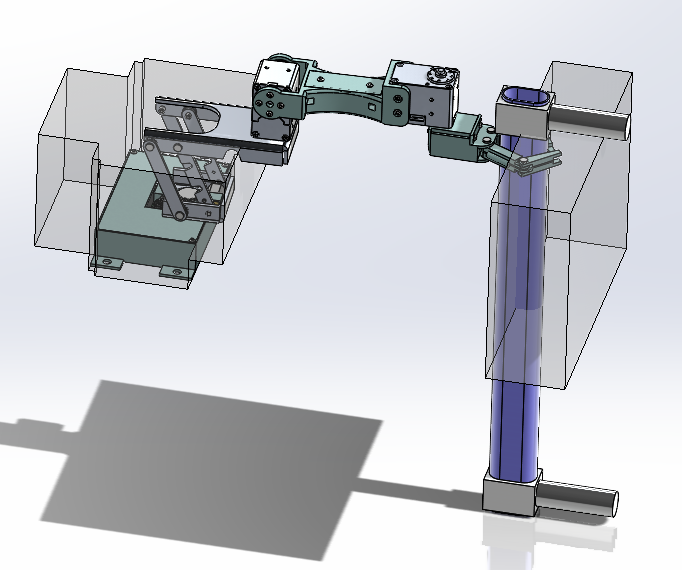** | **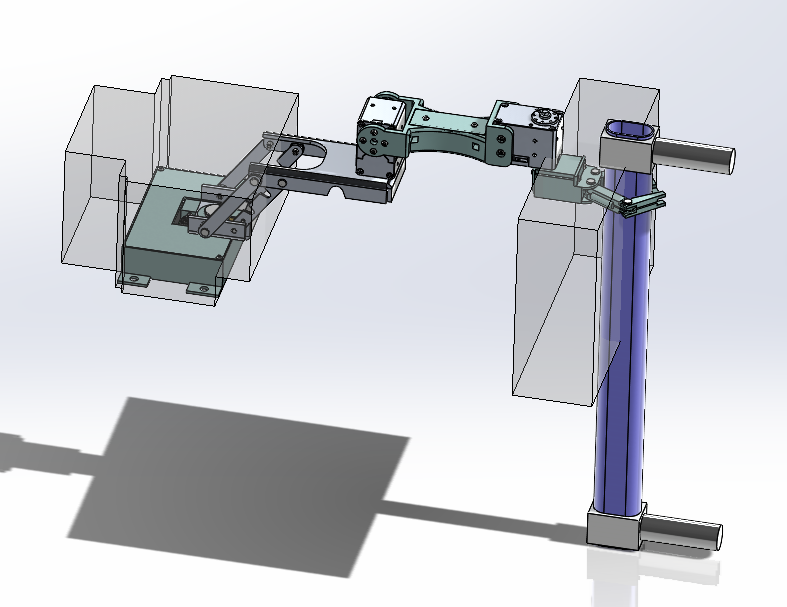** |
| **Right Extreme** | **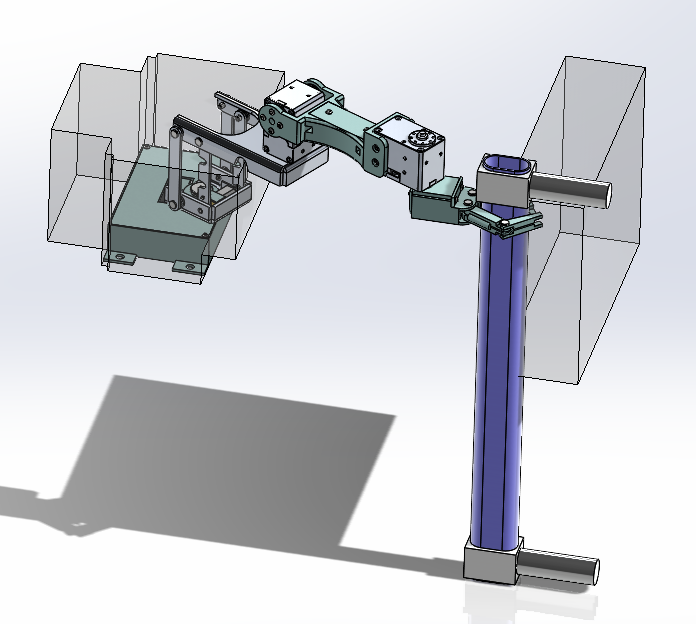** | **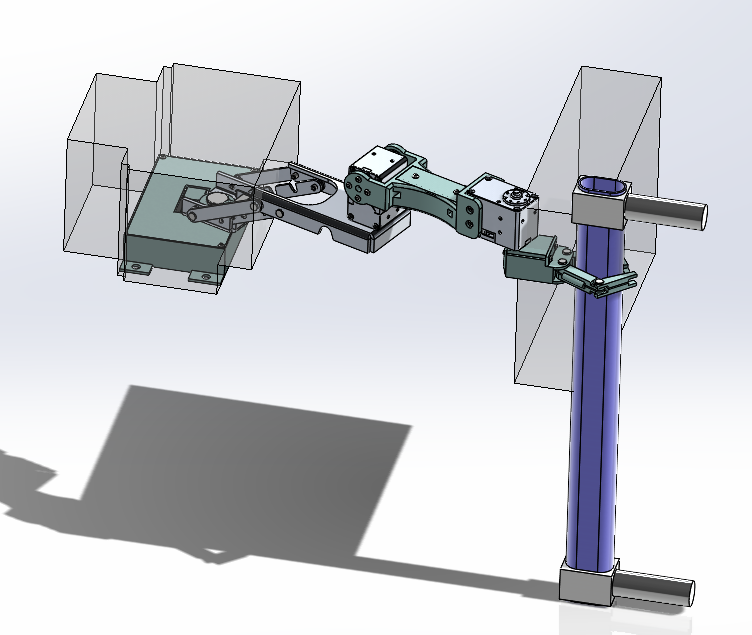** |

# Design Drawings

## Attached to an ISS Handrail at (300 mm, 50 mm, -95 mm)


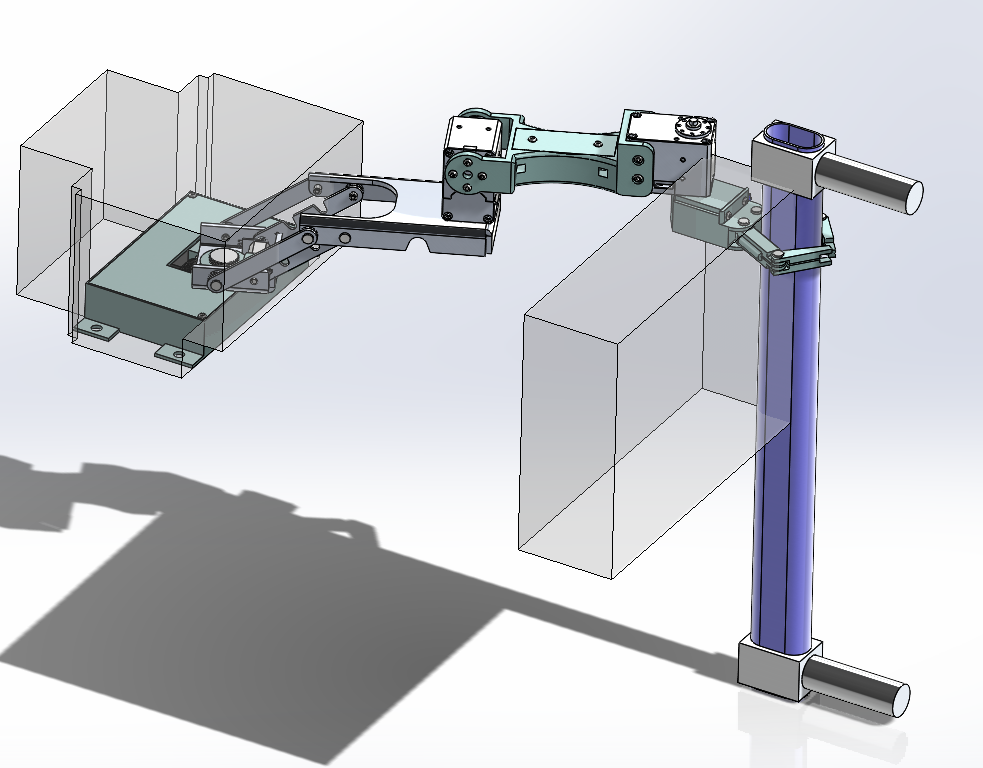


## Attached to a Handrail at (300, 50, -95), with Astrobee oriented with Tilt angle = 30° and Pan angle = 45°.


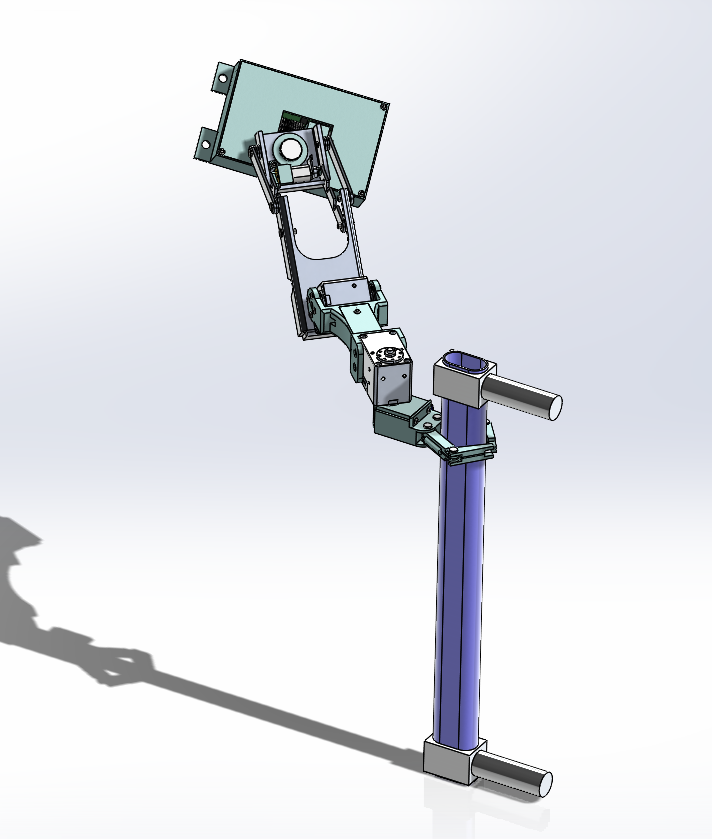

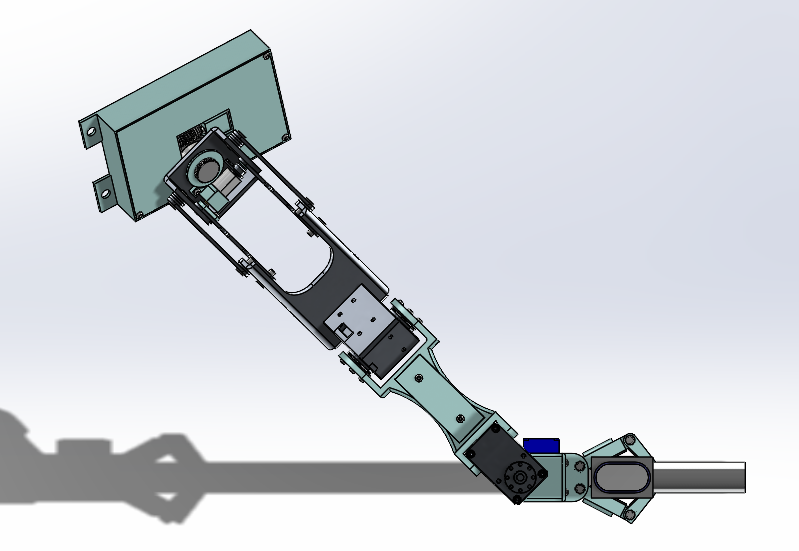


## Stowed in the Astrobee Payload Bay. Indicate closest distance to any Payload Bay wall in this stowed configuration

| **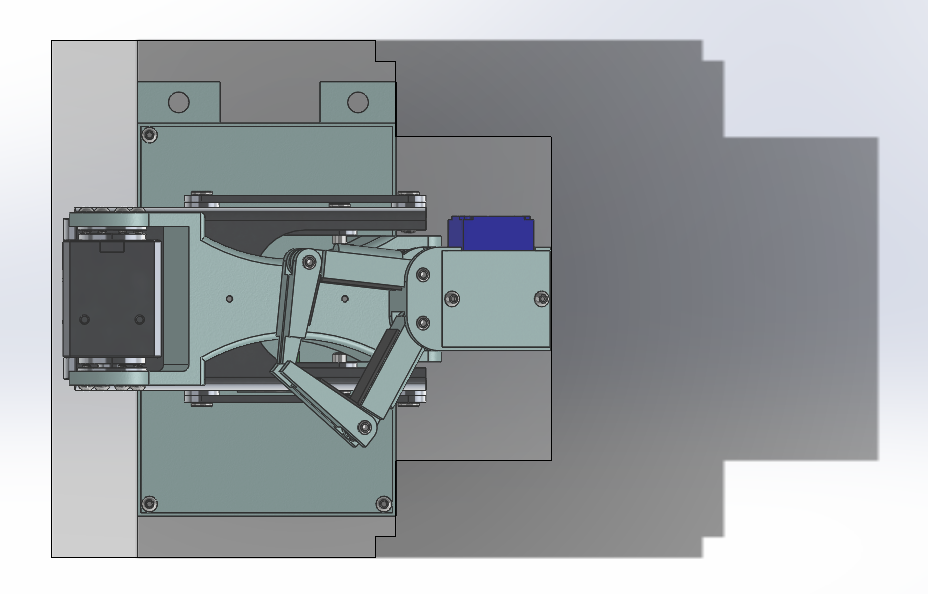**  **Top** | |
| --- | --- |
| 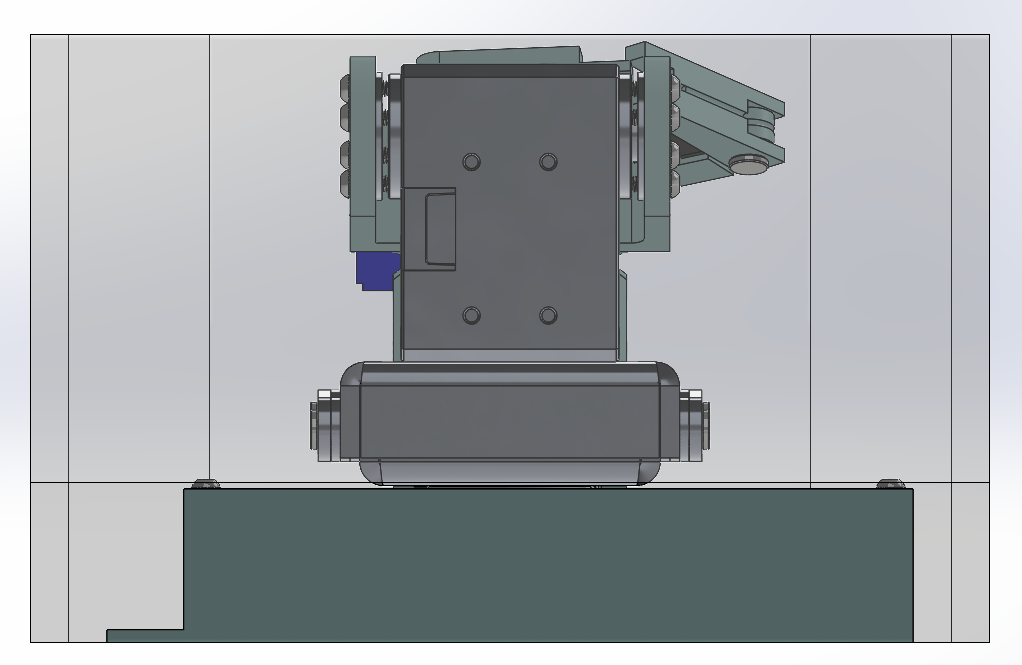  **Front** | **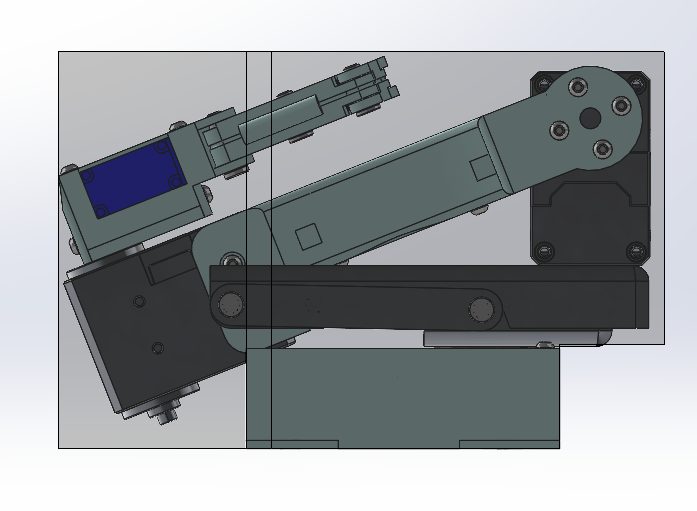**  **Side** |

The closest is a 0mm intersection, indicated with the red circle.

# Software Description

We use ROS extensively to do the specific path planning for the robotic arm.


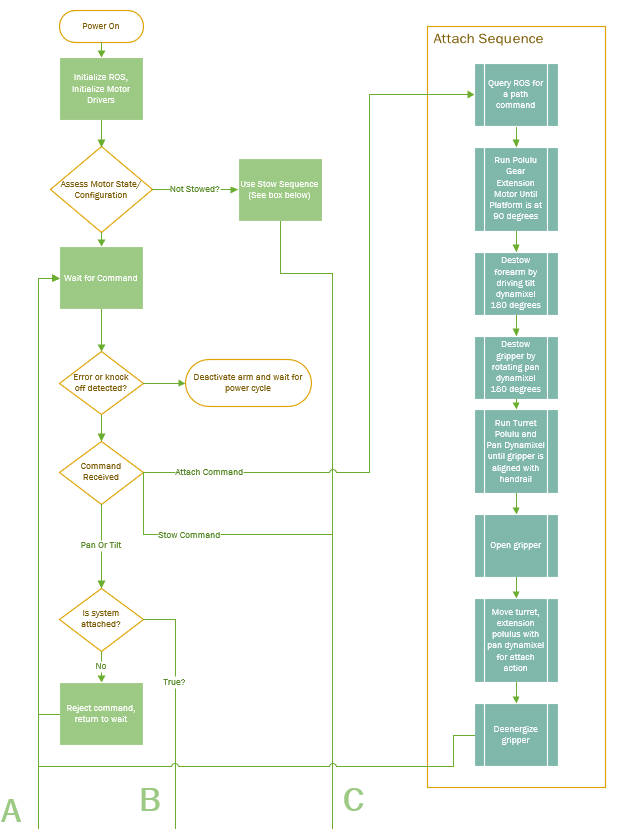


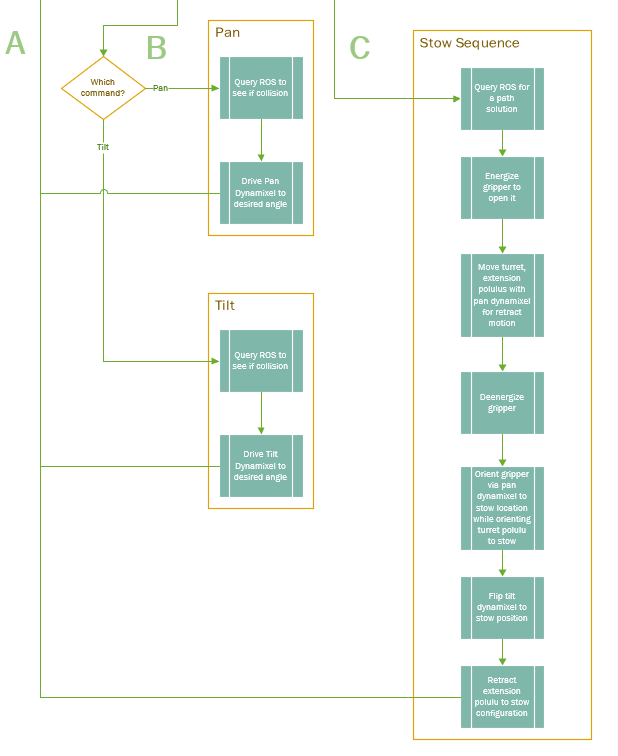


# Power Usage Description

## Mode Descriptions

|  | **Power Mode Description** |  | | |  | |  | |  | |  |  |
| --- | --- | --- | --- | --- | --- | --- | --- | --- | --- | --- | --- | --- |
| **Mode 1** | ROS Query |  | |  | |  | |  | |  |  |  |
| **Mode 2** | Extension Drive |  |  |  |  |  |  |  |  |  |  |  |
| **Mode 3** | Dynamixel Move (pan, tilt movement) |  |  |  |  |  |  |  |  |  |  |  |
| **Mode 4** | Turret Move + Dynamixel |  |  |  |  |  |  |  |  |  |  |  |
| **Mode 5** | Extension + Turret + Dynamixel (For planar grasping movement) + Gripper Energize |  |  |  |  |  |  |  |  |  |  |  |
| **Mode 6** | Energize Gripper |  |  |  |  |  |  |  |  |  |  |  |

## Modes

|  |  |  | ***Mode 1*** | ***Mode 2*** | ***Mode 3*** | ***Mode 4*** | ***Mode 5*** | ***Mode 6*** |
| --- | --- | --- | --- | --- | --- | --- | --- | --- |
|  |  |  | Current (A) | Current (A) | Current (A) | Current (A) | Current (A) | Current (A) |
| **1.0** | **Base Module** |  |  |  |  |  |  |  |
|  | Polulu Gear Rotation |  |  |  |  | 0.2 | 0.2 |  |
|  | Polulu Rotation Sensor |  | 0.1 | 0.1 | 0 | 0.1 | 0.1 | 0.1 |
|  | Electronics Assembly |  | 0.4 | 0.2 | 0 | 0.2 | 0.2 | 0.2 |
| **2.0** | **Turret and Extension** |  |  |  |  |  |  |  |
|  | Polulu Gear Extension |  |  | 0.2 |  |  | 0.2 |  |
|  | Polulu Rotation Sensor |  | 0.1 | 0.1 | 0 | 0.1 | 0.1 | 0.1 |
| **3.0** | **Base Module** |  |  |  |  |  |  |  |
|  | Dynamixel Tilt |  | 0.4 | 0.4 | 0.5 | 0.5 | 0.5 | 0.4 |
|  | Dynamixel Pan |  | 0.4 | 0.4 | 0.5 | 0.4 | 0.5 | 0.4 |
| **4.0** | **Gripper Module** |  |  |  |  |  |  |  |
|  | SG-90 Servo |  |  |  |  |  | 0.1 | 0.1 |
|  | Total steady-state current (A): | | 1.4 | 1.4 | 1 | 1.5 | 1.9 | 1.3 |

## Power Profiles

|  |  |  | |  | **Which Power Mode** | **Time On (s)** | **Energy (W)** | | |  | | | |  |  |
| --- | --- | --- | --- | --- | --- | --- | --- | --- | --- | --- | --- | --- | --- | --- | --- |
| **(1) Attach to Handrail** | | | |  |  |  |  | | |  | | |  |  |  |
|  | ROS and Startup | | |  | Mode 1 | 30 | 0.16 | | |  | |  |  |  |  |
|  | Extension Drive | | |  | Mode 2 | 15 | 0.08 | | |  | |  |  |  |  |
|  | Dynamixel Move (Tilt, then Pan) | | |  | Mode 3 | 15 | 0.06 | | |  | |  |  |  |  |
|  | Energize Gripper | | |  | Mode 6 | 10 | 0.05 | | |  | |  |  |  |  |
|  | Align turret | | |  | Mode 4 | 30 | 0.18 | | |  | |  |  |  |  |
|  | Extend and Close | | |  | Mode 5 | 30 | 0.22 | | |  | |  |  |  |  |
|  |  |  | |  | *Time to complete (min)* | 2 |  | | |  | | | |  |  |
|  |  |  | |  |  |  |  | | |  | | | |  |  |
| **(2) Remain Attached (60 min)** | | | |  |  |  |  | | |  | | |  |  |  |
|  | Wait for next command (using a low power mode) | | | | Mode 3 in ultra-low power mode 10 | 3600 | 1.40 | | |  | | |  |  |  |
|  |  |  | |  |  |  |  | | |  | | | |  |  |
| **(3) Retract to Stowed configuration** | | | |  |  |  |  | | |  | | |  |  |  |
|  | ROS and Startup | | |  | Mode 1 | 30 | 0.16 | | |  | |  |  |  |  |
|  | Open and Retract | | |  | Mode 5 | 30 | 0.22 | | |  | |  |  |  |  |
|  | Align base | | |  | Mode 4 | 10 | 0.06 | | |  | |  |  |  |  |
|  | Dynamixel Move | | |  | Mode 3 | 15 | 0.06 | | |  | |  |  |  |  |
|  | Retract back | | |  | Mode 2 | 1 | 0.01 | | |  | |  |  |  |  |
|  |  |  | |  | *Time to complete (min)* | 1 |  | | |  | | | |  |  |
|  |  |  | |  |  |  | | | | | | | |  |  |
|  |  |  | **Total Energy for Operational Sequence (W-hr)** | | | | | 2.66 |  | |  |  |  |  |  |

# Exit Survey

Exit Survey for Freelancer <<NG_Roman>> complete per completion code: <<3251993>>
