# Supplementary material for: Linking solver characteristics, solving processes and solution attributes: A data explainer for an open innovation generated robotic design dataset
Source: Data Brief. 2023 Sep 6;50:109547. doi: 10.1016/j.dib.2023.109547 (PMC10518673; doi:10.1016/j.dib.2023.109547)
Supplement: Supplementary file 1 [file mmc1.zip › Release/Solvers/Survey Questions/Registration Survey.pdf]

Tools Saved at 3:53 PM Live Preview Publish

Registration Survey

ExpertReview score Fair

Survey Overview

I01

This survey must be completed before you can participate in any of the NASA Astrobee Challenges. It should take 15-20 minutes. Once completed you will be redirected to the Astrobee page where you will be able to see the detailed descriptions of all available contests.

NASA is collecting this information to better understand who in the Freelancer community is solving problems. Anonymous data collected here will be shared with researchers and data scientists.

After a few background questions, you will be asked to make a large number of comparisons among robotics-related objects.

Page Break

R01 *

Please enter your Freelancer username (the raffle/drawing will be based on your username so please check to make sure it's entered correctly):

R01 *

In a few sentences, please tell us why you are interested in registering for this contest?

R03 *

Please indicate your gender:

- Male
- Female
- Prefer not to say

R04

How old are you?

- Under 18
- 18 - 24
- 25 - 34
- 35 - 44
- 45 - 54
- 55 - 64
- 65 or older
- Prefer not to say

R05 | List of Countries

In which country do you currently reside?

Afghanistan

Page Break

R06

Educational Background. Please fill in the below table indicating all of your degrees and/or certifications.

	Have you completed this degree/certification?		List subject area (e.g., Chemistry)
	Yes	No	No acronyms please
High School	○	●	N/A
Associates	○	●	N/A
Bachelors	○	●	N/A
Masters	○	●	N/A
Professional (MBA, JD, MD)	○	●	N/A
Doctorate	○	●	N/A
Other Certification	○	●	N/A

Page Break

R07

In what disciplines/fields have you previously worked? Please select all that apply.

- ☐ Architecture or Design
- ☐ Art
- ☐ Business and Finance
- ☐ Education
- ☐ Engineering or Science
- ☐ Aerospace and Defense
- ☐ IT Services
- ☐ Law
- ☐ Maintenance and Repair
- ☐ Medicine
- ☐ Production or Manufacturing
- ☐ Other, please specify: ____

Page Break

R08

If you have ever worked in a technical organization, for each area of specialization, indicate your number of years of experience.

	Have you worked or volunteered in this field?		If yes, for how many years?
	Yes	No	Number of Years
Electrical Engineering	○	◉	0
Engineering Drawing	○	◉	0
Design	○	◉	0
Industrial Engineering	○	◉	0
Manufacturing	○	◉	0
Material Science or Engineering	○	◉	0
Mechanical Engineering	○	◉	0
Project Management	○	◉	0
Robotics or Mechatronics	○	◉	0
Software or Computer Engineering or Computer Science	○	◉	0
Other ____	○	◉	0

Page Break

R09

Have you ever participated in any sports or serious hobbies? If so, please list them:

____

R10

A space robotics design problem is:

Inside my field of expertise	......	.....	At the boundary of my field of Experitise	.....	.....	Outside my field of expertise
○	○	○	○	○	○	○

Page Break

Q15

In the next section of this survey you will be asked to rate the similarity of objects like those shown below. There are 11 objects total and you will be asked to compare each one to every other one. This may feel repetitive (there are 55 comparison!), but your responses will tell NASA a lot about the perspective you bring to this contest. This is the last step before you're registered. We appreciate your patience!

I02

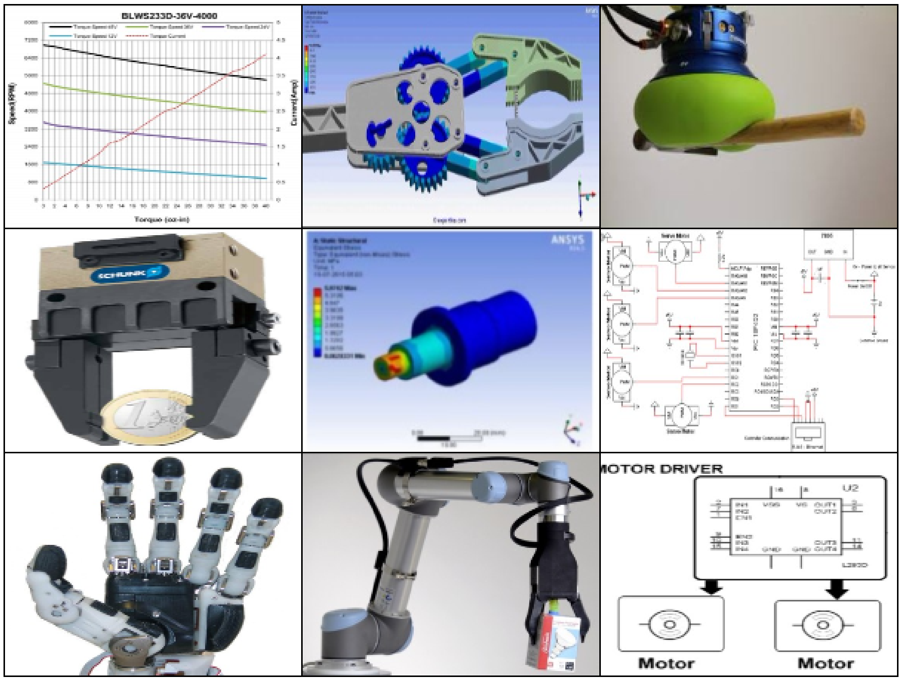

Import from library

Add new question

Add Block

Object Comparisons - Astrobee01

T01

This question lets you record and manage how long a participant spends on this page. This question will not be displayed to the participant.

Q26

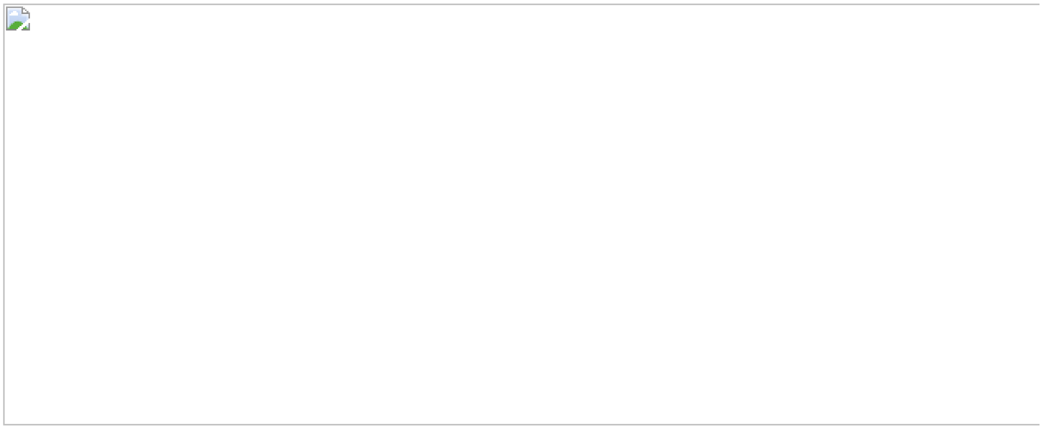

On a scale of *0* to *100*, how **similar** are these?

0 25 50 75 100

Page Break

Import from library Add new question

Add Block

End of Survey

We thank you for your time spent taking this survey.

Your response has been recorded.
