# Supplementary material for: Linking solver characteristics, solving processes and solution attributes: A data explainer for an open innovation generated robotic design dataset
Source: Data Brief. 2023 Sep 6;50:109547. doi: 10.1016/j.dib.2023.109547 (PMC10518673; doi:10.1016/j.dib.2023.109547)
Supplement: Supplementary file 1 [file mmc1.zip › Release/Solvers/Survey Questions/Exit Survey.pdf]

Tools Saved Sep 8, 2022 at 3:23 PM Live

Preview Publish

Exit Survey

ExpertReview score Fair

This Exit Survey is a required part of your submission. At the end of the survey

Q1

This Exit Survey is a required part of your submission. At the end of the survey you will receive a completion code. Please include it in your submission files.

Page Break

Q2

Please enter your Freelancer username:

Q3

In a few sentences, please tell us why you chose to participate in this contest:

Q4

How similar was this contest to projects/problems you normally work on?

Identical	Very similar	Similar	Different	Very different
○	○	○	○	○

Q5

Please describe the most similar project you've worked on in the past.

Q6

Skip to

In completing your solution, did you ... if No Is Selected

Did you acquire any new equipment or learn any new skills to participate in this contest?

- Yes
- No

Q7

Please describe the new equipment or skills.

Q8

Skip destination Go to skip origin

In completing your solution, did you draw on any skills or knowledge that you don't typically use in your professional life? If yes, please tell us what they were.

Q9

Compared to what you expected, how difficult was this contest?

Much easier	.	.	As expected	.	.	Much more difficult
◯	◯	◯	◯	◯	◯	◯

Q15

How long did you spend solving this problem? (Estimate time in hours)

Q10

Please choose the level that best fills in the blank. I am ____________ with similar problems

Not experienced at all	.	.	Moderately experienced	.	.	Very experienced
○	○	○	○	○	○	○

Page Break

Q11

This is your survey completion code: ${e://Field/Completion%20code}
Please be sure to include it in your submission document.

Import from library Add new question

Add Block

End of Survey

We thank you for your time spent taking this survey.

Your response has been recorded.
