# Supplementary material for: Linking solver characteristics, solving processes and solution attributes: A data explainer for an open innovation generated robotic design dataset
Source: Data Brief. 2023 Sep 6;50:109547. doi: 10.1016/j.dib.2023.109547 (PMC10518673; doi:10.1016/j.dib.2023.109547)
Supplement: Supplementary file 1 [file mmc1.zip › Release/Process/Challenge Rules/D5-MDC/MDC Submission Guidelines.pdf]

Submission Guidelines for the Mechanically Driven Clamp

In this contest, you were asked to design a Mechanically Driven Clamp (MDC) that will be mounted to the free end of a separately designed robotic arm.

This document provides detailed guidelines on how you must describe and present each aspect of your design in order to be considered for the prize. This document looks long but very little text is required. It's mostly pasting in figures and tables in a structured way. Your submission document must include each of the sections detailed below and all of the information requested in each. Several templates and examples are provided to clarify what constitutes a complete solution.

Use the exact section and subsection header words, shown below.

1 FUNCTIONAL DESCRIPTION 2

1.1 NARRATIVE (WORD) DESCRIPTION OF DESIGN 2
1.2 FUNCTIONAL ANALYSIS 2

2 MASS SUMMARY AND COMPONENT LIST 3

3 SYSTEM LAYOUT 4

4 DESIGN DESCRIPTION 5

4.1 DESIGN DRAWINGS 5
4.2 OPERATING MODES 5

5 EXIT SURVEY 7

1 Functional Description

1.1 Narrative (word) description of design

In this section, describe how your design for the Mechanically Driven Clamp (MDC) works. In a few sentences, please describe how your solution does each of the following:

1) Close: How does your MDC attach to the Handrail?
2) Hold: How does your MDC stay fixed to the Handrail, in the *attached* configuration?
3) Release: How does your MDC return to the *pre-attached* configuration?
4) Astronaut Pull Away: How does your MDC release from the handrail when the astronaut pull load is applied to the interface plate?

Although it is not required, you may embed images with sketches, models, storyboards or other illustrations in your written descriptions to help explain how your MDC design accomplishes these actions.

Minimum content requirement: Text response to each of the above questions.

1.2 Functional Analysis

In this section, describe your logic and/or analysis for the following aspects of your MDC design. Including equations and mathematics is acceptable if it helps clarify the logic behind your design, but please ensure that it will be understood by our reviewers by annotating your process or describing the math being done and why.

(1) What is the typical force you expect your MDC to exert on the Handrail while in the *Attached* configuration (R2)?
(2) How does your MDC design close on a Handrail when the Handrails has its maximum offset (per R1.2)?
(3) In your EDC design, how close do you get to the boundary of the *pre-attached* volume (R1.1)?
(4) How did you confirm that your MDC will not exceed the mechanical power limit (C3)?

Minimum content requirement: Text responding to each of the above questions.

2 Mass Summary and Component List

In this section, list all the elements of your MDC solution using the template provided [MDCMassTemplate available as .odt, .xlsx]. For each component/piece/part, include an estimate of its mass and a brief explanation of where the estimate came from. Please be sure to include the reasons supporting your mass estimate for each element since they will be part of the evaluation of the credibility of your MDC mass estimate.
Table 1 provides an example of how the template should be filled in.

MDC Components		Is this a powered component?	Estimated Mass per Unit (kg)	Quantity (# units)	Total Mass (kg)	Basis of estimate
**1.0**	**Subsystem #1**					
	Mechanism #1	Yes	0.100	1.0	0.10	Typical mass of component that I use all the time in design of systemX.
	Sensor #1-a	Yes	0.010	1.0	0.01	Called some former coworker who builds these, and asked for a typical masses
	Mechanism #2	No	0.080	1.0	0.08	Made a CAD model, assumed SS316, to obtain this mass
	Sensor #1-b	Yes	0.001	1.0	0.00	Weighed a prototype I built
				Total Mass	0.53	kg

Table 1 Mass Summary and Component list - example

Minimum content requirement: Paste your filled table into this section of the document. No additional text is required.

3 System Layout

In this section, provide a diagram(s) identifying all the physical components/piece/part of your MDC design and how components connect to, and/or move each other.

Please use the names of components from the Mass Summary and Component List described in section 2.

An electro-mechanical schematic similar that gives an “exploded view” is the preferred format. See Figure 1 for an example. The defining feature of an exploded view is that it conveys information about how all the mechanical and electro-mechanical (actuators and sensors) pieces connect to one another.

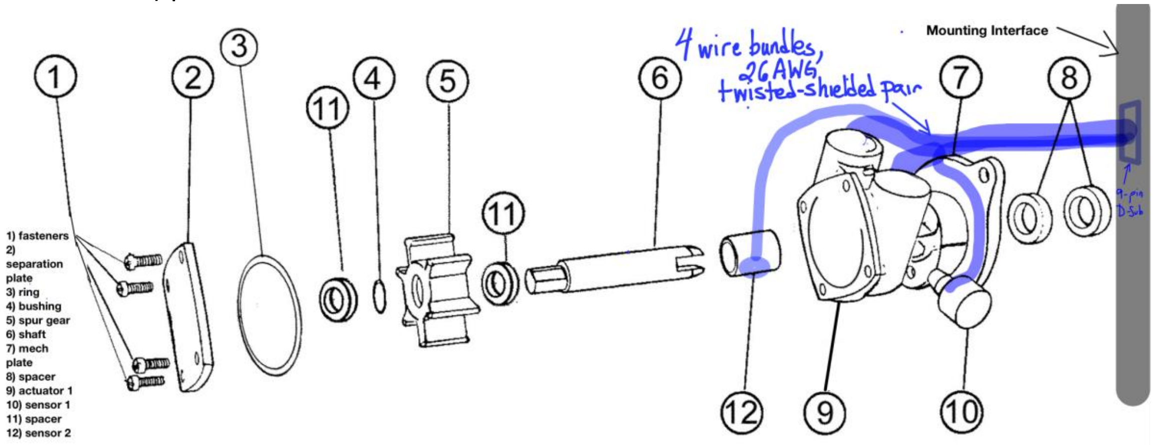

Figure 1 Electro-mechanical layout - example

Minimum content requirement: Your submission must include either an electro-mechanical schematic, like Figure 1, or a similar diagram that shows the same kind of information. A legend defining any symbols or colors used in the diagrams is also required.

4 Design Description

4.1 Design Drawings

In this section, include engineering-style drawings showing your solution's physical configuration for each of the following.

1) *Pre-attached configuration* (R1.1)
2) *Attached configuration* where your MDC is fixed to an ISS Handrail (R1.3)

You can use any CAD software you like, sketch them by hand, or photograph a prototype; but accurate dimensions of the whole system are required. At minimum, you must indicate the position of every electro-mechanical component (e.g., actuators and sensors, but not individual bolts). On each figure, please label as many elements and subassemblies as possible. Use the names specified in your Mass Summary and Component List (section 2).

Minimum content requirement: Two design drawings– one for each configuration listed above. Figures must be clearly labeled and dimensioned.

4.2 Operating Modes

In this section, briefly explain how you expect your design to be used for each operation. Specifically, define what 'ending the operation' means in the context of your MDC design for:

1) <u>Close</u> (ends in *attached* configuration)
2) <u>Release</u> (ends in *pre-attached* configuration)

Please describe how to correctly move the drive shaft you have chosen to perform both of these operations. There should also be enough detail for the robotic arm to "know" when to stop moving the drive shaft. Here are two examples of the kind of information that should be conveyed:

- Operating modes based on sensor output: If you have are using a sensor (from the list of permissible sensors) to corresponds to "ending" the <u>close</u> operation, indicate which sensor type and what threshold.
- Operating modes based on drive shaft torque: If you intend the drive shaft's torque to be used to define when a mechanism is closed, specify what torque threshold corresponds to ending <u>close</u> (i.e., being *attached*).
- Operating modes based on timing: if your mechanism will always reach an "ending" position when moved at a particular speed for a fixed time interval, specify how long the shaft should be powered to complete the operation.

The above are examples of the kind of information to be included in your submission. Table 2 provides a template for how you should communicate this information. The first row fills in one example, as illustration. Please use the same names as in the Component List in describing your operation modes.

Operation	Components Moving	Active Components Involved	Description of Operation	Termination Condition
**1) Close**<br>***Example***	*Component A*<br>*Component B*	*Linear drive*<br>*Sensor #1*	*Linear drive shaft moves components A and B until Sensors #1 reaches a particular value.*	*Sensor #1 value = 10 mm*
2) **Release**				

Table 2 – Example Operating Modes Template

Minimum content requirement: One table (like Table 2) with four rows, each defining how your MDC design fulfills the operating modes (close, release and pack).

5 Exit Survey

To complete your submission, please take the Exit Survey by going to this webpage: https://seasgwu.qualtrics.com/jfe/form/SV_2r9DaeSlh48uMcZ

At the end of the survey you will receive a unique code. In your submission include this section and the text: Exit Survey for Freelancer <<insert Freelancer username>> complete per completion code: <<insert completion code>>.

To be complete, your submission must include the following text: Exit Survey for Freelancer <<insert Freelancer username>> complete per completion code: <<insert completion code>>.
