# Supplementary material for: Linking solver characteristics, solving processes and solution attributes: A data explainer for an open innovation generated robotic design dataset
Source: Data Brief. 2023 Sep 6;50:109547. doi: 10.1016/j.dib.2023.109547 (PMC10518673; doi:10.1016/j.dib.2023.109547)
Supplement: Supplementary file 1 [file mmc1.zip › Release/Process/Challenge Rules/D5-MDC/MDC Blurb.docx]

“Mechanically Driven Clamp” (MDC)

In this challenge, you are asked to design a Mechanically Driven Clamp (MDC) that will be mounted on, and driven by, a separately designed robotic arm. The MDC receives mechanical power (either by a rotary shaft or linear actuator) that it must convert into the desired actions.

***How it works*:** Initially, the MDC will be positioned at a Handrail, in a ready-to-clamp orientation. When powered, the MDC must be capable of performing three high-level actions: 1) close on the Handrail, 2) maintain a hold for an extended period of time and 3) release from the handrail.

*Click on the links below to see detailed design instructions, constraints and solution templates for this problem.*

***Challenge rules***: A prize of **$250** will be awarded for the **lowest mass, technically feasible** solution submitted by **August 1^st^ 2018**. No working prototype is required for submission, but the design must be sufficiently detailed to allow experts to assess the feasibility of your design (i.e., comply with all requirements) and the credibility of your mass estimate. Only complete submission packages will be evaluated.

Attachments:

- **MDCProblemDescription.pdf**
- **MDCSubmissionGuidelines.pdf**

Templates

- MDCMassTemplate [.xlsx, odt]
