# Supplementary material for: Linking solver characteristics, solving processes and solution attributes: A data explainer for an open innovation generated robotic design dataset
Source: Data Brief. 2023 Sep 6;50:109547. doi: 10.1016/j.dib.2023.109547 (PMC10518673; doi:10.1016/j.dib.2023.109547)
Supplement: Supplementary file 1 [file mmc1.zip › Release/Process/Challenge Rules/D5-MDC/MDC Problem Description.pdf]

1 Contest Description

In this contest you are asked to design a Mechanically Driven Clamp (MDC) that will be mounted to the free end of a separately designed robotic arm. ***This challenge is focused on a mechanical system only (i.e. no internal computing or circuitry).***

The robotic arm will provide mechanical power to the MDC to close to an International Space Station (ISS) handrail (“Handrail”), maintain a hold on it, and release the Handrail. The below specification details how the MDC will work, its functional requirements and interface constraints/assumptions. A separate document provides detailed guidelines on how your design must be presented and submitted.

A prize of $250 be awarded for the lowest mass, technically feasible solution, submitted before 21:00 GMT on August 1st, 2018.

2 Concept of Operations – How the MDC needs to work

2.1 Normal Operations

Mechanical power is provided to the MDC at its interface to the robotic arm through either a rotating shaft or a shaft with a variable linear stroke. When provided mechanical power, the MDC must be able to perform two operations: 1) close, which involves attaching the MDC to the Handrail and maintaining a hold on it; 2) hold which involves maintaining the *attached* configuration while resisting externally applied loads and 3) release which involves releasing its hold on the Handrail, and returning to a *pre-attached* configuration. The relationships among the configurations and operations are illustrated in
Figure 1. The requirements for each operation (underlined) and configuration (italicized) are detailed in section 3.

2.2 Contingency (Emergency) Operations

There are several scenarios when normal operations may be disrupted. The ranges of permissible responses are detailed in section 3.4. This section summarizes the scenarios: 1) when the MDC attempts to close on the Handrail, but there is no Handrail present; 2) when the MDC experiences higher than expected loads while attached (e.g., because an astronaut or other object bumps or smashes into Astrobee); and 3) when an astronaut manually removes the MDC from the Handrail.

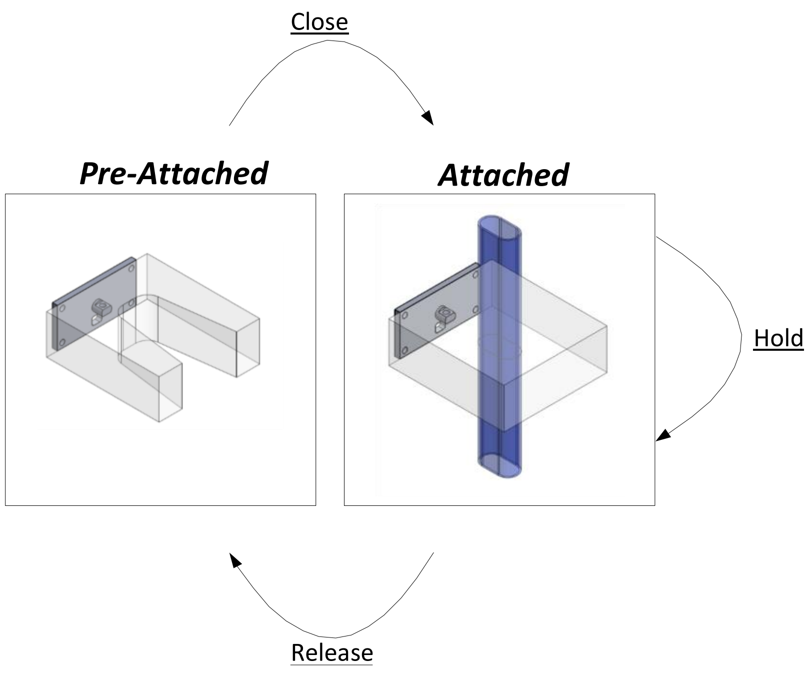

Figure 1 – Concept of Operations Illustration

3 Functional Requirements

This section details all of the functional requirements that the MDC must meet.

3.1 Motion Requirements

R1 Close: The MDC shall be able to move from the *pre-attached* configuration to the *attached* configuration.

R1.1 *Pre-attached* configuration: The pre-attached configuration volume is defined in Figure 2, as 114mm x 102mm x 40 mm, with a cutout to permit placement on the Handrail.

R1.2 Pre-attach offset: When Close is initiated, the MDC shall not be offset from the center of the Handrail by more than:

- x = 59 mm $\pm$ 7 mm
- y = 0mm $\pm$ 7 mm
- z = 0 mm $\pm$ 7 mm
- $\theta_x = 0 \pm 5$ degrees
- $\theta_y = 0 \pm 5$ degrees
- $\theta_z = 0 \pm 5$ degrees

The coordinate system for these offsets is shown graphically in Figure 3 Figure 4. The MDC shall only ever be controlled to attach to a standard Handrail as defined C4.

R1.3 *Attached* configuration: The MDC shall be considered *attached* when it is fixed to the Handrail; defined as being able to resist slipping or twisting when subjected to normal operating loads of up to 3.5 Nm about either the Y-axis or Z-axis (ref Figure 3).

R1.4 *Attached* configuration volume: When Close is complete, the MDC must be attached to the ISS Handrail within the attached configuration volume in Figure 4.

R2 Hold: The MDC shall be able to maintain a fixed attachment (defined in R1.3) for an extended period (per R6).

R3 Release: The MDC shall be able to release the Handrail and move from the *attached* configuration (R1.3) to the *pre-attached* configuration (R1.1).

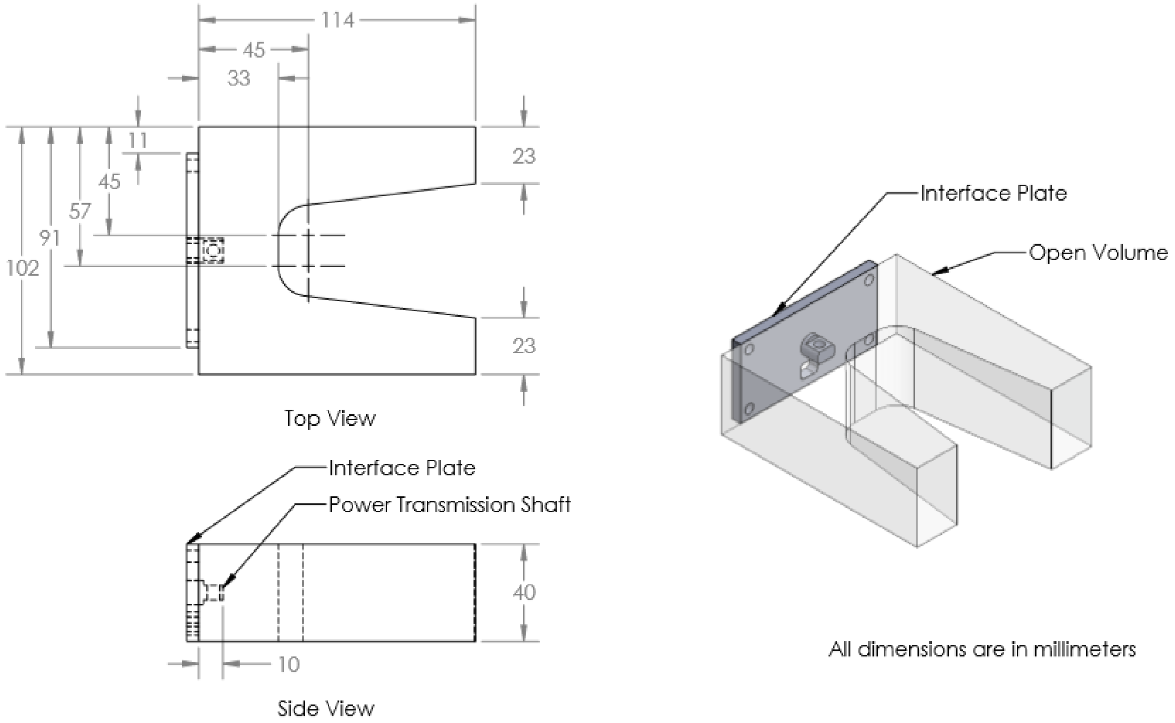

Figure 2 – Pre-attached Configuration

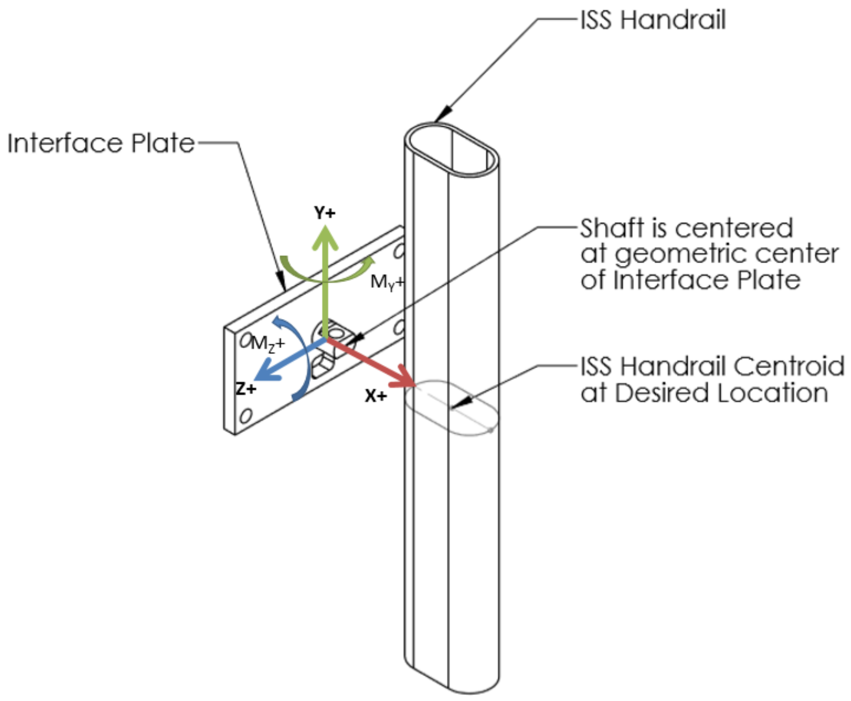

Figure 3 – MDC Frame of Reference

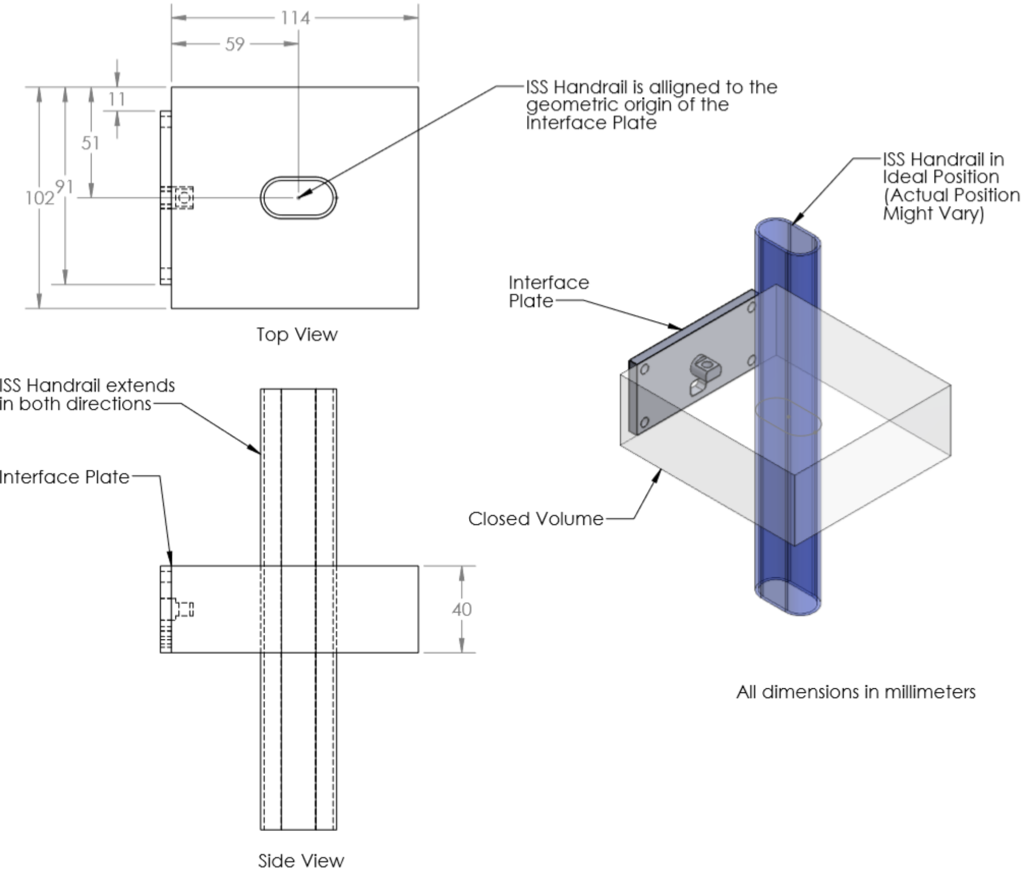

Figure 4 - Attached configuration

3.2 Sensor and Wiring Requirements

R4 The MDC design shall include enough sensors to provide the information that the robotic arm needs to control its operations. For both MDC configurations (i.e. *Pre-attached* and *Attached*), your submission must define a) what that configuration means in the context of your design and b) what sensing or signal outputs you are defining to enable the robotic arm to determine when the MDC completes an operation and is in either configuration. Your design must also include the placement of the selected sensors, if any are used.

R4.1 The categories of permissible sensors and their mechanical properties are provided in Table 1. No other sensors may be used. Further information about the design, mounting, and sensing elements of the available sensors is provided in the appendix.

R4.2 All sensors have wire bundles that need to be considered in your mechanical design and mass. In selecting sensors for your design, include mechanical accommodation of wires from the component to the interface plate. Wires should be fixed to structure to relieve strain if you expect the wires to be subjected to significant deflection or movement (e.g. pulling, twisting) during regular operations. In accommodating your sensor wire bundles, assume outside bundle diameter of 2.5 mm, with a 12.6 mm minimum bend radius, and a linear mass of 12.8 kg/km (26 AWG twisted shield pair bundles).

Table 1 - Permissible Sensor Options

	Volume	Mass	Information provided
No dedicated sensors – drive shaft characteristics	You may use an approach without dedicated sensors. The robot arm is able to sense torque (or force) and position of the input rotary (or linear) drive. There is no extra mass or volume for this approach, but you must clearly communicate how your design is intended to work in your submission.		
Continuous Rotation Sensor	Small knob with hole for sensing shaft (see Figure 9)	4 grams	Angle: Continuous and/or relative rotation of sensor shaft hole at 0.1 degrees resolution
Contact Sensor	Flat box with sensing face that depresses 1 mm (see Figure 10)	1 gram	Whether contact has been made: Discrete: On or Off
Linear Displacement Sensor	Flat plate with sliding sensing head that can slide 1" (see Figure 11)	10 grams	Position: Continuous and Relative Displacement Information at 1 mm resolution.
Force Sensor	Flat Plate that determines force placed on the sensing face (see Figure 12)	1 gram	Force: Continuous and absolute force from 0-100 N at 1 N resolution.

3.3 Resource Requirements

3.3.1 Timing Requirements

R5 Time to <u>Close</u>: The MDC shall be able to <u>close</u> within two minutes.

R6 Time in <u>Hold</u>: The MDC shall remain in an a*ttached* configuration on the Handrail for a maximum duration of 1 hr.

R7 Time to <u>Release</u>: The MDC shall be able to <u>release</u> within two minutes.

3.3.2 Energy Requirements

You may assume that only mechanical power is available from the robotic arm to drive your MDC. The details of that interface are described in C2.3.

R8 Mechanical Energy Budget: The MDC shall not use more than 4 Watt-hours to support all operations (R1-R3). Assume a maximum of 1 hr 26 minutes of passive operations (during <u>Hold</u>)

3.4 Safety Requirements

R9 The MDC shall have no sharp edges, defined as a radius of 3 mm, for astronaut safety.

R10 The MDC shall have no loops of material greater than 25.4 mm in diameter for astronaut safety, and no unsupported or unattached material more than 40 mm from the structure of the MDC.

R11 The MDC shall not damage itself through normal operations.

3.5 Environmental Requirements

R12 The MDC shall operate in the ISS zero gravity environment.

R13 The MDC shall operate in an atmosphere comparable to that of Earth. Assume temperature of 21 °C, and pressure of 101 kPA [1 atm], and relative humidity that is 40% - 70%.

R14 The MDC shall not contribute any particulates (e.g. dust) to the ISS atmosphere.

R15 The MDC shall enclose all lubricated components to prevent lubricants from leaking into the atmosphere of the ISS.

3.6 Contingency Requirements

R16 *No Handrail*: In some cases, the MDC may be controlled into attach configuration but there is no Handrail present. The MDC shall not damage itself while executing the operation.

R17 *Excessive Loads*: This scenario may occur if an astronaut or piece of equipment contacts Astrobee while the MDC is attached (including while experiencing normal operating loads per R1.3). The MDC shall break away from the Handrail if it experiences a force of greater than 18N [4 lbf] applied at the MDC interface in the negative Y-direction and a simultaneous moment of 6.7 Nm [3.7 ft-lbs] about the positive Z-axis as seen in Figure 5.

R18 *Astronaut intervention*: The MDC shall be removable from the Handrail by an astronaut. Assume an astronaut can apply a pull-away force of 35.6 N [8 lbf] in the negative X-direction as seen in Figure 5.

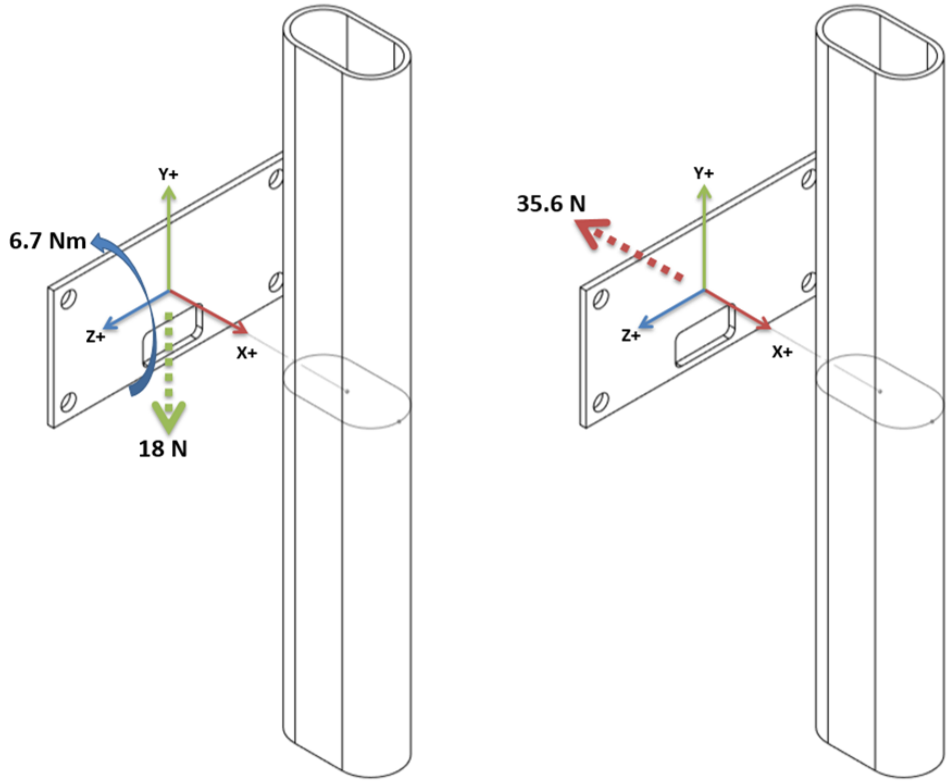

Figure 5 – Contingency loads: Excessive loads (left); Astronaut intervention loads (right)

4 Interface Requirements

The MDC has a fixed interface to a separately designed robotic arm and a dynamic interaction with Handrails. The section describes all constraints imposed by those interfaces.

4.1 MDC-Robotic Arm Interface

4.1.1 Mechanical Interface

C1 Constraint 1 (C1) Mounting Interface: The MDC shall mount to the interface plate shown in Figure 6. There are four available screw holes in the specified locations seen in Figure 7.

C1.1 All external loads are applied at the interface plate.

C1.2 All wires used (from sensor selection in Section 3.2) must fit through the Wiring Hole in the interface plate.

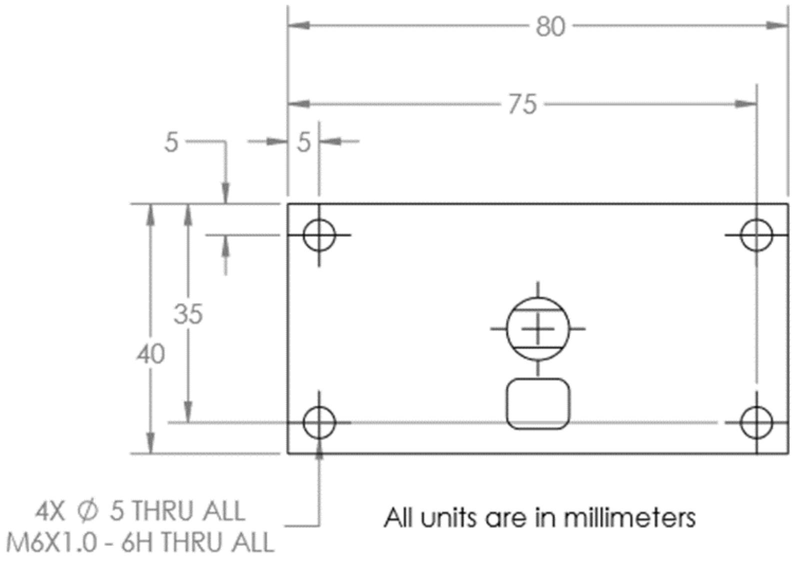

Figure 6 – MDC mounting holes

4.1.2 Power Interfaces

Mechanical power is provided for <u>Close</u> and <u>Release</u> operations. Specific constraints for electrical and mechanical power available are included below, in this section.

C2 *Mechanical Power interfaces*: there are two options for mechanical power for MDC – (a) rotary drive shaft, or a (b) linear drive shaft. You may only choose to use one option in your MDC design.
Mounting and geometry details of each drive shaft interface are described in Figure 7. Both options have the same mounting features – and flat feature and a threaded thru hole, M6 (fine thread).

- C2.1 The rotary shaft can rotate continuously in either direction, with 1° position accuracy.
- C2.2 The linear shaft has a 20 mm stroke, with 1 mm position accuracy.

C3 *Mechanical Power limit*: Maximum mechanical power available for MDC operations is 28 W [0.04 hp]. You may assume that the drive shaft can provide the torque (for rotary power interface) or force (for linear power interface) and speed required for your design.

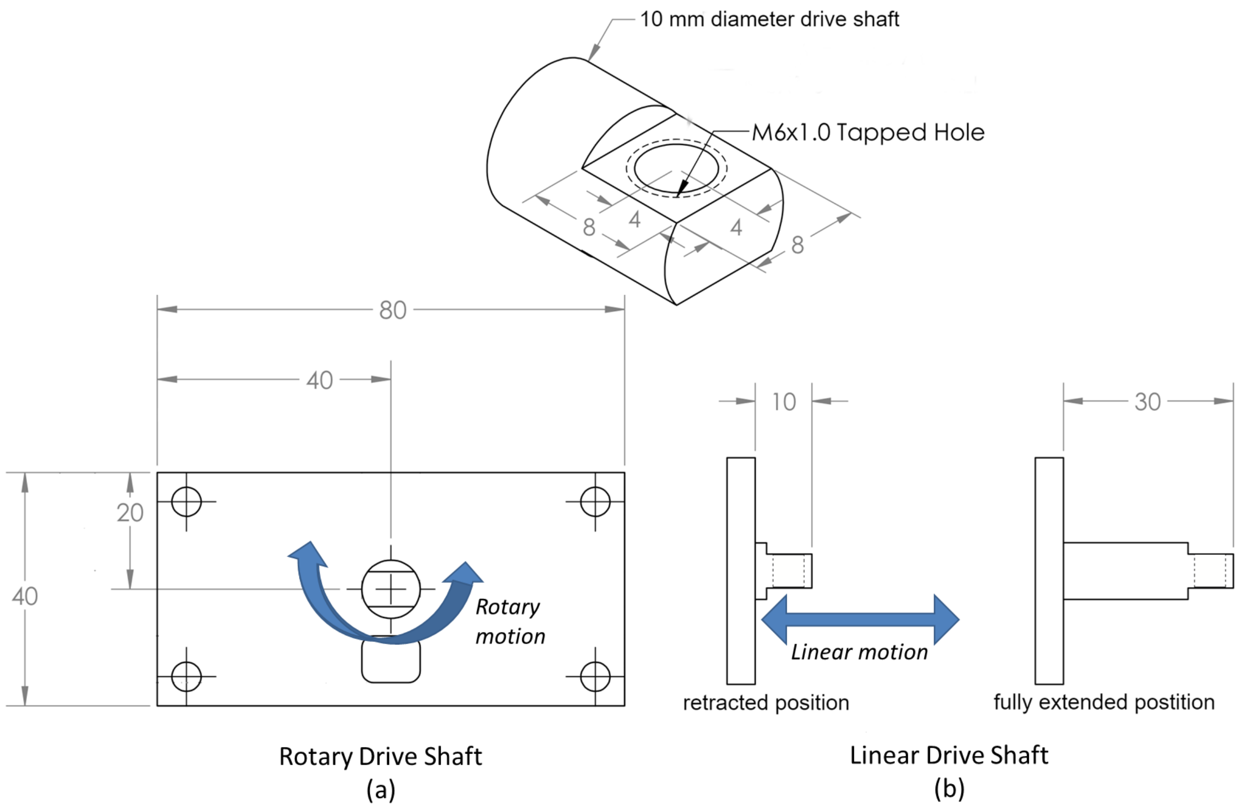

Figure 7 - MDC Mechanical Power Interface Options: (a) rotary shaft; (b) linear shaft

4.2 Handrail Interface

C4 Handrail definition:

- C4.1 The shape of a standard Handrail is defined in Figure 8.
- C4.2 The Handrail is made of anodized aluminum. Assume the material is 6061 Aluminum of type T4 in terms of material properties and friction properties.
- C4.3 The Handrail is a 1.59mm [1/16”] thick aluminum 6061 extrusion.

C5 The Handrail shall not be damaged during operations through excessive force (per R17 and R18). Damage includes, but is not limited to: crushing, denting, or bending.

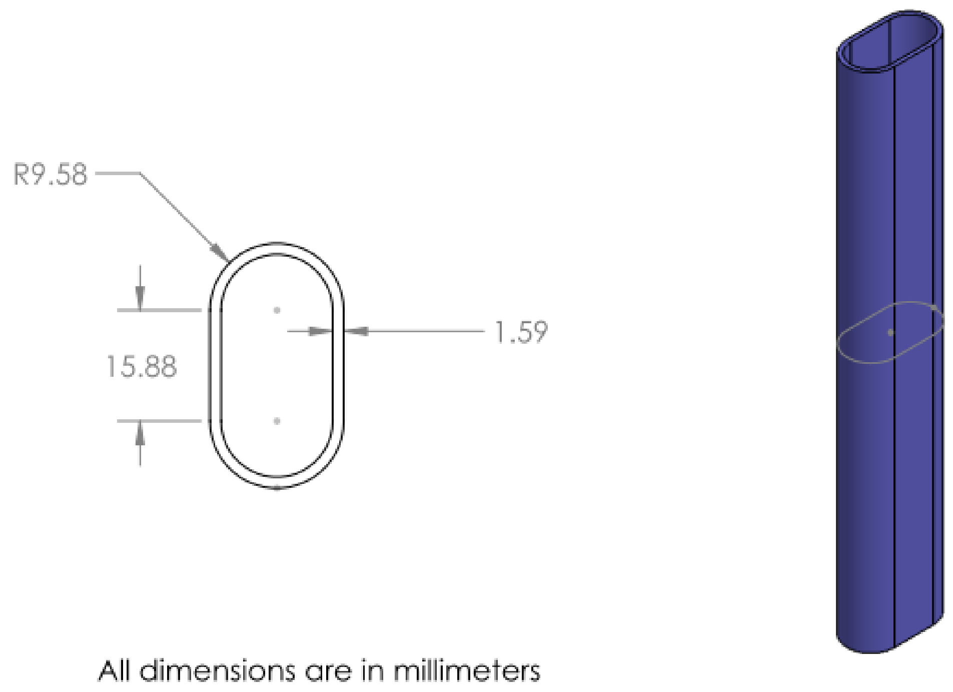

Figure 8 - ISS Handrail Definition

5 Sensor Appendix

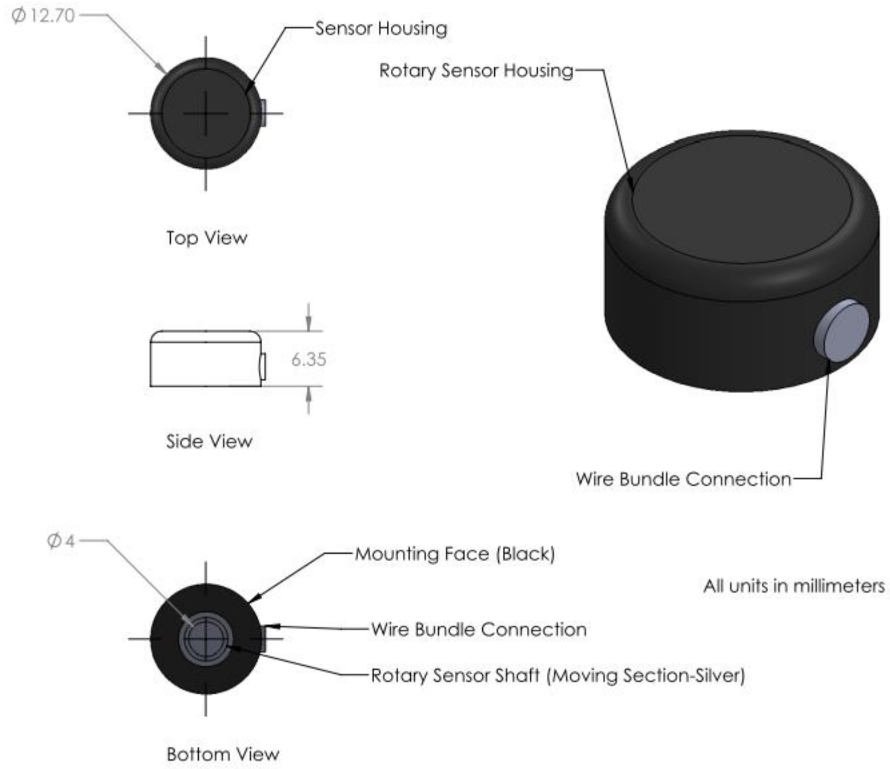

Figure 9: Continuous Rotation Sensor

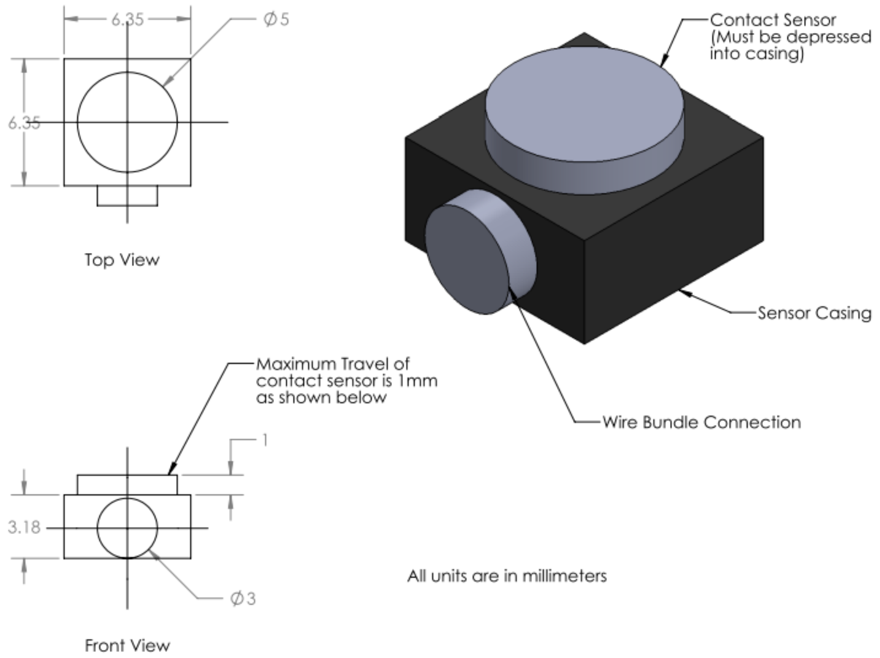

Figure 10: Contact Sensor

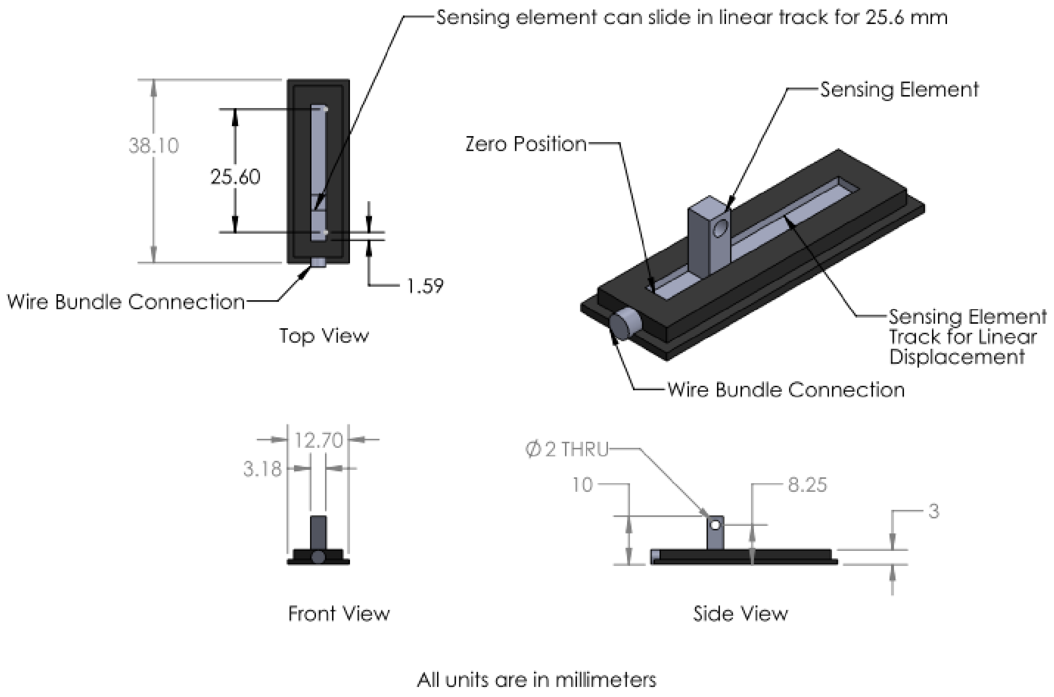

Figure 11: Linear Displacement Sensor

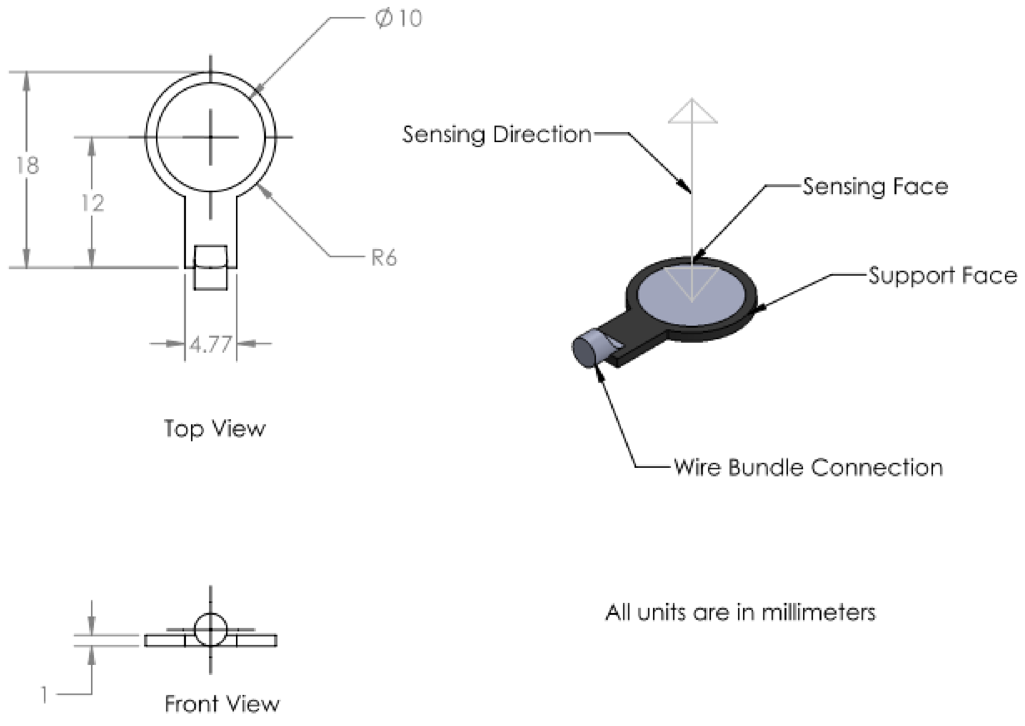

Figure 12: Force Sensor
