# Supplementary material for: Linking solver characteristics, solving processes and solution attributes: A data explainer for an open innovation generated robotic design dataset
Source: Data Brief. 2023 Sep 6;50:109547. doi: 10.1016/j.dib.2023.109547 (PMC10518673; doi:10.1016/j.dib.2023.109547)
Supplement: Supplementary file 1 [file mmc1.zip › Release/Process/Challenge Rules/D4-EMA/EMA Problem Description.pdf]

1 Contest Description

In this contest, you are asked to design an "Electro-Mechanical Arm" (EMA) that mechanically mounts directly to Astrobee. The EMA will be electrically-powered and driven by a (separately-designed) set of electronics hardware and control software.

Although the EMA will be electrically-powered, this contest is focused on the mechanical design only. You are being asked to design a mechanical system that will be driven and controlled by electrical components (like motors, switches and sensors). However, you are not required to design or select those components. You need only describe how each electrically driven component needs to work for your EMA design.

The EMA receives all power and control signals from the separately designed control system, but can accomplish the following motions when controlled: deploying from and re-stowing into a compact payload bay, attaching to and releasing from an International Space Station (ISS) Handrail, and orienting Astrobee by rotating in two directions ("Pan" and "Tilt").

The details below describe how the EMA should work, its functional requirements and interface constraints/assumptions. A separate document provides detailed guidelines on how your design must be presented and submitted.

A prize of $4000 will be awarded for the lowest mass, technically feasible solution, submitted before 21:00 GMT on September 14th, 2018.

2 Concept of Operations – How the EMA needs to work

2.1 Normal Operations

The EMA must be able to perform four high-level operations (underlined for emphasis in this document) when it receives power and control signals from Astrobee: <u>attach</u> securely to an ISS Handrail from a stowed configuration in Astrobee's Payload Bay, orient Astrobee (<u>pan</u> and <u>tilt</u>), and return to <u>stow</u> into the Astrobee Payload Bay. For reference, Figure 1 shows Astrobee with an open Payload Bay hovering near a Handrail and illustrates the coordinate systems. The requirements for each operation are detailed in Section 3.

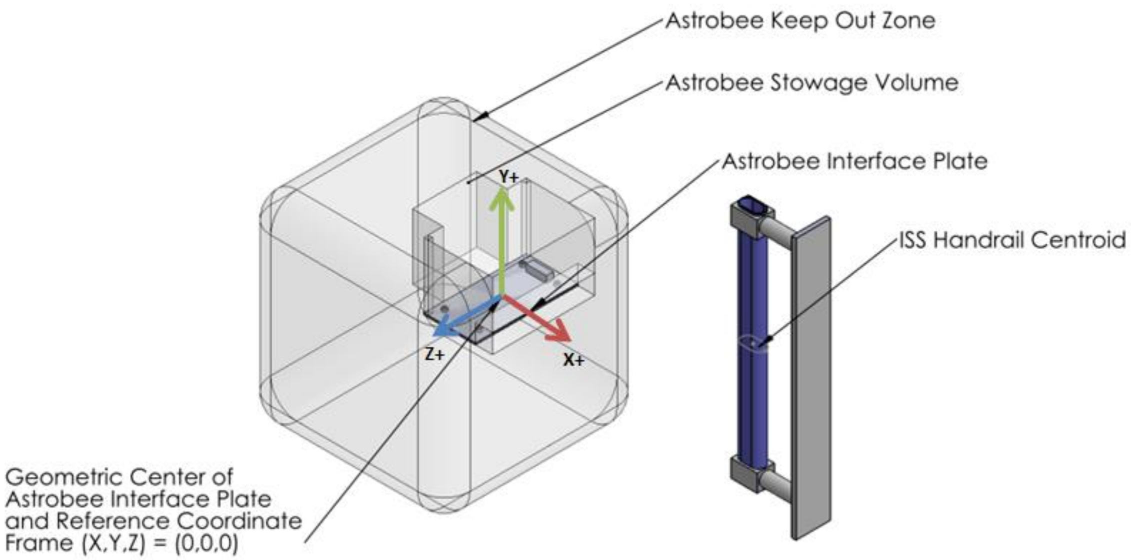

Figure 1 –Astrobee near an ISS Handrail

2.2 Contingency (Emergency) Operations

There are three scenarios when normal operations may be disrupted. Acceptable responses to these scenarios are detailed in section 3.4. This section summarizes those scenarios: (1) when the EMA experiences higher than expected loads while orienting and attached (e.g., because an astronaut or other object bumps or contacts Astrobee); (2) when an astronaut manually pulls on the EMA to remove the system from the handrail; and (3) when the EMA attempts to close on an ISS Handrail and no Handrail is present.

3 Functional Requirements

This section details all of the requirements that the EMA must meet. To clearly define the motion involved in these operations, we define a coordinate reference frame that has its origin at the geometric center of the Astrobee Interface Plate (see Figure 1).

3.1 Motion Requirements

R1 Attach: The EMA shall be able to move from a *stowed* configuration (inside Astrobee's Payload Bay– see C1) and attach to an ISS Handrail that could be located anywhere in the ISS Handrail workspace defined below

R1.1 ISS Handrail Workspace: EMA's "workspace" volume is defined, in Cartesian coordinates, by the centroid of the Handrail location (x, y, z), such that:

- 265 mm $\leq$ x $\leq$ 315 mm
- -25 mm $\leq$ y $\leq$ 125 mm
- -100 mm $\leq$ z $\leq$ 100 mm

With the origin (0,0,0) of this coordinate system at the center of the Astrobee Interface Plate (see C2). Figure 2 illustrates the workspace and reference coordinate system.

R1.2 ISS Handrail Orientation: You can assume that the ISS Handrail cross-section will remain parallel to the bottom of the Astrobee Payload Bay, as illustrated in Figure 1.

R1.3 *Attached* Configuration: The EMA shall be considered attached when it is fixed to the Handrail. Fixed is defined as being able to resist slipping or twisting when subjected to normal operating loads of up to 3.5 Nm about either the Y-axis or Z-axis (see Figure 3).

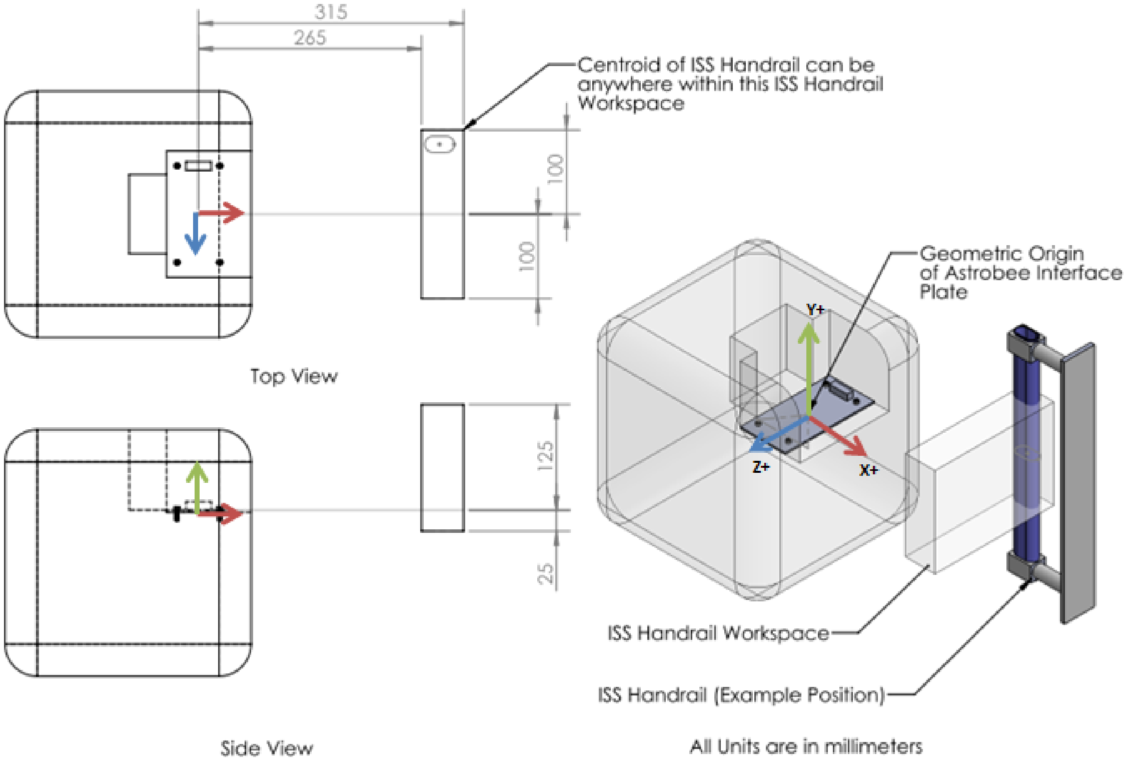

Figure 2 – ISS Handrail Workspace

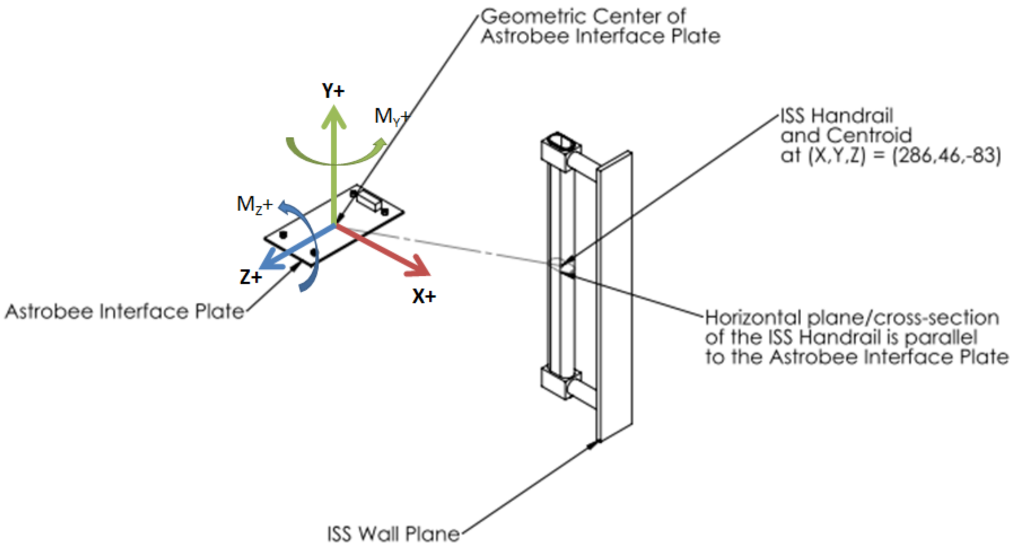

Figure 3 – EMA Frame of Reference.

R2 Orient: When *attached* to an ISS Handrail, the EMA shall be able to Pan and Tilt (rotate in two perpendicular directions) Astrobee about the ISS Handrail

R2.1 Center of Rotation: Center of rotation for Pan and Tilt must be within 130 mm of the ISS Handrail centroid. The selection of the exact position for the Pan/Tilt center of rotation is part of your EMA design. An illustration of what this could look like is shown in Figure 4.

R2.2 Pan Range of Motion and Accuracy: The EMA shall be able to pan through the range: -65° < θ_x < 65° , +/- 5° (see Figure 4)

R2.3 Tilt Range of Motion and Accuracy: the EMA shall be able to tilt through the range: 0° < θ_y < 90° , +/- 5° (see Figure 4)

R2.4 Single Movements: No simultaneous panning and tilting shall ever be requested.

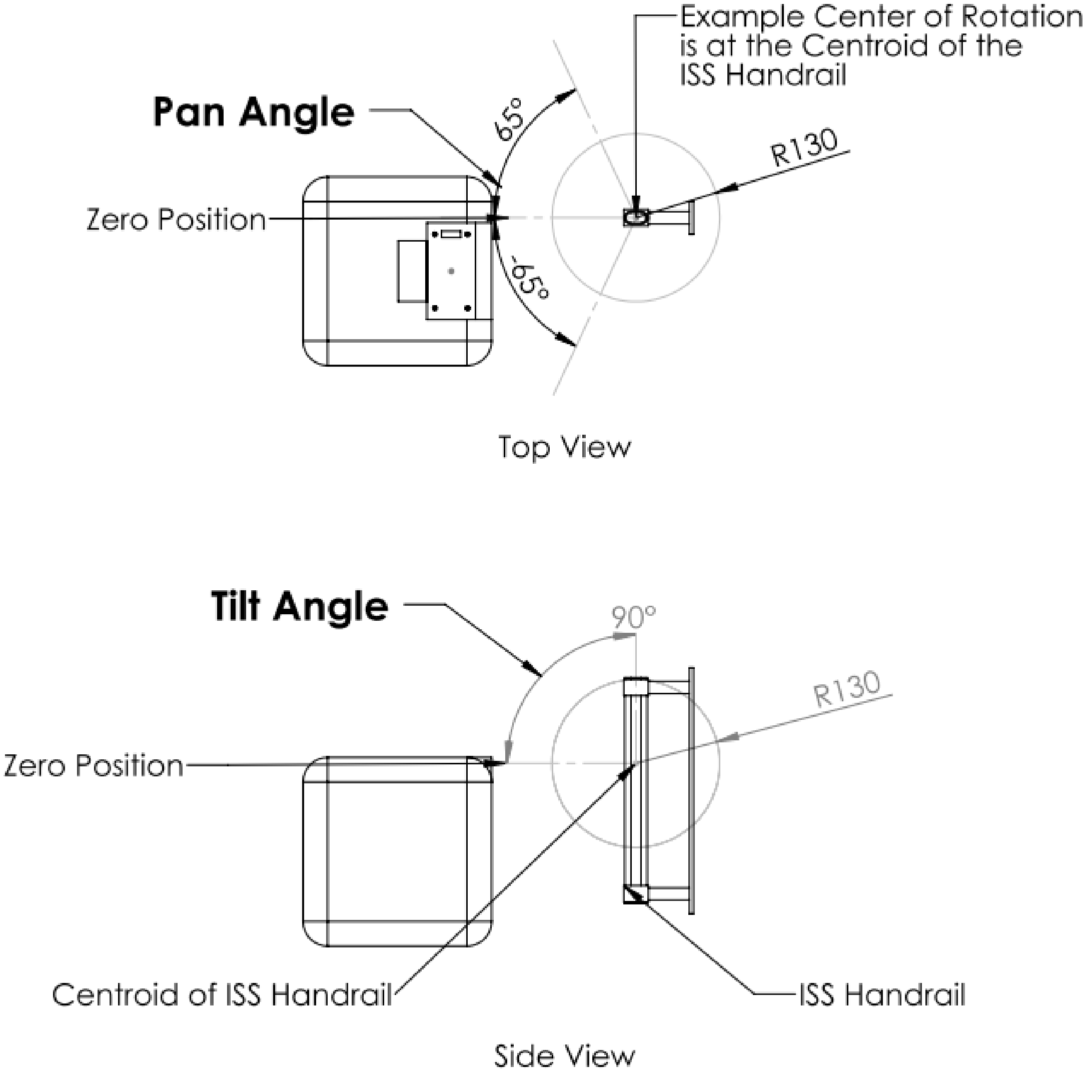

Figure 4 - Pan & Tilt Center of Rotation and Ranges of Motion

R3 Stow: The EMA shall be able to detach from the ISS Handrail and maneuver to return to its *Stowed* configuration inside Astrobee's Payload Bay (defined in C1).

Stowed Configuration: All components of EMA must fit within the Astrobee Payload bay in the *Stowed* configuration.

R4 Active Degrees of Freedom: The EMA may use as many electrically-driven degrees of freedom as necessary to accomplish the required operations (R1, R2, R3). Examples of active degrees of freedom include motors and linear actuators.

However, every active degree of freedom will add mass to your EMA design. Since contest submissions will be judged on estimated total mass and feasibility, designs that require fewer or smaller active degrees of freedom are preferred. Please refer to the Submission Guidelines document for how to describe active degrees of freedom in you EMA design.

R5 Sensors: The EMA may use as many electrically-driven sensors as necessary to accomplish the required operations (R1,R2, and R3).

However, every sensor will add mass to your EMA design. Since contest submissions will be judged on estimated total mass and feasibility, designs that require fewer sensors are preferred.

R6 Astrobee Contact: EMA shall not contact Astrobee, including its Payload Bay walls, during any operations. This "Astrobee Keep Out Zone" is illustrated in Figure 2, and a detailed definition of Astrobee dimensions is in C1 below.

3.2 Safety Requirements

R7 The EMA shall have no sharp edges, defined as a radius of 3 mm, for astronaut safety.

R8 The EMA shall have no loops of material greater than 25.4 mm in diameter for astronaut safety and unsupported or unattached for more than 40 mm from the structure of the EMA.

R9 The EMA shall not damage itself through normal operations.

3.3 Environmental Requirements

R10 The EMA shall operate in the ISS zero gravity environment.

R11 The EMA shall operate in an atmosphere comparable to that of Earth. Assume temperature of 21 °C [70 °F], and pressure of 101 kPa [1 atm], and relative humidity that is 40% - 70%.

R12 The EMA shall not contribute any particulates (e.g. dust) to the ISS atmosphere.

R13 The EMA shall enclose all lubricated components to prevent lubricants from leaking into the atmosphere of the ISS.

3.4 Contingency Requirements

R14 Excessive loads: This scenario may occur if an astronaut or piece of equipment contacts Astrobee while the EMA is secured to the ISS Handrail The EMA shall maintain normal Orient operations (R2) when subject to a force of up to 18N applied at the baseplate in the negative Y-direction (see Figure 5a).

R15 Astronaut intervention: Astrobee is required to be removable from the ISS Handrail by an astronaut after the EMA is attached to the ISS Handrail. Assume that for the EMA, this translates to a pull-away force of 35.6 N in the negative X-direction (see Figure 5b) applied through the baseplate.

R16 No Handrail: In some cases, a EMA may be driven to attach, but there is no ISS Handrail at the expected location. The EMA shall not damage itself while trying to complete the operation.

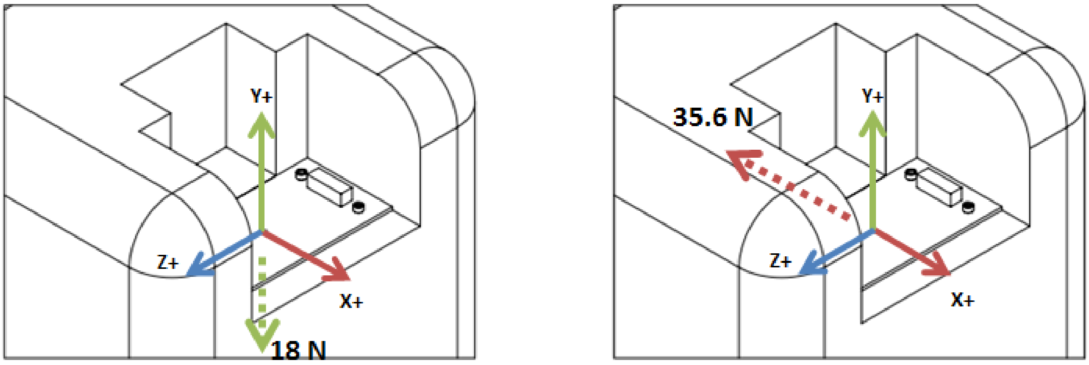

Figure 5 – Contingency Loads a) shows Excessive Loads; b) shows Astronaut intervention loads

4 Interface Requirements

The EMA has a fixed interface to Astrobee and a dynamic interaction with ISS Handrails. The section describes all constraints imposed by those interfaces.

4.1 EMA-Astrobee Mechanical Interface

C1 Constraint 1 (C1) Volume Constraint: The EMA is stowed in Astrobee's Payload Bay when in the *stowed* configuration. Figure 6 and Figure 7 define the Payload Bay with respect to Astrobee.

- C1.1 Figure 6 defines the dimensions of the Astrobee volume that must not be contacted (Astrobee "Keep Out Zone")
- C1.2 Figure 7 defines the dimensions of the Astrobee Payload Bay, which the EMA must stow within in the *stowed* configuration.

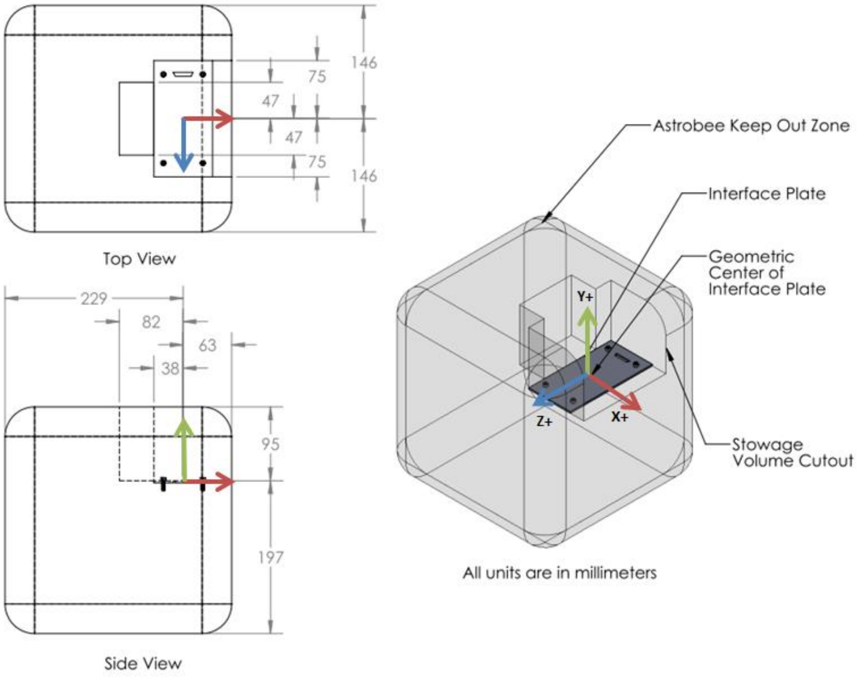

Figure 6 - Astrobee Keep-Out Zone and Adapter Plate

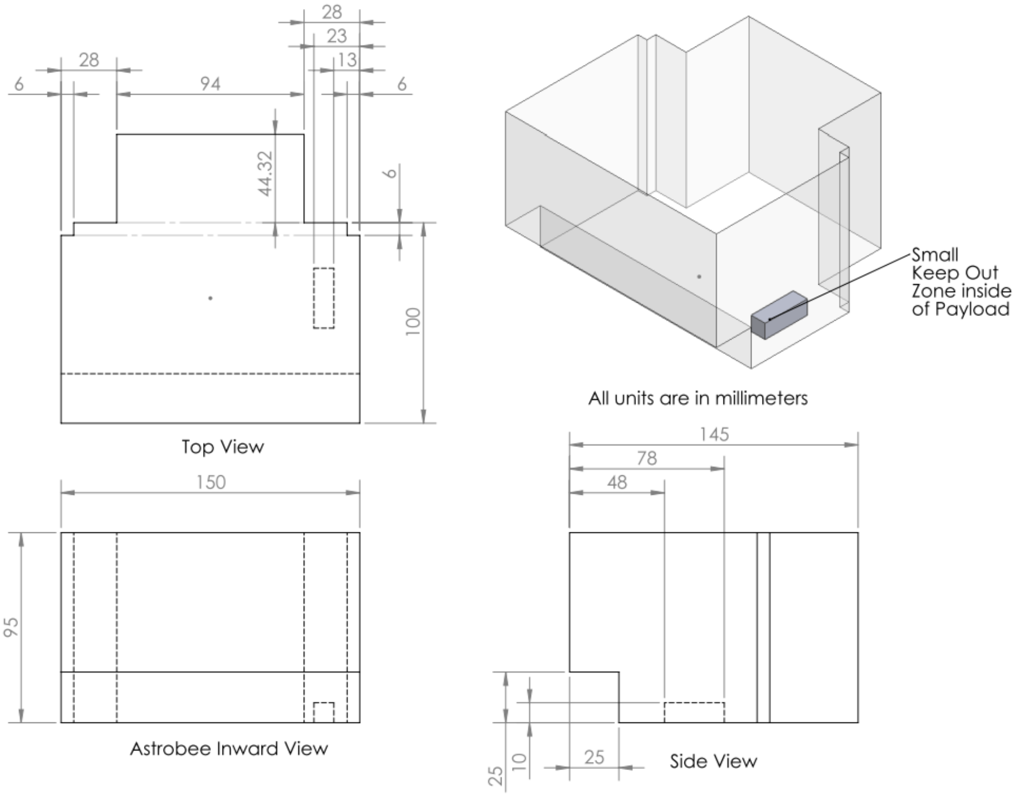

Figure 7 – Astrobee Payload Bay

C2 Mounting Interface: The EMA shall mechanically mount to a flat metal plate shown in Figure 8.

- C2.1 All external loads are applied at the interface plate.
- C2.2 There are four available screw holes in the specified locations.

C3 Mass Properties: Treat Astrobee as mass of 6 kg with center of mass = (-83mm, -48 mm, 0 mm) relative to the EMA Coordinate Reference Frame as seen in Figure 1.

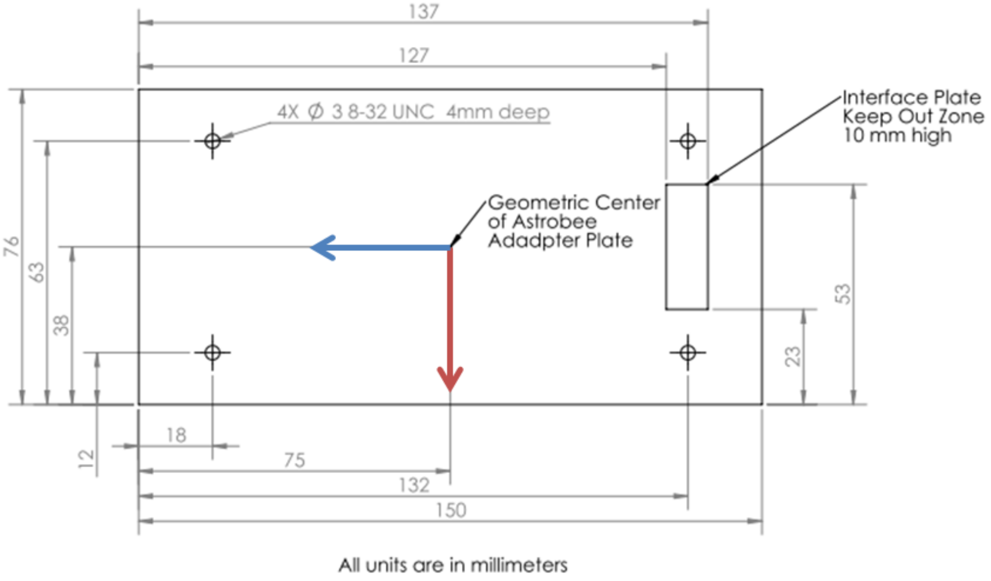

Figure 8 - Astrobee Interface Plate

4.2 Handrail Interface

C4 ISS Handrail definition:

- C4.1 The shape of a standard ISS Handrail is defined in Figure 9.
- C4.2 The ISS Handrail is made of anodized aluminum. Assume the material is 6061 Aluminum of type T4 in terms of material properties and friction properties.
- C4.3 The ISS Handrail is a 1.59mm [1/16"] thick aluminum 6061 extrusion.

C5 The ISS Handrail shall not be damaged during operations. Damage includes, but is not limited to: crushing, denting, or bending.

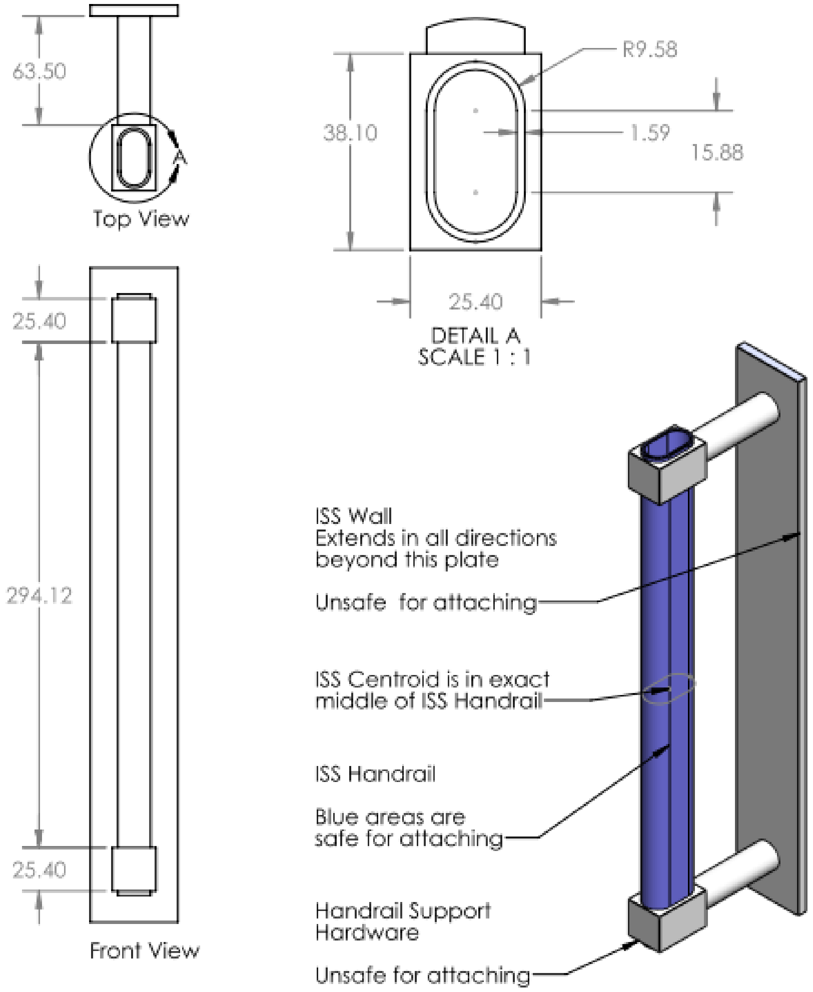

Figure 9- ISS Handrail Definition
