# Supplementary material for: Linking solver characteristics, solving processes and solution attributes: A data explainer for an open innovation generated robotic design dataset
Source: Data Brief. 2023 Sep 6;50:109547. doi: 10.1016/j.dib.2023.109547 (PMC10518673; doi:10.1016/j.dib.2023.109547)
Supplement: Supplementary file 1 [file mmc1.zip › Release/Process/Challenge Rules/D4-EMA/EMA Submission Guidelines.pdf]

Submission Guidelines for the Electro-Mechanical Arm

In this contest, you were asked to design an Electro-Mechanical Arm that will be mounted to Astrobee and attach to an ISS Handrail

This document provides detailed guidelines on how you must describe and present each aspect of your design in order to be considered for the prize. This document looks long but very little text is required. Your submission document must include each of the sections detailed below and all of the information requested in each. Several templates and examples are provided to clarify what constitutes a complete solution.

Table of Contents

1 SUBMISSION SUMMARY 2

2 FUNCTIONAL DESCRIPTION 2

2.1 NARRATIVE (WORD) DESCRIPTION OF DESIGN 2
2.2 FUNCTIONAL ANALYSIS 2

3 MASS SUMMARY AND COMPONENT LIST 3

4 ACTIVE ELEMENT DESCRIPTIONS 3

4.1 ACTIVE DEGREES OF FREEDOM TABLE 3
4.2 SENSING ELEMENT TABLE 4

5 SYSTEM LAYOUT 4

6 DESIGN DRAWINGS 5

7 EXIT SURVEY 6

Minimum Content Requirement: Your submission should include a Table of Contents. Use the exact section titles in the Table of Contents shown above to ensure that your submission is complete.

1 Submission Summary

Mass (kilograms)	
Exit Survey Confirmation Code	

2 Functional Description

2.1 Narrative (word) description of design

In this section, describe how your design for the Electro-Mechanical Arm system works. In a few sentences, please describe how your solution does each of the following:

1) Attach: How does your EMA move to attach to the ISS Handrail from a stowed configuration (R1)? Can your EMA attach to the ISS Handrail in all eight corners of the ISS Workspace?
2) Orient - Pan: How does your EMA move to pan the Astrobee side to side (R2)?
3) Orient - Tilt: How does your EMA move to tilt the Astrobee up and down (R2)?
4) Stow: How does your EMA move to stow itself into the Astrobee payload bay (R3)?
5) How does your EMA release from the ISS Handrail when the astronaut pulls away (R22)?

Although it is not required, you may embed images with sketches, models, storyboards or other illustrations in your written descriptions to help explain how your EMA moves.

Minimum content requirement: Text reponse to each of the above questions.

2.2 Functional Analysis

In this section, describe your logic and/or analysis for the following aspects of your EMA design. Including equations and mathematics is acceptable if it helps clarify the logic behind your design, but please ensure that it will be understood by our reviewers by annotating your process or describing the math being done and why.

(1) What is the typical force you could expect the EMA will exert on the Handrail while *attached* (R1 and R2)?
(2) How much force could be applied to the ISS Handrail while *attached* and excessive loads are applied (R15)?
(3) How many active degrees of freedom does your design have?
(4) During each operation, when do you need maximum force/torque in each driven degree of freedom and what is that amount of force or torque? Describe what is happening during the maximum force/torque.

Mimumum content requirement: Text responding to each of the above questions. Response must include numerical value.

3 Mass Summary and Component List

In this section, list all the elements of your EMA solution using the template provided [EMAMassTemplate, available .odt, .xlsx]. For each component/piece/part, include an estimate of its mass and a brief explanation of where the estimate came from. Please be sure to include the reasons supporting your mass estimate for each element since they will be part of the evaluation of the credibility of your EMA mass estimate.

Table 1 provides an example of how the template should be filled in.

			Estimated Mass per Unit (kg)	Quantity (# units)	Total Mass (kg)	Basis of estimate
1.0	Subsystem #1					
	Mechanism #1		0.100	1.0	0.10	Typical mass of component that I use all the time in design of systemX.
	Sensor #1-a		0.010	1.0	0.01	Called some former coworker who builds these, and asked for a typical masses
	Mechanism #2		0.080	1.0	0.08	Made a CAD model, assumed SS316, to obtain this mass
	Sensor #1-b		0.001	1.0	0.00	Weighed a prototype I built

Table 1 - Mass Summary and Components List example

In your mass summary, please include the following elements at a minimum. This is not an exhaustive list either, so if your design incorporates a wide variety of components, please include them into your table:

- All moving components
- All structural elements
- Bolts, screws, rivets, and even zip ties

Minimum content requirement: Paste your filled tables into this section of the document. No additional text is required.

4 Active Element Descriptions

4.1 Active Degrees of Freedom Table

For each independent degree of freedom, the EMA design should clearly define the type of motion, description of motion, range of motion, maximum force/torque needed, and any additional motion requirements. For example:

Name of Degree of Freedom	Type of Motion	Description of Motion	Range of Motion	Maximum Force/ Torque	Additional Motion Requirements
Pivot	*rotary*	*rotation of Beam #4 around Shaft #1 as shown in Figure 1*	*Continuous (360 degrees)*	*2 Nm*	*Stall torque = 3 Nm*
Include an additional row for each active degree of freedom					

If you used a Commercial, Off-The-Shelf Actuator in your design, please include the information in the same table above.

Feel free to use describe any other important performance or mechanical characteristics in the last (*additional motion requirements)* column.

4.2 Sensing Elements Table

For each type of information needed to operate EMA, your design should clearly define the type of information needed, description and range of information collected, required resolution of information, and any additional sensor requirements. For example:

Name of sensor	Type of information	Description of information collected	Range of information	Resolution of information	Additional information requirements
Handrail proximity sensor	*Distance*	*Position of Linkage #2 relative to Bracket A, as shown in Figure 3*	*0 to 90 mm*	*0.1 mm*	*Need distance measured every 0.5 s*
Include an additional row for each type of information necessary for your EMA design to work					

If you used a Commercial, Off-The-Shelf Actuator in your design, please include the information in the same table above.

Feel free to use describe any other functional, performance, or physical characteristics in the *additional sensor requirements* column.

Minimum content requirement: Paste your two filled tables into this section of the document.

5 System Layout

In this section, provide a diagram(s) identifying all the physical components/pieces/parts of your EMA design and how components connect to, move and/or, power each other.

Please use the names of components from the Mass Summary and Component List described in section 3.

Use a schematic similar to an "exploded view" of your design. See Figure 2 for an example. The defining feature of an exploded view is that it conveys information about how all the mechanical pieces connect to one another. Please include some information about how electrical connections are made or how it wraps around joints.

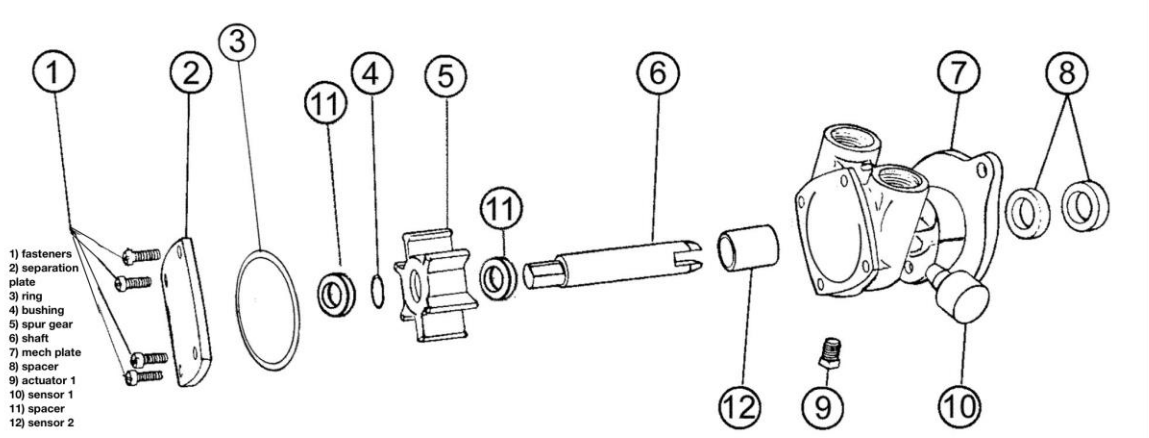

Figure 1 - Mechanical (exploded view) schematic – example

Minimum content requirement: Your mechanical schematic

6 Design Drawings

In this section, include engineering-style drawings showing your solution's physical configuration for each of the following.

1) Attached to an ISS Handrail at (300 mm, 50 mm, -95 mm)
2) Attached to a Handrail at (300, 50, -95), with Astrobee oriented with Tilt angle = 30° and Pan angle = 45°.
3) Stowed in the Astrobee Payload Bay. Indicate closest distance to any Payload Bay wall in this stowed configuration

You can use any CAD software you like, sketch them by hand or photograph a prototype, but accurate dimensions of the whole system are required (we'd like to clearly see that your design can fit in its stowage volume). For each view, please provide at least one off-angle view to show perspective. Please label all elements and subassemblies. Use the names specified in your Mass Summary and Component List (section 3).

Minimum content requirement: Three design drawing figures – one for each configuration listed above. Figures must be clearly labeled and dimensioned.

7 Exit Survey

To complete your submission, please take the Exit Survey by going to this webpage: https://seasgwu.qualtrics.com/jfe/form/SV_2r9DaeSlh48uMcZ

At the end of the survey you will receive a unique code. In your submission include this section and the text: Exit Survey for Freelancer <<insert Freelancer username>> complete per completion code: <<insert completion code>>.

To be complete, your submission must include the following text: Exit Survey for Freelancer <<insert Freelancer username>> complete per completion code: <<insert completion code>>.
