# Supplementary material for: Linking solver characteristics, solving processes and solution attributes: A data explainer for an open innovation generated robotic design dataset
Source: Data Brief. 2023 Sep 6;50:109547. doi: 10.1016/j.dib.2023.109547 (PMC10518673; doi:10.1016/j.dib.2023.109547)
Supplement: Supplementary file 1 [file mmc1.zip › Release/Process/Challenge Rules/D4-EMA/EMA Blurb.pdf]

Electro-Mechanical Arm (EMA)

In this contest, you are asked to design an "Electro-Mechanical Arm" (EMA) that mechanically mounts directly to Astrobee. The EMA will be electrically-powered and driven by a (separately-designed) set of electronics hardware and control software. This challenge is focused on the mechanical design of a robotic arm controlled by a separately designed piece of hardware.

How it works: Initially, the EMA will be stowed in Astrobee's payload bay. When powered and controlled by a separately-designed motion control system, the EMA shall be able to accomplish the following high-level actions: 1) unpacking from a stowed configuration in Astobee's compact payload bay and attaching to an International Space Station (ISS) Handrail, 2) orienting Astrobee by rotating in two directions ("pan" and "tilt"), and 3) detaching from the Handrail and (re-)stowing in the payload bay.

Click on the links below to see detailed design instructions, constraints and solution templates for this problem.

Challenge Rules: A prize of **$4,000** will be awarded for the **lowest mass, technically feasible** solution submitted by September 14th, 2018. No working prototype is required for submission, but the design must be sufficiently detailed to allow experts to assess the feasibility of your design (i.e., comply with all requirements) and the credibility of your mass estimate. Only complete submission packages will be evaluated.

Attachments:

EMAProblemDescription.pdf

EMASubmissionGuidelines.pdf

Templates

- EMAMassTemplate [.xlsx .ods]
- EMAPowerTemplate [.xlsx .ods]
