# Supplementary material for: Linking solver characteristics, solving processes and solution attributes: A data explainer for an open innovation generated robotic design dataset
Source: Data Brief. 2023 Sep 6;50:109547. doi: 10.1016/j.dib.2023.109547 (PMC10518673; doi:10.1016/j.dib.2023.109547)
Supplement: Supplementary file 1 [file mmc1.zip › Release/Process/Challenge Rules/D2-SAM/SAM Blurb.docx]

“Smart” Attachment Mechanism (no positioning capability) (SAM)

In this challenge, you are asked to design a “Smart” Attachment Mechanism (SAM) that will be mounted to the free end of a separately designed robotic arm. The SAM receives all external power and high-level commands through its interface to the robotic arm, but implements the below functions autonomously.

How it works: Initially, the SAM will be packed in a stowed configuration. Once activated, the SAM must be capable of autonomously performing five high-level actions: 1) unpack itself in preparation for placement near a Handrail, 2) close on the Handrail, 3) maintain a hold on the handrail for an extended period of time, 4) release from the handrail, and 5) pack back into the initial stowed configuration.

*Click on the links below to see detailed design instructions, constraints and solution templates for this problem.*

Challenge rules: A prize of **$1,500** will be awarded for the **lowest mass, technically feasible** solution submitted by **June 28^th^ 2018**. No working prototype is required for submission, but the design must be sufficiently detailed to allow experts to assess the feasibility of your design (i.e., comply with all requirements) and the credibility of your mass estimate. Only complete submission packages will be evaluated.

Attachments:

SAMProblemDescription.pdf

SAMSubmissionGuidelines.pdf

Templates

- SAMMassTemplate [.xlsx, odt, [google docs](https://docs.google.com/spreadsheets/d/1UmqD0LIF9dLLOzj5aM8AAJZNtRzuXSuKXvUyyBBan80/edit?usp=sharing)]
- SAMPowerProfileTemplate [.xlsx, .odt, [google docs](https://docs.google.com/spreadsheets/d/1bWctAfjUNZsxvCb61tCjyArO5FG0sYFHzfSiaIF3RS4/edit?usp=sharing)]

Steve, actual links for mass: <https://docs.google.com/spreadsheets/d/1UmqD0LIF9dLLOzj5aM8AAJZNtRzuXSuKXvUyyBBan80/edit?usp=sharing>

Power: <https://docs.google.com/spreadsheets/d/1bWctAfjUNZsxvCb61tCjyArO5FG0sYFHzfSiaIF3RS4/edit?usp=sharing>

Actual text from contest description field on Freelancer site:

Design a “Smart” Attachment Mechanism (no positioning capability) (SAM)

In this challenge, you are asked to design a “Smart” Attachment Mechanism (SAM) that will be mounted to the free end of a separately designed robotic arm. The SAM receives all external power and high-level commands through its interface to the robotic arm, but implements the below functions autonomously.

How it works: Initially, the SAM will be packed in a stowed configuration. Once activated, the SAM must be capable of autonomously performing five high-level actions: 
1) unpack itself in preparation for placement near a Handrail, 
2) close on the Handrail, 
3) maintain a hold on the handrail for an extended period of time, 
4) release from the handrail, and 
5) pack back into the initial stowed configuration. 

Click on the links below to see detailed design instructions, constraints and solution templates for this problem. 
- The SAMMassTemplate may be submitted in any of the 3 formats provided: XLSX, ODT, or Google Sheets (<https://docs.google.com/spreadsheets/d/1UmqD0LIF9dLLOzj5aM8AAJZNtRzuXSuKXvUyyBBan80/edit?usp=sharing>)
- The SAMPowerProfileTemplate may be submitted in any of the 3 formats provided: XLSX, ODT, or Google Sheets (<https://docs.google.com/spreadsheets/d/1bWctAfjUNZsxvCb61tCjyArO5FG0sYFHzfSiaIF3RS4/edit?usp=sharing>)

Challenge rules: 
The prize will be awarded for the lowest mass, technically feasible solution, submitted by the contest deadline. 
No working prototype is required for submission, but the design must be sufficiently detailed to allow experts to assess the feasibility of your design (i.e., comply with all requirements) and the credibility of your mass estimate. 
Only complete submission packages will be evaluated (see attachments).

NASA may select multiple winners or provide additional prize compensation on entries that are particularly novel or innovative.

NASA will be available to respond to clarifying questions, but feedback on quality is otherwise limited. 
All complete submissions will be confirmed with 3-star ratings. 
Note that final judgement of quality and winners will only happen after the submission deadline.
