# Supplementary material for: Linking solver characteristics, solving processes and solution attributes: A data explainer for an open innovation generated robotic design dataset
Source: Data Brief. 2023 Sep 6;50:109547. doi: 10.1016/j.dib.2023.109547 (PMC10518673; doi:10.1016/j.dib.2023.109547)
Supplement: Supplementary file 1 [file mmc1.zip › Release/Process/Challenge Rules/D2-SAM/SAM Problem Description.pdf]

# 1 Contest Description

In this contest, you are asked to design a “Smart” Attachment Mechanism (SAM) that will be mounted to the free end of a separately designed robotic arm. The SAM receives all power and high-level commands through its interface to the robotic arm, but implements the following functions autonomously: packing and unpacking from a stowed configuration, closing on and releasing from an International Space Station (ISS) Handrail and maintaining a hold on that Handrail. The below specification details how the SAM will work, it’s functional requirements and interface constraints/assumptions. A separate document provides detailed guidelines on how your design must be presented and submitted.

**A prize of $1,500 will be awarded for the lowest mass, technically feasible solution, submitted before 13:00 GMT on June 28st 2018.**

# 2 Concept of Operations – How the SAM needs to work

## 2.1 Normal Operations

The SAM must be able to autonomously perform six operations: 1) unpack, which involves a transition from the *packed* configuration to the *open* configuration; 2) close, which involves a transition from the *open* configuration to the *attached* configuration, 3) hold, which involves maintaining the *attached* configuration while resisting externally applied loads; 4) release, which involves a transition from the *attached* configuration to the *open* configuration; 5) pack, which involves a transition from *open* configuration to *packed* configuration; and 6) standby which is a low powered waiting mode used between unpack and close, and release and pack.

In transitioning among configurations (i.e., from *packed* to *open* and *open* to *attached)*, the SAM must never exceed its *dynamic envelope*. The relationships among the configurations and operations are illustrated in Figure 1. The requirements for each operation (underlined) and intermediate configuration (italicized) are detailed in section 3.

## 2.2 Contingency (Emergency) Operations

There are several scenarios when normal operations may be disrupted. The ranges of permissible responses are detailed in section 3.6. This section summarizes those scenarios. Contingency operations may occur when: 1) the SAM is commanded to attach, but there is no handrail present; 2) when the SAM experiences higher than expected loads while attached (e.g., because an astronaut or other object bumps or smashes into Astrobee); and 3) when an astronaut manually removes the SAM from the handrail.

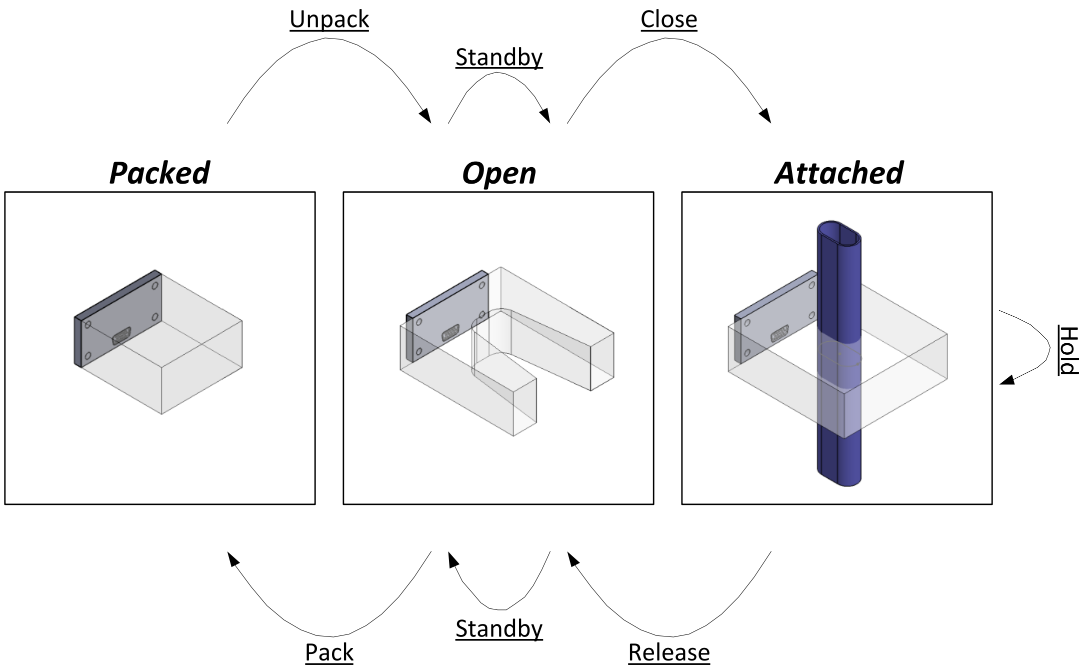


**Figure 1 – Concept of Operations Illustration**

# 3 Functional Requirements

This section details all of the functional requirements that the SAM must meet.

## 3.1 Motion Requirements

R1 Unpacking: The SAM shall be able to move from the *packed* configuration and stop moving in the *open* configuration without exceeding the *dynamic envelope.*

R1.1 Packed configuration: The packed configuration volume is defined in Figure 2, as 76.2mm x 76.2 mm x 38.1mm [3” x 3” x 1.5”]

R1.2 Open configuration: The open configuration volume is defined in Figure 3, as 114.3mm x 101.6mm x 38.1 mm [4.5” x 4” x 1.5”], with a cutout to permit placement on the Handrail.

R1.3 Dynamic envelope: The dynamic envelope is defined in Figure 4, as 120.65 x 114.3 x 50.8 mm [4.75” x 4.5” x 2“].

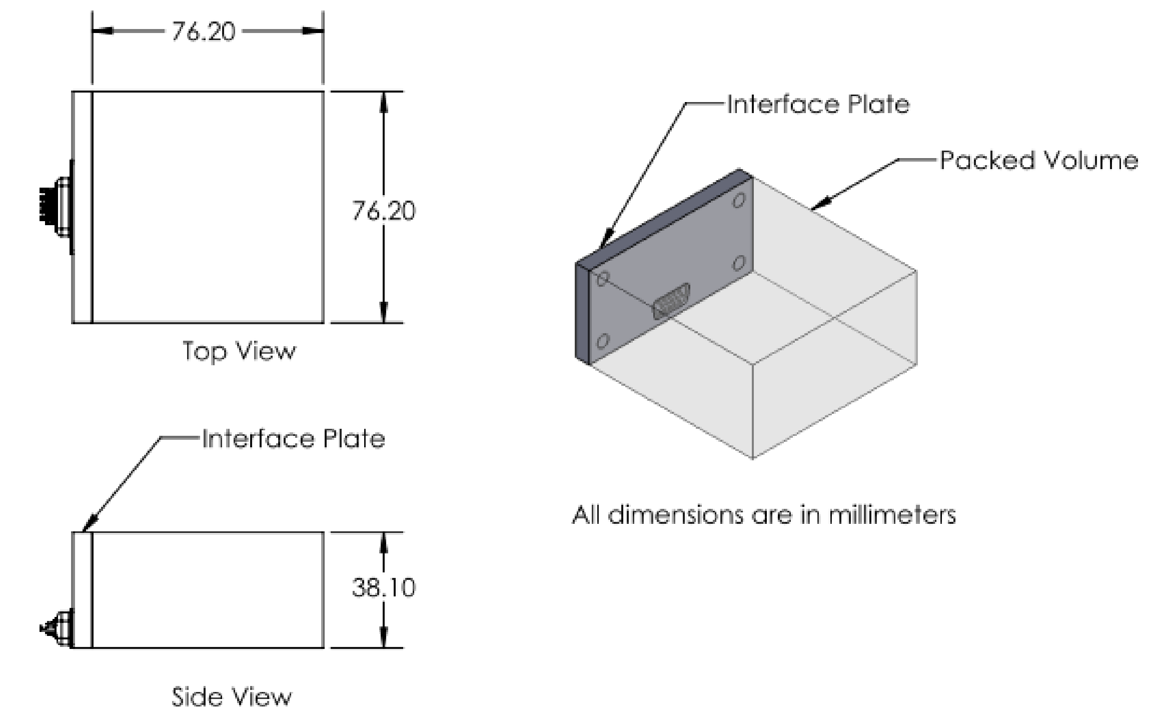


**Figure 2 – Packed Configuration**

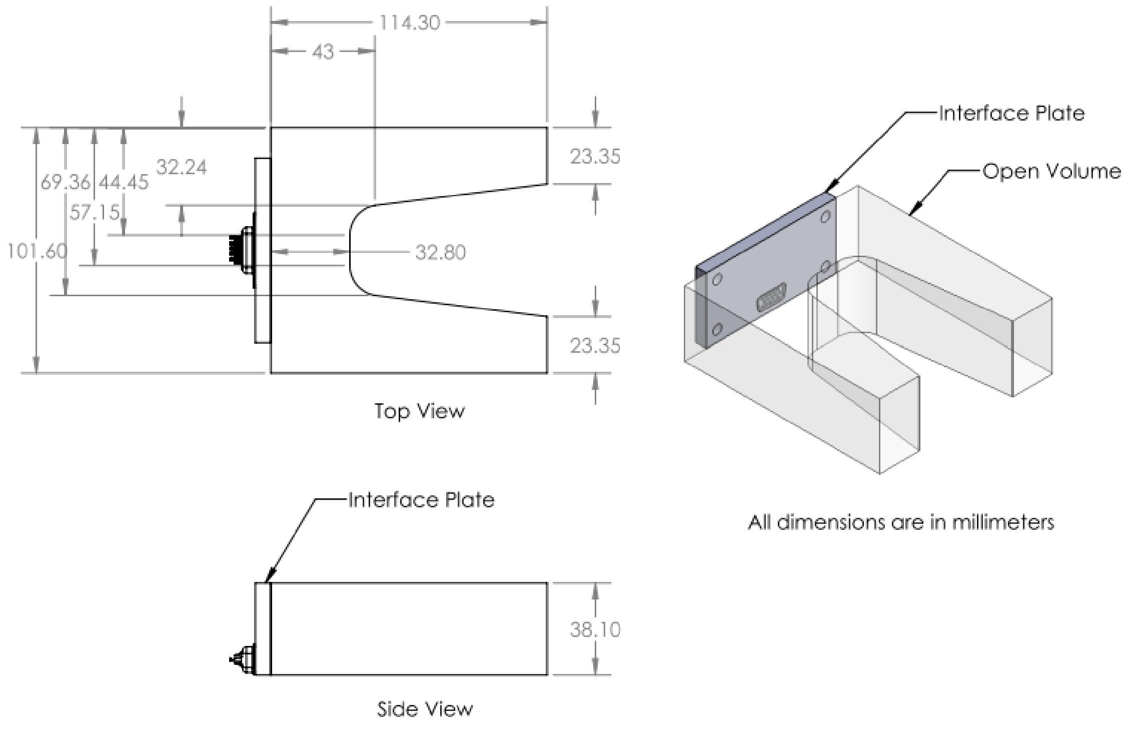


**Figure 3 – Open Configuration**

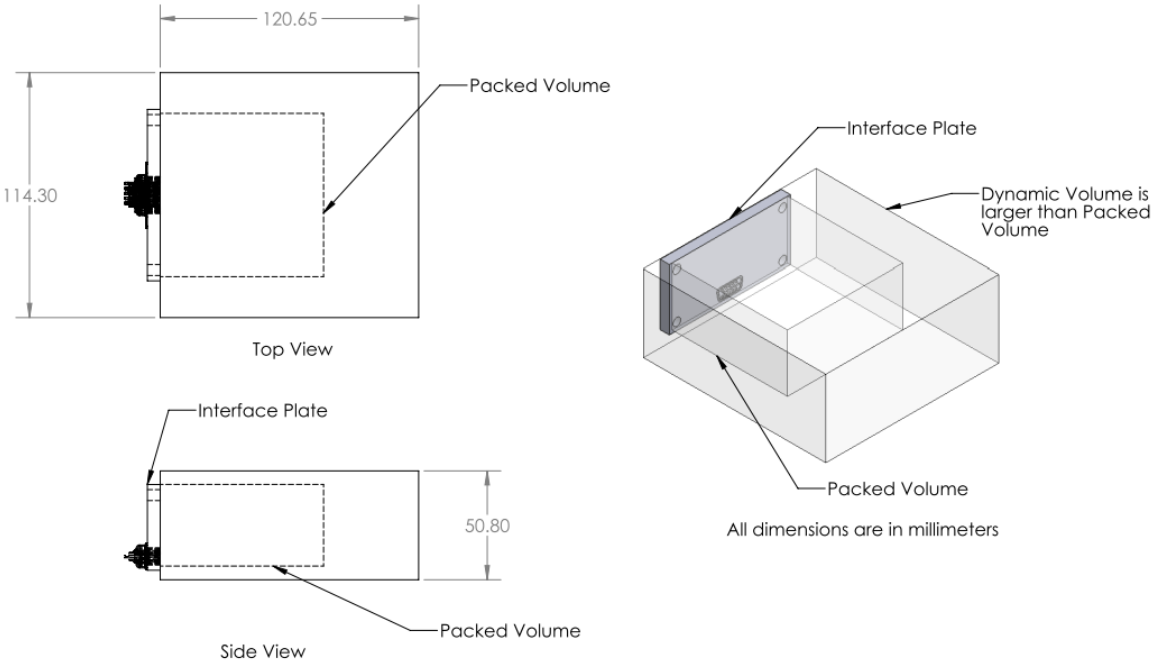


**Figure 4 - Dynamic Envelope**

R2 <u>Closing</u>: The SAM shall be able to move from the *open* configuration (R1.2) to the *attached* configuration without exceeding the dynamic envelope (R1.3).

R2.1 Pre-attach offset: When closing is initiated, the SAM shall not be offset from the center of the ISS Handrail by more than:

- x = 58.7 mm $\pm$ 6.35 mm [2.3" $\pm$ .25"]
- y = 0mm $\pm$ 6.35 mm [0" $\pm$ .25"]
- z = 0 mm $\pm$ 6.35 mm [0" $\pm$ .25"]
- $\theta_x = 0 \pm 5$ degrees
- $\theta_y = 0 \pm 5$ degrees
- $\theta_z = 0 \pm 5$ degrees

The coordinate system for these offsets is shown graphically in Figure 5.

R2.2 The SAM shall only ever be commanded to attach to a standard ISS Handrail as defined C9.

R2.3 Attach configuration: The SAM shall be considered attached when it if fixed to the Handrail Fixed is defined as being able to resist slipping or twisting when subjected to normal operating loads of up to 3.5 Nm [2.6 ft-lbf] about either the Y-axis or Z-axis (ref Figure 5).

R2.4 While attached, the SAM shall not exceed the attached configuration volume defined in Figure 6.

R3 <u>Holding</u>: The SAM shall be able to maintain a rigid attachment (defined in R2.3) for an extended period of time (per R12).

R4 <u>Releasing</u>: The SAM shall be able to move from the *attached* configuration (R2.2) to the *open* configuration (R1.2) without exceeding the *dynamic envelope* (R1.3)

R5 <u>Packing</u>: The SAM shall be able to return to the *packed* configuration (R1.1) from the *open* configuration (R1.2) without exceeding the *dynamic envelope* (R1.3)

R6 <u>Standby</u>: The SAM shall be able to enter a standby mode wherein it uses minimal power but can receive commands. The power requirements are defined in 4.1.1.

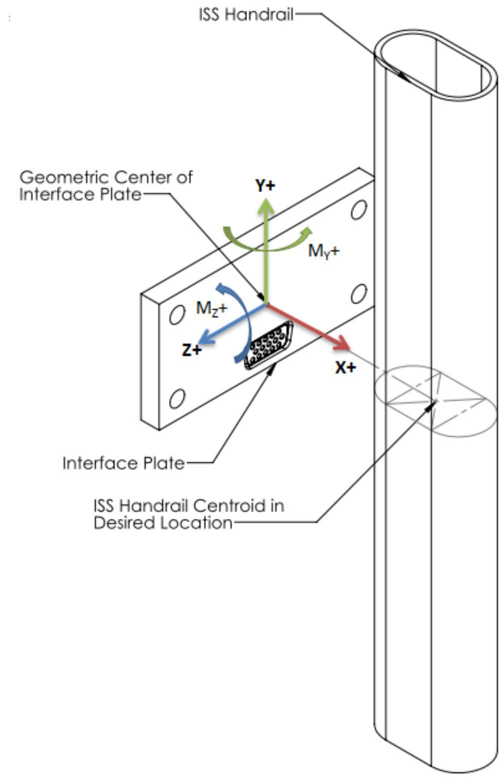


**Figure 5 – SAM Frame of Reference.**

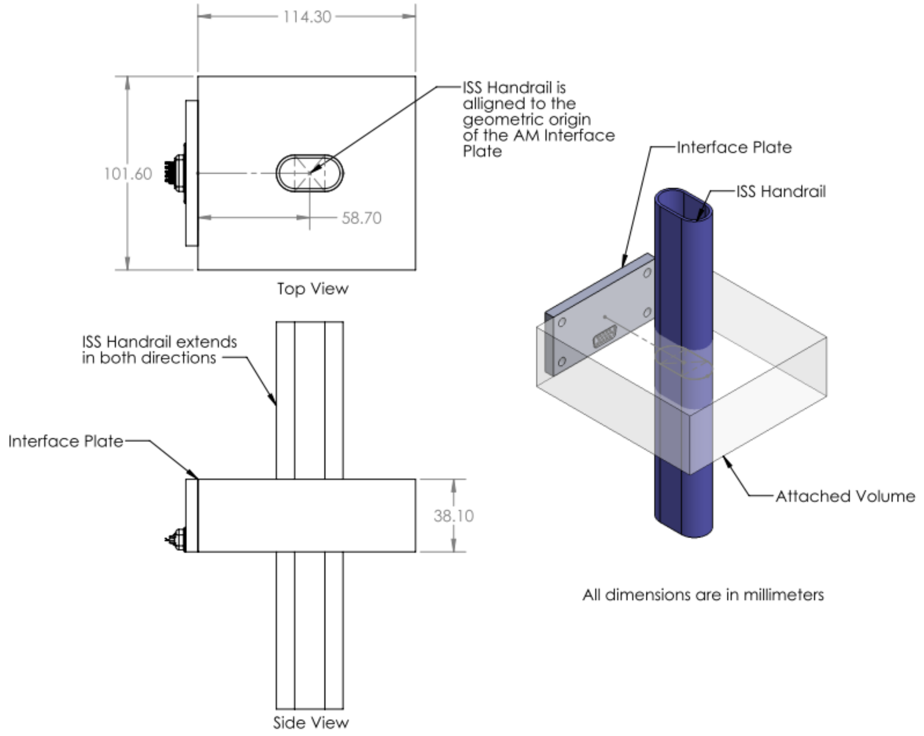


**Figure 6 - Attached configuration**

## 3.2 Control Requirements

The format of all commands is specified in 4.1.2. This section describes how the SAM shall be controlled.

R7 <u>Unpack</u>
- R7.1 When commanded to "unpack", the SAM shall autonomously unpack (R1).
- R7.2 When unpacking is complete, the SAM shall send an "unpackconfirmed" signal to the robotic arm.
- R7.3 When unpacking is complete, the SAM shall enter standby mode (R6).

R8 <u>Close</u>
- R8.1 When commanded to "close", the SAM shall autonomously close (R2).
- R8.2 When closing is complete, the SAM shall send a "closeconfirmed" signal to the robotic arm.
- R8.3 When closing is complete, the SAM shall enter hold mode (R3).

R9 <u>Release</u>
- R9.1 When commanded to "release", the SAM shall autonomously release (R4).
- R9.2 When releasing is complete, the SAM shall send a "releaseconfirmed" signal to the robotic arm.
- R9.3 When releasing is complete, the SAM shall enter standby mode (R6).

R10 <u>Pack</u>
- R10.1 When commanded to "pack", the SAM shall autonomously pack (R5).
- R10.2 When packing is complete, the SAM shall send a "packconfirmed" signal to the robotic arm.
- R10.3 When packing is complete, the SAM shall enter standby mode (R6).

## 3.3 Resource Requirements

### *3.3.1 Timing Requirements*

R11 Time to <u>Unpack</u> and <u>Attach</u>:
- R11.1 Combined, the unpack and close operations shall not exceed 2 minutes.
- R11.2 The timing is measured based on the initiation and confirmation command structure specified in R7 and R8.

R12 Time in <u>Hold</u>: The SAM shall not be closed on the Handrail for more than 1hr. The timing is measured from when close completes and initiates hold (R8.2) and a release command is received (R9).

R13 Time to <u>Release</u> and <u>Pack</u>:
- R13.1 Combined, the releasing and packing operations shall not exceed 2 minutes.
- R13.2 The timing is measured based on the initiation and confirmation command structure specified in R9 and R10.

R14 Time in Standby: Time in standby shall not exceed 26 minutes.

### *3.3.2 Power Requirements*

All power is transmitted through the connector described in interface constraint 4.1.1.

R15 Energy Budget: The SAM shall not use more than 12 Watt-hours across all operations (R1-R6). Assume 1 hr 26 minutes of passive operations (during standby and hold). The time to complete active operations (unpack, close, release and pack) is a feature of your design.

## 3.4 Safety Requirements

R16 The SAM shall have no sharp edges, defined as a radius of 3 mm [.11”], for astronaut safety.
R17 The SAM shall have no loops of material greater than 25.4 mm [1”] in diameter for astronaut safety.
R18 The SAM shall not damage itself through normal operations.
R19 The SAM shall be able to return to its normal operations if power is momentarily lost.

## 3.5 Environmental Requirements

R20 The SAM shall operate in the ISS zero gravity environment.
R21 The SAM, when unpowered, shall not be damaged by electrostatic discharge <4,000V.
R22 The SAM shall operate in an atmosphere comparable to that of Earth. Assume 21 degrees centigrade [70 degrees Fahrenheit], with low humidity, and pressurized to 100 kPA [750 mm Hg, 14.5 psi].
R23 The SAM shall not contribute any particulates (e.g. dust) to the ISS atmosphere.
R24 The SAM shall enclose all lubricated components to prevent lubricants from leaking into the atmosphere of the ISS.

## 3.6 Contingency Requirements

R25 No Handrail: In some cases, a command to complete the close operation may be sent to the SAM, but there is no Handrail at the specified location. The SAM need not recognize this type of error, but shall also not damage itself while trying to complete the operation.
R26 Contingency loads cases. This scenario may occur if an astronaut or piece of equipment contacts Astrobee while attached (including while experiencing normal operating loads per R2.3). The SAM shall break away from the Handrail if it experiences a force of greater than 18N [4 lbf] applied at the SAM interface in the negative Y-direction and a simultaneous moment of 5Nm [3.7 ft-lbs] about the positive Z-axis as seen in Figure 7.
R27 Astronaut intervention: The SAM shall be removable from the Handrail by an astronaut. Assume an astronaut can apply a pull-away force of 35.6 N [8 lbf] in the negative X-direction as seen in Figure 7. This will not occur during any other operation.

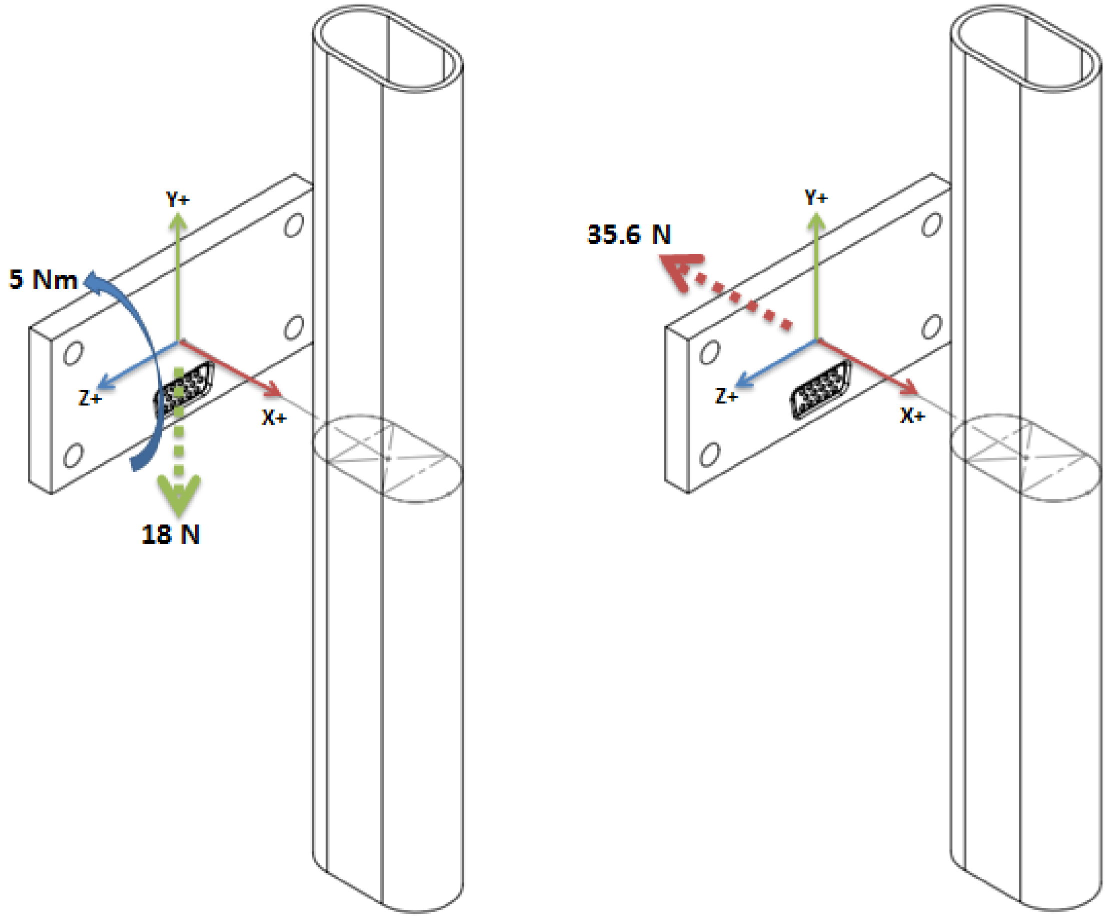


Figure 7 - Off-nominal loads: a) Contingency loads; b) Astronaut intervention loads

# 4 Interface Requirements

The SAM has a fixed interface to a separately designed robotic arm and a dynamic interaction with ISS Handrails. The section describes all constraints imposed by those interfaces.

## 4.1 SAM-Robotic Arm Interface

C1 Constraint 1 (C1) Mounting Interface: The SAM shall mount to the interface plate shown in Figure 8. There are four available screw holes in the specified locations.

- C1.1 All external loads are applied at the interface plate.
- C1.2 Screws for your selected electrical connectors must only require hand tightening of locking screws. Assume that no external loads are applied through the electrical connector.

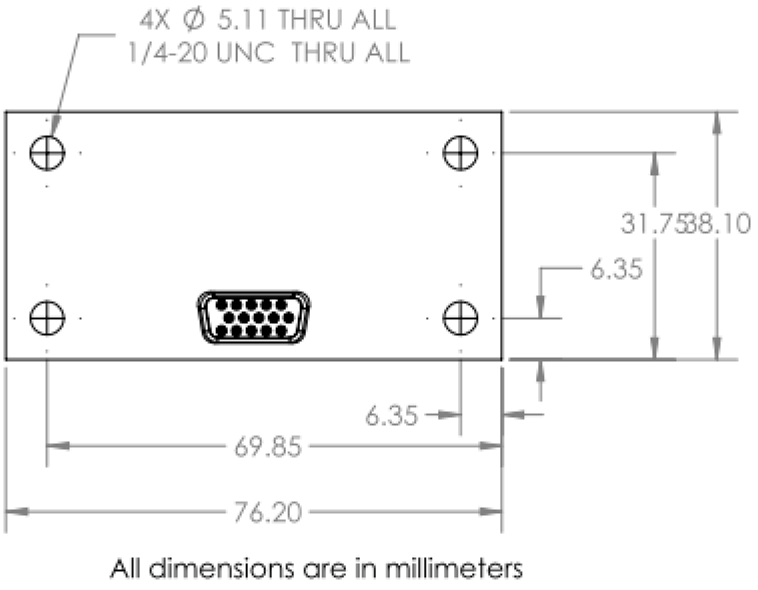


Figure 8 - SAM-Robotic Arm Mounting Interface

### *4.1.1 Power Interface:*

C2 The SAM shall connect to the power and data connector shown in Figure 9. It is a standard D-Subminiature High Density 15pin connector (DSUB-15).

C3 Astrobee Bus Voltage+ is nominally 14.4 volts DC, but can vary between 11 to 17 volts DC.

C4 Max current: The SAM shall not draw more than 3 Amps.

C5 Steady State Current: The SAM shall not draw more than 2 Amps at steady state.

C6 Pin out is as follows: Pin 4 is bus voltage. Pin 1 is ground.

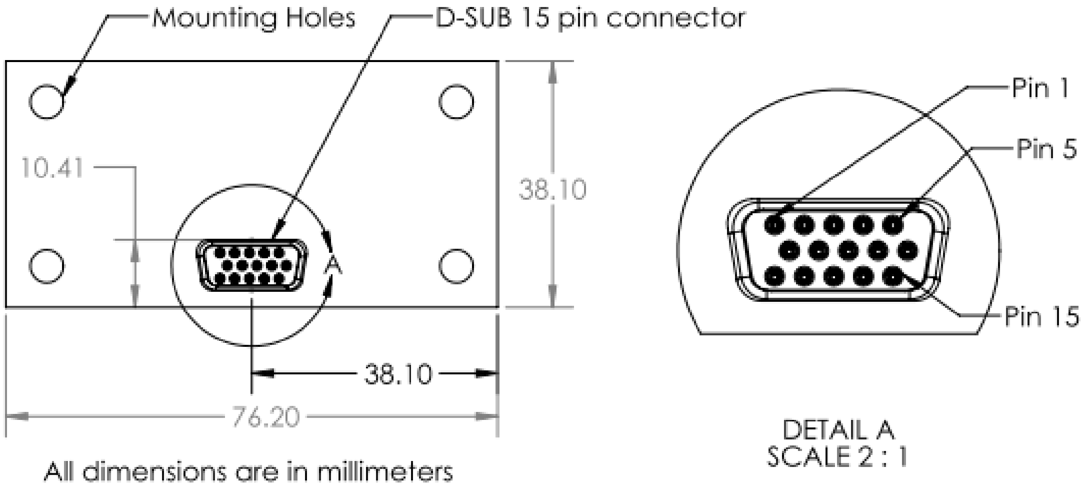


Figure 9 - Power/data connector location

### *4.1.2 Data Interface:*

C7 All commands will be received and transmitted as part of a serial command using the second and third pins. The second pin is the positive serial command line, and the third pin is the negative serial command line.

C8 Commands (received and transmitted) shall be serial and formatted in ASCII using the RS-232 protocol once. They are specified in Table 1.

**Table 1 – Command format**

| Command format | Action |
|---|---|
| Sent from robotic arm to SAM | |
| "unpack" | Initiate unpack (R7.1) |
| "close" | Initiate close (R8.1) |
| "release" | Initiate releasing (R9.1) |
| "pack" | Initiate pack (R9.1) |
| Sent from SAM to robotic arm | |
| "unpackconfirmed" | Confirm unpack has completed (R7.2) |
| "closeconfirmed" | Confirm close has completed (R8.2) |
| "releaseconfirmed" | Confirm release has completed (R9.2) |
| "packconfirmed" | Confirm unpack has completed (R10.2) |

## 4.2 Handrail Interface

C9 Handrail definition:

C9.1 The shape of a standard Handrail is defined in Figure 10.

C9.2 The ISS Handrail is made of anodized aluminum. Assume the material is 6061 Aluminum of type T4 in terms of material properties and friction properties.

C9.3 The ISS Handrail is a 1.59mm [1/16"] thick aluminum 6061 extrusion.

C10 The Handrail shall not be damaged during operations through excessive force (per R2.3, R22 and R23). Damage includes, but is not limited to: crushing, denting, or bending.

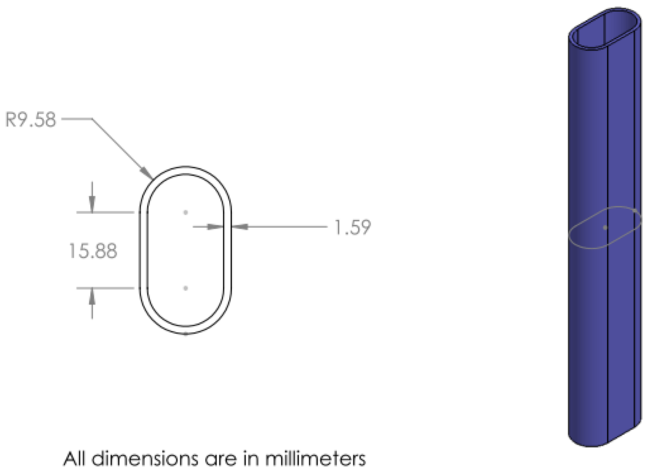


**Figure 10 - ISS Handrail Definition**
