# Supplementary material for: Linking solver characteristics, solving processes and solution attributes: A data explainer for an open innovation generated robotic design dataset
Source: Data Brief. 2023 Sep 6;50:109547. doi: 10.1016/j.dib.2023.109547 (PMC10518673; doi:10.1016/j.dib.2023.109547)
Supplement: Supplementary file 1 [file mmc1.zip › Release/Process/Challenge Rules/D5-EBD/EBDProblemDescription_SubmissionGuidelines_rev2.pdf]

# 1 Contest Description

In this contest, we need your help in designing a lightweight electronics box to accommodate three individual electronics boards. Your design should also accommodate two electrical connectors and a mechanical mounting bolt pattern.

***Challenge Rules***: A prize will be awarded for the lowest mass, technically feasible design.

Specific examples of the boards and connectors we'd like your box to house are identified in the table below:

| | |
|---|---|
| Boards # 1 & 2 | Faulhauber Control Board, MC 5004 P RS/CO: https://www.faulhaber.com/en/products/series/mc-5004-p/ |
| Board #3 | Arduino Mega 2560: https://cdn-shop.adafruit.com/datasheets/arduino_hole_dimensions.pdf |
| External Connector #1 | Glenair 9-socket micro-D connector: Glenair M83513/02-AN |
| External Connector #2 | Glenair 31-socket micro-D connector: Glenair M83513/01-EN |

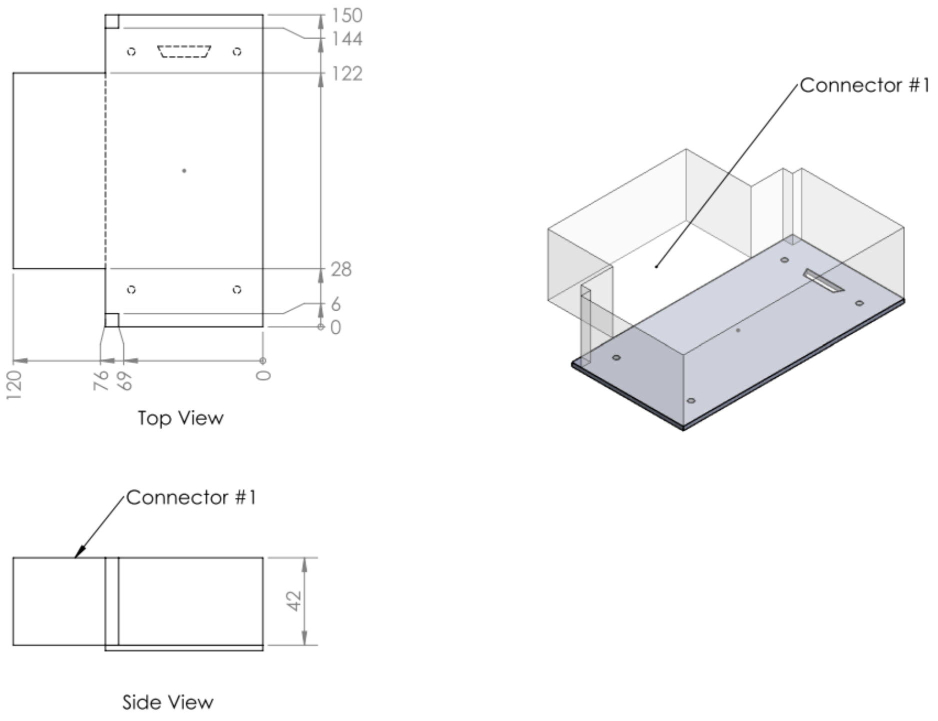


**Figure 1 - Available volume for electronics box (all dimensions are in mm)**

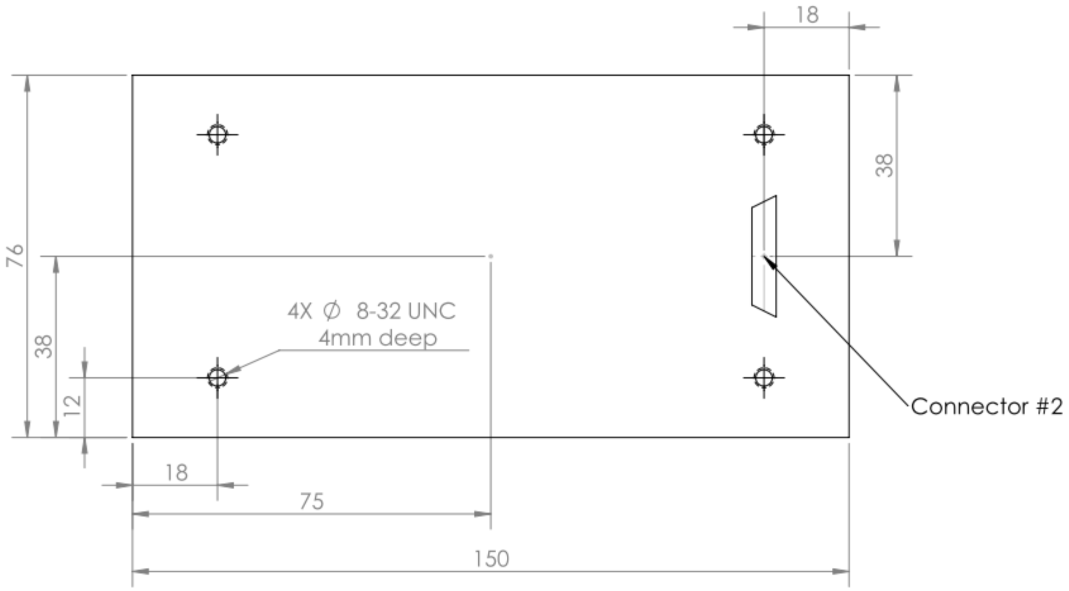


**Figure 2 – Mechanical mounting bolt pattern (all dimensions are in mm)**

- Your box design should not exceed the volume shown in Figure 1.
- Figure 1 identifies the side of the volume where Connector #1 should be. You may choose the specific location for Connector #1 anywhere on this side of the volume as long as the connector receptacle can be accessed from the surface identified in Figure 1.
- The specific bolt pattern for mounting your box design is shown in Figure 2.
- Figure 2 also identifies the desired location for Connector #2.
- The individual boards should not contact each other and should be 10 mm from external connectors that are on box surfaces.
- You should assume the box would be made out of Aluminum.

# 2 Submission Guidelines

1. ***Design Description:*** How does your design meet the goals described above? Drawings, CAD, photos are all acceptable ways of describing your electronics box design.

2. ***Design Volume:*** Show how your design fits within Volume in Figure 1.

3. ***Mass Estimation:*** For your EBD solution, provide a mass estimate. Please explain how you arrived at your estimate and be sure to include all assumptions you have made. The credibility of your estimate depends mainly on the information you provide here.

4. ***Exit Survey:*** To complete your submission, please take the Exit Survey by going to this webpage:

   https://seasgwu.qualtrics.com/jfe/form/SV_2r9DaeSlh48uMcZ

   At the end of the survey you will receive a unique code. In your submission, include this text:

   Exit Survey for Freelancer <<*insert Freelancer username*>> complete with completion code: <<*insert completion code*>>.
