# Supplementary material for: Linking solver characteristics, solving processes and solution attributes: A data explainer for an open innovation generated robotic design dataset
Source: Data Brief. 2023 Sep 6;50:109547. doi: 10.1016/j.dib.2023.109547 (PMC10518673; doi:10.1016/j.dib.2023.109547)
Supplement: Supplementary file 1 [file mmc1.zip › Release/Process/Challenge Rules/D1-SRA/SRA Blurb.pdf]

Smart Robotic Arm (SRA)

In this contest, you are asked to design a Smart Robotic Arm (SRA) that will be mounted to Astrobee and can attach to an ISS Handrail after being stowed in a Payload Bay.

How it works: Initially, the SRA will be packed in a stowed configuration. When it is powered and commanded by the Astrobee, the SRA must be able to autonomously perform four high-level actions: 1) unpacking from the stowed configuration and attaching to a Handrail, 2) moving the Astrobee side to side, "panning", 3) moving the Astrobee up and down, "tilting" and 4) detaching from the Handrail and (re-)stowing in Astrobee's payload bay

Click on the links below to see detailed design instructions, constraints and submission templates for this problem.

Challenge Rules: A prize of **$5,000** will be awarded for the **lowest mass, technically feasible** solution submitted by October 5nd, 2018. No working prototype is required for submission, but the design must be sufficiently detailed to allow experts to assess the feasibility of your design (i.e., comply with all requirements) and the credibility of your mass estimate. Only complete submission packages will be evaluated.

Attachments:

SRA_ProblemDescription.pdf

SRA_SubmissionGuidelines.pdf

Templates:

SRAMassTemplate [.xlsx .ods]

SRAPowerTemplate [.xlsx .ods]
