# Supplementary material for: Linking solver characteristics, solving processes and solution attributes: A data explainer for an open innovation generated robotic design dataset
Source: Data Brief. 2023 Sep 6;50:109547. doi: 10.1016/j.dib.2023.109547 (PMC10518673; doi:10.1016/j.dib.2023.109547)
Supplement: Supplementary file 1 [file mmc1.zip › Release/Process/Challenge Rules/D1-SRA/SRA Problem Description.pdf]

1 Contest Description

In this contest, you are asked to design a "Smart" Robotic Arm (SRA) that mounts directly to Astrobee through the Astrobee Interface Plate.

The SRA receives all power and high-level commands from Astrobee, but implements the following functions autonomously: stowing and deploying from a compact payload volume, attaching to and releasing from an International Space Station (ISS) Handrail, and orienting Astrobee by rotating in two directions (pan and tilt).

The details below describe how the SRA should work, its functional requirements and interface constraints/assumptions. A separate document provides detailed guidelines on how your design must be presented and submitted.

A prize of $5000 will be awarded for the lowest mass, technically feasible solution, submitted before 21:00 GMT on October 5th, 2018.

2 Concept of Operations – How the SRA needs to work

2.1 Normal Operations

The SRA must be able to autonomously perform four high-level operations when it receives a corresponding command from Astrobee: <u>attach</u> securely to an ISS Handrail from a stowed configuration in Astrobee's Payload Bay, orient (<u>pan</u> and <u>tilt</u>), and return to <u>stow</u> into the Astrobee Payload Bay. For reference, Figure 1 shows Astrobee with an open Payload Bay hovering near a Handrail and illustrates the coordinate systems. The requirements for each operation are detailed in Section 3.

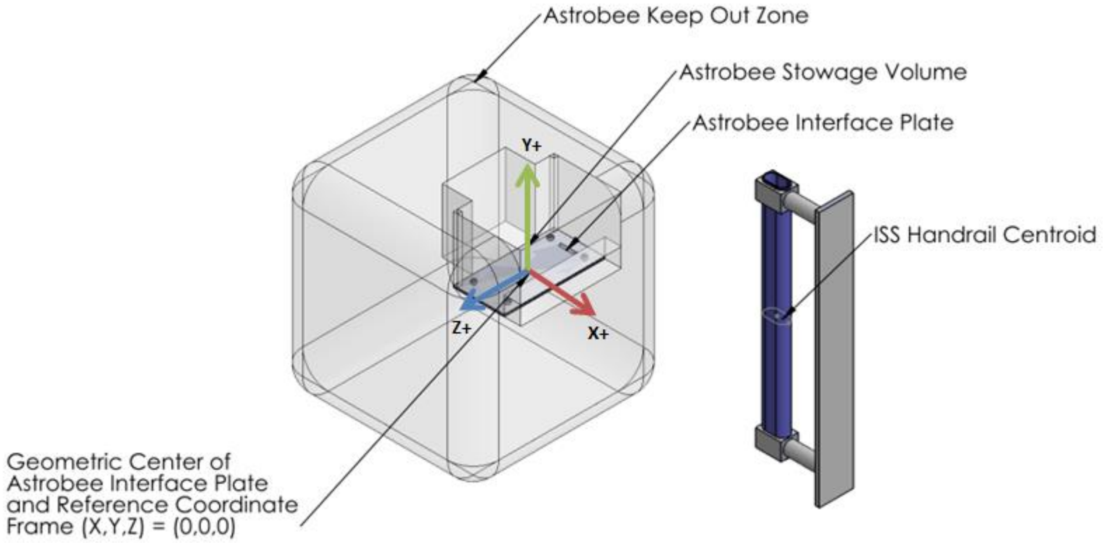

Figure 1 –Astrobee near an ISS Handrail

2.2 Contingency (Emergency) Operations

There are three scenarios when normal operations may be disrupted. Acceptable responses to these scenarios are detailed in section 3.6. This section summarizes those scenarios: (1) when the SRA experiences higher than expected loads while orienting and attached (e.g., because an astronaut or other object bumps or contacts Astrobee); (2) when an astronaut manually pulls on the SRA to remove the system from the ISS Handrail; and (3) when the SRA attempts to close on an ISS Handrail and no ISS Handrail is present.

3 Functional Requirements

This section details all of the requirements that the SRA must meet. To clearly define the motion involved in these operations, we define a coordinate reference frame that has its origin at the geometric center of the Astrobee Interface Plate (see Figure 1).

3.1 Motion Requirements

R1 Attach: The SRA shall be able to move from a *stowed* configuration (inside Astrobee's Payload Bay – see C1) and attach to an ISS Handrail that could be located anywhere in the ISS Handrail workspace defined below

R1.1 ISS Handrail Workspace: The SRA's "workspace" is defined, in Cartesian coordinates, by the centroid of the ISS Handrail location (x, y, z), such that:

- $265 \text{ mm} \leq x \leq 315 \text{ mm}$
- $-25 \text{ mm} \leq y \leq 125 \text{ mm}$
- $-100 \text{ mm} \leq z \leq 100 \text{ mm}$

With the origin (0,0,0) of this coordinate system at the center of the Astrobee Interface Plate (see C2). Figure 2 illustrates the workspace and reference coordinate system.

R1.2 ISS Handrail Orientation: You can assume that the ISS Handrail cross-section will remain parallel to the bottom of the Astrobee Payload Bay, as illustrated in Figure 1.

R1.3 *Attached* Configuration: The SRA shall be considered attached when it is fixed to the ISS Handrail. Fixed is defined as being able to resist slipping or twisting when subjected to normal operating loads of up to 3.5 Nm about either the Y-axis or Z-axis (see Figure 3).

R1.4 Collision Avoidance: The SRA shall not contact Astrobee, including its Astrobee Payload Bay walls, during any operations. Detailed definition of Astrobee dimensions is in C1.

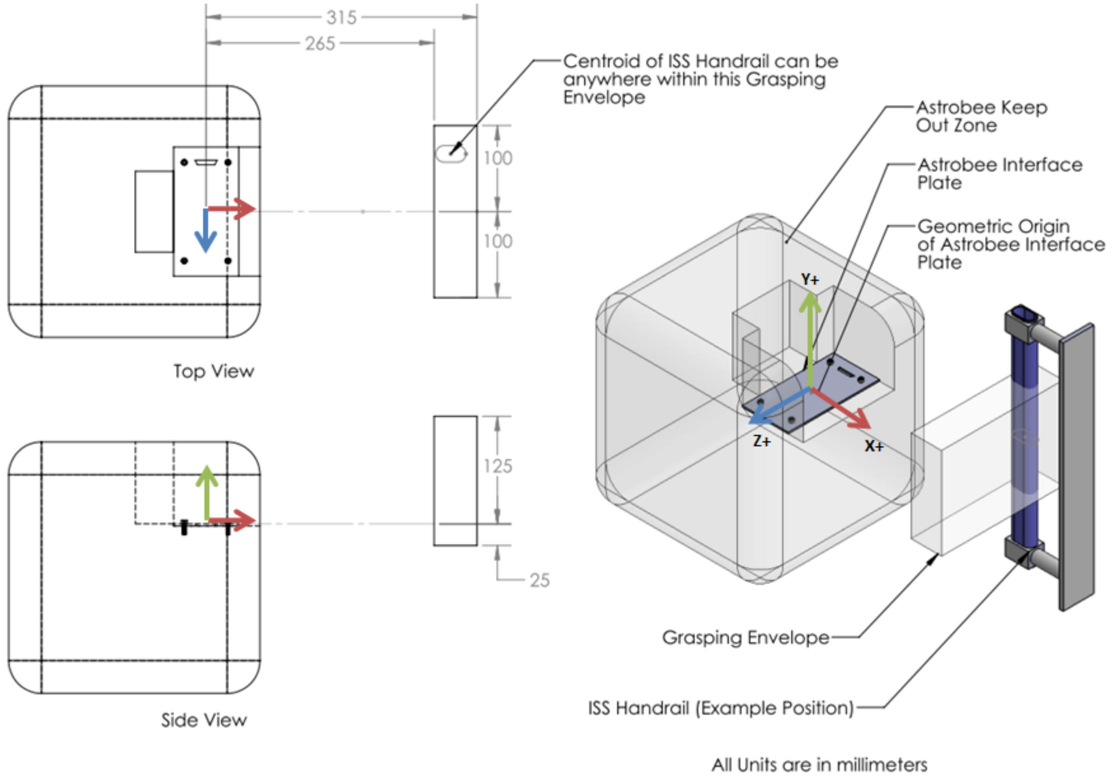

Figure 2 - ISS Handrail Workspace

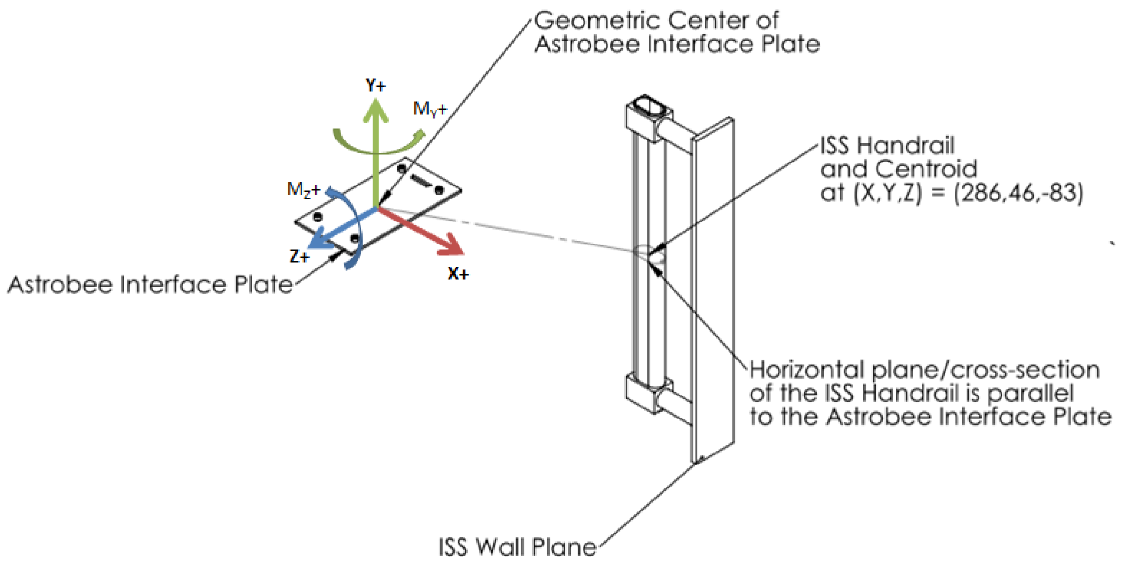

Figure 3 – SRA Frame of Reference.

R2 Orient: When *attached* to a ISS Handrail and commanded by Astrobee, the SRA shall be able to <u>pan</u> and <u>tilt</u> (rotate in two perpendicular directions) Astrobee about the ISS Handrail

R2.1 Center of Rotation: Center of rotation for <u>pan</u> and <u>tilt</u> must be within 130 mm of the ISS Handrail Centroid. The selection of the exact position for the Pan/Tilt center of rotation is part of your SRA design. An illustration of what this could look like is shown in Figure 4. The pan and tilt measurements are absolute movements based on the point of rotation you choose, but must be oriented in the same way as seen in Figure 4.

R2.2 Clearance: Minimum clearance between the moving components of the SRA, and the Astrobee Keep Out Zone during these movements is 2 mm

R2.3 Pan Range of Motion and Accuracy: The SRA shall be able to pan through the range: -65° < θ_x < 65° , +/- 5° as detailed in Figure 4

R2.4 Tilt Range of Motion and Accuracy: the SRA shall be able to tilt through the range: 0° < θ_y < 90° , +/- 5° as detailed in Figure 4

R2.5 Single Movements: No simultaneous panning and tilting shall ever be requested.

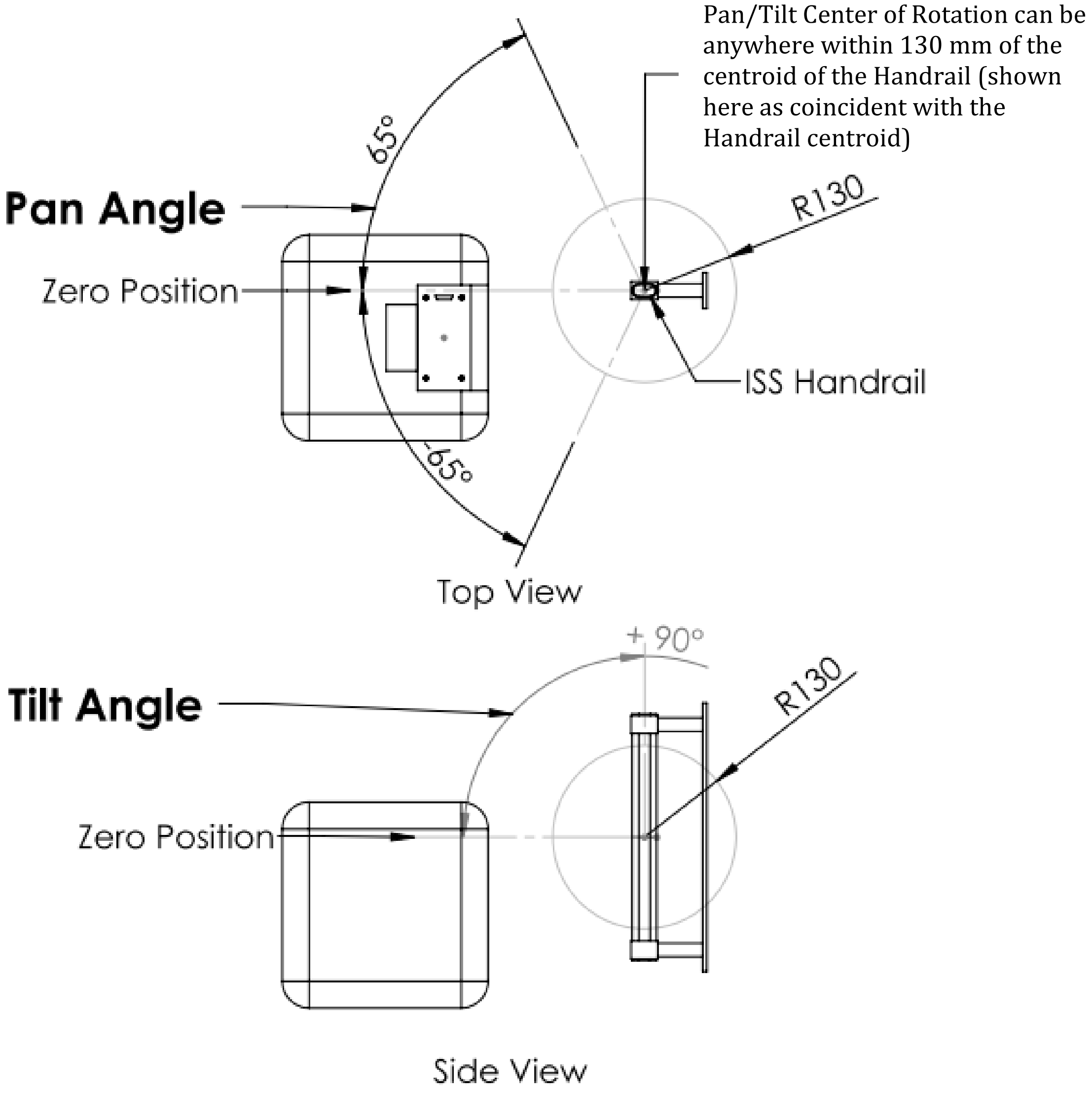

Figure 4 - Pan & Tilt Center of Rotation and Ranges of Motion

R3 Stow: The SRA shall be able to detach from the ISS Handrail and maneuver to return to its *stowed* configuration inside Astrobee's Payload Bay – see C1).

Stowed Configuration*:* All components of the SRA must fit within the Astrobee Payload Bay in the s*towed* configuration.

3.2 Control Requirements

The format of all commands is specified in C10. This section describes how the SRA shall be controlled.

R4 When commanded to "attach(x,y,z)",
1. The SRA shall autonomously attach (R1) to the ISS Handrail location at X,Y,Z.
2. When attaching is complete (R1.3), The SRA shall send an "attachconfirmed" signal to Astrobee (C10)

R5 When commanded to "pan(θ_x)",
1. The SRA shall complete the specified pan rotation (R2), θ_X
2. When panning is complete, the SRA shall send a "panconfirmed" signal to Astrobee (C10)

R6 When commanded to "tilt(θ_y)"
1. The SRA shall complete the specified tilt rotation (R2), θ_y
2. When tilting is complete, the SRA shall send a "tiltconfirmed" signal to Astrobee (C10)

R7 When commanded to "stow"
1. The SRA shall autonomously stow in Astrobee's Payload Bay (R3)
2. When tilting is complete, the SRA shall send a "stowconfirmed" signal to Astrobee (C10)

3.3 Resource Requirements

3.3.1 Timing Requirements

R8 Time to Attach: Attach operations shall not exceed 15 minutes. The timing is measured based on the SRA receiving the "attach(x,y,z)" command to the "attachconfirmation" signal being sent.

R9 Time to Orient:

R9.1 The SRA shall be able to pan 90 degrees in 15 seconds.

R9.2 The SRA shall be able to tilt 90 degrees in 15 seconds.

R9.3 Combined pan and tilt operations shall not exceed 1 hour.

R10 Time to Stow: Stow operations shall not exceed 15 minutes. The timing is measured based on the SRA receiving the "stow" command to the "stowconfirmation" signal being sent.

3.3.2 Power Requirements

All power is transmitted through the connector described in interface constraint 4.1.2.

R11 Energy Budget: The SRA shall not use more than 42 Watt-hours across all operations. In calculating your energy budget, assume that pan and tilt are ongoing for 1 hour.

3.4 Safety Requirements

R12 The SRA shall have no sharp edges, defined as a radius of 3 mm, for astronaut safety.

R13 The SRA shall have no loops of material greater than 25.4 mm in diameter for astronaut safety and unsupported or unattached for more than 40 mm from the structure of the SRA.

R14 The SRA shall not damage itself through normal operations.

R15 The SRA shall be able to return to its normal operations if power is momentarily lost.

3.5 Environmental Requirements

R16 The SRA shall operate in the ISS zero gravity environment.

R17 The SRA, when unpowered, shall not be damaged by electrostatic discharge <4,000V.

R18 The SRA shall operate in an atmosphere comparable to that of Earth. Assume temperature of 21 °C [70 °F], and pressure of 101 kPa [1 atm], and relative humidity that is 40% - 70%.

R19 The SRA shall not contribute any particulates (e.g. dust) to the ISS atmosphere.

R20 The SRA shall enclose all lubricated components to prevent lubricants from leaking into the atmosphere of the ISS.

3.6 Contingency Requirements

R21 Excessive loads: This scenario may occur if an astronaut or piece of equipment contacts Astrobee while the SRA is secured to the ISS Handrail. The SRA shall maintain normal orient operations (R2) when subject to a force of up to 18N applied at the baseplate in the negative Y-direction (see Figure 5a).

R22 Astronaut intervention: Astrobee is required to be removable from the ISS Handrail by an astronaut after the SRA is attached to the ISS Handrail. Assume that for the SRA, this translates to a pull-away force of 35.6 N in the negative X-direction (see Figure 5b) applied through the baseplate.

R23 No Handrail: In some cases, a command to <u>attach</u> may be sent to the SRA, but there is no ISS Handrail at the specified location. The SRA need not recognize this type of error, but shall also not damage itself while trying to complete the operation.

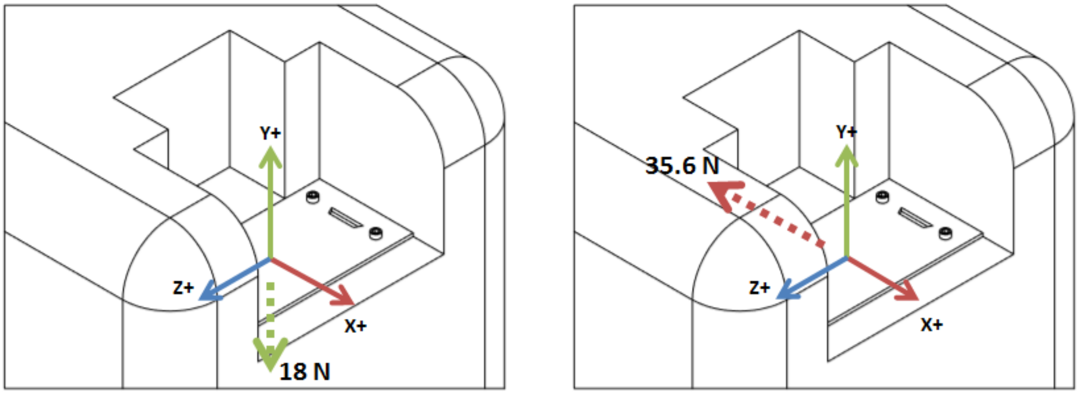

Figure 5 – Contingency Loads a) shows Excessive Loads; b) shows Astronaut intervention loads

4 Interface Requirements

The SRA has a fixed interface to Astrobee and a dynamic interaction with ISS Handrails. The section describes all constraints imposed by those interfaces.

4.1 SRA-Astrobee Interface

4.1.1 Mechanical Interface

C1 Constraint 1 (C1) Volume Constraint: The SRA is stowed in Astrobee's Payload Bay when in the *stowed* configuration. Figure 6 and Figure 7 define the Astrobee Payload Bay with respect to Astrobee.

- C1.1 Figure 6 defines the dimensions of the Astrobee volume that must not be contacted (Astrobee Keep Out Zone)
- C1.2 Figure 7 defines the dimensions of the Astrobee Payload Bay, which the SRA must stow within in the *stowed* configuration.

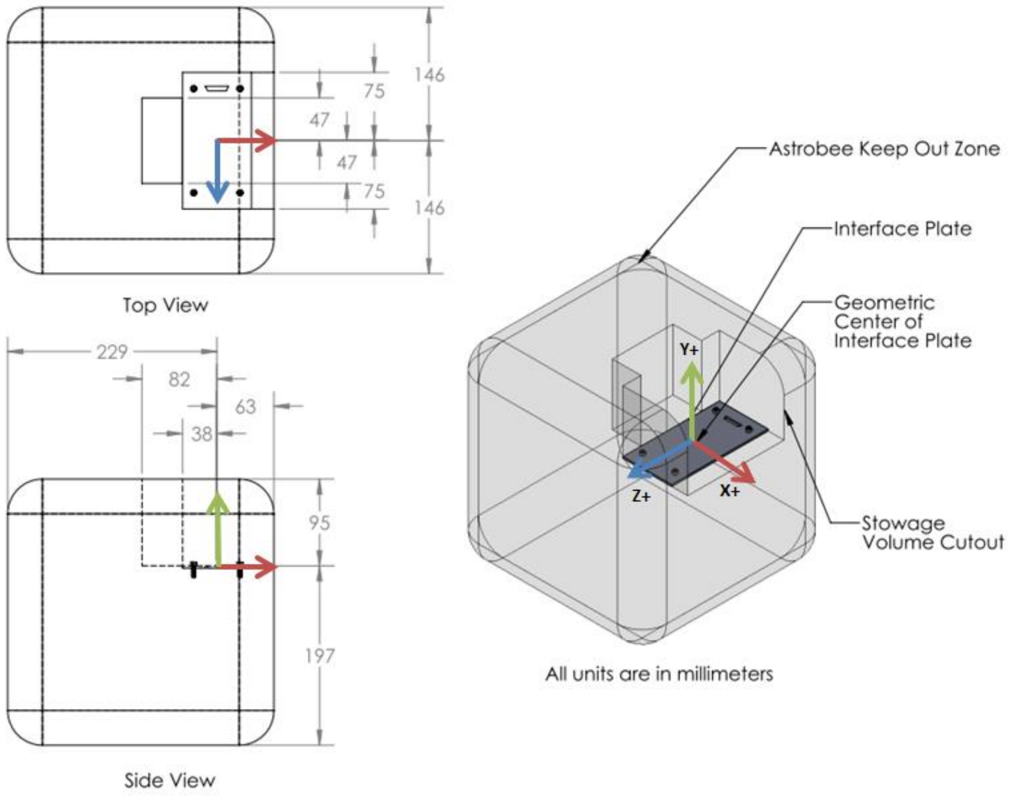

Figure 6 - Astrobee Keep Out Zone and Adapter Plate

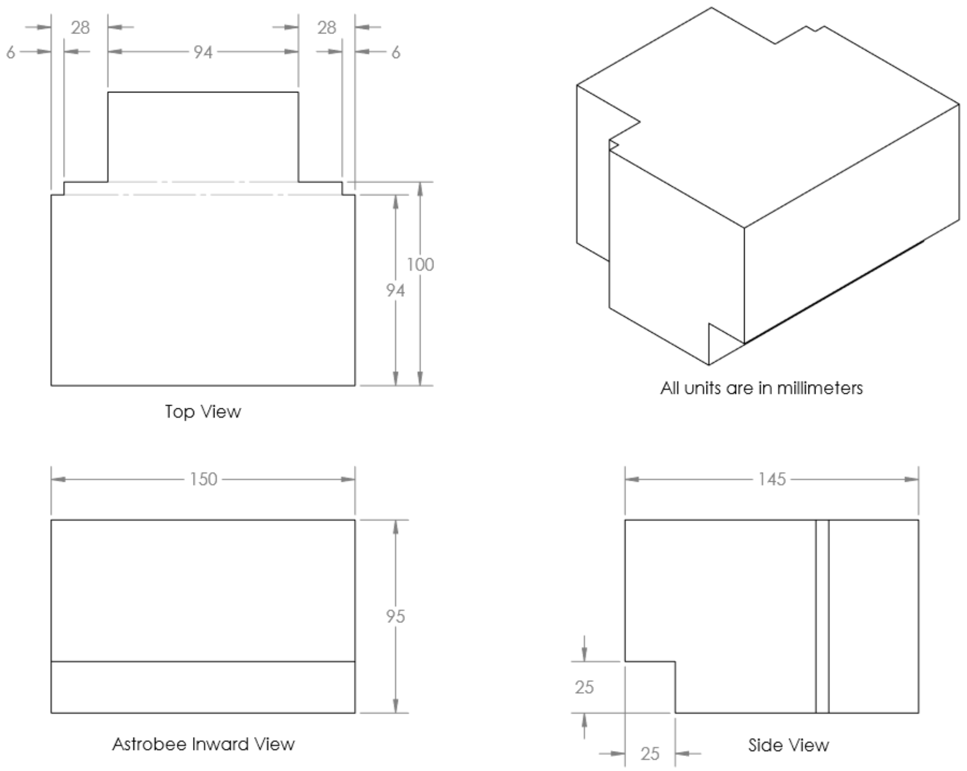

Figure 7 – Astrobee Payload Bay

C2 Astrobee Interface Plate: The SRA shall mechanically mount to a flat metal plate show in Figure 8.

C2.1 All external loads are applied at the Interface Plate.

C2.2 There are four available screw holes in the specified locations.

C2.3 Screws for your selected electrical connectors must only require hand tightening of locking screws of the Interface Plate. Assume that no external loads are applied through the electrical connector.

C3 Mass Properties: Treat Astrobee as mass of 6 kg with center of mass = (-83mm, -48 mm, 0 mm) relative to the SRA Coordinate Reference Frame as seen in Figure 1.

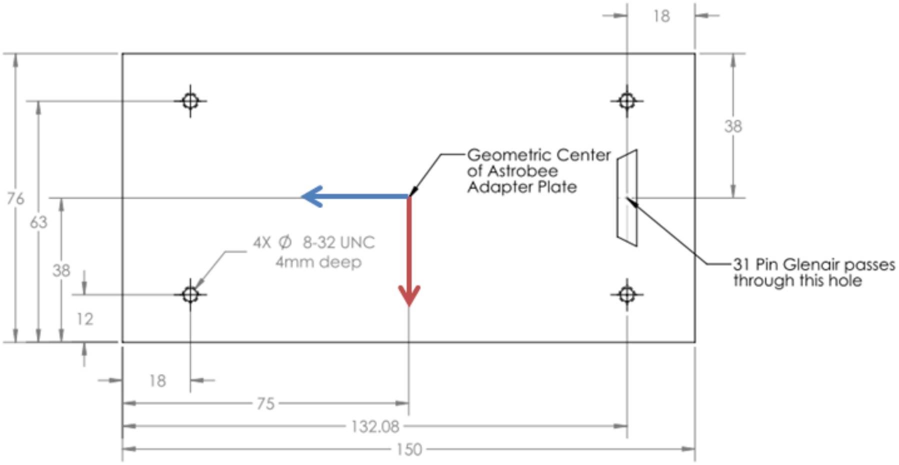

Figure 8 - Astrobee Interface Plate

4.1.2 Power Interface:

C4 The SRA shall connect to the power and data connector shown in Figure 8. It is a 31-pin payload Glenair M83513/03-E03N connector located in the baseplate.

C5 Astrobee Bus Voltage + is normally 14.4 volts DC, but can vary between 11 to 17 volts DC.

C6 Max Current: The SRA shall not draw more than 3 A peak.

C7 Steady State Current: The SRA shall not draw more than 2 A at steady state.

C8 Pinout is as follows: Pin 4 is bus voltage. Pin 1 is ground.

4.1.3 Data Interface:

C9 All commands will be received and transmitted as part of a serial command using the second and third pins of the 31-pin connector. The second pin is the positive serial command line, and the third pin is the negative serial command line.

C10 Commands (received and transmitted) shall be serial and formatted in ASCII using the RS-232 protocol once. They are specified in Table 1.

Table 1 – Command format

Command format	Action
Received from Astrobee to the SRA	
"attach(x,y,z)"	Initiate attach to a Handrail at specified location (R4)
"pan(θ_x)"	Initiate pan for specified degrees (R5)
"tilt(θ_y)"	Initiate tilt for specified degrees (R6)
"stow"	Initiate stow (R7)
Sent from SRA to Astrobee	
"attachconfirmed"	Confirm attach has completed (R4)
"panconfirmed"	Confirm pan has completed (R5)
"tiltconfirmed"	Confirm tilt has completed (R6)
"stowconfirmed"	Confirm stow has completed (R7)

4.2 Handrail Interface

C11 ISS Handrail definition:

- C11.1 The shape of a standard ISS Handrail is defined in Figure 9.
- C11.2 The ISS Handrail is made of anodized aluminum. Assume the material is 6061 Aluminum of type T4 in terms of material properties and friction properties.
- C11.3 The ISS Handrail is a 1.59mm thick aluminum 6061 extrusion.

C12 The ISS Handrail shall not be damaged during operations through excessive force (per R1.3,R21,R22, and R23). Damage includes, but is not limited to: crushing, denting, or bending.

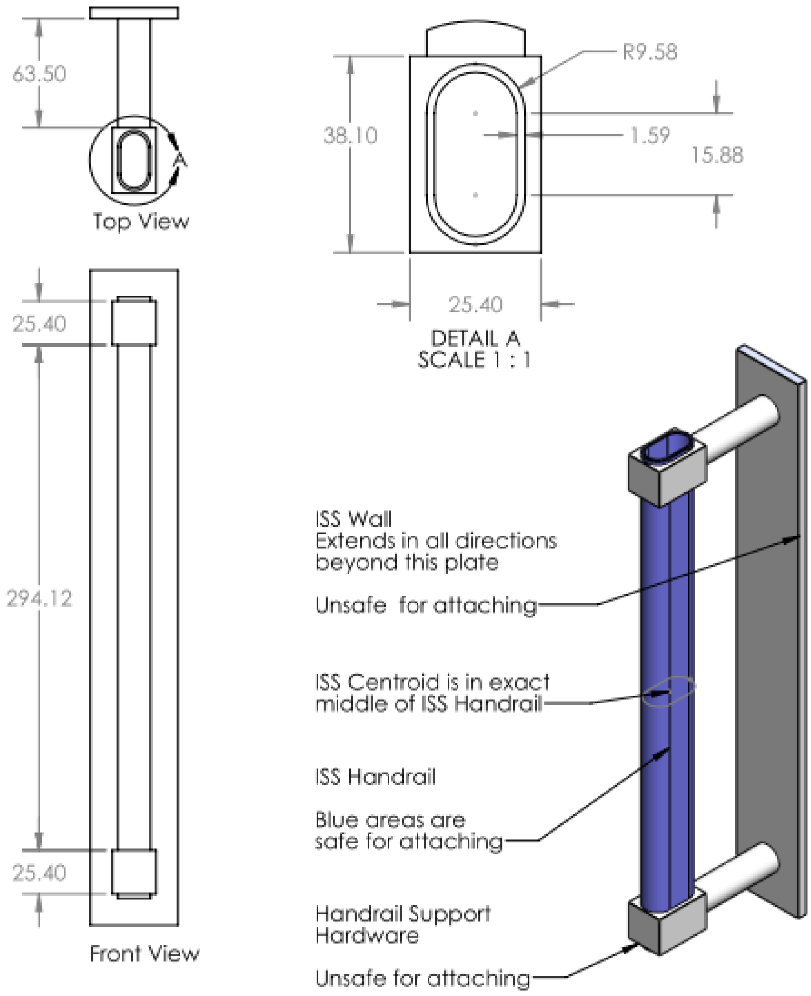

Figure 9- ISS Handrail Definition
