# Supplementary material for: Linking solver characteristics, solving processes and solution attributes: A data explainer for an open innovation generated robotic design dataset
Source: Data Brief. 2023 Sep 6;50:109547. doi: 10.1016/j.dib.2023.109547 (PMC10518673; doi:10.1016/j.dib.2023.109547)
Supplement: Supplementary file 1 [file mmc1.zip › Release/Process/Challenge Rules/D5-BMA/BMAProblemDescription_SubmissionGuidelines.pdf]

1 Contest Description

In this contest, we need your help in improving the design of a box for some electronics hardware. The Box accommodates three internally-mounted components and a bolt pattern for mounting the box itself.

You are asked to create a robust design by analyzing a box for mechanical failure and improving its design to achieve lowest mass possible. You are given a starting-point design, but are free to modify some features and change the Box materials for your custom design. Some details of the starting-point box design are described below in Figure 1 and Table 1, and a solid model file is available for download.

Challenge Rules: A prize will be awarded for the lowest mass, technically feasible design.

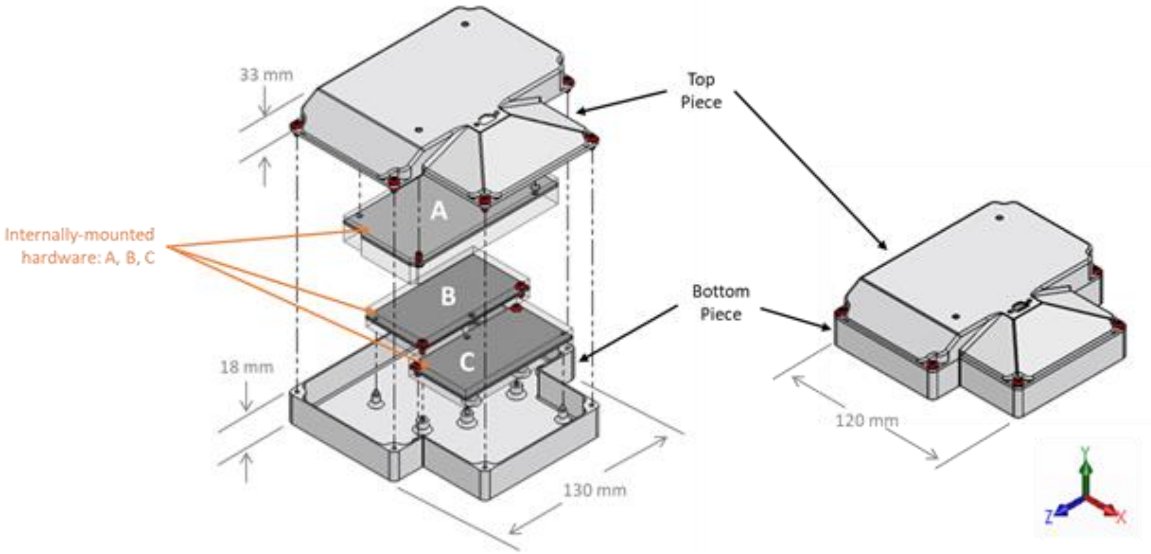

Figure 1: Starting-point Box design snapshot; CAD file provided as attachment

- You may assume that the Box is attached to a relatively large, stainless steel plate through four M4 bolts at corners of the Box Bottom Piece. Precise locations are included in the attached CAD file.

- As you improve the starting-point box design, your design must match the relative location of the bolt pattern on the Bottom Piece for the external mounting and the internally-mounted hardware.

- You may change the dimensions of the starting-point Box design, but should not exceed the original size by more than 5 mm.

- For material properties of the internally-mounted hardware (Boards A, B, C), you may assume 1.6 mm thick G-10 fiberglass epoxy laminate sheet (http://www.matweb.com/search/datasheet_print.aspx?matguid=8337b2d050d44da1b8a9a5e61b0d5f85) with dimensions shown in Figure 2. Additional properties of the three internally-mounted components are included in Table 1.

- You may change the number and type of fasteners for the internally-mounted hardware, but must use existing holes shown in Figure 2.

Table 1: Starting-point Box and component properties

Part Name	Description	Material
Box elements:		
Box - Top Piece	Top of Box - Figure 1 shows "starting point" design	Aluminum
Box - Bottom Piece	Bottom of Box – Figure 1 shows "starting point" design	Aluminum
Box fasteners	Attaches Box Top Piece to Bottom Piece<br>M3 x 6 mm button head cap screws	Stainless steel
Internally-mounted Hardware:		
Board "A"	Mounted to Box Top Piece<br>Figure 2a shows specific dimensions of board<br>Additional mass = 20 g, at center of gravity	G-10 fiberglass epoxy laminate sheet (1.6 mm thick)
Boards "B" & "C"	Mounted to Box Bottom Piece<br>Figure 2b shows specific dimensions of board<br>Additional mass = 12 g, at center of gravity	G-10 fiberglass epoxy laminate sheet (1.6 mm thick)
Board fasteners	M3 x 6 mm button head cap screws	Stainless steel

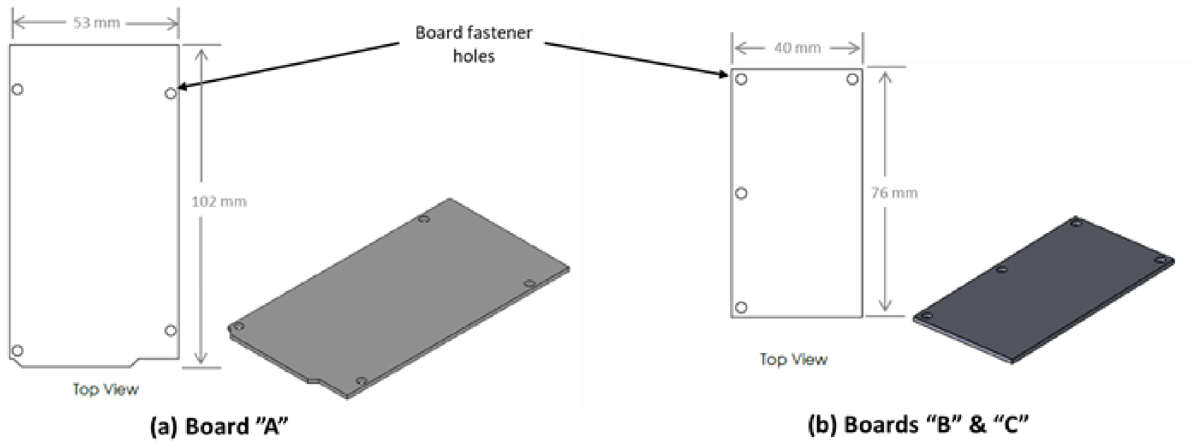

Figure 2: Dimensions of internally-mounted hardware; CAD file attached

- The Box may be subjected to external static loads. Specific levels for loads are identified in Table . You may assume that all loads in Table are applied simultaneously.
    - None of the parts of your box or the internally-mounted hardware should break under these loads.
    - The internally-mounted hardware should not deflect more than 0.5 mm.

Table 2: Externally applied load factors

N_x	N_y	N_z	R_x	R_y	R_z
10 g	5 g	5 g	15 rad/s^2	15 rad/s^2	15 rad/s^2

2 Submission Guidelines

1. ***Design Description:*** What does your Box look like? How does it meet the goals described above? Please make sure to identify all materials used in your Box design, and indicate where they are being used.

   Drawings, CAD, photos are all acceptable ways of describing your final Box design.

2. ***Mechanical Analysis:***
   2.1. Show how your Box design responds to mechanical loads described above. For what kinds of failure did you analyze your Box design? Under what levels of loading does your Box design fail?
   2.2. What is minimum clearance of parts when your box is unloaded and loaded?

   Please make sure to justify your analysis or any numbers you present.

3. ***Mass Estimation:*** For your BMA solution, provide a mass estimate. Please explain how you arrived at your estimate and be sure to include all assumptions you have made. The credibility of your estimate depends mainly on the information you provide here.

4. ***Exit Survey:*** To complete your submission, please take the Exit Survey by going to this webpage:
   https://seasgwu.qualtrics.com/jfe/form/SV_2r9DaeSlh48uMcZ
   At the end of the survey you will receive a unique code. In your submission, include this text:
   Exit Survey for Freelancer <<*insert Freelancer username*>> complete with completion code: <<*insert completion code*>>.
