# Supplementary material for: Linking solver characteristics, solving processes and solution attributes: A data explainer for an open innovation generated robotic design dataset
Source: Data Brief. 2023 Sep 6;50:109547. doi: 10.1016/j.dib.2023.109547 (PMC10518673; doi:10.1016/j.dib.2023.109547)
Supplement: Supplementary file 1 [file mmc1.zip › Release/Process/Challenge Rules/D5-AM/AM Submission Guidelines.pdf]

Submission Guidelines for the Attachment Mechanism

In this contest, you were asked to design an Attachment Mechanism (AM) that will be mounted to the free end of a separately designed robotic arm.

This document provides detailed guidelines on how you must describe and present each aspect of your design in order to be considered for the prize. This document looks long but very little text is required. It's mostly pasting in figures and tables in a structured way. Your submission document must include each of the sections detailed below and all of the information requested in each. Several templates and examples are provided to clarify what constitutes a complete solution.

Use the exact section and subsection header words, shown below.

1 FUNCTIONAL DESCRIPTION 2

1.1 NARRATIVE (WORD) DESCRIPTION OF DESIGN 2

1.2 FUNCTIONAL ANALYSIS 2

2 MASS SUMMARY AND COMPONENT LIST 3

3 SYSTEM LAYOUT 4

4 ELECTRO-MECHANICAL DESIGN 6

4.1 DESIGN DRAWINGS 6

4.2 OPERATING MODES 6

4.3 ACTUATOR DESIGN 7

5 EXIT SURVEY 8

1 Functional Description

1.1 Narrative (word) description of design

In this section, describe how your design for the Attachment Mechanism (AM) works. In a few sentences, please describe how your solution does each of the following:

1) Unpack: How does your AM move from the *packed* to *open* configuration without exceeding the dynamic envelope?
2) Close: How does your AM move from the *open* to *attached* configuration without exceeding the dynamic envelope?
3) Hold: How does your AM maintain the *attached* configuration?
4) Release: How does your AM move from the *attached* to *open* configuration without exceeding the dynamic envelope?
5) Pack: How does your AM move from the *open* to *packed* configuration without exceeding the dynamic envelope? If this is an exact reversal of unpack, you may just say so.
6) Astronaut Pull Away: How does your AM enable releasing from the handrail when the astronaut pull load is applied to the interface plate?

Although it is not required, you may embed images with sketches, models, storyboards or other illustrations in your written descriptions to help explain how your AM design accomplishes these high-level operations.

Minimum content requirement: Text reponse to each of the above questions.

1.2 Functional Analysis

In this section, describe your logic and/or analysis for the following aspects of your AM design. Including equations and mathematics is acceptable if it helps clarify the logic behind your design, but please ensure that it will be understood by our reviewers by annotating your process or describing the math being done and why.

(1) What is the typical force you expect your AM to exert on the Handrail while *attached* (R3)?
(2) How does your AM design close on a Handrail when the Handrails has its maximum offset (per R2.1)?
(3) In your AM design, how close do you get to the packed volume (R5)?
(4) How did you confirm that your AM will not exceed the power limits (R12, C3, C4)? Explain the assumptions you made in selecting and operating any active components (e.g., actuators and sensors).

Mimumum content requirement: Text responding to each of the above questions.

2 Mass Summary and Component List

In this section, list all the elements of your AM solution using the template provided [AMMassTemplate available as google doc, .odt, .xlsx]. For each component/piece/part, include an estimate of its mass and a brief explanation of where the estimate came from. Please be sure to include the reasons supporting your mass estimate for each element since they will be part of the evaluation of the credibility of your AM mass estimate.

Table 1 provides an example of how the template should be filled in.

		Is this a powered component?	Estimated Mass per Unit (kg)	Quantity (# units)	Mass (kg)	Basis of Estimate
**1.0**	**Subsystem #1**				**1.88**	
	Electronics Box A	Yes	0.200	1	0.20	Weighed a prototype I built
	Switch #1 & 2	Yes	0.030	2	0.06	Called some former coworker who builds these, and asked for a typical masses
	Mechanism #1	No	0.800	1	0.80	Made a CAD model, assumed SS316, to obtain this mass
	Attachment hardware	No	0.040	8	0.32	Typical mass of component that I use all the time in design of systemX.

Table 1 Mass Summary and Component list - example

Minimum content requirement: Paste your filled table into this section of the document. No additional text is required.

3 System Layout

In this section, provide a diagram(s) identifying all the physical components/piece/part of your AM design and how components connect to, and/or move each other.

Please use the names of components from the Mass Summary and Component List described in section 2.

In representing your design you may use one of the following formats:

Block Diagram: A Block Diagram shows how each of the components connect to one another. Each component is represented as a box and the lines that connect the boxes identify what is being transferred/passed or supported between the components (blocks). Be sure to identify how your design connects to the robotic arm interface. Figure 1 provides an example of a block diagram.

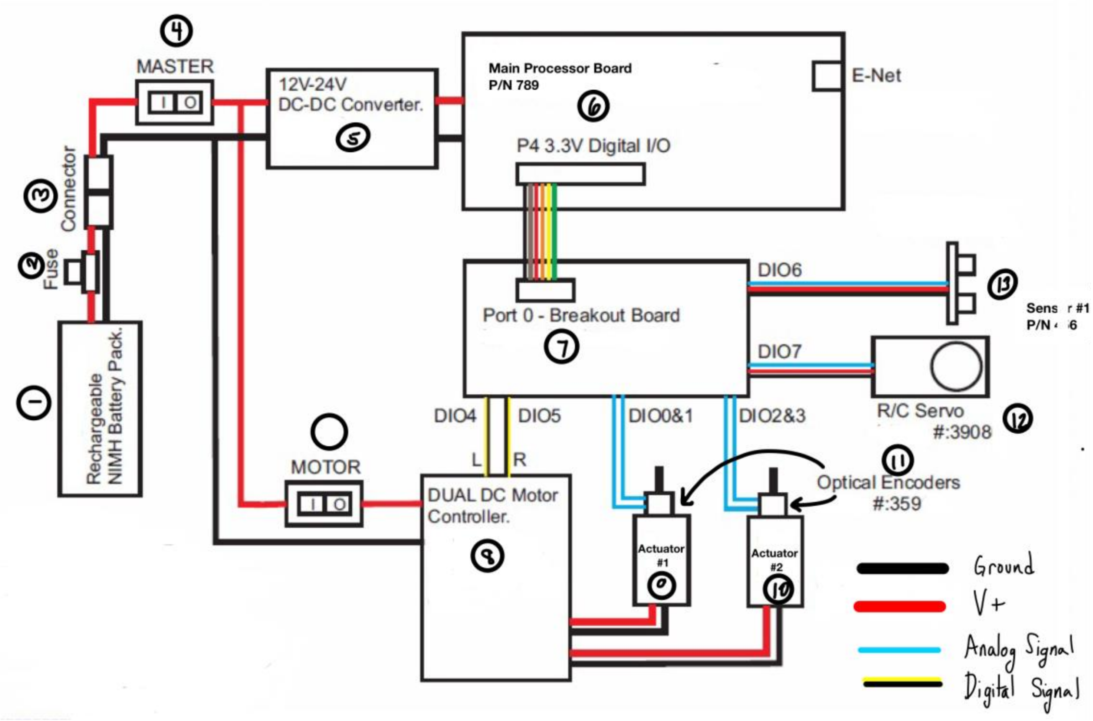

Figure 1 Block Diagram - example

Electro-Mechanical Schematic: An Electro-mechanical schematic similar, to an "Exploded View." See Figure 2 for an example. The defining feature of an exploded view is that it conveys information about how all the mechanical and electro-mechanical (actuators and sensors) pieces connect to one another.

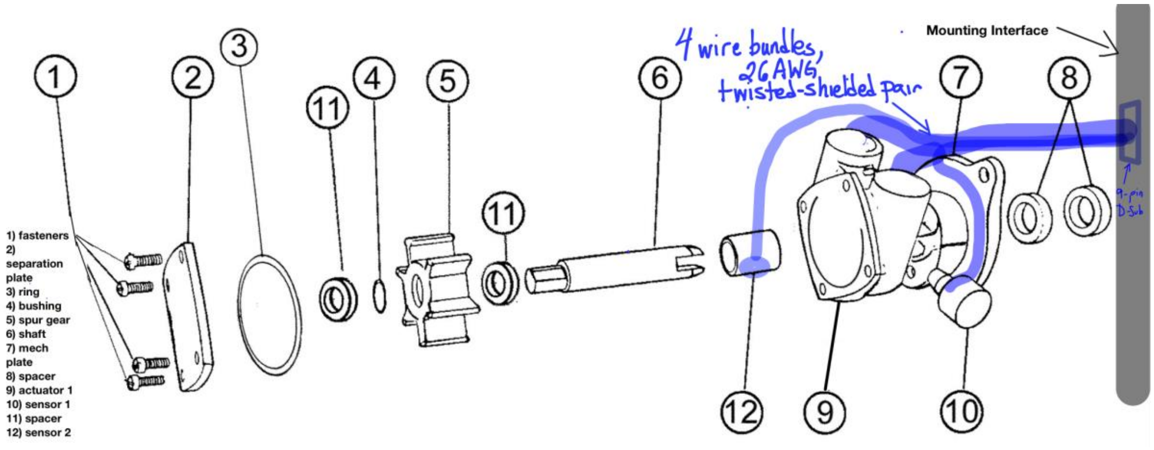

Figure 2 Electro-mechanical layout - example

Minimum content requirement: Your submission must include either 1) a block diagram that shows all your electro-mechanical and mechanical components and how they connect to one another or 2) an electro-mechanical schematic, like an exploded view, that shows the same information. A legend defining any symbols or colors used in the diagrams is also required.

4 Electro-Mechanical Design

4.1 Design Drawings

In this section, include engineering-style drawings showing your solution's physical configuration for each of the following.

1) *Packed configuration* (R1.1)
2) *Open configuration* (R1.2)
3) *Attached configuration* (R2.2)

You can use any CAD software you like, sketch them by hand, or photograph a prototype, but accurate dimensions of the whole system are required. At minimum, you must indicate the position of every electro-mechanical component (e.g., actuators and sensors, but not individual bolts). On each figure, please label as many elements and subassemblies as possible. Use the names specified in your Mass Summary and Component List (section 2).

Minimum content requirement: Three design drawing figures – one for each configuration listed above. Figures must be clearly labeled and dimensioned.

4.2 Operating Modes

In this section, briefly explain how you expect your design to be used for each operation. Specifically, define what 'ending the operation' means in the context of your AM design for:

1) Unpack (ends in open configuration)
2) Close (end in attached configuration)
3) Release (ends in open configuration)
4) Pack (ends in packed configuration)

There should be enough detail for the separately designed control system to "start" an operation by moving components and "know" when your AM ends that operation. Here are three contrasting examples of the kind of information that should be conveyed:

- Operating modes based on joint position: If you have two rotational joints and have mounted sensors that measure the angles of those joints, specify what joint angle(s) (i.e., the number of degrees) correspond to ending pack (i.e., being *packed*).
- Operating modes based on current threshold: If you intend the motor's stall torque to be used to define when a mechanism is closed, specify what current threshold corresponds to ending close (i.e., being *attached*).
- Operating modes based on timing: if your actuators will always reach a particular position when powered for a fixed time interval, specify how long they should be powered to complete, for example unpack.

The above are examples of the kind of information to be included in your submission

Table 2 provides a template for how you should communicate this information. The first row fills in one example, as illustration. Please use the same names as in the Component List in describing your operation modes.

Operation	Components Moving	Active Components Involved	Description of Operation	Termination Condition
**1) Pack** ***Example***	*Component A Component B*	*Actuator #1 Actuator #2 Sensor #1 Sensor #2*	*Actuators #1 and #2 move components A and B until Sensors #1 and #2 show particular position*	*Sensor #1 value =+20^0 AND Sensor #2 value = -20^0*
**2) Unpack**				
**3) Close**				
4) **Release**				

Table 2 – Example Template

Minimum content requirement: One table (like Table 2) with four rows, each defining how your AM design fulfills the operating modes (unpack, close, release and pack).

4.3 Actuator Design

For each actuator in your design you must tell us what kind of actuator is it and what is required to drive it. If the actuator uses an off-the-shelf driver, simply list it. If your actuator requires a non-standard type of driver you must also specify the custom driver.

Minimum content requirement: Text describing 1) the type of each actuator; 2) how that actuator is operated (e.g., list off-the-shelf driver)

5 Exit Survey

To complete your submission, please take the Exit Survey by going to this webpage:
https://seasgwu.qualtrics.com/jfe/form/SV_2r9DaeSlh48uMcZ

At the end of the survey you will receive a unique code. In your submission include this section and the text: Exit Survey for Freelancer <<insert Freelancer username>> complete per completion code: <<insert completion code>>.

To be complete, your submission must include the following text: Exit Survey for Freelancer <<insert Freelancer username>> complete per completion code: <<insert completion code>>.
