# Supplementary material for: Linking solver characteristics, solving processes and solution attributes: A data explainer for an open innovation generated robotic design dataset
Source: Data Brief. 2023 Sep 6;50:109547. doi: 10.1016/j.dib.2023.109547 (PMC10518673; doi:10.1016/j.dib.2023.109547)
Supplement: Supplementary file 1 [file mmc1.zip › Release/Process/Challenge Rules/D5-AM/AM Problem Description.pdf]

1 Contest Description

In this contest you are asked to design an Attachment Mechanism (AM) that will be mounted to the free end of a separately designed robotic arm. ***This challenge is focused on the electro-mechanical system only (i.e., no internal computing or circuitry).*** The AM will be powered and controlled by the robotic arm to execute the following functions: packing and unpacking from a stowed configuration, closing on and releasing from an International Space Station (ISS) Handrail ("Handrail") and maintaining a hold on that Handrail. The below specification details how the AM will work, it's functional requirements and interface constraints/assumptions. A separate document provides detailed guidelines on how your design must be presented and submitted.

A prize of $500 will be awarded for the lowest mass, technically feasible solution, submitted before 13:00 GMT on June 21st 2018.

2 Concept of Operations – How the AM needs to work

2.1 Normal Operations

When powered and controlled, the AM must be able to perform six operations: 1) unpack, which involves a transition from the *packed* configuration to the *open* configuration; 2) close, which involves a transition from the *open* configuration to the *attached* configuration; 3) hold, which involves maintaining the *attached* configuration while resisting externally applied loads; 4) release, which involves a transition from the *attached* configuration to the *open* configuration; 5) pack, which involves a transition from *open* configuration to *packed* configuration; and 6) standby which is a low powered waiting mode used between unpack and close, and release and pack.

In transitioning among configurations (i.e., from *packed* to *open* and *open* to *attached)*, the AM must never exceed its *dynamic envelope*. The relationships among the configurations and operations are illustrated in Figure 1. The requirements for each operation (underlined) and intermediate configuration (italicized) are detailed in section 3.

2.2 Contingency (Emergency) Operations

There are several scenarios when normal operations may be disrupted. The ranges of permissible responses are detailed in section 3.4. This section summarizes the scenarios: 1) when the AM attempts to attach, but there is no handrail present; 2) when the AM experiences higher than expected loads while attached (e.g., because an astronaut or other object bumps or smashes into Astrobee); and 3) when an astronaut manually removes the AM from the handrail.

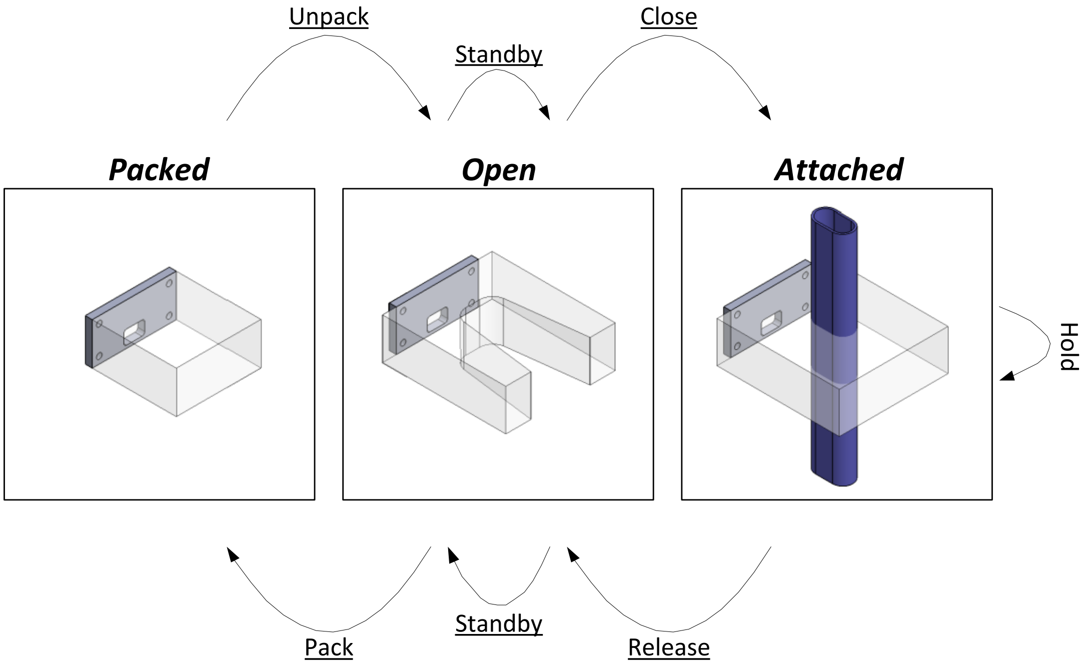

Figure 1 – Concept of Operations Illustration

3 Functional Requirements

This section details all of the functional requirements that the AM must meet.

3.1 Motion Requirements

R1 <u>Unpacking</u>: The AM shall be able to move from the *packed* configuration to the *open* configuration without exceeding the *dynamic envelope.*

R1.1 Packed configuration: The packed configuration volume is defined in Figure 2, as 76.2mm x 76.2 mm x 38.1mm [3” x 3” x 1.5”]

R1.2 Open configuration: The open configuration volume is defined in Figure 3, as 114.3mm x 101.6mm x 38.1 mm [4.5” x 4” x 1.5”], with a cutout to permit placement on the Handrail.

R1.3 Dynamic envelope: The dynamic envelope is defined in , as 120.65 x 114.3 x 50.8 mm [4.75” x 4.5” x 2“].

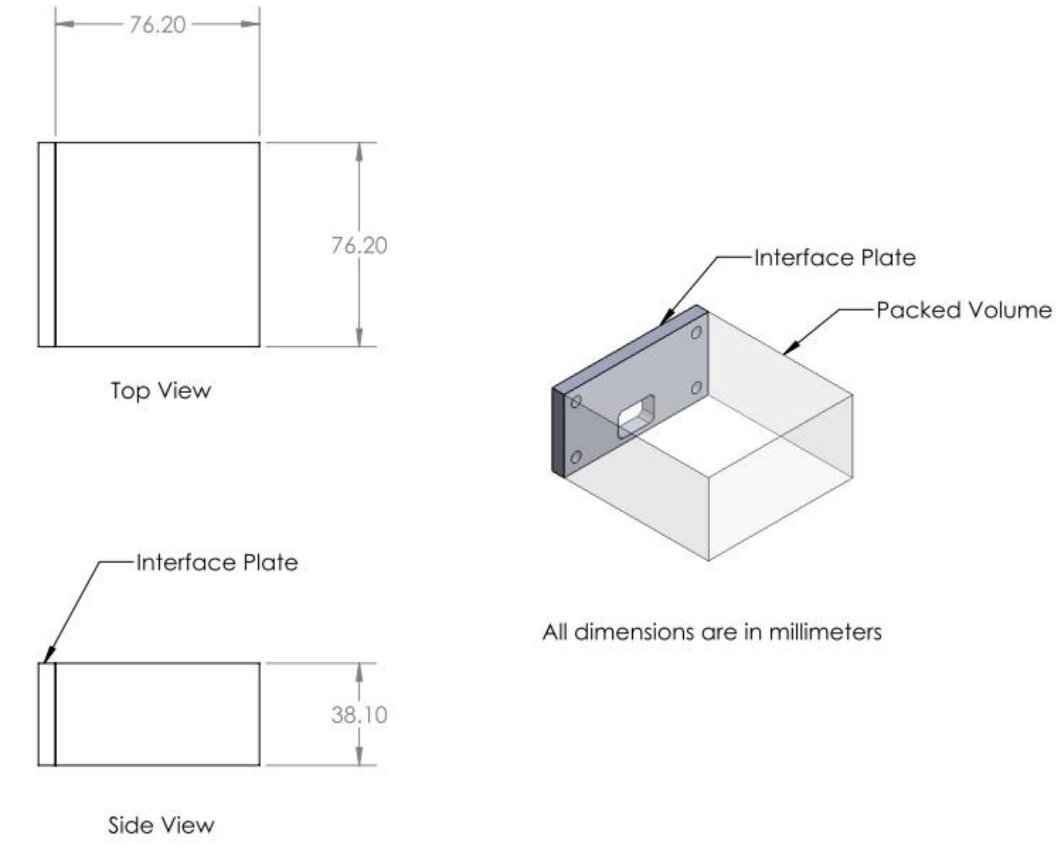

Figure 2 – Packed Configuration

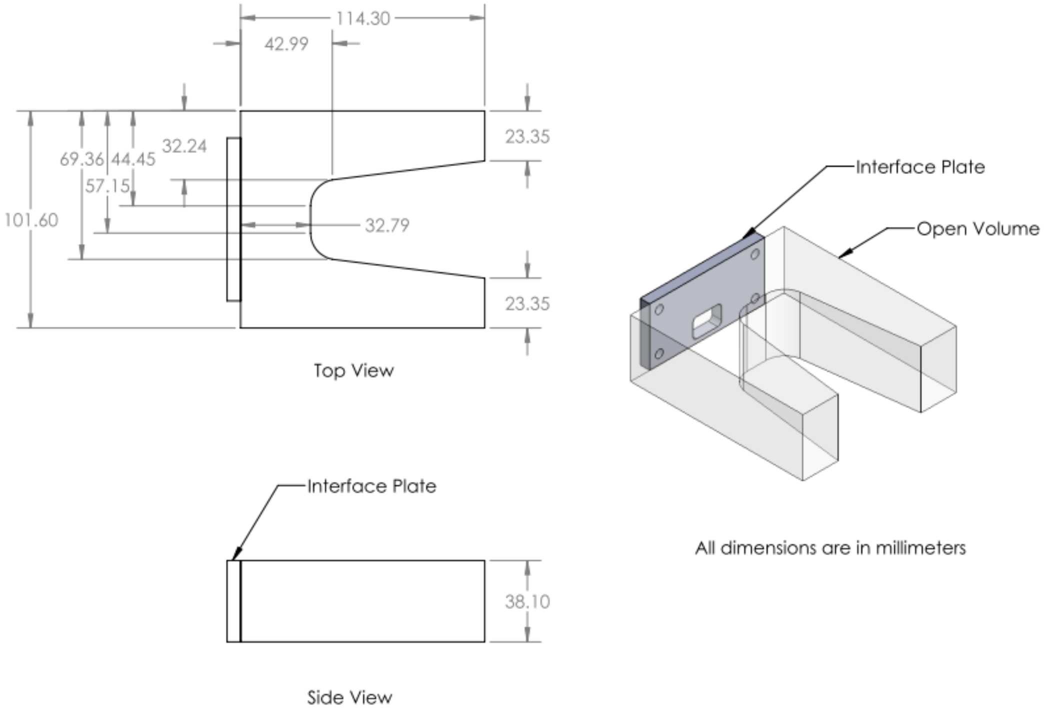

Figure 3 – Open Configuration

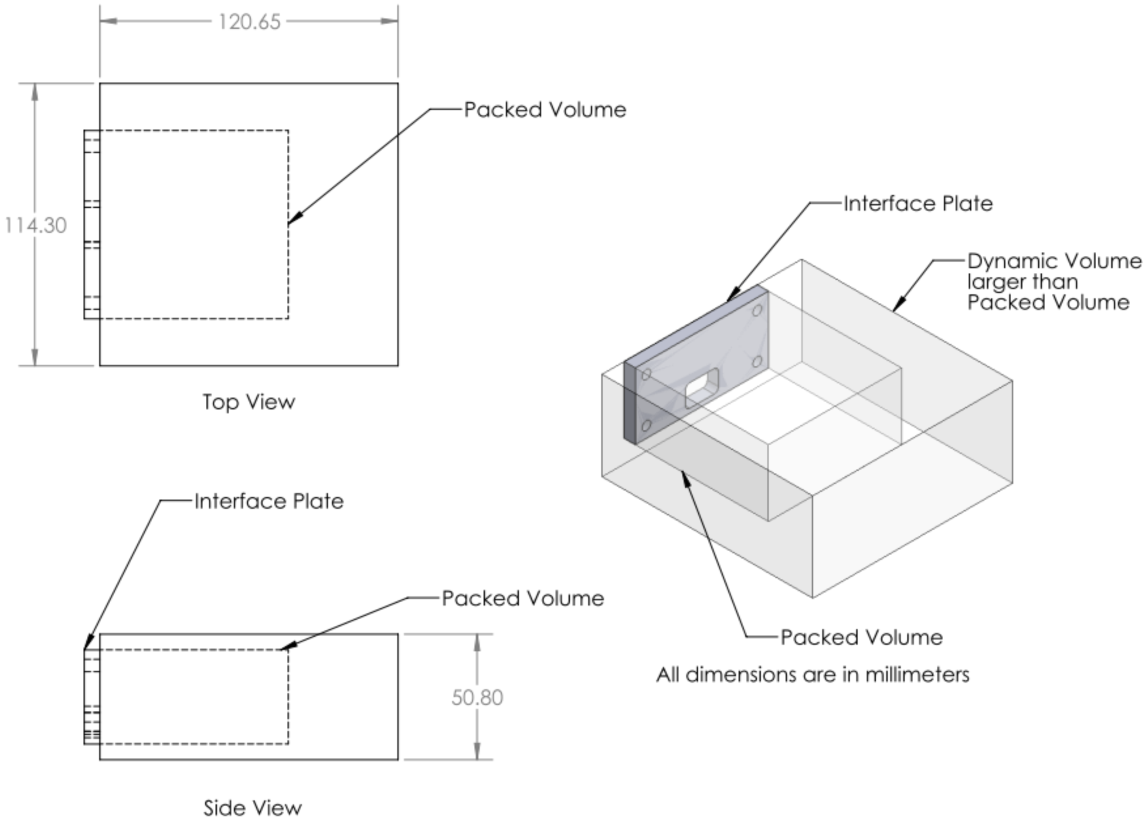

Figure 4 - Dynamic Envelope

R2 Closing: The AM shall be able to move from the *open* configuration (R1.2) to the *attached* configuration without exceeding the dynamic envelope (R1.3).

R2.1 Pre-attach offset: When closing is initiated, the AM shall not be offset from the center of the ISS Handrail by more than:

- x = 58.7 mm ± 6.35 mm [2.3” ± .25"]
- y = 0mm ± 6.35 mm [0” ± .25"]
- z = 0 mm ± 6.35 mm [0” ± .25"]
- θ_x = 0 ± 5 degrees
- θ_y = 0 ± 5 degrees
- θ_z = 0 ± 5 degrees

The coordinate system for these offsets is shown graphically in Figure 5. The AM shall only ever be commanded to attach to a standard ISS Handrail as defined C8.

R2.2 Attach configuration: The AM shall be considered attached when it if fixed to the Handrail. Fixed is defined as being able to resist slipping or twisting when subjected to normal operating loads of up to 3.5 Nm [2.6 ft-lbf] about either the Y-axis or Z-axis (ref Figure 5). While attached, the AM shall not exceed the attached configuration volume defined in Figure 6.

R3 Holding: The AM shall be able to maintain a rigid attachment (defined in 0) for an extended period of time (per R9).

R4 Releasing: The AM shall be able to move from the *attached* configuration (0) to the *open* configuration (R1.2) without exceeding the *dynamic envelope* (R1.3)

R5 Packing: The AM shall be able to return to the *packed* configuration (R1.1) from the *open* configuration (R1.2) without exceeding the *dynamic envelope* (R1.3)

R6 Standby: The AM shall have a standby mode wherein it uses minimal power but can receive commands.

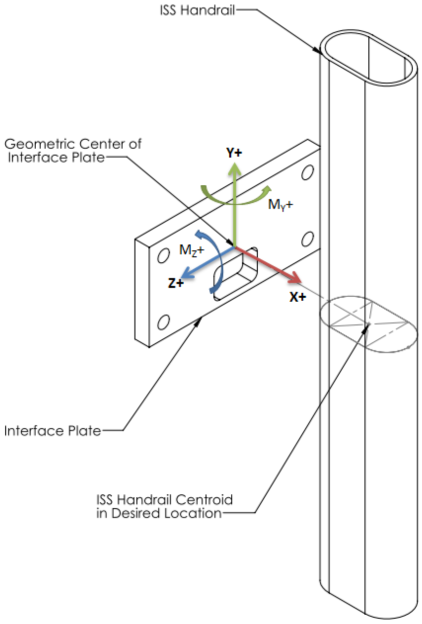

Figure 5 – AM Frame of Reference.

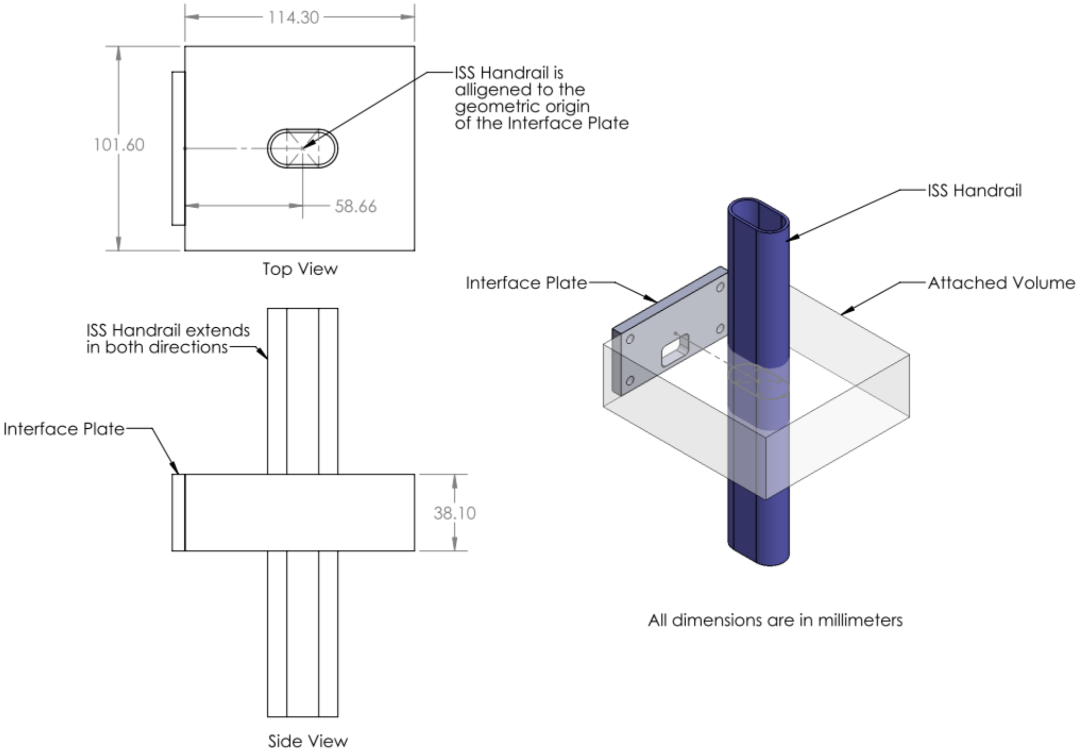

Figure 6 - Attached configuration

3.2 Sensor and Wiring Requirements

R7 The AM design shall include enough sensors to provide the information that the robotic arm needs to control its operations. For each system configuration (i.e., packed, open and attached) your submission must define a) what that configuration means in the context of your design and b) what sensing or signal outputs you are providing to enable the robotic arm to determine when/if the AM is in that configuration. Your design must also include the placement of the selected sensors, if any.

R7.1 The categories of permissible sensors and their mechanical properties are provided in Table 1. No other sensors may be used. Further information about the design, mounting, and sensing elements of the available sensors is provided in the appendix.

R7.2 All sensors have wire bundles that need to be considered in your mechanical design and mass. In selecting active components (sensors and actuators) for your design, include mechanical accommodation of wires from the component to the interface plate. Wires should be fixed to structure to relieve strain if you expect the wires to be subjected to significant deflection or movement (e.g. pulling, twisting) during regular operations. In accommodating your sensor wire bundles, assume outside bundle diameter of 2.5 mm, with a 12.6 mm minimum bend radius, and a linear mass of 12.8 kg/km (26 AWG twisted shield pair bundles).

Table 1 - Permissible Sensor Options

Abstracted Sensor Decomposition	Volume	Mass of Sensor	Energy Cost	Information provided
No explicit sensor	You are free to use a non-sensing approach, for example, using the stall torque of your motor to determine when a mechanism has closed. There is no extra mass or volume for this, but be sure to clearly communicate how the design is intended to work in your submission.			
Continuous Rotation Sensor	Small knob with hole for sensing shaft (see Figure 11)	4 grams	0.09 Wh	Angle: Continuous and/or relative rotation of sensor shaft hole at 0.1 degrees resolution
Contact Sensor	Flat box with sensing face that depresses 1 mm (see Figure 12)	1 gram	0.02 Wh	Whether contact has been made: Discrete: On or Off
Linear Displacement Sensor	Flat plate with sliding sensing head that can slide 1” (see Figure 13)	10 grams	.375 Wh	Position: Continuous and Relative Displacement Information at 1 mm resolution.
Force Sensor	Flat Plate that determines force placed on the sensing face (see Figure 14)	1 gram	.375 Wh	Force: Continuous and absolute force from 0-100 Networks at 1 Newton resolution.

3.3 Resource Requirements

3.3.1 Timing Requirements

R8 Time to Unpack and Attach: Combined, the unpack and close operations shall not exceed 2 minutes. Assume that each of the degrees of freedom of your design is operated in sequence when evaluating whether it can meet this requirement.

R9 Time in Hold: The AM shall not be closed on the ISS Handrail for more than 1hr.

R10 Time to Release and Pack: Combined, the releasing and packing operations shall not exceed 2 minutes. Assume that each of the degrees of freedom of your design is operated in sequence when evaluating whether it can meet this requirement.

R11 Time in Standby: Time in standby shall not exceed 26 minutes.

3.3.2 Power Requirements

You may assume that electric power is applied to all actuators and sensors as needed to drive them. The details of the interface and bus voltage are described in C2.3.

R12 Energy Budget: The AM shall not require more than 9 Watt-hours to support all operations (R1-R6). Assume a maximum of 1 hr 26 minutes of passive operations (during standby and hold) and up to 4 minutes of active operations (during unpack, close, release and pack).

3.4 Safety Requirements

R13 The AM shall have no sharp edges, defined as a radius of 3 mm [.11”], for astronaut safety.

R14 The AM shall have no loops of material greater than 25.4 mm [1”] in diameter for astronaut safety.

R15 The AM shall not damage itself through normal operations.

R16 The AM shall be able to return to its normal operations if power is momentarily lost.

3.5 Environmental Requirements

R17 The AM shall operate in the ISS zero gravity environment.

R18 The AM, when unpowered, shall not be damaged by electrostatic discharge <4,000V.

R19 The AM shall operate in an atmosphere comparable to that of Earth. Assume 21 degrees centigrade [70 degrees Fahrenheit], with low humidity, and pressurized to 100 kPA [750 mm Hg, 14.5 psi].

R20 The AM shall not contribute any particulates (e.g. dust) to the ISS atmosphere.

R21 The AM shall enclose all lubricated components to prevent lubricants from leaking into the atmosphere of the ISS.

3.6 Contingency Requirements

R22 No Handrail: In some cases, the AM may be driven into attach configuration but there is no Handrail present. The AM need not recognize this type of error, but shall also not damage itself while executing the operation.

R23 Excessive loads cases. This scenario may occur if an astronaut or piece of equipment contacts Astrobee while the AM is attached (including while experiencing normal operating loads per R2.2). The AM shall break away from the Handrail if it experiences a force of greater than 18N [4 lbf] applied at the AM interface in the negative Y-direction and a simultaneous moment of 5Nm [3.7 ft-lbs] about the positive Z-axis as seen in Figure 7

R24 Astronaut intervention: The AM shall be removable from the Handrail by an astronaut. Assume an astronaut can apply a pull-away force of 35.6 N [8 lbf] in the negative X-direction as seen in Figure 7. This will not occur during any other operation.

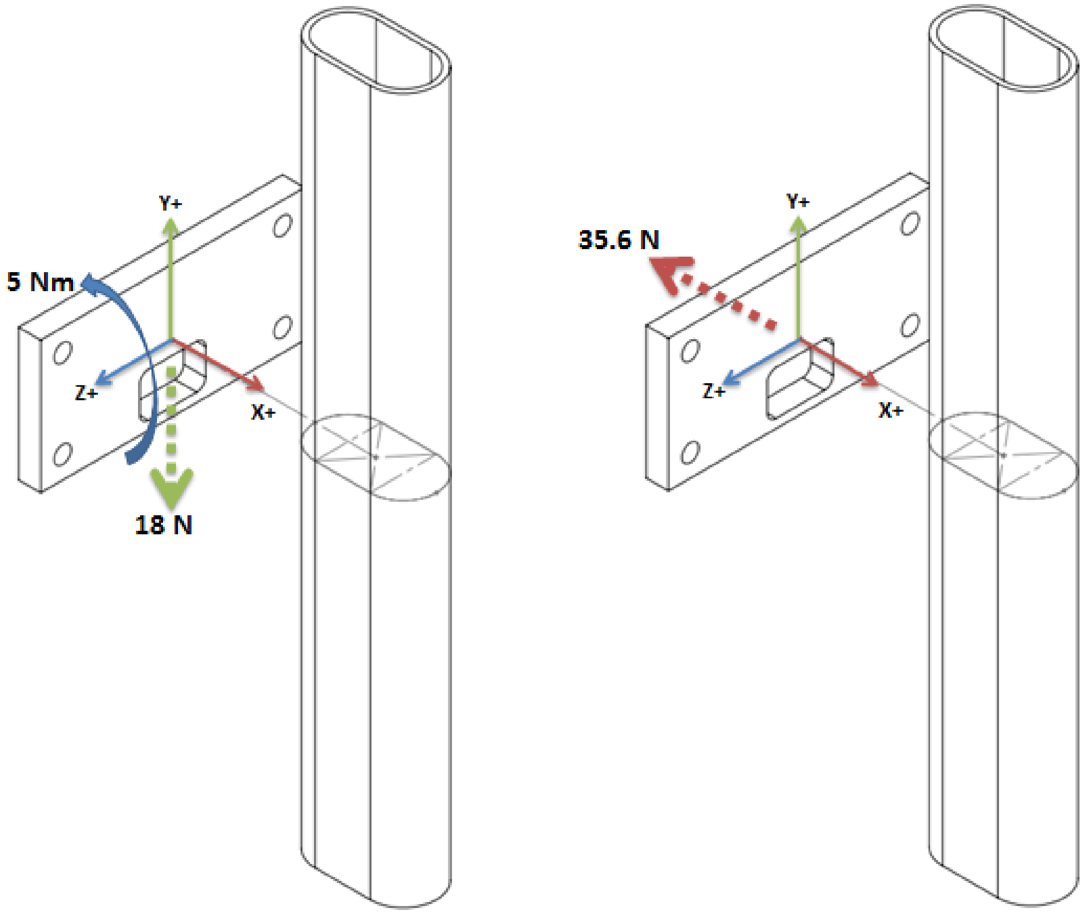

Figure 7 – Contingency loads: a) Excessive loads; b) Astronaut intervention loads

4 Requirements

The AM has a fixed interface to a separately designed robotic arm and a dynamic interaction with ISS Handrails. The section describes all constraints imposed by those interfaces.

4.1 AM-Robotic Arm Interface

4.1.1 Mechanical Interface

C1 Constraint 1 (C1) Mounting Interface: The AM shall mount to the interface plate shown in Figure 8. There are four available screw holes in the specified locations seen in Figure 9.

C1.1 All external loads are applied at the interface plate.

C1.2 All wires must fit through the hole in the interface plate. Actuator wires are defined by the design. If you wish, you may use an assumption of a 6 gauge

wire, at 5 mm in diameter [.192"], and a linear mass of 167 grams per meter [0.11 lb per feet] for each actuator.

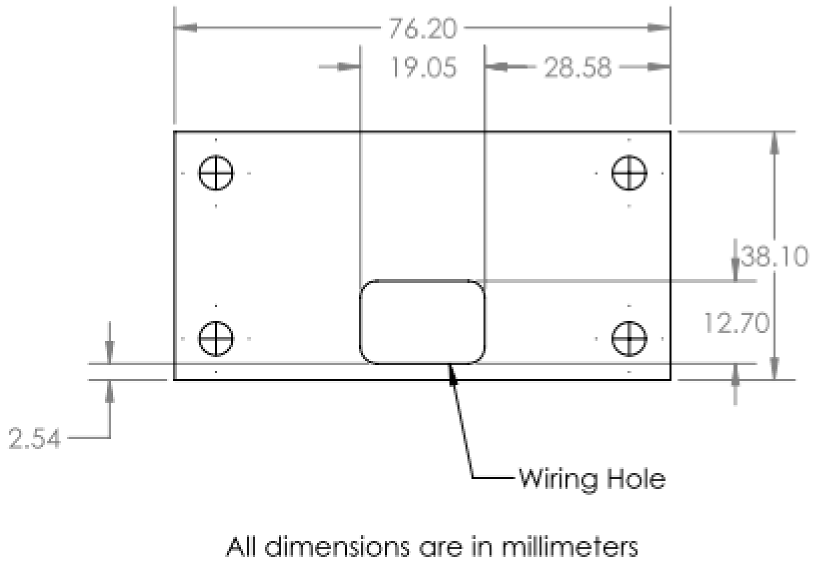

Figure 8 - AM-Robotic Arm Interface Plate

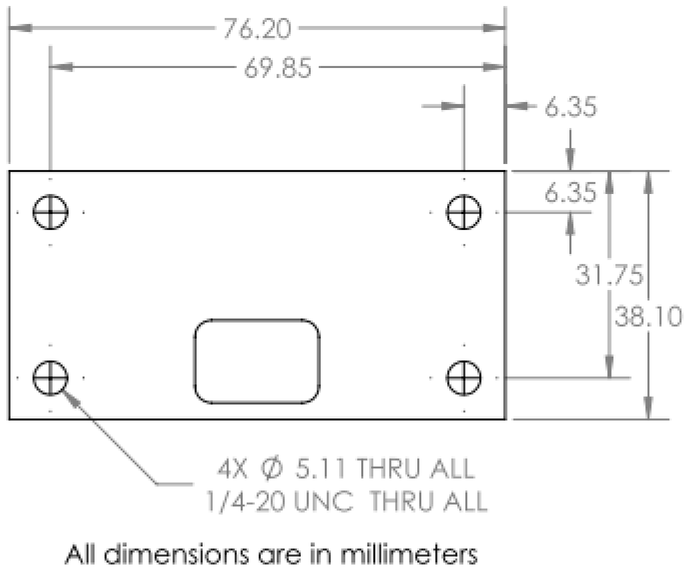

Figure 9 - AM Robotic Arm Mounting Holes

4.1.2 Power Interface:

C2 Astrobee Bus Voltage+ is nominally 14.4 volts DC, but can vary from 11 to 17 volts DC.

C3 Max current: The AM shall not draw more than 3 Amps.

C4 Steady State Current: The AM shall not draw more than 2 Amps at steady state.

C5 Astrobee Bus ground is available.

4.1.3 Control Interface:

C6 The robotic arm can provide any off-the-shelf actuator driver. If your design requires a non off-the-shelf driver, you must specify the driver. Here, actuators are defined as any device that moves a design element using electricity (e.g., solenoid, motor, piezo-motor, shape memory alloy or polymer etc.).

C7 All specified sensors (Table 1) can be read by the robotic arm.

4.2 Handrail Interface

C8 Handrail definition:

- C8.1 The shape of a standard Handrail is defined in Figure 10.
- C8.2 The ISS Handrail is made of anodized aluminum. Assume the material is 6061 Aluminum of type T4 in terms of material properties and friction properties.
- C8.3 The ISS Handrail is a 1.59mm [1/16”] thick aluminum 6061 extrusion.

C9 The Handrail shall not be damaged during operations through excessive force (per R2.3, R22 and R23). Damage includes, but is not limited to: crushing, denting, or bending.

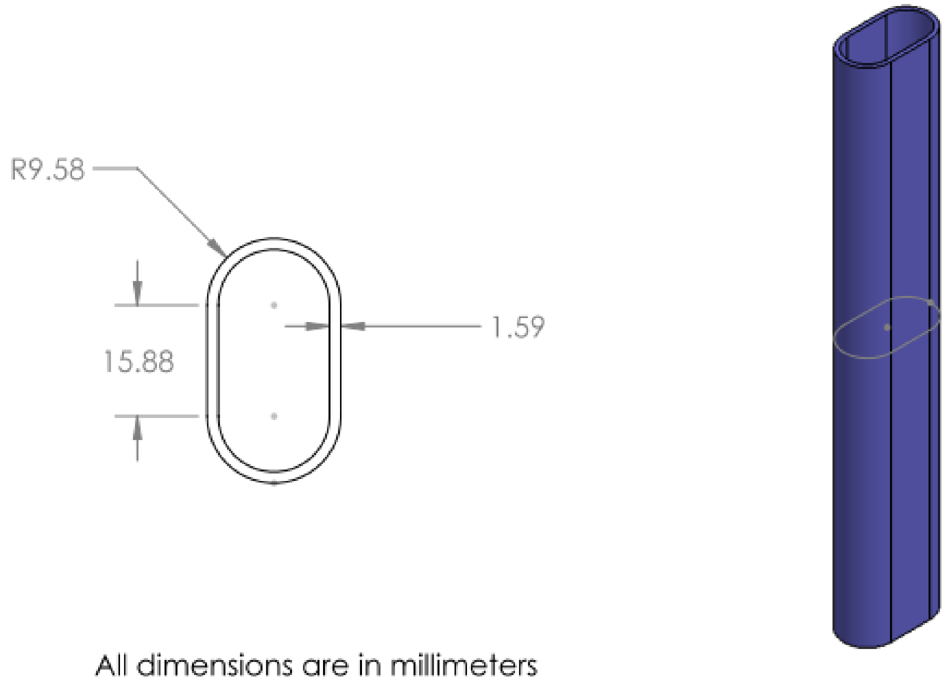

Figure 10 - ISS Handrail Definition

5 Sensor Appendix

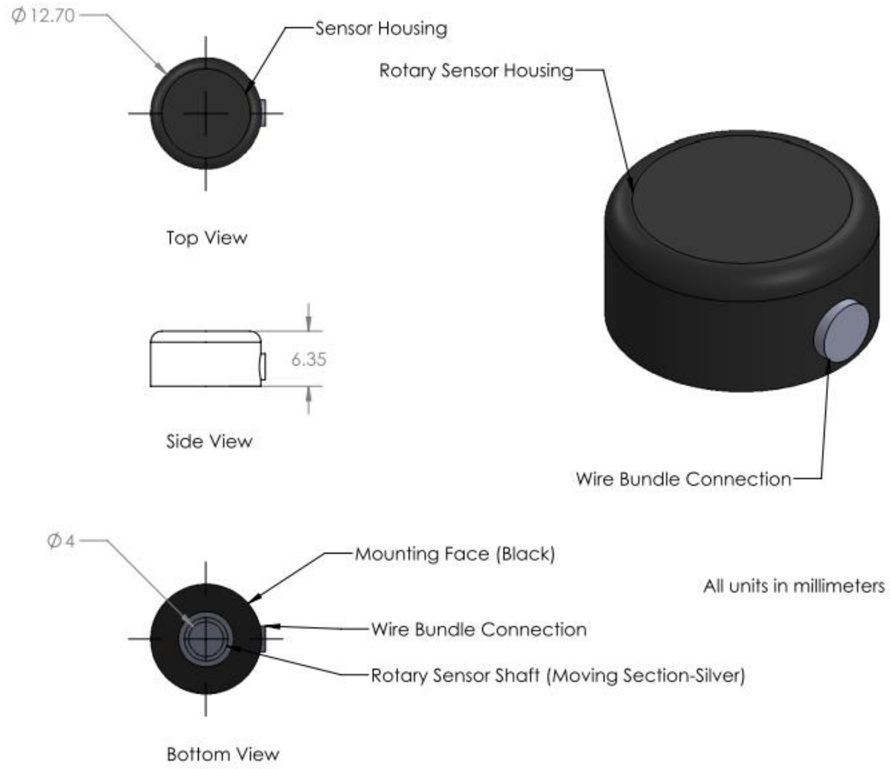

Figure 11: Continuous Rotation Sensor

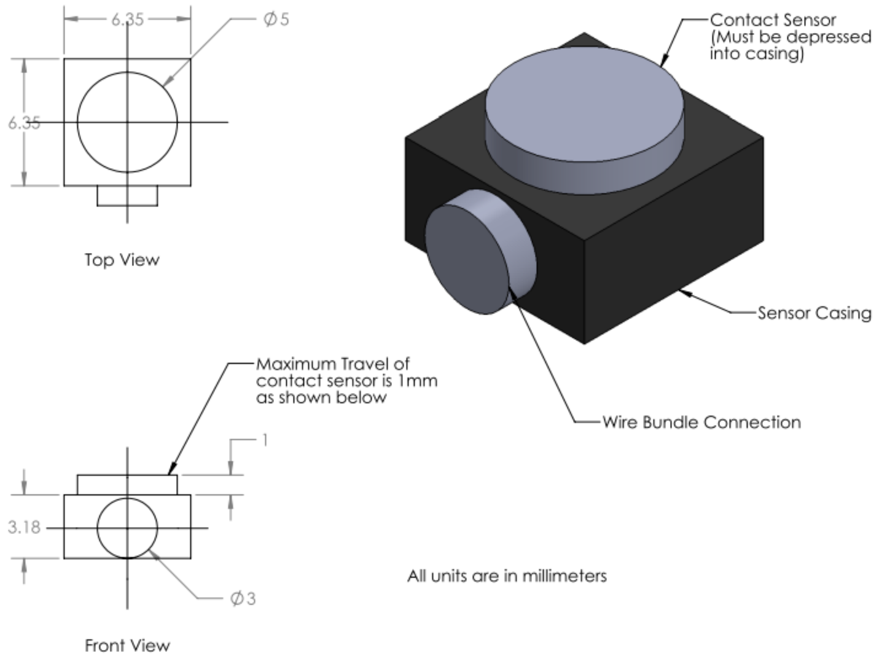

Figure 12: Contact Sensor

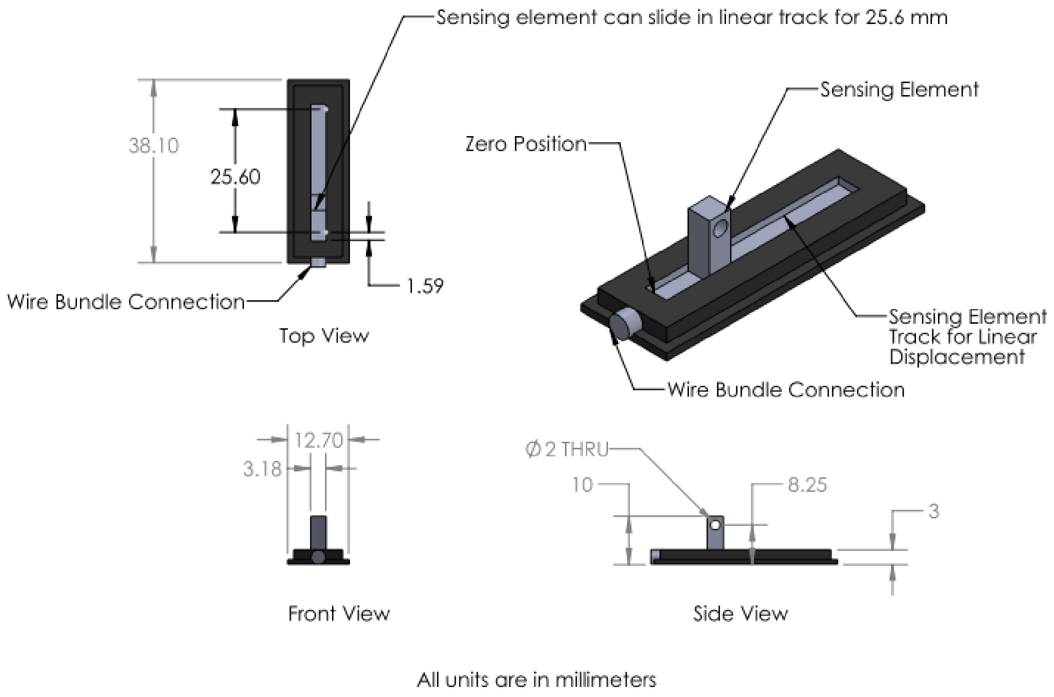

Figure 13: Linear Displacement Sensor

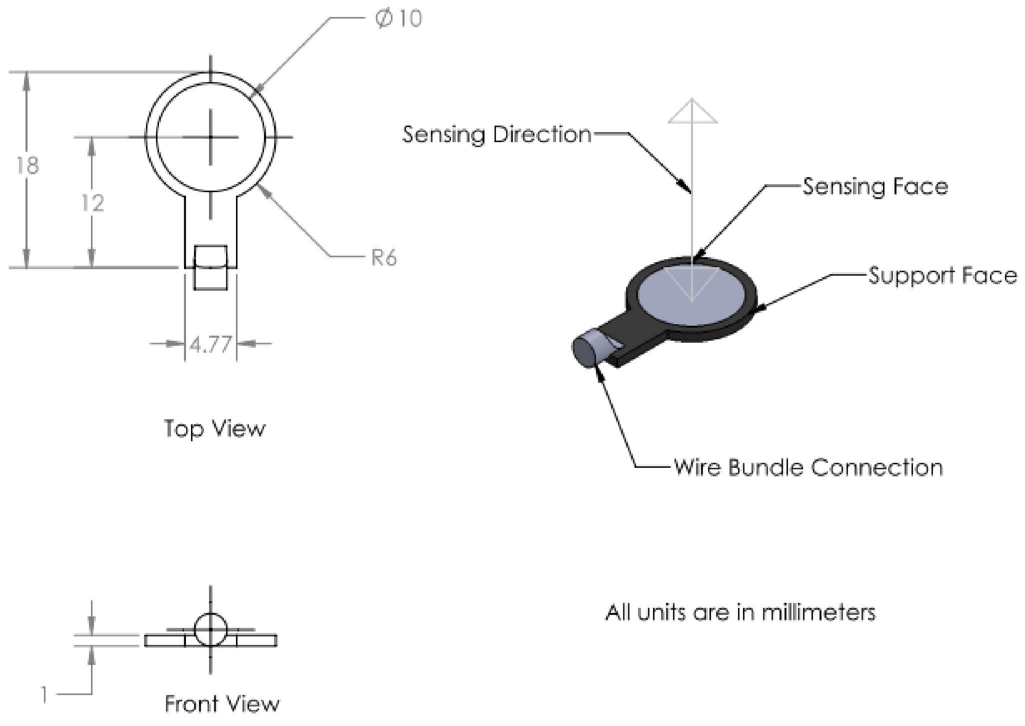

Figure 14: Stress Pressure Sensor
