# Supplementary material for: Linking solver characteristics, solving processes and solution attributes: A data explainer for an open innovation generated robotic design dataset
Source: Data Brief. 2023 Sep 6;50:109547. doi: 10.1016/j.dib.2023.109547 (PMC10518673; doi:10.1016/j.dib.2023.109547)
Supplement: Supplementary file 1 [file mmc1.zip › Release/Process/Challenge Rules/D5-AM/AM Blurb.docx]

Attachment Mechanism (AM)

In this challenge, you are asked to design an Attachment Mechanism (AM) that will be mounted to, and being controlled and powered by a separately designed robotic arm. This challenge is focused on the electro-mechanical system only (i.e., no internal computing or circuitry)

How it works: Initially, the AM will be packed in a stowed configuration. When powered and controlled, the AM must be capable of executing five high-level actions: 1) unpack in preparation for placement near a Handrail, 2) close on the Handrail, 3) maintain a hold on the handrail for an extended period of time, 4) release from the handrail, and 5) pack back into the initial stowed configuration.

*Click on the links below to see detailed design instructions, constraints and solution templates for this problem.*

Challenge rules: A prize of **$500** will be awarded for the **lowest mass, technically feasible** solution submitted by **June 21^st^ 2018**. No working prototype is required for submission, but the design must be sufficiently detailed to allow experts to assess the feasibility of your design (i.e., comply with all requirements) and the credibility of your mass estimate. Only complete submission packages will be evaluated.

Attachments:

AMProblemDescription.pdf

AMSubmissionGuidelines.pdf

Templates

- AMMassTemplate [.xlsx, .odt, [google docs](https://docs.google.com/spreadsheets/d/11VKX1Apn61nIKuCXwaYLpz0FZEjSXCgEqT-OGYqOZmg/edit#gid=276913658)]

Steve, if you need the actual link: https://docs.google.com/spreadsheets/d/11VKX1Apn61nIKuCXwaYLpz0FZEjSXCgEqT-OGYqOZmg/edit#gid=276913658

Actual Final Text Pasted from Freelancer Site Contest Description Field:

Design an Attachment Mechanism (AM)

In this challenge, you are asked to design an Attachment Mechanism (AM) that will be mounted to, and be controlled and powered by a separately designed robotic arm. This challenge is focused on the electro-mechanical system only (i.e., no internal computing or circuitry) 

How it works: Initially, the AM will be packed in a stowed configuration. When powered and controlled, the AM must be capable of executing five high-level actions: 
1) unpack in preparation for placement near a Handrail, 
2) close on the Handrail, 
3) maintain a hold on the handrail for an extended period of time, 
4) release from the handrail, and 
5) pack back into the initial stowed configuration. 

Click on the attachments below to see 1) the AM Problem Description, which provides more detailed instructions about all the requirements your design needs to meet; 2) AM Solution Guidelines, which details the format an content that your submission must follow; and 3) the templates that are referenced in the solution guidelines. Microsoft Excel and Open Office Calc formats are attached below. Should you prefer a Google Sheets format, you can access it here: https://docs.google.com/spreadsheets/d/11VKX1Apn61nIKuCXwaYLpz0FZEjSXCgEqT-OGYqOZmg/edit#gid=276913658

Challenge rules: 
- The prize will be awarded for the lowest mass, technically feasible solution, submitted by the contest deadline. 
- No working prototype is required for submission, but the design must be sufficiently detailed to allow experts to assess the feasibility of your design (i.e., comply with all requirements) and the credibility of your mass estimate. 
- Only complete submission packages will be evaluated (see attachments).

NASA may select multiple winners or provide additional prize compensation on entries that are particularly novel or innovative.

NASA will be available to respond to clarifying questions, but feedback on quality is otherwise limited. 
All complete submissions will be confirmed with 3-star ratings. 
Note that final judgment of quality and winners will only happen after the submission deadline.
