# Supplementary material for: Linking solver characteristics, solving processes and solution attributes: A data explainer for an open innovation generated robotic design dataset
Source: Data Brief. 2023 Sep 6;50:109547. doi: 10.1016/j.dib.2023.109547 (PMC10518673; doi:10.1016/j.dib.2023.109547)
Supplement: Supplementary file 1 [file mmc1.zip › Release/Process/Challenge Rules/D3-SCA/SCA Blurb.docx]

# Smart Coarse Positioning Arm (SCA)

In this contest, you are asked to design a “Smart” Coarse-positioning Arm (SCA) that will be mounted to Astrobee and move a stowed end effector to a specified location and control the end effector. The SCA receives all power and high-level commands from the Astrobee free flyer, but implements the following functions autonomously: stowing and deploying from a payload volume, positioning its free end at a specified location near and Handrail, and orienting Astrobee by panning and tilting it.

How it works: Initially, the SCA will be packed in a stowed configuration. When powered and commanded by the Astrobee, the SCA must be capable of performing three operations autonomously: 1) deploy, which involves moving from a stowed configuration and placing an interface plate at a pre-determined location and then commanding the End Effector to attach, 2) orient, which involves the SCA moving the attached Astrobee side to side and up and down around the now-static interface plate, and 3) stow, which involves commanding the end effector to stow and then stowing itself and the attached end effector back in the stowage volume.

*Click on the links below to see detailed design instructions, constraints and solution templates for this problem.*

Challenge Rules: A prize of **$1,500** will be awarded for the **lowest mass, technically feasible** solution submitted by August 15^th^, 2018. No working prototype is required for submission, but the design must be sufficiently detailed to allow experts to assess the feasibility of your design (i.e., comply with all requirements) and the credibility of your mass estimate. Only complete submission packages will be evaluated.

Attachments:

SCAProblemDescription.pdf

SCASubmissionGuidelines.pdf

Templates

SCAMassTemplate [.xlsx .ods]

SCAPowerTemplate [.xlsx .ods]
