# Supplementary material for: Linking solver characteristics, solving processes and solution attributes: A data explainer for an open innovation generated robotic design dataset
Source: Data Brief. 2023 Sep 6;50:109547. doi: 10.1016/j.dib.2023.109547 (PMC10518673; doi:10.1016/j.dib.2023.109547)
Supplement: Supplementary file 1 [file mmc1.zip › Release/Process/Challenge Rules/D3-SCA/SCA Submission Guidelines.pdf]

Submission Guidelines for the Smart Coarse Positioning Arm

In this contest, you were asked to design a "Smart" Coarse Positioning Arm that will be mounted to Astrobee and place an End Effector at a given location.

This document provides detailed guidelines on how you must describe and present each aspect of your design in order to be considered for the prize. This document looks long but very little text is required. Your submission document must include each of the sections detailed below and all of the information requested in each. Several templates and examples are provided to clarify what constitutes a complete solution.

Use the exact section and subsection header words, shown below.

1 FUNCTIONAL DESCRIPTION 2

1.1 NARRATIVE (WORD) DESCRIPTION OF DESIGN 2

1.2 FUNCTIONAL ANALYSIS 2

2 MASS SUMMARY AND COMPONENT LIST 3

3 SYSTEM LAYOUT 4

4 DESIGN DRAWINGS 6

5 SOFTWARE DESCRIPTION 7

6 POWER USAGE DESCRIPTION 9

7 EXIT SURVEY 10

1 Functional Description

1.1 Narrative (word) description of design

In this section, describe how your design for the “Smart” Coarse Positioning Arm System works. In a few sentences, please describe how your solution does each of the following:

1) Deploy: How does your SCA move from the stowed configuration to deploy the interface plate?
2) Orient - Pan: How does your SCA pan the Astrobee side to side?
3) Orient - Tilt: How does your SCA tilt the Astrobee up and down?
4) Stow: How does your SCA stow itself into the Astrobee payload bay?
5) Astronaut Pull Away: How does your SCA handle the force of an astronaut pulling away?

Although it is not required, you may embed images with sketches, models, storyboards or other illustrations in your written descriptions to help explain how your SCA design accomplishes these high-level operations.

Minimum content requirement: Text reponse to each of the above questions.

1.2 Functional Analysis

In this section, describe your logic and/or analysis for the following aspects of your SCA design. Including equations and mathematics is acceptable if it helps clarify the logic behind your design, but please ensure that it will be understood by our reviewers by annotating your process or describing the math being done and why.

(1) When an astronaut applies a pull-away force (R24) what is the load at the End Effector Interface Plate?
(2) When the Astrobee is struck with an excessive load (R23), how does the SCA respond and how much force and torque is applied through the End Effector Interface plate?
(3) How does the SCA avoid colliding the Astrobee and the End Effector Keep Out volume when panning and tilting (R2.1)?
(4) How does your design transmit commands and power to the End Effector (C15-21)?
(5) How close does your SCA position the End Effector Stowage Volume to the boundaries of the Astrobee Stowage Volume/payload area (C1)?

Mimumum content requirement: Text responding to each of the above questions. Some responses must include a numerical value.

2 Mass Summary and Component List

In this section, list all the elements of your SCA solution using the template provided [SCAMassTemplate.xlsx, .odt]. For each component/piece/part, include an estimate of its mass and a brief explanation of where the estimate came from. Please be sure to include the reasons supporting your mass estimate for each element since they will be part of the evaluation of the credibility of your SCA mass estimate.

Table 1 provides an example of how the template should be filled in.

		Is this a powered component?	Estimated Mass per Unit (kg)	Quantity (# units)	Mass (kg)	Basis of Estimate
**1.0**	**Subsystem #1**				**1.88**	
	Electronics Box A	Yes	0.200	1	0.20	Weighed a prototype I built
	Switch #1 & 2	Yes	0.030	2	0.06	Called some former coworker who builds these, and asked for a typical masses
	Mechanism #1	No	0.800	1	0.80	Made a CAD model, assumed SS316, to obtain this mass
	Attachment hardware	No	0.040	8	0.32	Typical mass of component that I use all the time in design of systemX.

Table 1 - Mass Summary and Components List example

Minimum content requirement: Paste your filled table into this section of the document. No additional text is required.

3 System Layout

In this section, provide a diagram(s) identifying all the physical components/pieces/parts of your SCA design and how components connect to, move and/or, power each other.

Please use the names of components from the Mass Summary and Component List described in section 1. You may represent the electrical and mechanical aspects of your design together or separately.

If you represent mechanical and electrical together, the system layout should be presented as a block diagram. A Block Diagram shows how each of the components connect to one another. Each component is represented as a box and the lines that connect the blocks identify what is being transferred/passed or supported between the blocks. Be sure to identify how your design connects to the robotic arm interface. Figure 1 provides an example of a block diagram.

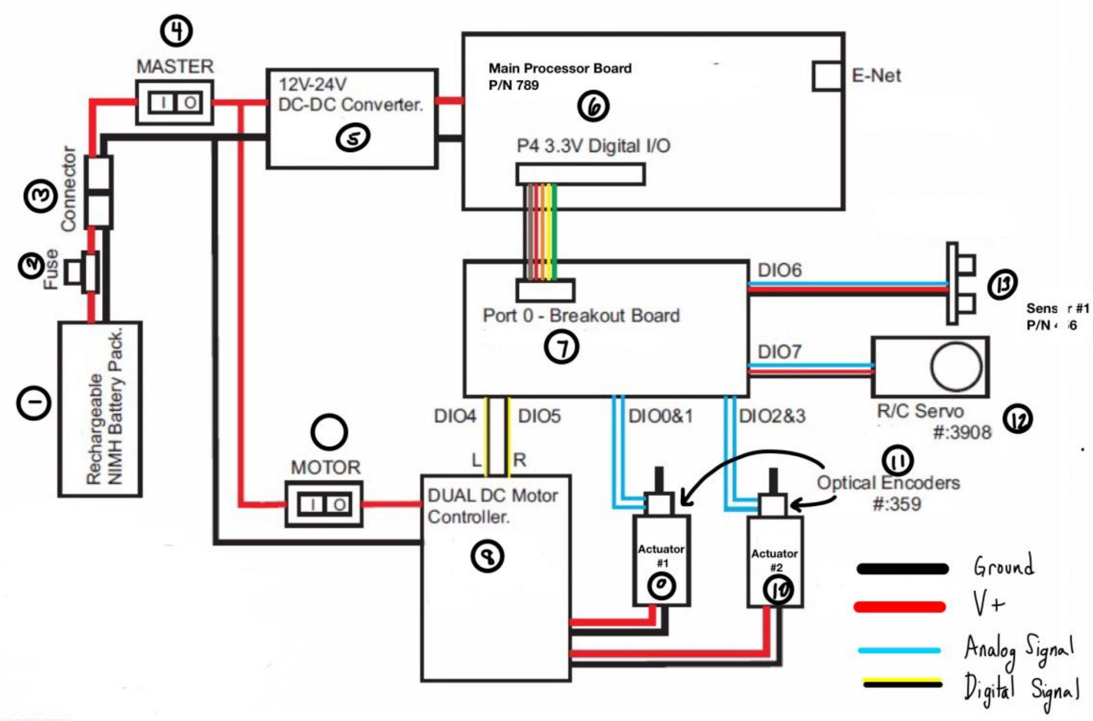

Figure 1 - Block Diagram - example

If you represent mechanical and electrical separately, you should use the following formats:

For mechanical, use a schematic similar to an “exploded view” of your design. See Figure 2 for an example. The defining feature of an exploded view is that it conveys information about how all the mechanical pieces connect to one another.

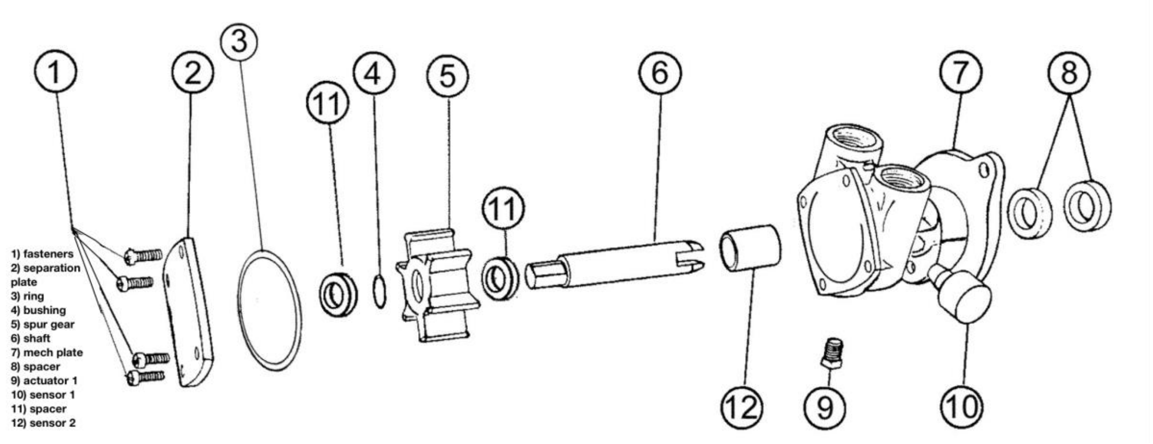

Figure 2 - Mechanical (exploded view) schematic - example

For electrical, use a schematic similar to a “Wiring Diagram” See Figure 3 for an example. The schematic must identify all the direct[1] electrical connections among all the electrical, electro-mechanical (such as actuators, motors, solenoids, etc.), and electro-computational (driver circuits, signal wires etc.) components of your solution. Make sure to identify any connection between your electrical system and the connector in the interface plate.

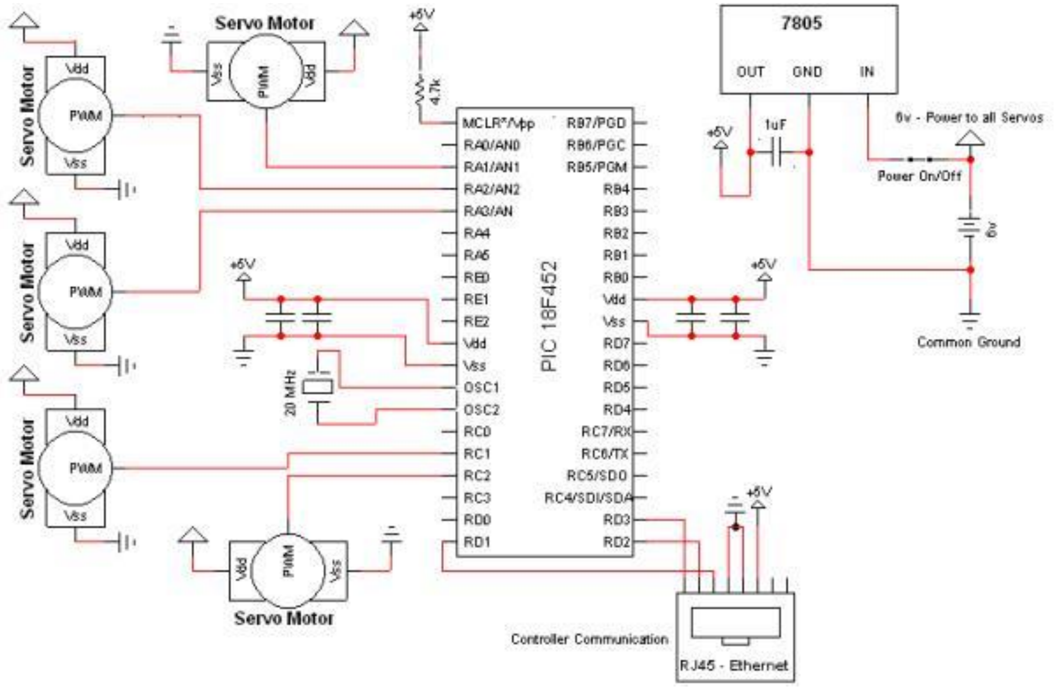

Figure 3 - Wiring Diagram - example

[1] Only detail the direct electrical connections. For example, if you have a gearbox connected to an electrical motor which has electrical leads coming off of it, this diagram would only show the electric motor and the electrical leads as well as those lead’s connection to the larger system. The diagram would not need to show the gearbox, as it is separated from the electrical system by the electric motor.

4 Design Drawings

In this section, include engineering-style drawings showing your solution's physical configuration for each of the following.

1) *Deployed – Placing the interface plate at the specified location (R1)*
2) *Oriented 30 degrees tilted and 45 degrees panned.*
3) *Stowing - Stowed in the final configuration*

You can use any CAD software you like, sketch them by hand or photograph a prototype, but accurate dimensions of the whole system are required (we'd much rather have proof that your deign can fit in its stowage volume than the specific location of each bolt). For each view, please provide at least one off-angle view to show perspective. Please label as many elements and subassemblies as possible. Use the names specified in your Mass Summary and Component List (section 1).

Minimum content requirement: Three design drawing figures – one for each configuration listed above. Figures must be clearly labeled and dimensioned.

5 Software Description

In this section, describe at a high-level how your SCA design autonomously accomplishes the following reference sequence of operations. You do not need to specify exact code or strategies involved in your software, just generally what function might be used and how. Be sure to include how each element or subassembly identified in the Component List is being controlled, and how your software uses any feedback from (e.g., sensors). Your software description should be complete enough that an experienced programmer could implement the algorithm.

Reference sequence:

1) Commanded to Attach
2) Commanded to Pan to 30 degrees immediately after attach
3) Commanded to Tilt to 90 degrees after 10 minutes
4) Commanded to Pan to -30 degrees after 20 minutes
5) Commanded to Tilt to -45 and then Pan to 45 degrees after 10 minutes
6) Commanded to Stow after 20 minutes

Present your software's control flow description using an Activity/Control Flow Diagram, also known as a Flowchart. An Activity Flow Diagram use squares to represent processes, diamonds to represent true/false case structures, ovals to indicate start and stops of programs, and arrows to designate program flow and sequence. Your Activity Flow Diagram must show every outcome and what steps are taken to get there. See Figure 4 for an example.

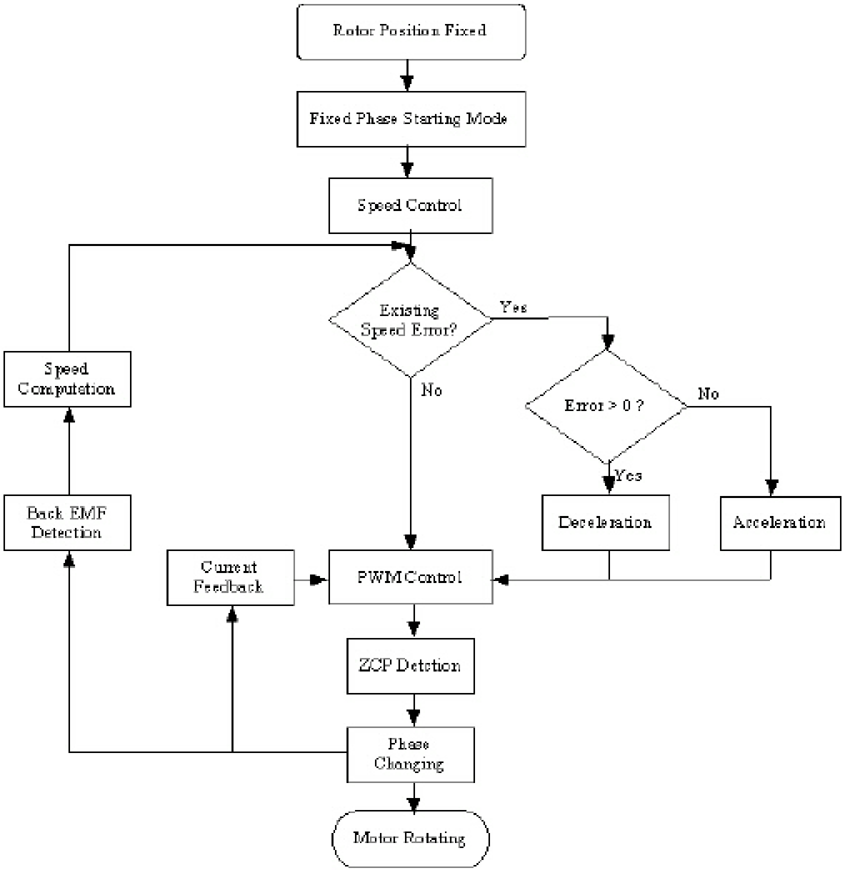

Figure 4 - Software Control Flow Diagram - example

Minimum content requirement: A flowchart that shows how your software would address the reference sequence. No text is required. Standard symbols must be used.

6 Power Usage Description

In this section, describe how your solution stays within the electrical power constraints (R13, C6, C7) by populating the attached Power Profile template [SCAPowerProfileTemplate.xlsx, .ods]. For each component/part that uses power, you must estimate its usage (time and power level) during each expected operation. The operational sequence for power usage is:

(1) Deploy
(2) Orient for 60 minutes (moving back and forth between pan 45 degrees, tilt 30 degrees and pan -40 degrees, tilt 0 degrees every 5 minutes)
(3) Stow to the Stowed configuration

Table 2 shows an example of a filled in power profile.

Step 1: Description of power modes

	**Power Mode Description**
**Mode 1**	Reading all sensors,
**Mode 2**	Running Actuators #1, Sensor #2, and Switches #1, #4-#6
**Mode 3**	Power draw while waiting (and attached to Handrail) for next operation

Step 2: List of Powered Elements

		Mode 1	*Mode 2*	*Mode 3*	*Mode 4*	*Mode 5*
		Current (A)	Current (A)	Current (A)	Current (A)	Current (A)
**1.0**	**Element #1**					
	Electronics Box A	0.1	0.1	0.1	0.1	0.1
	Switch #1		0.2			
	Switch #2					

Step 3: Power Profile for Sequence of Operations

		Which Power Mode	Time On (s)	Energy (W)
(1) Attach to Handrail				
	Attach command received			
	Action #1 - (operations in low power mode)	Mode 1	30	0.08
	Drive Element #2	Mode 4	90	0.49

Table 2 - Power Profile Example

Please continue to use the same names of components used in your Component List.

Minimum content requirement: Paste a figure of your filled in tables for the Power Profile into this document. No additional text is required.

7 Exit Survey

To complete your submission, please take the Exit Survey by going to this webpage: https://seasgwu.qualtrics.com/jfe/form/SV_2r9DaeSlh48uMcZ

At the end of the survey you will receive a unique code. In your submission include this section and the text: Exit Survey for Freelancer <<insert Freelancer username>> complete per completion code: <<insert completion code>>.

To be complete, your submission must include the following text: Exit Survey for Freelancer <<insert Freelancer username>> complete per completion code: <<insert completion code>>.
