# Supplementary material for: Linking solver characteristics, solving processes and solution attributes: A data explainer for an open innovation generated robotic design dataset
Source: Data Brief. 2023 Sep 6;50:109547. doi: 10.1016/j.dib.2023.109547 (PMC10518673; doi:10.1016/j.dib.2023.109547)
Supplement: Supplementary file 1 [file mmc1.zip › Release/Process/Challenge Rules/D3-SCA/SCA Problem Description.pdf]

1 Contest Description

In this contest, you are asked to design a "Smart" Coarse-positioning Arm (SCA) that will be mounted to Astrobee. A separately designed mechanism will be mounted to the free end of the SCA, and is referred to as the "End-Effector" in this contest. The End-Effector grabs and holds on to an ISS Handrail when commanded to do by the SCA. The End-effector should be considered as a "black box" that grabs and holds on to a Handrail with defined mechanical, power and data interfaces to SCA. After SCA is securely attached to a Handrail, the SCA rotates Astrobee in two directions.

The SCA receives all power and high-level commands from Astrobee, but implements the following functions autonomously: stowing and deploying from a payload volume, positioning and commanding the End-Effector, and orienting Astrobee by rotating it in two directions.

The details below describe how the SCA should work, its functional requirements and interface constraints/assumptions. A separate document provides detailed guidelines on how your design must be presented and submitted.

A prize of $1,500 will be awarded for the lowest mass, technically feasible solution, submitted before 21:00 GMT on August 15th, 2018.

2 Concept of Operations – How the SCA needs to work

2.1 Normal Operations

The SCA must be able to perform three high-level functions when it receives a corresponding command from Astrobee: Attaching to a Handrail, Orienting the Astrobee, Stowing into the Astrobee payload bay. Each function requires a combination of performing actions itself and commanding the operations of the attached End-Effector. The sequence of operations and commands are described below:

1) Attaching to a Handrail:
    a. <u>Deploy</u>: which involves the SCA unpacking from its *Stowed* configuration in Astrobee's payload bay and moving the End-Effector to a standard *Deployed* location, without contacting Astrobee; then
    b. Sending a command to the End-Effector grab and hold onto a Handrail at a location specified by Astrobee ("EE_attach")
    c. <u>Standby</u>: which involves SCA waiting for a confirmation from the End-Effector that it is attached to the Handrail in a *Secured* configuration; then
    d. Sending a confirmation of achieving the *Secured* configuration to Astrobee.
2) Orienting the Astrobee:
    a. <u>Pan</u>, <u>Tilt</u>: which involves the SCA independently rotating Astrobee in two directions as commanded by Astrobee, without contacting an End-Effector/Handrail Keep Out Zone.
3) Stowing into Astrobee's payload bay:

a. Sending a command to the End-Effector to remove itself from the Handrail ("EE_retract")
b. Standby: which involves SCA waiting for a confirmation from the End-Effector that it is no longer attached to the Handrail, and returning to the *Deployed* configuration; then
c. Stow: which involves SCA packing the SCA and End-Effector back into Astrobee's payload bay (*Stowed* configuration)
d. Sending a confirmation of achieving the *Stowed* configuration to Astrobee.

The relationships among the configurations (italicized), operations (underlined), commands to/from Astrobee (blue dashed arrows), and commands to/from the End-Effector (red solid arrows) are highlighted in Figure 1. The coordinate system and definitions of certain elements are seen in Figure 2. The requirements for each operation, configuration and command are detailed in Section 3.

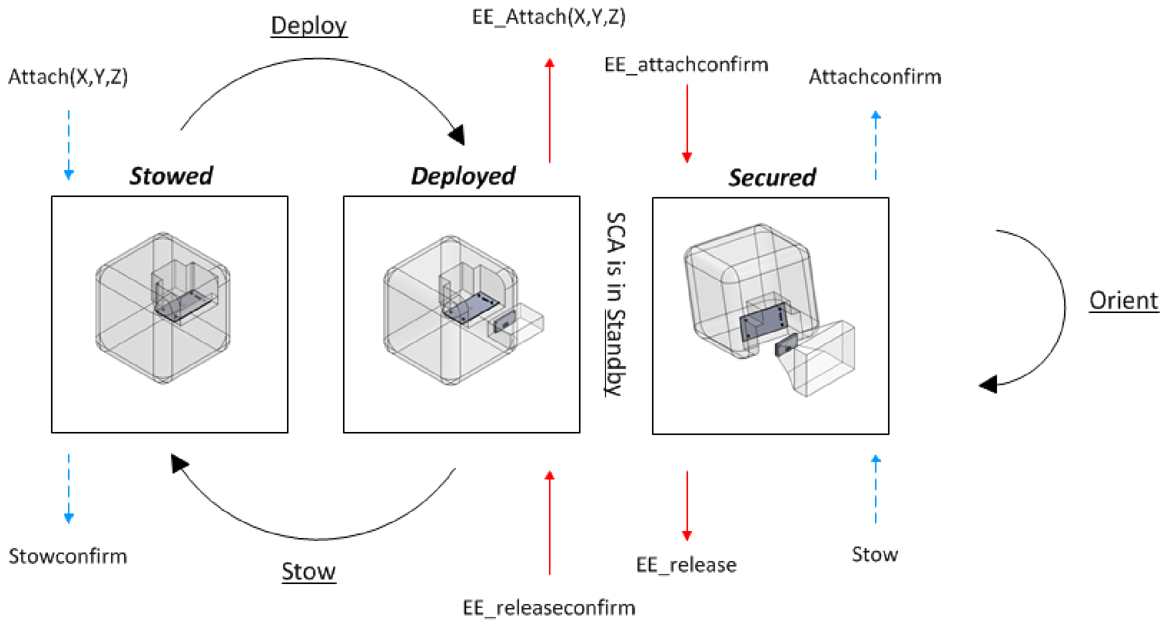

Figure 1 - SCA Concept of Operations

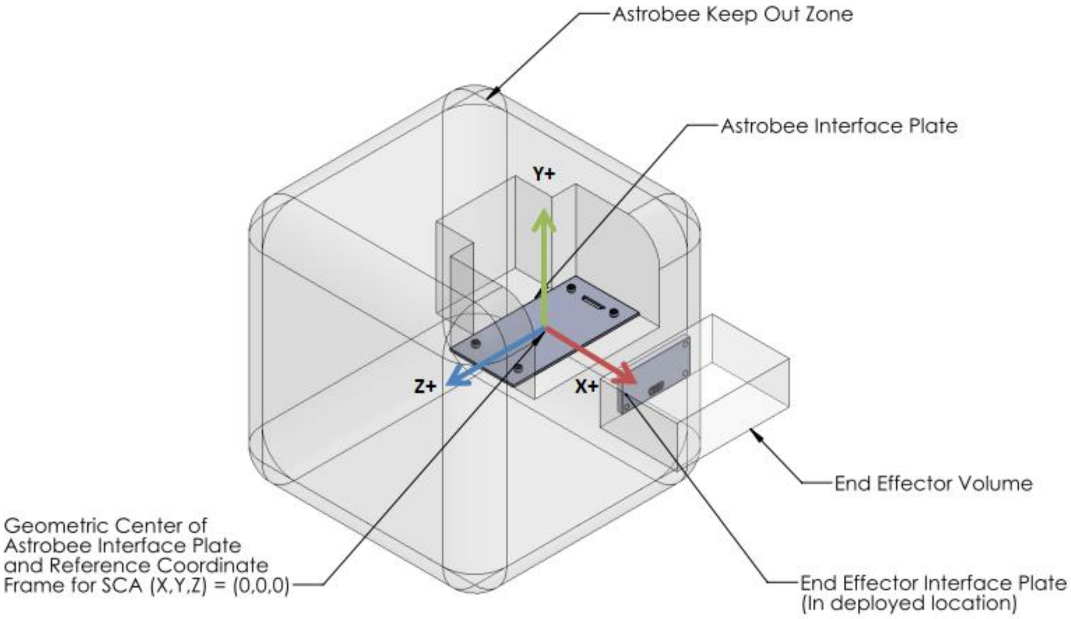

Figure 2 – SCA Coordinate Reference Frame and Mechanical Interfaces

2.2 Contingency (Emergency) Operations

There are several scenarios when normal operations may be disrupted. The ranges of permissible responses are detailed in Section 3.6. This section summarizes those scenarios: (1) when the SCA experiences higher than expected loads while in a *Deployed* configuration (e.g., because an astronaut or other object bumps or contacts Astrobee); and (2) when an astronaut manually pulls on the SCA to remove the system from the handrail.

3 Functional Requirements

This section details all the requirements that the SCA must meet to accomplish the operations described above. To clearly define the motion involved in these operations, we define a coordinate reference frame that has its origin at the geometric center of the Astrobee Interface Plate (see Figure 2). Also illustrated in Figure 3 is the End-Effector in its Deployed configuration. Since the End-Effector is treated as a "black box" in your SCA design, we show only its overall volume and its required mounting interface plate.

3.1 Motion Requirements

R1 Deploy: The SCA shall be able to move from the Astrobee payload bay (C1.2) and place the End-Effector at the *Deployed* location and orientation without contacting Astrobee (Astrobee Keep Out zone defined in C1.1). The coordinates for the *Deployed* location is shown graphically in Figure 3.

R1.1 Deployed Location and Orientation: The End-Effector Interface Plate (C12) shall be placed at the following position with no more error than:

- x = 134 mm $\pm$ 5 mm
- y = 20 $\pm$ 5 mm
- z = 0 $\pm$ 5 mm
- θ_x = 0 $\pm$ 5 degrees (rotation around the x axis)
- θ_y = 0 $\pm$ 5 degrees (rotation around the y axis)
- θ_z = 0 $\pm$ 5 degrees (rotation around the z axis)

Orientation of the End-Effector Interface plate should be as shown in Figure 3, with the face of the plate parallel with the front of Astrobee.

R1.2 The SCA and attached End-Effector shall maintain a minimum clearance of 2 mm from Astrobee while Deploying. Key dimensions of Astrobee are included in Section 4 (C1.1).

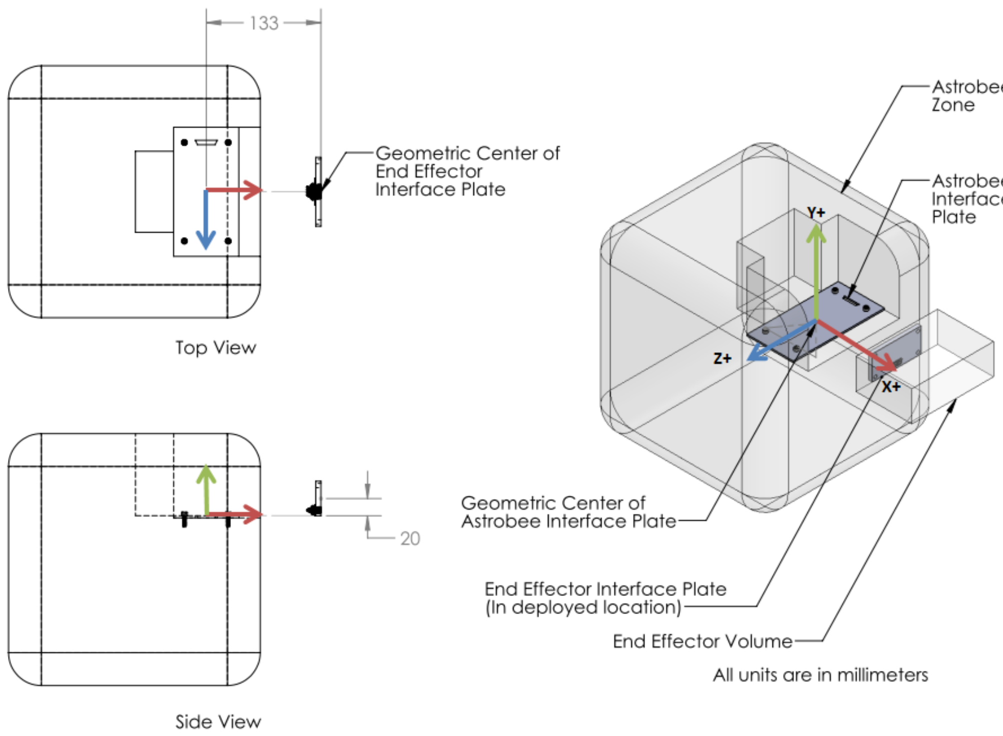

Figure 3 - Deployed Location and Orientation of End-Effector Interface Plate

R2 Orient: When commanded by Astrobee, the SCA shall be able to Pan and Tilt (rotate in two perpendicular directions) Astrobee about the geometric center of the End-Effector Interface Plate. Assume that the SCA is fixed to the Handrail through the End-Effector (i.e. the *Secured* configuration) during all Orienting operations.

R2.1 During panning and tilting the SCA and Astrobee shall not contact the End-Effector Keep Out Zone (Figure 4)

R2.2 Pan and Tilt coordinates are measured with respect to the End Effector Interface Plate coordinate system shown in Figure 5.

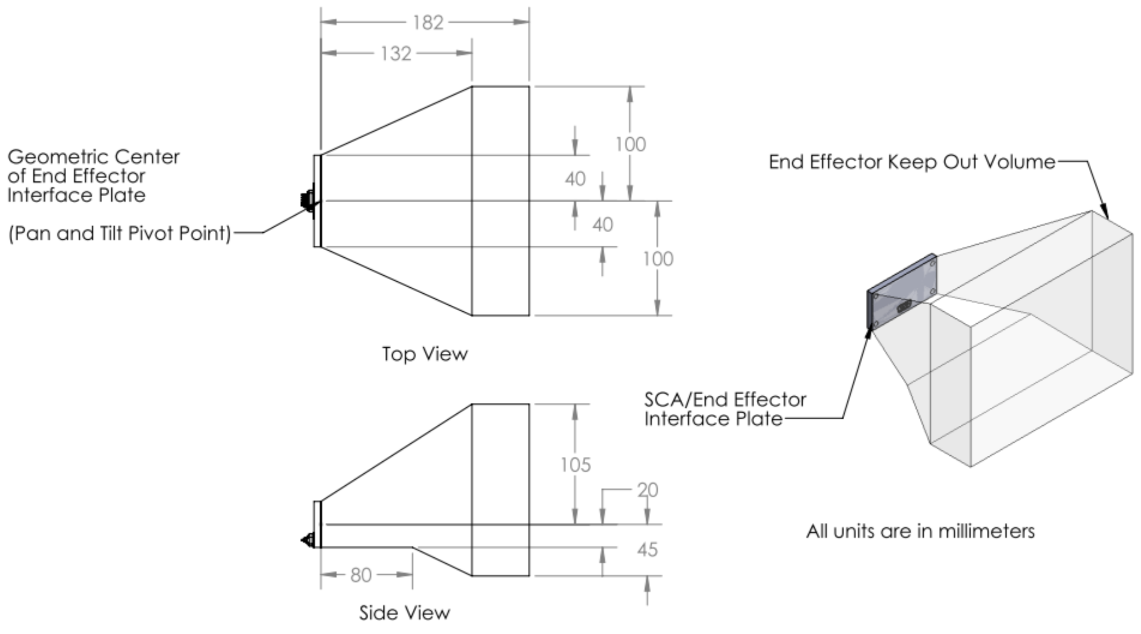

Figure 4 – End-Effector Keep Out Volume

R2.3 Rotation for Pan and Tilt occurs about the center of the End-Effector Interface Plate (C12). Translation of the Astrobee is acceptable, along with rotation, if Astrobee's final position is rotated about the center of the End-Effector Interface Plate. (See Figure 6)

R2.4 Minimum clearance between the moving components of SCA and Astrobee and the End-Effector Keep Out Volume during Pan and Tilt movements is 2 mm.

R2.5 Pan: The SCA shall be able to rotate through the range: -65° < θ_x < 65° , +/- 5° as detailed in Figure 5 .

R2.6 Tilt: the SCA shall be able to rotate through the range: 0° < θ_y < 90° , +/- 5° as detailed in Figure 5.

R2.7 No simultaneous panning and tilting is required.

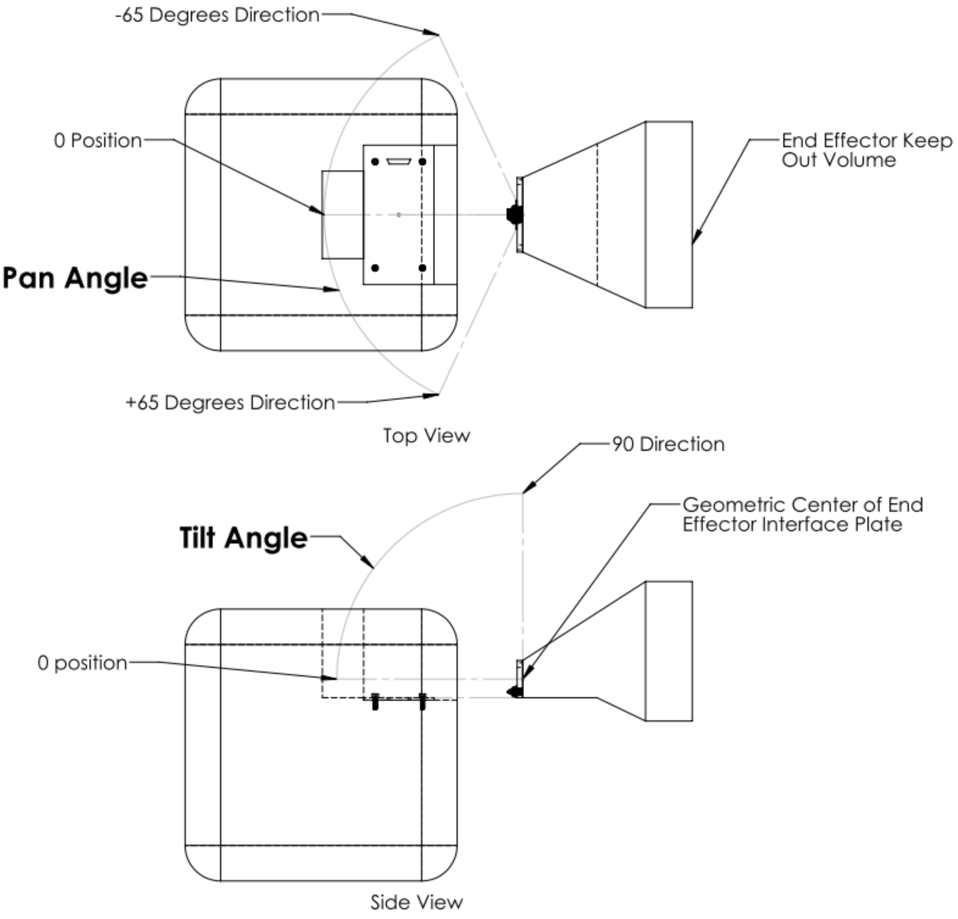

Figure 5 - Astrobee Orienting Operations: Pan Range of Motion (top), Tilt Range of Motion(bottom)

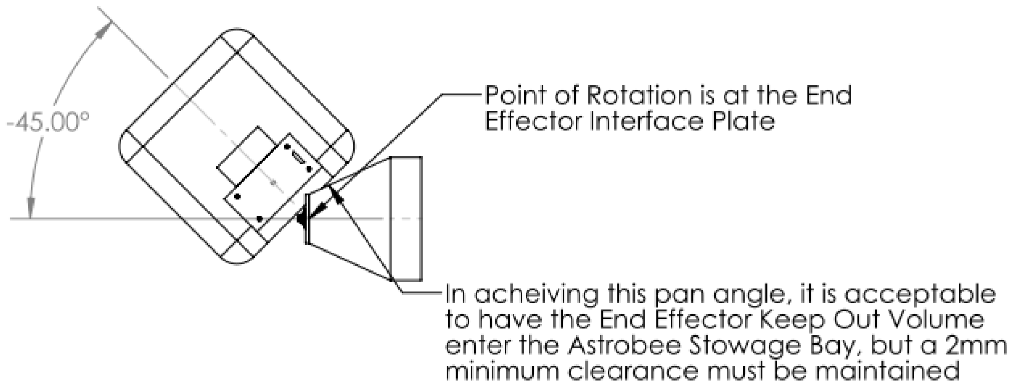

Figure 6 – Illustration of acceptable Astrobee motion during Pan operations

R3 Stow: The SCA shall be able to return itself (with End Effector attached) to a *stowed* configuration, fitting completely within Astrobee's payload bay (see C1.2).

R4 Standby: The SCA shall be able to enter a standby mode that uses minimal power as it waits to receive confirmation messages from the End-Effector or commands from Astrobee.

3.2 Control Requirements

The format of all commands sent by the Astrobee is specified in section C10. This section describes how the SCA shall be controlled. The SCA only receives four high-level commands from Astrobee and must execute the rest of its operations and interactions with the End Effector autonomously. The SCA is also responsible to command the attached End Effector to change its configurations and eventually attach (and release) a Handrail.

R5 When commanded to "attach(x,y,z)" the SCA shall perform the following sequence without further instruction from Astrobee:

1. The SCA shall initiate Deploy (R1).
2. Once the SCA places the End Effector Interface Plate at the *Deployed* location, *(*R1.1), the SCA shall:
   a. command the End Effector to "EE_attach (x, y, z)" (C20) and
   b. enter Standby (R4) until it receives a "EE_attachconfirmed" signal from the End Effector (C20) and then:
   c. The SCA shall send a "attachconfirmed" signal to Astrobee and
   d. Enter Standby (R4) until it receives another command from Astrobee.

R6 When commanded to "pan(θ_x)" the SCA shall:

1. The SCA shall complete the specified rotation θ_x(R2)
2. When Panning is complete:
   a. the SCA shall send a "panconfirmed" signal to Astrobee and
   b. enter standby (R4)

R7 When commanded to "tilt (θ_y)"

1. The SCA shall complete the specified rotation θ_y (R2)
2. When Tilting is complete:
   a. the SCA shall send a "tiltconfirmed" signal to Astrobee and
   b. enter standby (R4)

R8 When commanded to "stow," the SCA shall perform the following sequence without further instruction from Astrobee:

1. The SCA shall:
   a. Send a "EE_retract" command to the End Effector (C20) and
   b. enter Standby (R4) until SCA receives a "EE_retractconfirmed" signal(C20) from the End Effector and then
   c. Stow (R3) in Astrobee's payload bay and
   d. send a "stowconfirmed" signal to Astrobee.

3.3 Resource Requirements

3.3.1 Timing Requirements

R9 Attaching operations (R5) shall not exceed a total of 15 minutes. The timing for attach is measured from Astrobee's command to "attach(X, Y, Z)" to SCA's transmission of "attachconfirmed" to Astrobee. This includes up to 7.5 minutes of standby while the End Effector attaches to the ISS Handrail.

R10 Time to Orient:
a. The SCA shall be able to Pan 30 degrees in 5 seconds.
b. The SCA shall be able to Tilt 30 degrees in 5 seconds.
c. Combined pan and tilt operations shall not exceed 1 hour.

R11 Stowing operations (R8) shall not exceed 15 minutes. The timing for stow is measured from the Astrobee's command to "stow" to the SCA's transmission of "stowconfirmed" to Astrobee. This includes up to 7.5 minutes of standby while the End Effector releases from the Astrobee and stows in the End Effector Volume.

R12 Total time in Standby shall not exceed 15 minutes.

3.3.2 Energy Requirements

All power transmitted to SCA and the End Effector is described in the interface Section 4. Below are total energy limits on SCA operations.

R13 The SCA shall not use more than 24 Watt-hours of energy for all its operations: Attaching, Orienting and Stowing. In calculating your energy budget, assume maximum durations for Pan, Tilt and Standby as described in R9 through R12. The time for Deploy and Stow are functions of your design.

3.4 Safety Requirements

R14 The SCA shall have no sharp edges, defined as a radius of 3 mm, for astronaut safety.

R15 The SCA shall have no loops of material greater than 25.4 mm in diameter for astronaut safety, and no unsupported or unattached material more than 40 mm from the structure of the SCA.

R16 The SCA shall not damage itself through normal operations.

R17 The SCA shall be able to return to its normal operations if power is momentarily lost.

3.5 Environmental Requirements

R18 The SCA shall operate in the ISS zero gravity environment.

R19 The SCA, when unpowered, shall not be damaged by electrostatic discharge <4,000V.

R20 The SCA shall operate in an atmosphere comparable to that of Earth. Assume temperature of 21 °C, and pressure of 101 kPa [1 atm], and relative humidity that is 40% - 70%.

R21 The SCA shall not contribute any particulates (e.g. dust) to the ISS atmosphere.

R22 The SCA shall enclose all lubricated components to prevent lubricants from leaking into the atmosphere of the ISS.

3.6 Contingency Requirements

R23 Excessive loads: This scenario may occur if an astronaut or piece of equipment contacts Astrobee while the SCA is in the Deployed configuration (including while experiencing normal operating loads). The SCA shall maintain normal orient operations when subject to a force of up to 18N applied at the baseplate in the negative Y-direction and a simultaneous moment of 1.8 Nm about the Z-axis (see Figure 7a).

R24 Astronaut intervention: Astrobee is required to be removable from the Handrail by an astronaut. with a pull-away force of 35.6 N. This force is applied in the negative X-direction applied through the baseplate (see Figure 7b), with the End-Effector

fixed in space. This will not occur during any other operation.

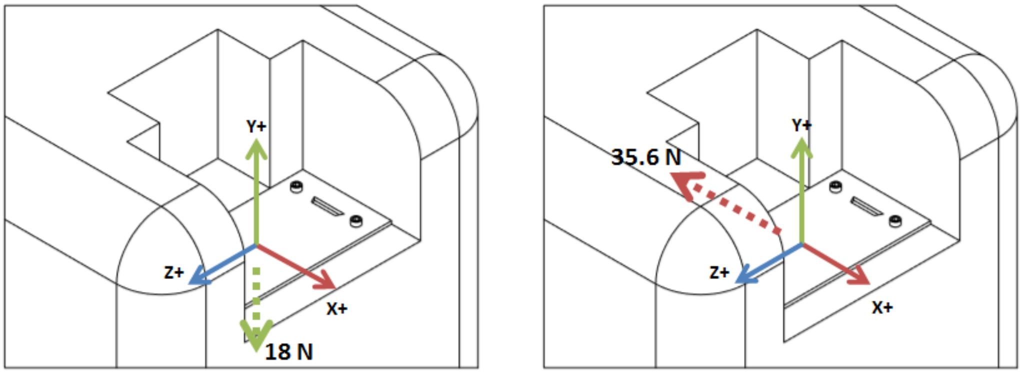

Figure 7 – Contingency Cases: Excessive Loads (left); Astronaut intervention (right)

4 Interface Requirements

The SCA is fixed to both Astrobee and the End-Effector, each with its own interface. This section describes all constraints imposed by those interfaces.

4.1 SCA-Astrobee Interface

4.1.1 SCA-Astrobee Mechanical Interface

C1 Constraint 1 (C1) Volume Constraint: The SCA and End Effector are stowed in Astrobee's payload bay. Figure 8 defines the payload bay with respect to Astrobee

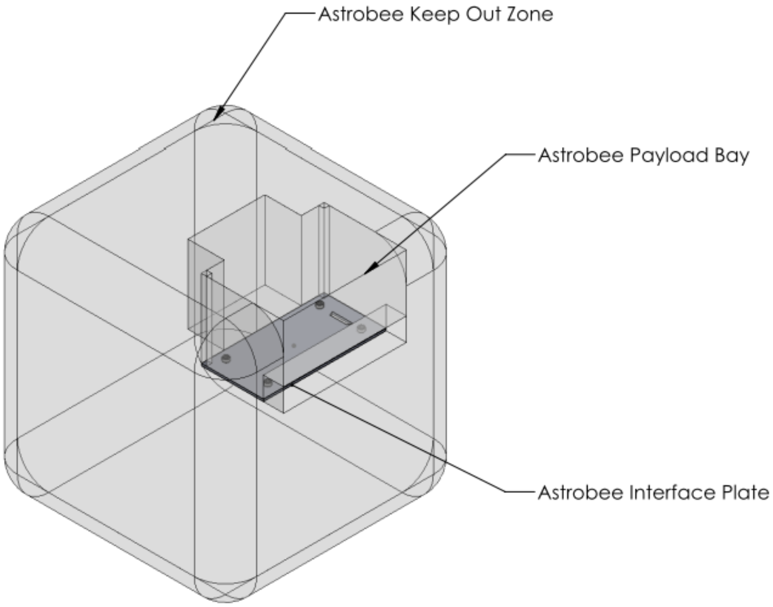

Figure 8 - Astrobee and Payload Bay

C1.1 Figure 9 defines the dimensions of the Astrobee Keep Out volume that must not be contacted while operating.

C1.2 Figure 10 defines the dimensions of the payload bay, which the SCA (with attached End-effector) must fit within when in the *Stowed configuration*.

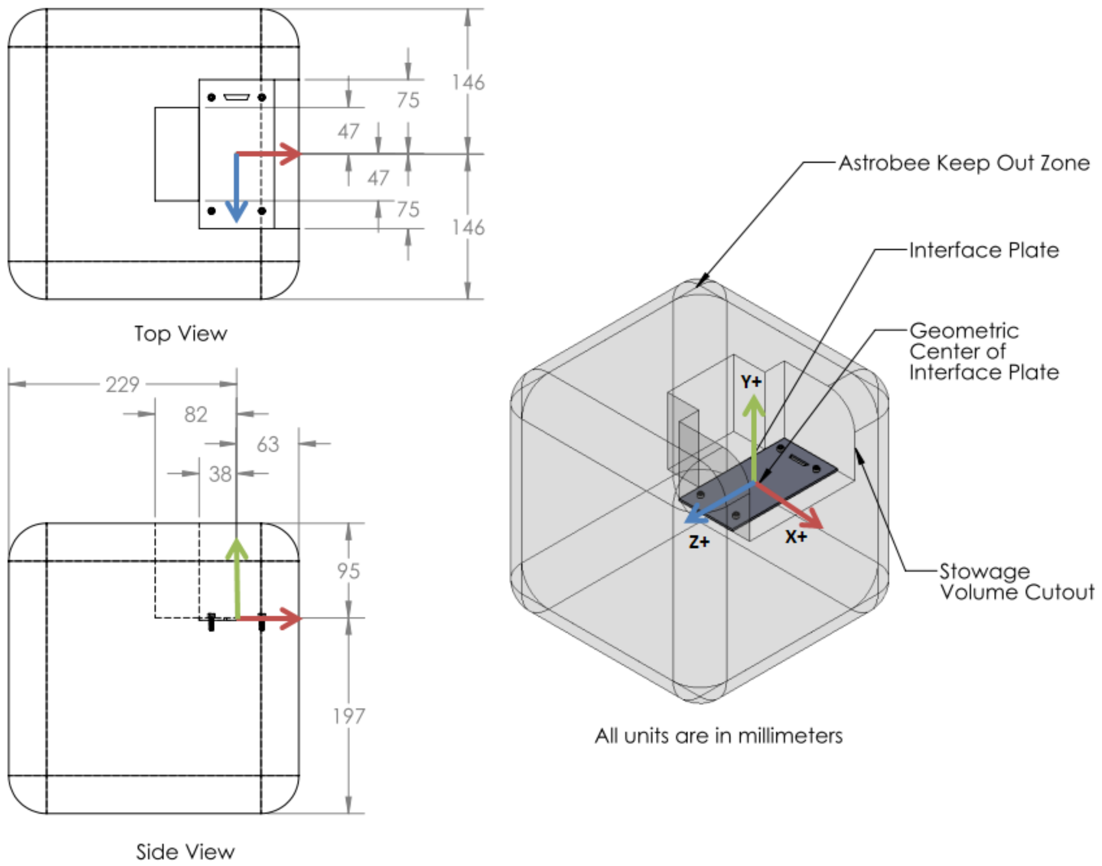

Figure 9 - Astrobee Keep-Out Zone

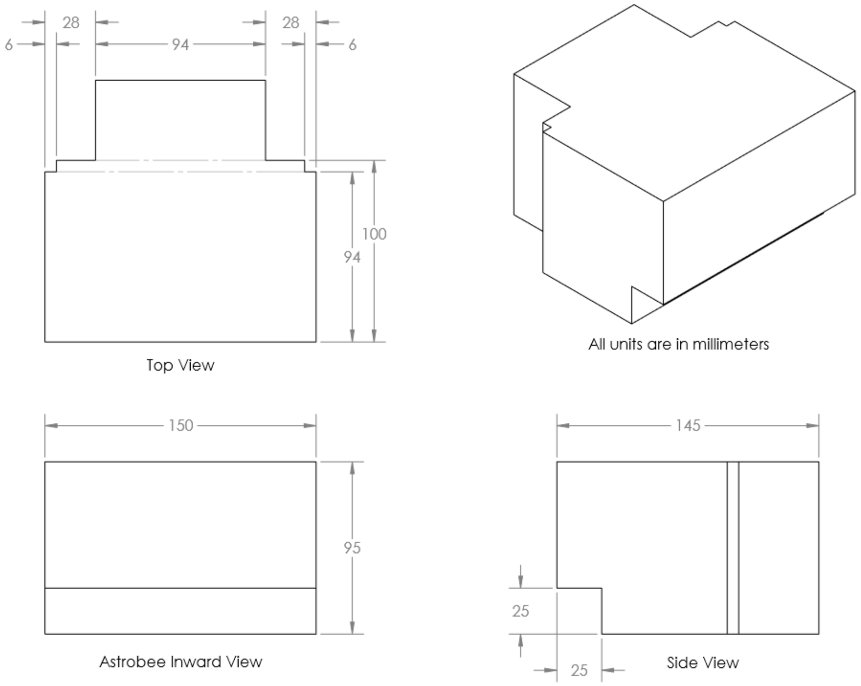

Figure 10 – SCA (+ End-Effector) Stowage Volume

C2 Mounting interface: The SCA shall mechanically mount to a flat metal plate shown in Figure 11.

- C2.1 All external loads (Section 3.6) are applied at the interface plate.
- C2.2 There are four available screw holes in the specified location.
- C2.3 Screws for your selected electrical connectors must only require hand tightening of locking screws of the adapter. Assume that no external loads are applied through the electrical connector.

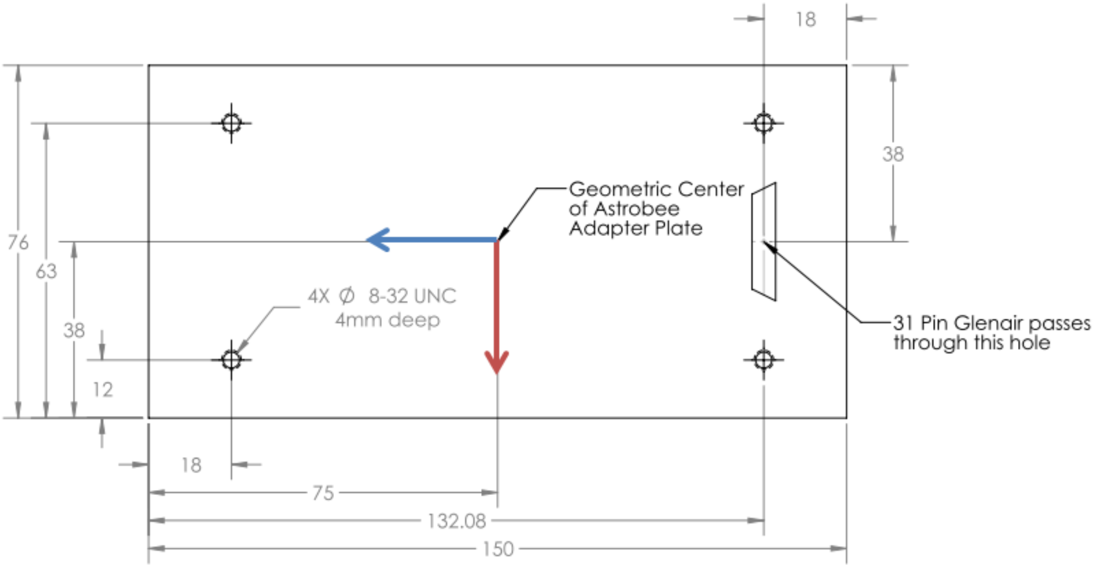

Figure 11 - SCA mounting interface

C3 Astrobee mass Properties: Treat Astrobee as mass of 6 kg with center of mass at (-83mm, -48 mm, 0 mm) relative to the SCA Coordinate Reference Frame (Figure 2 – SCA Coordinate Reference Frame and Mechanical Interfaces)

4.1.2 SCA-Astrobee Power Interface

C4 The SCA shall connect to the power and data connector shown in Figure 11. It is a 31-pin payload Glenair M83513/03-E03N connector located in the baseplate.

C5 Astrobee Supply Voltage, $+V_s$, is normally 14.4 V DC, but can vary between 11 to 17 V DC.

C6 Max Current: The SCA shall not draw more than 3 A peak.

C7 Steady State Current: The SCA shall not draw more than 2 A at steady state.

C8 Pinout is as follows: Pin 4 is supply voltage, V_s. Pin 1 is ground.

4.1.3 SCA-Astrobee Data Interface

C9 All commands will be received and transmitted as part of a serial command using the second and third pins of the 31-pin connector. The second pin is the positive serial command line, and the third pin is the negative serial command line.

C10 Commands (received and transmitted) shall be serial and formatted in ASCII using the RS-232 protocol once. They are specified in Table 1.

Table 1 – Command format

Command format	Action
Received from Astrobee to the SCA	
"attach(x,y,z)"	Initiate deploy (R1,R5)
"pan(θ_X)"	Initiate pan for specified degrees (R2, R6)
"tilt(θ_y)"	Initiate tilt for specified degrees (R2, R7)
"stow"	Initiate stow (R3, R8)
Sent from SCA to Astrobee	
"attachconfirmed"	Confirm deploy has completed (R1,R5)
"panconfirmed"	Confirm pan has completed (R2, R6)
"tiltconfirmed"	Confirm tilt has completed (R2, R7)
"stowconfirmed"	Confirm stow has completed (R3, R8)

4.2 SCA-End Effector Interface

4.2.1 SCA-End Effector Mechanical Interface

C11 End Effector Volume: The End-Effector occupies a volume that can be treated as a black box. The End-Effector Volume is as 92 mm x 134 mm x 50 mm (Figure 12)

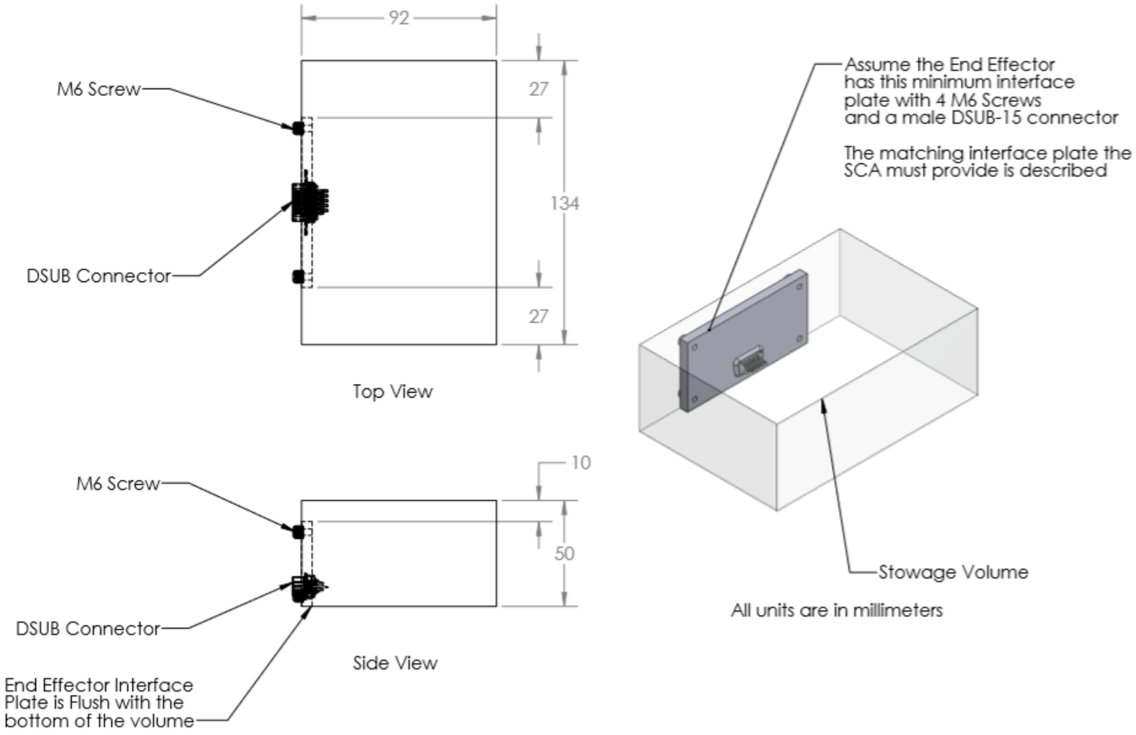

Figure 12 - End Effector Mechanical Interface

C12 End Effector Mounting Interface Plate: The free end of the SCA shall provide the interface plate shown in Figure 13 to mechanically mate to the End-Effector.

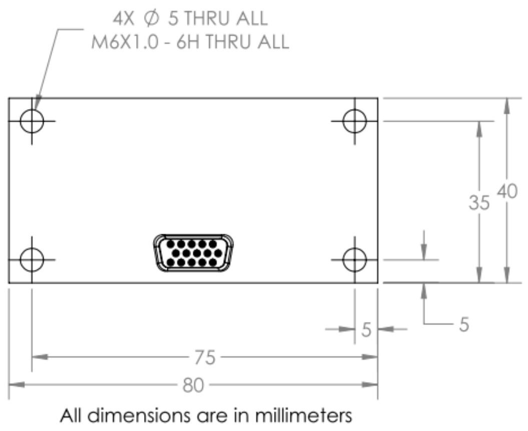

Figure 13 - End Effector Interface Plate

C13 Mass Properties: Treat the End Effector as mass of 2 kg with its center of gravity at the geometric center of its defined Volume (Figure 12). You can assume the center of gravity is the same during all operations.

4.2.2 SCA-End Effector Power Interface

C14 The SCA shall power and command the End Effector through the electrical connector shown in Figure 14. It is a standard D-Subminiature High Density 15 pin connector (DSUB-15).

C15 Supply voltage, $+V_S$: The SCA shall provide +14.4 V DC, varying between 11 V to 17 V DC, to the End-Effector.

C16 Max current: The SCA shall accommodate a max current draw of up to 3 A peak when the End Effector is commanded to operate.

C17 Steady State Current: The SCA shall accommodate a steady state current draw of up to 2 A when the End Effector is commanded to operate.

C18 Pin out is as follows: Pin 4 is $+V_S$, supply voltage. Pin 1 is ground.

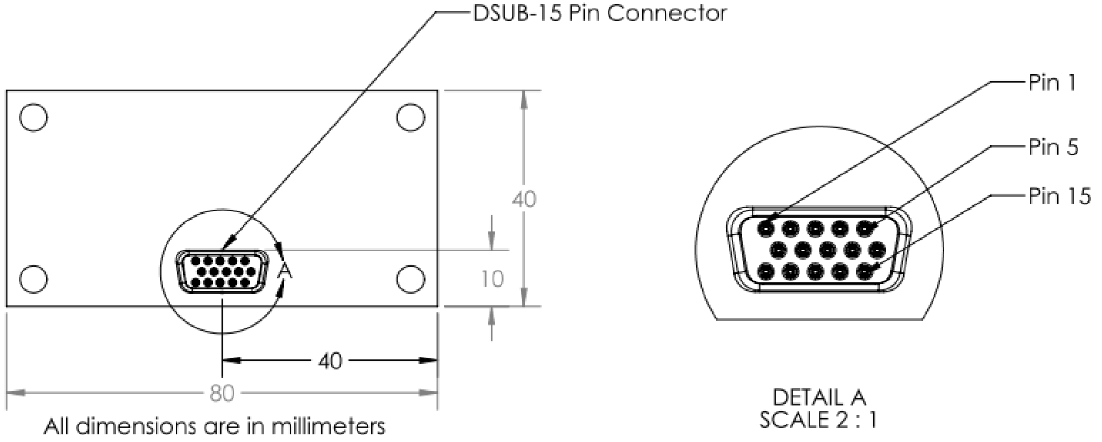

Figure 14 - Interface Plate Data and Electrical Connections

4.2.3 SCA-End Effector Data Interface

C19 All commands will be received and transmitted as part of a serial command using the second and third pins. The second pin is the positive serial command line, and the third pin is the negative serial command line.

C20 Commands (received and transmitted) shall be serial and formatted in ASCII using the RS-232 protocol once. They are specified in Table 2.

Table 2 – Command and Confirmation formats

Command	Action
**(1) Sent from SCA to End Effector**	
"EE_attach(x, y, z)"	Initiate attach to Handrail at the specific location (x,y,z)
"EE_retract"	Initiate retract operation
**(2) Sent from End Effector to SCA**	
"EE_attachConfirmed"	Confirm attach has completed
"EE_retractConfirmed"	Confirm retract has completed
