# Supplementary material for: Linking solver characteristics, solving processes and solution attributes: A data explainer for an open innovation generated robotic design dataset
Source: Data Brief. 2023 Sep 6;50:109547. doi: 10.1016/j.dib.2023.109547 (PMC10518673; doi:10.1016/j.dib.2023.109547)
Supplement: Supplementary file 1 [file mmc1.zip › Release/Process/Challenge Rules/D4-RASA/RASA Problem Description.pdf]

# 1 Contest Description

In this challenge, you are asked to design the Robotic Arm Software Architecture (RASA) that will control a Robotic Arm (RA) that has been separately designed to attach the Astrobee Robotic Free Flyer to a Handrail within the International Space Station (ISS), and orient Astrobee in two perpendicular directions. The RASA will receive high-level commands from Astrobee and implement them through an electronics suite to control the RA.

Section 2 describes the overall system and how your RASA is expected to operate. Section 3 provides details on the desired software functionality, and Section 4 describes the software and hardware interfaces your RASA design should support. A separate document provides guidelines on how your RASA design must be presented and submitted.

Note that no executable code is required, but your proposed software architecture must be sufficiently descriptive to allow experts to assess its feasibility (i.e., comply with all the requirements) and follow the prescribed format.

***Challenge Rules:*** A prize will be awarded for the **most efficient, technically feasible** design,**.** Design efficiency will be evaluated based on the lowest, credible estimated lines of code for implementing a solution.

# 2 How the RASA needs to work

The Robotic Arm Software Architecture will be used to develop software that controls a Robotic Arm when given high-level commands by Astrobee. The specific, high-level commands your RASA must receive and execute are described in detail below (Section 5, Table 1).

The Robotic Arm reference design in this contest has 7 independent degrees of freedom, each driven by its own motor and gearbox (or “actuator”), and controlled with data from a variety of sensors. Six of the seven Robotic Arm actuators are used to position the end of the RA, and the seventh (“Grasp” actuator) is used to hold on securely to the Handrail. You may assume the RA hardware and all its associated control electronics hardware have been already designed. Details on the functionality and interfaces to the RA control electronics hardware are included in Section 4.1.

The Robotic Arm’s control electronics suite contains the processing components on which your software will run in real time. All low-level, hardware-dependent driver software are also designed elsewhere, and a description of these software modules are included in Section 5.1, C3. Hardware-dependent software includes all drivers for controlling each actuator, sensors that provide knowledge of actuator state (such

as position, speed or torque), and two additional sensors that provide knowledge of where the Grasp Tool is with respect to the Handrail.

# 3 Functional Requirements

The RASA must be able to control the Robotic Arm to perform five primary functions: translating 4 high-level motion commands from Astrobee (R1-R5), and a power monitoring for when the Robotic Arm is active (R6). Key requirements for achieving these functions isdescribed below

## 3.1 Normal Operations

Under normal conditions, the RA will respond to 4 motion commands from Astrobee (specific format is summarized in Table 1, Section 5):

R1 **Attach(x,y,z)**: this command secures Astrobee to the desired handrail by moving the RA to a position close to the Handrail, and holding onto it with a "Grasp" tool by:
(1) positioning the robotic arm Near a handrail at the position (X, Y, Z) by using the Motion Planning Software Module, MoveTool(X, Y, Z). (See Section 5.1, C4 for details on the Motion Planning Software Module)
(2) grasping the handrail by Driving the A7/Grasp actuator to a closed position by moving at 2 RPM until the contact Switch (sensor, S2_contact) is "on".

R2 **Pan ($\theta_x$):** When *attached* to a ISS Handrail, this command rotates Astrobee about the ISS Handrail by rotating A6/Pan actuator to the position = $\theta_x$ [degrees]

R3 **Tilt ($\theta_y$ ):** When *attached* to a ISS Handrail, this command rotates Astrobee about the ISS Handrail by rotating A6/Tilt actuator to the position = $\theta_y$ [degrees]

R4 **Stow (X,Y,Z)**: This command disengages the Robotic Arm from the handrail, and returns it to the Astrobee payload bay from a position in space, (X, Y, Z) by:
A) releasing Handrail by driving grasp actuator to open position (+45°)
B) moving the robotic arm from near a Handrail at (X, Y, Z) by using the Motion Planning Software Module, StowTool(X, Y, Z)

R5 **Confirmation**: After each commanded motion is accomplished, a "confirm" message should be sent to Astrobee. Specific format of the confirmation message is identified in Table 1 below.

In addition to the four motion commands described above, RASA has an additional high-level function related to system power:

R6 **Power and Energy Monitoring**: The RASA is expected to provide Astrobee with up to date estimates of instantaneous power consumption and aggregate energy usage from the time the RA is deployed (starting with an Attach command) until it is Stowed in the Astrobee Payload bay again. When the RA is stowed in the payload bay again, energy usage total should be reset.

# 4 Hardware Interfaces

The RASA has physically interfaces to the robotic arm through a control electronics hardware suite. The section describes constraints imposed by those interfaces.

## 4.1 System Power and Data Block Diagram

As described above, for your RASA design, you may assume a generic suite of motion control electronics hardware. Figure 1, below, captures the primary elements of the control electronics suite that may be used by RASA for Robotic Arm motion planning. And specifically, we expect your RASA to run on the Realtime Processing Unit (RPU).

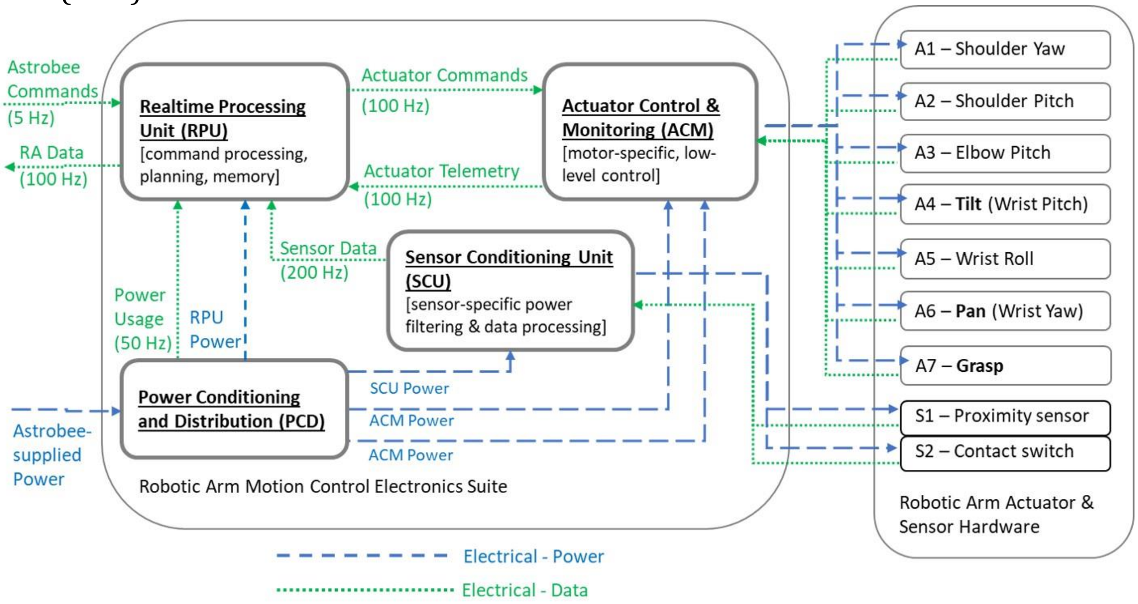


**Figure 1. RA Control Electronics Suite**

# 5 Software Interfaces

The RA control suite interfaces with Astrobee, and also contains the software drivers for lower-level, hardware-specific motion control elements such as joint motors and position sensors.

We are not providing a particular API for the individual modules of the RA motion control, sensor or power conditioning electronics (ACM, SCU, PCD from Figure 1), however we provide software interface information in this section. This includes specific commands, telemetry streams, function libraries, etc.

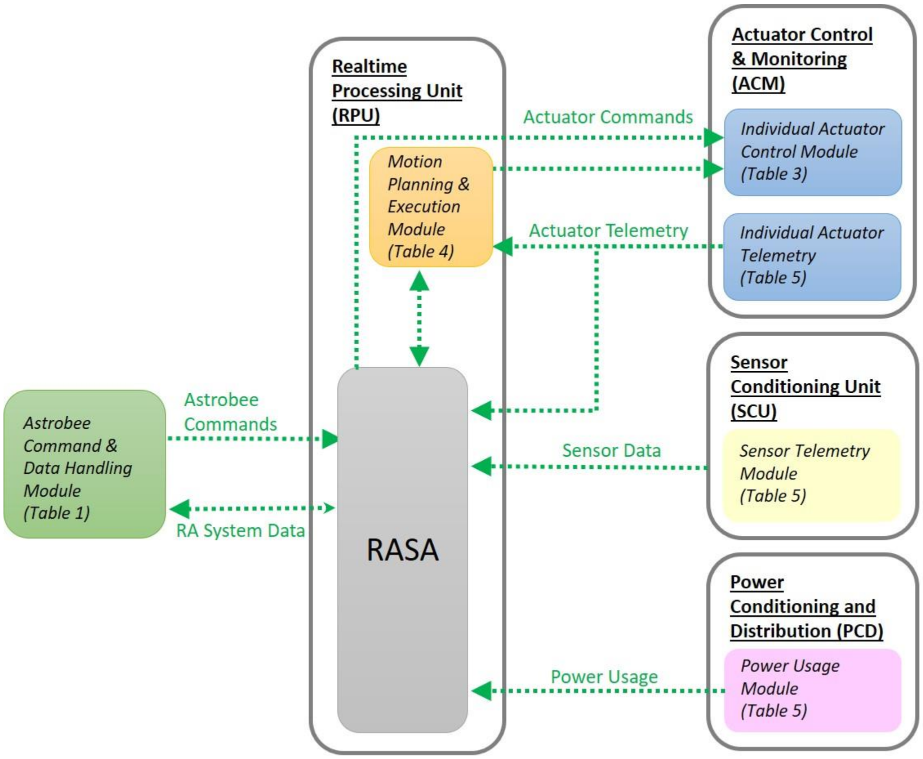


**Figure 2. Software Interfaces to RASA**

### C1 **Astrobee Command & Data Interface**

RASA interfaces to Astrobee through serial commands, formatted in ASCII, and specified in Table 1.

**Table 1. Format for commands to/from Astrobee**

| **Command format** | **Action** |
|---|---|
| *Sent from Astrobee, to RASA* | |
| "attach(x,yz)" | Moves RA to Handrail location, (x,y,z) |
| "pan($\theta_x$)" | Rotate Astrobee in the "pan" direction for specified degrees |
| "tilt($\theta_y$)" | Rotate Astrobee in the "tilt" direction for specified degrees |
| "stow(x,y,z)" | Return RA to Starting Pose in Payload bay from Handrail at (x,y,z) |
| *Sent from RASA, to Astrobee* | |
| "attachConfirmed" | Confirm attach has completed |
| "panConfirmed" | Confirm pan has completed |
| "tiltConfirmed" | Confirm tilt has completed |
| "stowConfirmed" | Confirm stow has completed |

## 5.1 Actuator Control Modes

This section describes the performance limits of each actuator and functional modes available to drive the 7 actuators in the RA through the ACM. This includes individual motor control as well as multi-motor, coordinated motion (Motion Planning Software Module)

C2 **Individual actuator control properties** – Range of Motion (or permissible actuator positions), acceptable speeds, and control uncertainties are summarized in Table 2.

**Table 2 - Actuator Control Details**

| Actuator Number/Name | Position Range | Position Uncertainty | Speed Range | Speed Uncertainty | Average Power |
|---|---|---|---|---|---|
| A1/Shoulder Yaw | -90° ↔90° | ±1.5 deg | 0 – 3 rpm | ±0.5 deg/s | 0.5 W |
| A2/Shoulder Pitch | -180° ↔ 90° | ±1.5 deg | 0 – 3 rpm | ±0.5 deg/s | 0.5 W |
| A3/Elbow Pitch | -180° ↔ 180° | ±1.5 deg | 0 – 3 rpm | ±0.5 deg/s | 0.5 W |
| A4/Tilt (Wrist Pitch) | -165° ↔ 165° | ±1.5 deg | 0 – 5 rpm | ±0.25 deg/s | 0.3 W |
| A5/Wrist Roll | -180° ↔ 180° | ±1.5 deg | 0 – 5 rpm | ±0.25 deg/s | 0.3 W |
| A6/Pan (Wrist Yaw) | -180° ↔ 180° | ±1.5 deg | 0 – 5 rpm | ±0.25 deg/s | 0.3 W |
| A7/Grasp | -45° ↔ 45° | ±1.5 deg | 0 – 2 rpm | ±0.25 deg/s | 0.3 W |

## C3 Individual Actuator Control Software Module

Table 3 summarizes the single-actuator modes of operation available to RASA for control of the RA through commands to the ACM. These low-level actuator control modes do not return any values indicating success or failure. For your RASA design, you may assume faults or errors from the ACM, PCD or Astrobee are handled elsewhere.

**Table 3 - Actuator Motion Control Modes**

| Control Mode | Description | Inputs to ACM | Format for ACM input | Example for actuator, A1 |
|---|---|---|---|---|
| Velocity mode | Drives actuator at specified velocity for a specified time | Direction (CW, CCW)*; Speed (deg/s); Total time (s); | A**X**_vel_(direction, speed, total time) | A1_vel_(CCW, 1.3, 65) |
| Position mode | Drives actuator to a specified position at a specified velocity | Position (deg); Velocity (deg/s); | A**X**_pos_(position, velocity) | A1_pos_(42, 0.7) |

*CW= Clockwise; CCW = Counter Clockwise

## C4 Motion Planning & Execution Software Module

This software module runs on the RPU, generating valid, multi-actuator motion sequences ofmotion commands that are sent to ACM in order to move the RA. A summary of Motion Planning Software Module commands is in Table 4 below.

(1) <u>MoveTool(x,y,z):</u> This command is intended to move the Robotic Arm from a stowed configuration in Astrobee's Payoad Bay to an extended position that places the free end (the "Tool") of the RA at a specific point and orientation near the Handrail without making contact with other Astrobee components, or other parts of the ISS.

(2) StowTool(x,y,z): This command is intended to move the Robotic Arm back to a stowed position within the Astrobee Payload Bay from a position at the

Handrail located at (x,y,z), while avoiding contact with other Astrobee components, or other parts of the ISS.

Note that the RA Motion Planning Software Module includes a collision avoidance capability, ensuring that MoveTool and StowTool do not make contact with any objects in the workspace while moving.

**Table 4 – Format for commands to/from Motion Planning Software Module**

| Command format | Action |
|---|---|
| *Sent to Motion Planner* | |
| "moveTool(x,yz)" | Moves RA to Handrail location, (x,y,z) |
| "stowTool(x,y,z)" | Return Robotic Arm to Starting Pose in Payload bay from Handrail at (x,y,z) |
| *Received from Motion Planner after completion* | |
| "MoveToolConfirmed" | Confirm moveTool operation has completed |
| "stowToolConfirmed" | Confirms stowTool operation has completed |

## C5 Telemetry Interfaces

Data types identified in Power & Data Block Diagram above (Figure 1) are described in more detail in this section.

Data available for your RASA design from all actuators and dedicated sensors in the ACM, PCD and SCU are summarized below in Table 5. In the table, details are given for Actuator 1 as an example; data and format for all other actuators is available for your RASA design with a change in the actuator number. For example, the data stream name for the position for Actuator 4 would be, "A4_pos".

**Table 5 - Actuator Telemetry, Sensor & Power Usage Data**

| Data Stream Name | Description | Units/Format | Source | Rate (Hz) |
|---|---|---|---|---|
| A1_pos | Actuator #1 output position | degrees | ACM | 100 |
| A1_spd | Actuator #1, speed | deg/s | ACM | 100 |
| A1_dir | Actuator #1, direction | CW or CCW | ACM | 100 |
| A1_power | Actuator #1, power | W | PCD | 50 |
| ACM_power | ACM module power usage | W | PCD | 50 |
| RPU_power | RPU module power usage | W | PCD | 50 |
| PCD_power | PCD module power usage | W | PCD | 50 |
| SCU_power | SCU module power usage | W | PCD | 50 |
| S1_dist | Distance Sensor output | mm | SCU | 200 |
| S2_contact | Contact Switch position | On or Off | SCU | 200 |
