# Supplementary material for: Linking solver characteristics, solving processes and solution attributes: A data explainer for an open innovation generated robotic design dataset
Source: Data Brief. 2023 Sep 6;50:109547. doi: 10.1016/j.dib.2023.109547 (PMC10518673; doi:10.1016/j.dib.2023.109547)
Supplement: Supplementary file 1 [file mmc1.zip › Release/Process/Challenge Rules/D4-RASA/RASA Submission Guidelines.pdf]

Submission Guidelines for the Robotic Arm Software Architecture (RASA)

In this contest, you were asked to design a Robotic Arm Software Architecture that will be deployed on a hardware environment with associated designed Robotic Arm.

This document provides detailed guidelines on how you must describe and present each aspect of your design in order to be considered for the prize. This document looks long but very little text is required. Your submission document must include each of the sections detailed below and all of the information requested in each. Some examples are provided to clarify what constitutes a complete solution.

Use the exact section and subsection header words, shown below.

Submission Guidelines for the Robotic Arm Software Architecture (RASA) 1
1 Design Description 1
1.1 Narrative (word) description of Robotic Arm Software Architecture 1
1.2 Logical Flow Diagram 2
1.3 Functional Analysis 3
2 Design Implementation 4
3 Exit Survey 4

1 Design Description

1.1 Narrative (word) description of Robotic Arm Software Architecture

Describe in words how your Robotic Arm Software Architecture is designed. Please include details on the components and high-level structure (e.g. modules, functional hierarchy, …)

You may additionally describe your RASA solution using software system architecture diagram that shows inputs to the system, locations where computing and processing happens, and where software elements interact with each other.

Minimum content requirement: Description of your system architecture

1.2 Logical Flow Diagram

Describe the logical flow or algorithm for the RASA when it receives a high-level command from Astrobee. Preferred format is a flow diagram, like the below examples of flow charts.

You do not need to specify exact code, just generally what function, operators, or comparisons would be used and how. Be sure to include how each element identified in your description in 1.1 is being used, and how any available feedback (e.g., actuator position) is used in your solution. Your software description should be complete enough that an experienced programmer could implement the algorithm.

Your diagrams must show:

- What inputs are being used either from the Astrobee or from sensors described in the telemetry list described in Section 5 (Table 5) of the problem document
- What operations are being used to perform actions
- What logic is present to make choices and what logical operators are being used
- If variables are being used, where those variables are being stored, and how often they are read or written

Present your software's control flow description using an Activity/Control Flow Diagram, also known as a Flowchart. An Activity Flow Diagram use squares to represent processes, diamonds to represent true/false case structures, ovals to indicate start and stops of programs, and arrows to designate program flow and sequence. Your Flowchart must show every outcome and what steps are taken to get there. Below is an example of a very high level flowchart describing a motor controller in Figure 1.

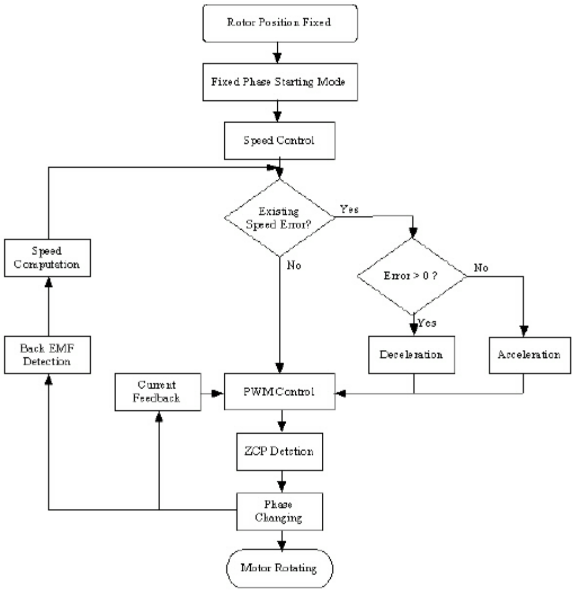

Figure 1 - Flow Chart Example

In the below example flowchart (Figure 2), you can see this visual language used:

- Rectangles (blue boxes in the example) to indicate processes
- Diamonds to indicate decisions being made
- Half cylinders (white shapes) to indicate where data is being stored and read from
- Curved Boxes (orange shapes) to indicate controls or messages being sent from the RASA to the actuator controllers or Astrobee.

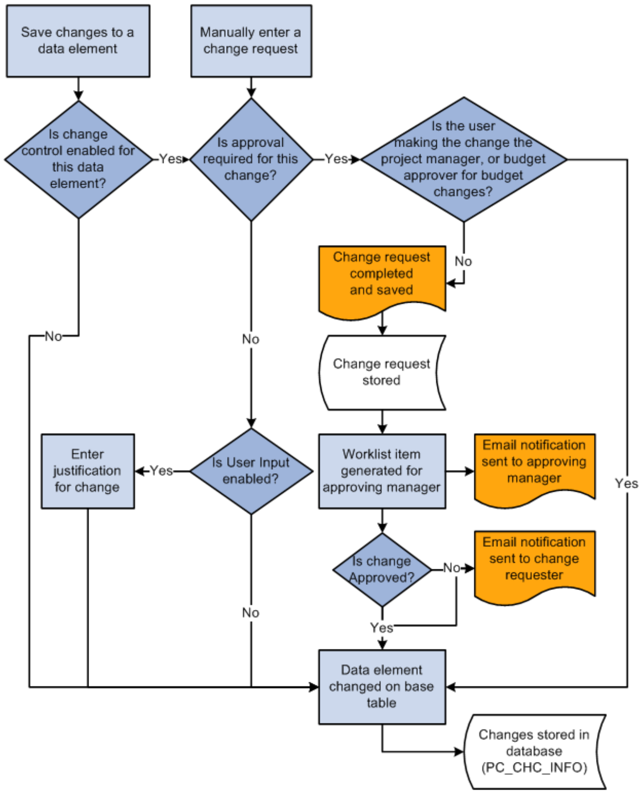

Figure 2 - Second Flow Chart Example

Minimum content requirement: Flowchart or Logical Flow Diagram that can handle all RASA inputs and reflects all required functionality.

1.3 Functional Analysis

1) How does your RASA determine when each of the Astrobee commands (Section 3, R1-R4) is complete?
2) Please describe the details of your RASA design's interface to Astrobee.
3) Is there any additional info about or changes to the electronics hardware suite (Section 4, Figure 1) that could improve the efficiency of your RASA design (1.1 above)?

Minimum content requirement: Text responding to each of the above questions.

2 Design Implementation

1) Describe the testing and/or simulation environment(s) required to validate/confirm your RASA solution works.

2) For all components of your RASA solution described above (in 1.1), describe how you would estimate the lines of code you'd expect to be necessary to implement the solution. Include an exact number for the total lines of code to implement your RASA solution.
   Please explain how you arrived at your estimate and be sure to include all assumptions you have made to generate this estimate. The credibility of your estimate depends mainly on the information you provide here.

Minimum content requirement: Text responding to each of the above questions.

3 Exit Survey

To complete your submission, please take the Exit Survey by going to this webpage:

https://seasgwu.qualtrics.com/jfe/form/SV_2r9DaeSlh48uMcZ

At the end of the survey you will receive a unique code. In your submission, include this section and the text:

Exit Survey for Freelancer <<insert Freelancer username>> complete per completion code: <<insert completion code>>.
