# Supplementary material for: Linking solver characteristics, solving processes and solution attributes: A data explainer for an open innovation generated robotic design dataset
Source: Data Brief. 2023 Sep 6;50:109547. doi: 10.1016/j.dib.2023.109547 (PMC10518673; doi:10.1016/j.dib.2023.109547)
Supplement: Supplementary file 1 [file mmc1.zip › Release/Process/Challenge Rules/D4-RASA/RASA Blurb.docx]

# Robotic Arm Software Architecture - (RASA)

In this challenge, you are asked to design the Robotic Arm Software Architecture (RASA) that will control a Robotic Arm (RA) that has been separately designed to attach the Astrobee Robotic Free Flyer to a Handrail within the International Space Station (ISS), and orient Astrobee in two perpendicular directions. The RASA will receive high-level commands from Astrobee and implement them through an electronics suite to control the RA.

Note that no executable code is required, but your proposed software architecture must be sufficiently descriptive to allow experts to assess its feasibility (i.e., comply with all the requirements) and follow the prescribed format.

***Challenge Rules:*** A prize of **$500** will be awarded for the **most efficient, technically feasible** design, **submitted before 21:00 GMT on November 15^th^ 2018.** Design efficiency will be evaluated based on the lowest, credible estimated lines of code for implementing a solution.

No working prototype is required for submission, but the design must be sufficiently detailed to allow experts to assess the feasibility of your design (i.e., comply with all requirements) and the credibility of your mass estimate. Only complete submission packages will be evaluated.

*Click on the links below to see detailed design instructions, constraints and solution templates for this problem.*

Attachments:

RASAProblemDescription.pdf

RASASubmissionGuidelines.pdf
