# Supplementary material for: Linking solver characteristics, solving processes and solution attributes: A data explainer for an open innovation generated robotic design dataset
Source: Data Brief. 2023 Sep 6;50:109547. doi: 10.1016/j.dib.2023.109547 (PMC10518673; doi:10.1016/j.dib.2023.109547)
Supplement: Supplementary file 1 [file mmc1.zip › Release/Process/Challenge Rules/D5-EDC/EDC Blurb.docx]

“Electrically Driven Clamp” (EDC)

In this challenge, you are asked to design an Electrically Driven Clamp (EDC) that will be mounted to, and driven by, a separately designed robotic arm. The EDC receives electrical power (i.e., an applied voltage) that it must convert into the desired actions. This contest is focused on the electro-mechanical system only (i.e., no internal computing or circuitry)

***How it works***: Initially, the EDC will be positioned at a Handrail, in a ready-to-clamp orientation.. When powered and controlled, the EDC must be capable of executing three high-level actions: 1) close on the Handrail, 2) maintain a hold on the handrail for an extended period of time, and 3) release from the handrail.

*Click on the links below to see detailed design instructions, constraints and solution templates for this problem.*

***Challenge rules***: A prize of **$250** will be awarded for the **lowest mass, technically feasible** solution submitted by **July 20^th^, 2018**. No working prototype is required for submission, but the design must be sufficiently detailed to allow experts to assess the feasibility of your design (i.e., comply with all requirements) and the credibility of your mass estimate. Only complete submission packages will be evaluated.

Attachments:

- EDCProblemDescription.pdf
- EDCSubmissionGuidelines.pdf

Templates

- EDCMassTemplate [.xlsx, .odt]
