# Supplementary material for: Linking solver characteristics, solving processes and solution attributes: A data explainer for an open innovation generated robotic design dataset
Source: Data Brief. 2023 Sep 6;50:109547. doi: 10.1016/j.dib.2023.109547 (PMC10518673; doi:10.1016/j.dib.2023.109547)
Supplement: Supplementary file 1 [file mmc1.zip › Release/Process/Challenge Rules/D5-EDC/EDC Problem Description.pdf]

1 Contest Description

In this contest you are asked to design an Electrically Driven Clamp (EDC) that will be mounted to the free end of a separately designed robotic arm. ***This challenge is focused on the electro-mechanical system only (i.e., no internal computing or circuitry).*** The EDC will be electrically powered by the robotic arm to close on an International Space Station (ISS) handrail ("Handrail"), maintaining a hold on it, and release the Handrail. The below specification details how the EDC will work, it's functional requirements and interface constraints/assumptions. A separate document provides detailed guidelines on how your design must be presented and submitted.

A prize of $250 will be awarded for the lowest mass, technically feasible solution, submitted before 21:00 GMT on August 6th, 2018.

2 Concept of Operations – How the EDC needs to work

2.1 Normal Operations

Electrical power will be provided to the EDC at its interface to the robotic arm through electrical connections. When provided power in a certain way that you specify later, the EDC must be able to perform three operations: 1) close, which involves attaching the EDC to the Handrail and maintaining a hold on it; 2) hold which involves maintaining the *attached* configuration while resisting externally applied loads and 3) release which involves releasing its hold on the Handrail. The relationships among the configurations and operations are illustrated in Figure 1. The requirements for each operation (underlined) and intermediate configuration (italicized) are detailed in section 3.

2.2 Contingency (Emergency) Operations

There are several scenarios when normal operations may be disrupted. The ranges of permissible responses are detailed in section 3.4. This section summarizes the scenarios: 1) when the EDC attempts to close on the Handrail, but there is no Handrail present; 2) when the EDC experiences higher than expected loads while attached (e.g., because an astronaut or other object bumps or smashes into Astrobee); and 3) when an astronaut manually removes the EDC from the Handrail.

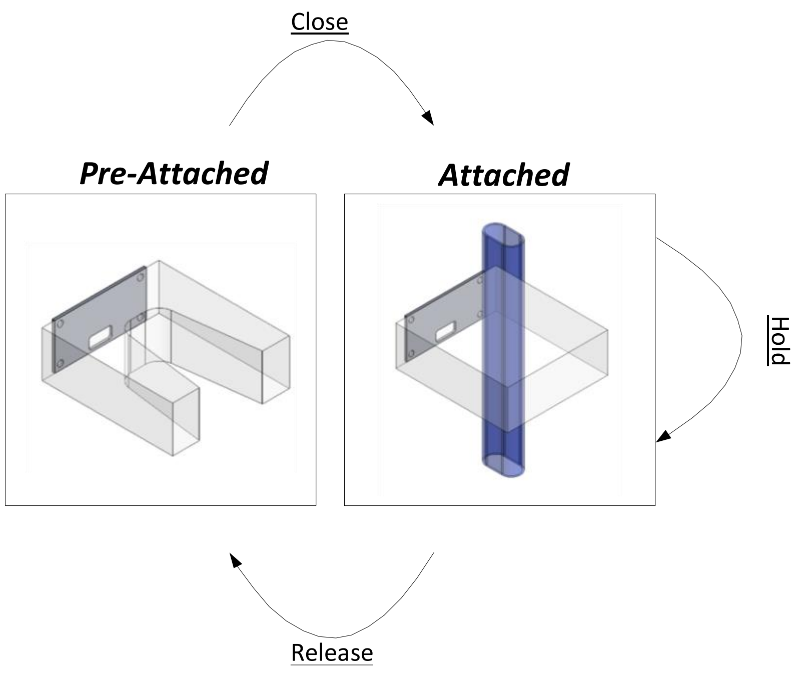

Figure 1 – Concept of Operations Illustration

3 Functional Requirements

This section details all of the functional requirements that the EDC must meet.

3.1 Motion Requirements

R1 Close: The EDC shall be able to move from the *pre-attached* configuration to the *attached* configuration.

R1.1 Pre-attached configuration: The pre-attached configuration volume is defined in Figure 2, as 114 mm x 102 mm x 40 mm, with a cutout to permit placement on the Handrail.

R1.2 Pre-attach offset: When closing is initiated, the EDC shall not be offset from the center of the Handrail by more than:

- x = 59 mm $\pm$ 7 mm
- y = 0mm $\pm$ 7 mm
- z = 0 mm $\pm$ 7 mm
- $\theta_x = 0 \pm 5$ degrees
- $\theta_y = 0 \pm 5$ degrees
- $\theta_z = 0 \pm 5$ degrees

The coordinate system for these offsets is shown graphically in Figure 3 . The EDC shall only ever be controlled to attach to a standard Handrail as defined C7.

R1.3 Attached configuration: The EDC shall be considered attached when it is fixed to the Handrail. Fixed is defined as being able to resist slipping or twisting when subjected to normal operating loads of up to 3.5 Nm about either the Y-axis or Z-axis (ref Figure 3).

R1.4 Attached configuration: When closing is complete, the EDC must attach to the ISS Handrail and remain within the attached configuration volume in Figure 4.

R2 <u>Hold:</u> The EDC shall be able to maintain a rigid attachment (defined in R1.3) for an extended period (per R6).

R3 <u>Release:</u> The EDC shall be able to release the Handrail and move from the *attached* configuration (R1.3) to the *pre-attach* configuration (R1.1).

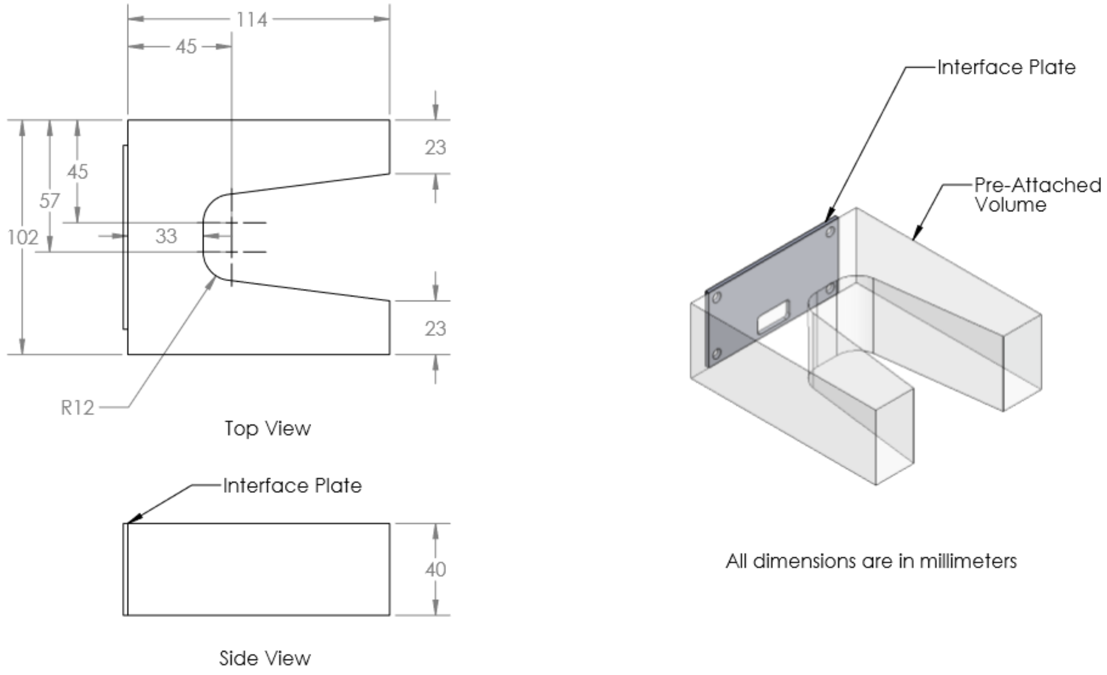

Figure 2 – Pre-attached Configuration

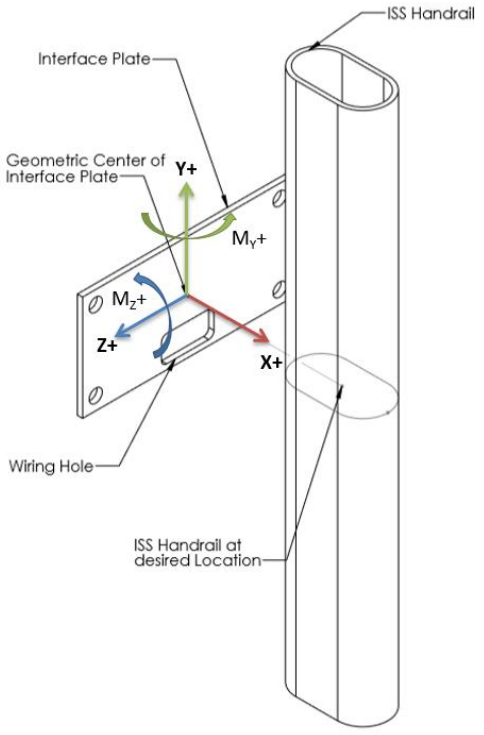

Figure 3 – EDC Frame of Reference.

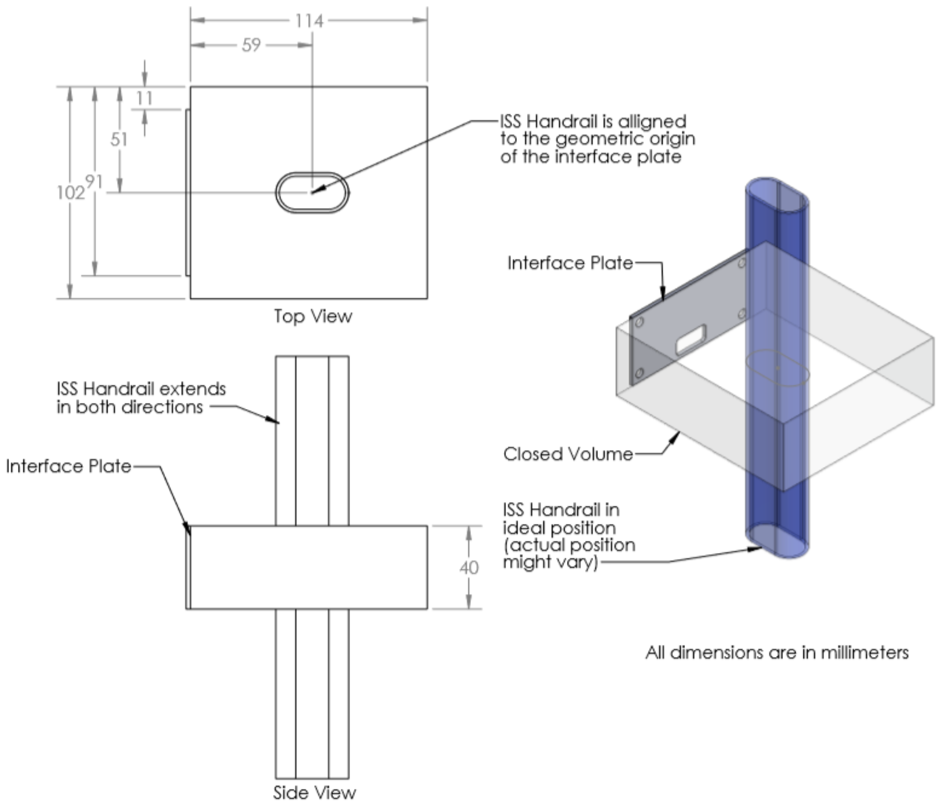

Figure 4 - Attached configuration

3.2 Sensor and Wiring Requirements

R4 The EDC design shall include enough sensors to provide the information that the robotic arm needs to control its operations. For each system configuration (i.e.- *attached* and *Attached*) your submission must define a) what that configuration means in the context of your design and b) what sensing or signal outputs you are defining to enable the robotic arm to determine when the EDC completes an operation and is in either configuration. Your design must also include the placement of the selected sensors, if they are used.

R4.1 The categories of permissible sensors and their mechanical properties are provided in Table 1. No other sensors may be used. Further information about the design, mounting, and sensing elements of the acceptable sensors is provided in the appendix.

R4.2 All sensors have wire bundles that need to be considered in your mechanical design and mass. In selecting sensors for your design, include mechanical accommodation of wires from the component to the interface plate. Wires should be fixed to structure to relieve strain if you expect the wires to be subjected to significant deflection or movement (e.g. pulling, twisting) during regular operations. In accommodating your sensor wire bundles, assume outside bundle diameter of 2.5 mm, with a 12.6 mm minimum bend radius, and a linear mass of 12.8 kg/km (26 AWG twisted shield pair bundles).

Table 1 - Permissible Sensor Options

Abstracted Sensor Decomposition	Volume	Mass of Sensor	Energy Cost	Information provided
No dedicated sensors - input power characteristics	You are free to use a non-sensing approach, for example, using the stall torque of your motor to determine when a mechanism has closed. There is no extra mass or volume for this, but be sure to clearly communicate how the design is intended to work in your submission.			
Continuous Rotation Sensor	Small knob with hole for sensing shaft (see Figure 9)	4 grams	0.09 Wh	Angle: Continuous and/or relative rotation of sensor shaft hole at 0.1 degrees resolution
Contact Sensor	Flat box with sensing face that depresses 1 mm (see Figure 10)	1 gram	0.02 Wh	Whether contact has been made: Discrete: On or Off
Linear Displacement Sensor	Flat plate with sliding sensing head that can slide 1" (see Figure 11)	10 grams	.375 Wh	Position: Continuous and Relative Displacement Information at 1 mm resolution.
Force Sensor	Flat Plate that determines force placed on the sensing face (see Figure 12)	1 gram	.375 Wh	Force: Continuous and absolute force from 0-100 N at 1 N resolution.

3.3 Resource Requirements

3.3.1 Timing Requirements

R5 Time to Close: The EDC shall be able to close within two minutes.

R6 Time in <u>Hold</u>: The MDC shall remain in an *attached* configuration on the Handrail for a maximum duration of 1 hr.

R7 Time to <u>Release</u>: The EDC shall be able to <u>release</u> within two minutes.

3.3.2 Power Requirements

You may assume that electric power is applied to all actuators and sensors as needed to drive them. The details of the interface and bus voltage are described in C2.3.

R8 Energy Budget: The MDC shall not use more than 4 Watt-hours to support all operations (R1-R3). Assume a maximum of 1 hr 26 minutes of passive operations during <u>Hold</u>

3.4 Safety Requirements

R9 The EDC shall have no sharp edges, defined as a radius of 3 mm, for astronaut safety.

R10 The EDC shall have no loops of material greater than 25.4 mm in diameter for astronaut safety, and no unsupported or unattached material more than 40 mm from the structure of the EDC.

R11 The EDC shall not damage itself through normal operations.

3.5 Environmental Requirements

R12 The EDC shall operate in the ISS zero gravity environment.

R13 The EDC, when unpowered, shall not be damaged by electrostatic discharge <4,000V.

R14 The MDC shall operate in an atmosphere comparable to that of Earth. Assume temperature of 21 °C , and pressure of 101 kPA , and relative humidity that is 40% - 70%.

R15 The EDC shall not contribute any particulates (e.g. dust) to the ISS atmosphere.

R16 The EDC shall enclose all lubricated components to prevent lubricants from leaking into the atmosphere of the ISS.

3.6 Contingency Requirements

R17 *No Handrail*: In some cases, the EDC may be controlled into attach configuration but there is no Handrail present. The EDC shall not damage itself while executing the operation.

R18 *Excessive loads cases*. This scenario may occur if an astronaut or piece of equipment contacts Astrobee while the EDC is attached (including while experiencing normal operating loads per R1.3). The EDC shall break away from the Handrail if it experiences a force of greater than 18N applied at the EDC interface in the negative Y-direction and a simultaneous moment of 6.7 Nm about the positive Z-axis as seen in Figure 5

R19 *Astronaut intervention*: The EDC shall be removable from the Handrail by an astronaut. Assume an astronaut can apply a pull-away force of 35.6 N in the negative X-direction as seen in Figure 5. This will not occur during any other operation.

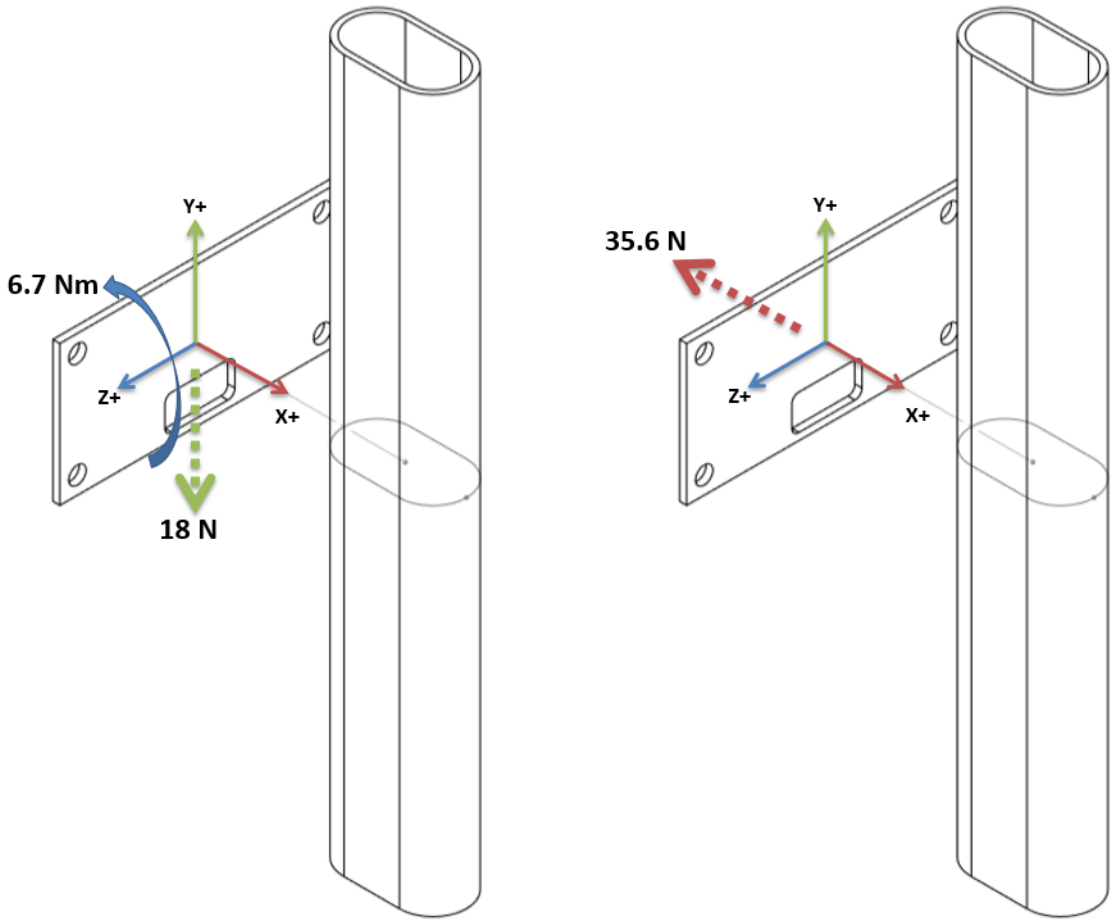

Figure 5 – Contingency loads: a) Excessive loads; b) Astronaut intervention loads

4 Interface Requirements

The EDC has a fixed interface to a separately designed robotic arm and a dynamic interaction with Handrails. The section describes all constraints imposed by those interfaces.

4.1 EDC-Robotic Arm Interface

4.1.1 Mechanical Interface

C1 Constraint 1 (C1) Mounting Interface: The EDC shall mount to the interface plate shown in Figure 6. There are four available screw holes in the specified locations seen in Figure 7.

C1.1 All external loads are applied at the interface plate.

C1.2 All wires must fit through the hole in the interface plate. Actuator wires are defined by the design. If you wish, you may use an assumption of a 6 gauge wire, at 5 mm in diameter , and a linear mass of 167 grams per meter for each actuator.

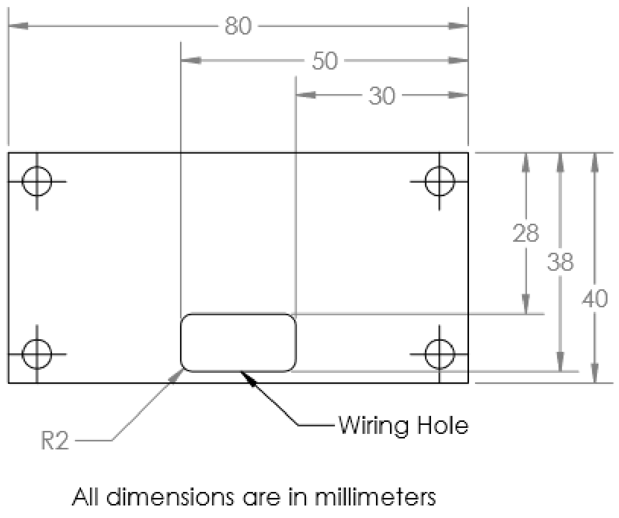

Figure 6 - EDC-Robotic Arm Interface Plate

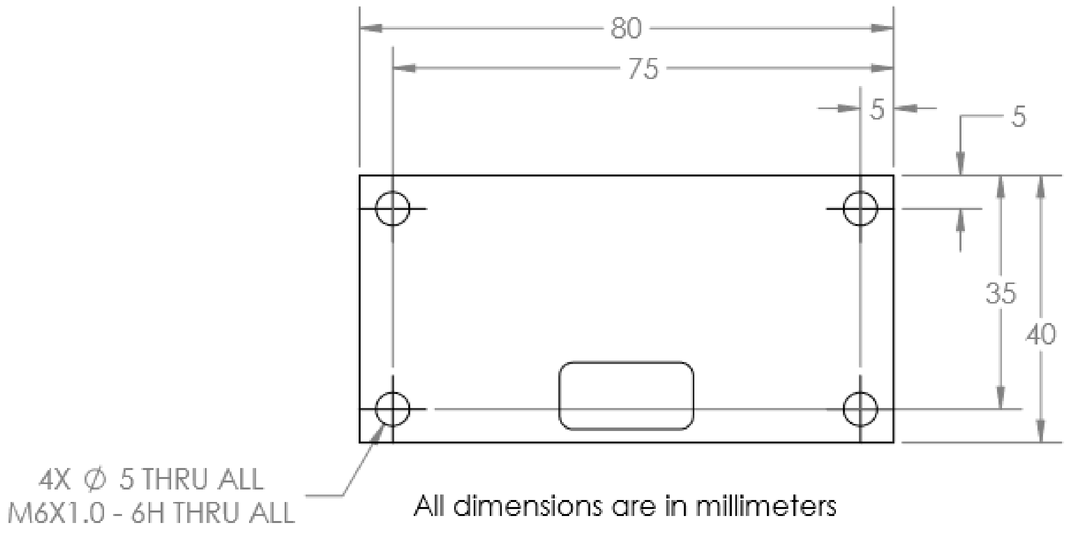

Figure 7 - EDC Robotic Arm Mounting Holes

4.1.2 Power Interface:

C2 Astrobee Bus Voltage+ is nominally 14.4 volts DC, but can vary from 11 to 17 volts DC.

C3 Max current: The EDC shall not draw more than 3 Amps.

C4 Steady State Current: The EDC shall not draw more than 2 Amps at steady state.

4.1.3 Control Interface:

C5 The robotic arm can provide any off-the-shelf actuator driver. If your design requires a non off-the-shelf driver, you must specify the driver. Here, actuators are defined as any device that moves a design element using electricity (e.g., solenoid, motor, piezo-motor, shape memory alloy or polymer etc.).

C6 All specified sensors (Table 1) can be read by the robotic arm.

4.2 Handrail Interface

C7 Handrail definition:

- C7.1 The shape of a standard Handrail is defined in Figure 8.
- C7.2 The Handrail is made of anodized aluminum. Assume the material is 6061 Aluminum of type T4 in terms of material properties and friction properties.
- C7.3 The Handrail is a 1.59mm thick aluminum 6061 extrusion.

C8 The Handrail shall not be damaged during operations through excessive force (per R2.3, R22 and R23). Damage includes, but is not limited to: crushing, denting, or bending.

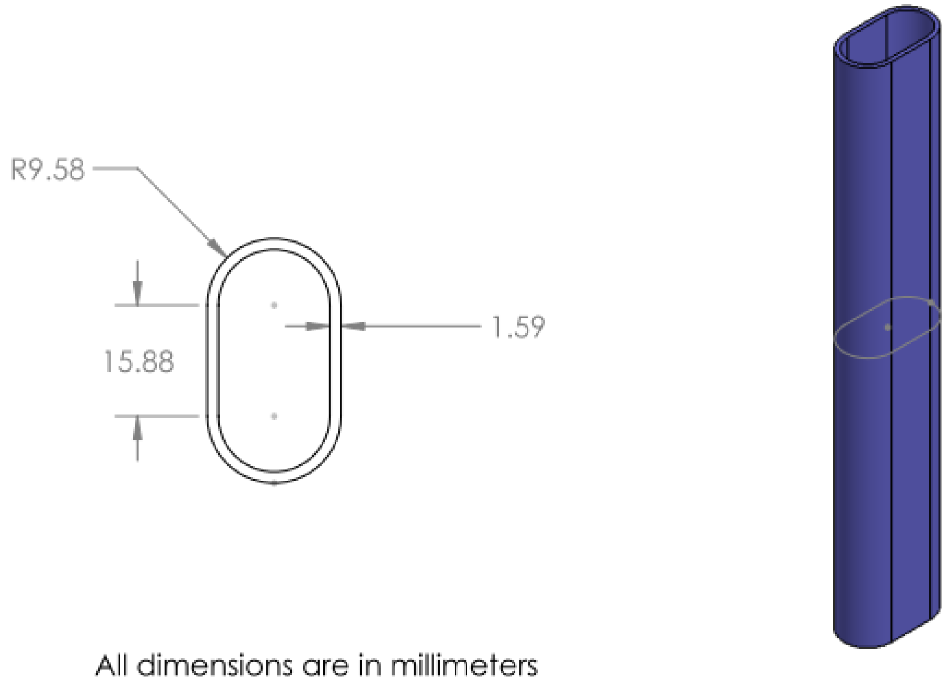

Figure 8 - ISS Handrail Definition

5 Sensor Appendix

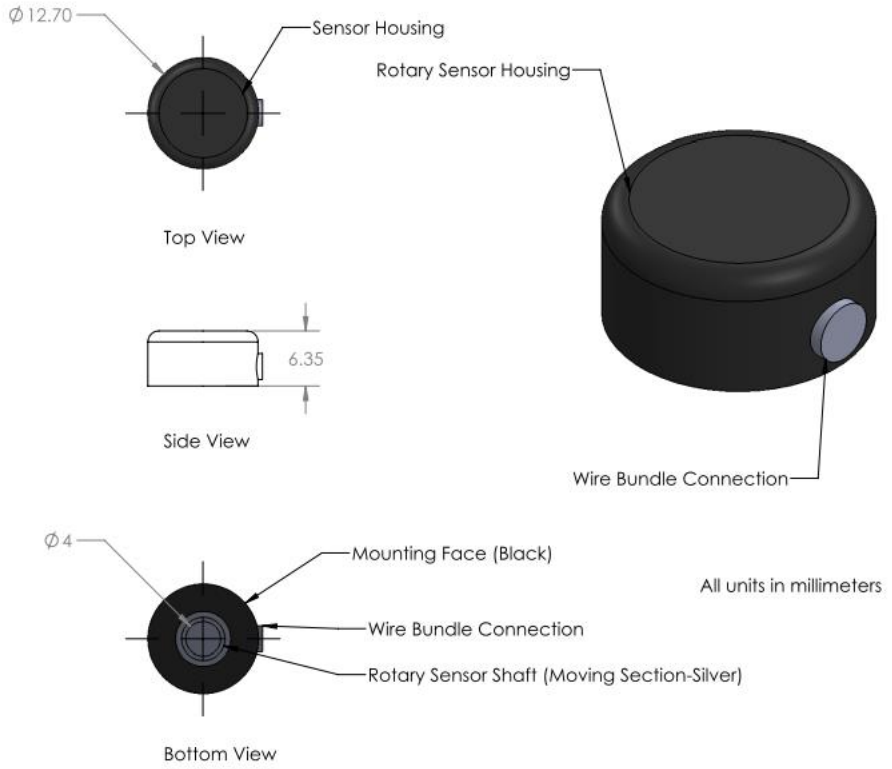

Figure 9: Continuous Rotation Sensor

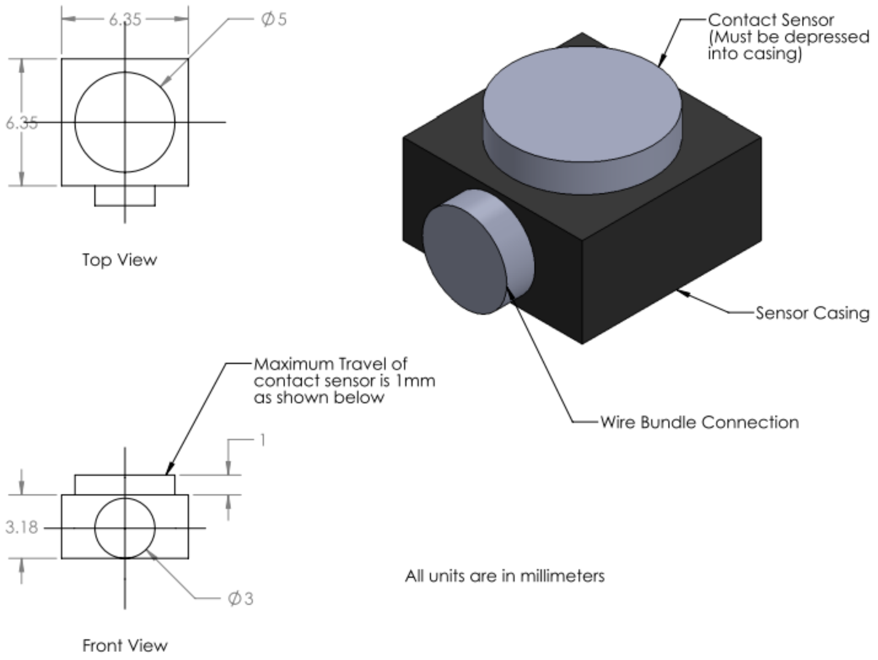

Figure 10: Contact Sensor

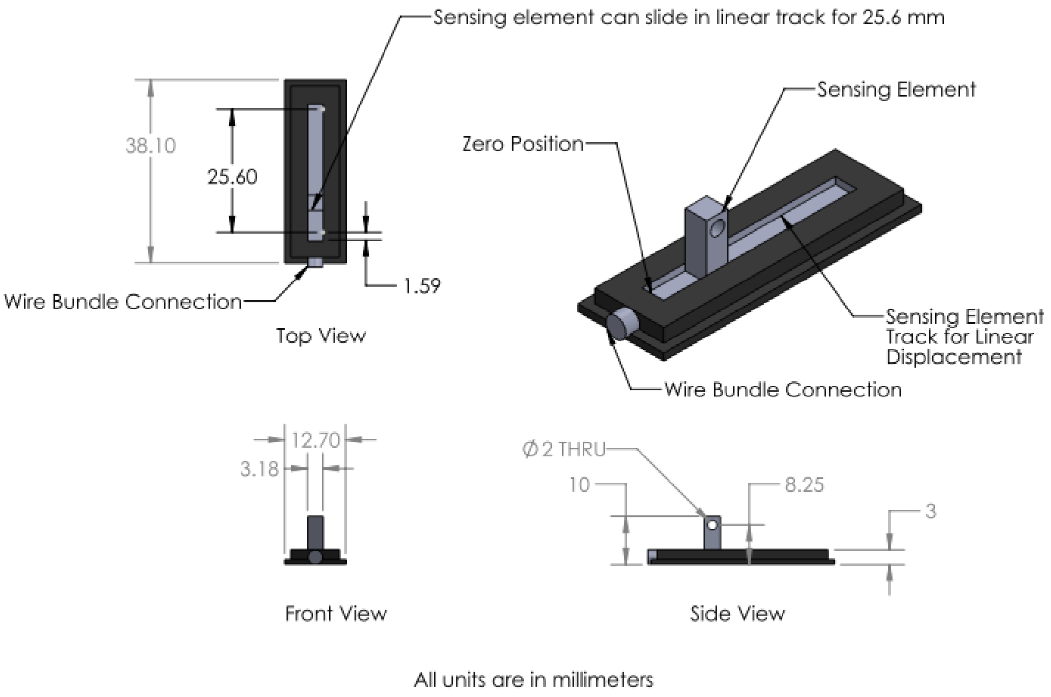

Figure 11: Linear Displacement Sensor

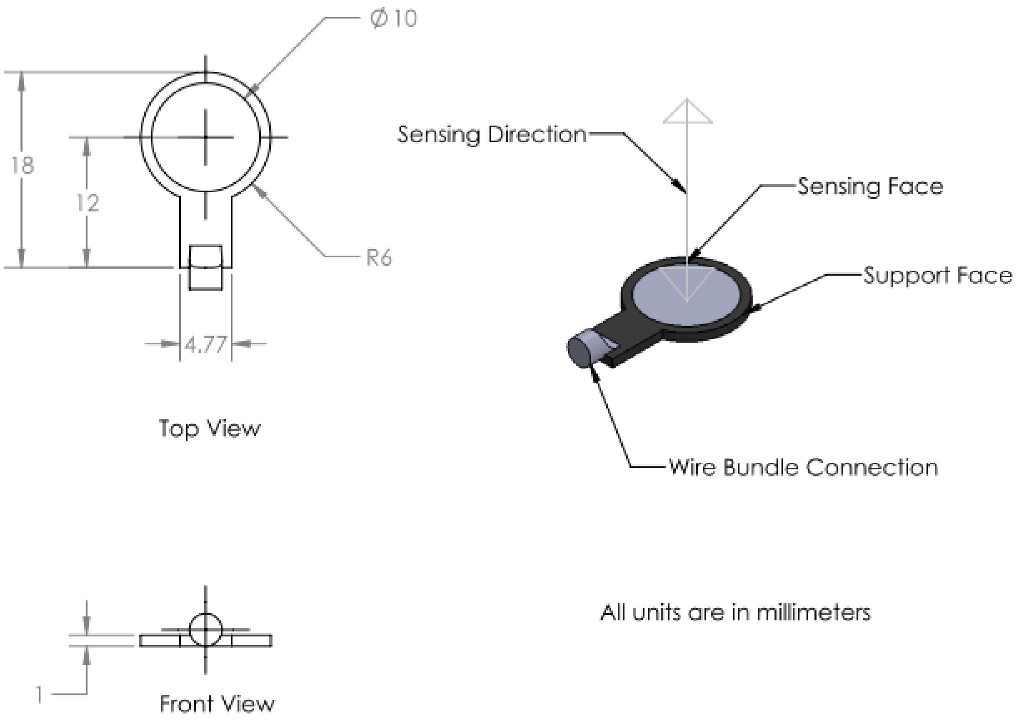

Figure 12: Stress Pressure Sensor
