# Supplementary material for: Linking solver characteristics, solving processes and solution attributes: A data explainer for an open innovation generated robotic design dataset
Source: Data Brief. 2023 Sep 6;50:109547. doi: 10.1016/j.dib.2023.109547 (PMC10518673; doi:10.1016/j.dib.2023.109547)
Supplement: Supplementary file 1 [file mmc1.zip › Release/Process/Challenge Rules/D4-CDPD/CDPD Problem Description.pdf]

1 Contest Description

In this challenge, you are asked to design a Command, Data and Power Distribution System (CDPD) that will control and power the elements of a separately-designed robotic arm. This robotic arm has been designed to grab a handrail on the International Space Station and move a camera on the "Astrobee" Free Flying robot in two directions. The CDPD receives all power and high-level commands through its electrical interface to Astrobee, and uses them to accomplish its primary functions described below.

The CDPD's primary job is to process electrical signals to and from Astrobee, read sensors in the robotic arm, and drive motors as directed by the robotic arm's high-level motion planning software. For this contest, you will be given a Reference Robotic Arm (RRA) design, with specific sensors and motors to read and control. The RRA design also includes the high-level control software that would run on your CDPD hardware, converting high-level commands from Astrobee into low-level, motor commands. Your CDPD then turns the low-level motor commands into appropriate power profiles to drive the RRA motors. Your CDPD must also be capable of converting raw signals from the sensors and motors into usable data streams for the RRA high-level control software.

A prize will be awarded for the most **efficient**, **technically feasible** solution. For this contest, efficiency is defined as the **minimizing mass and power.**

2 Concept of Operations – How the CDPD needs to work

2.1 Normal Operations

The CDPD should provide power and control signals to perform 4 high-level operations. When Astrobee is near a Handrail, four high-level commands are sent to CDPD to attach to the Handrail, point Astrobee in two directions, as commanded by human operators, release the Handrail and then return to a stowed configuration to await another Astrobee command. CDPD needs to execute motions originating from Astrobee commands by:

1. Converting electrical signals from Astrobee into high-level commands for RRA, and then passing these high-level commands to the RRA control software module.
2. Receiving low-level motor commands from the RRA control module, and driving appropriate RRA motors
3. Reading RRA sensors and providing telemetry to RRA error handling software module.
4. Passing motion confirmation messages from RRA control module to Astrobee.

Figures 1 & 2 illustrate how CDPD is expected to interact with the existing RRA mechanical components and high-level motion planning software. After these

activities have been completed the RASA may be commanded to repeat this process at some point in the future.

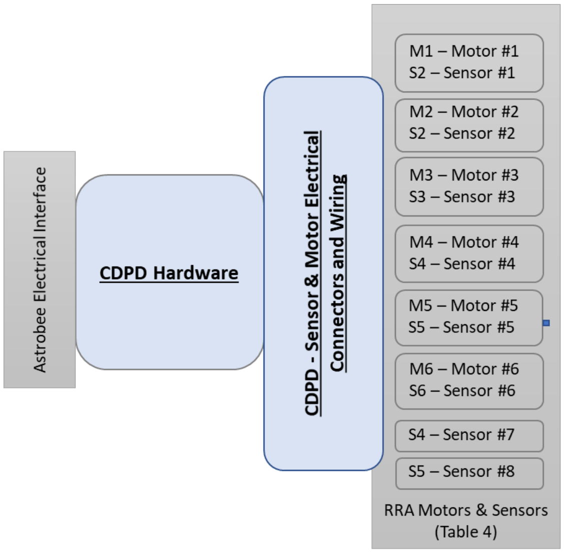

Figure 1 - CDPD Hardware connections

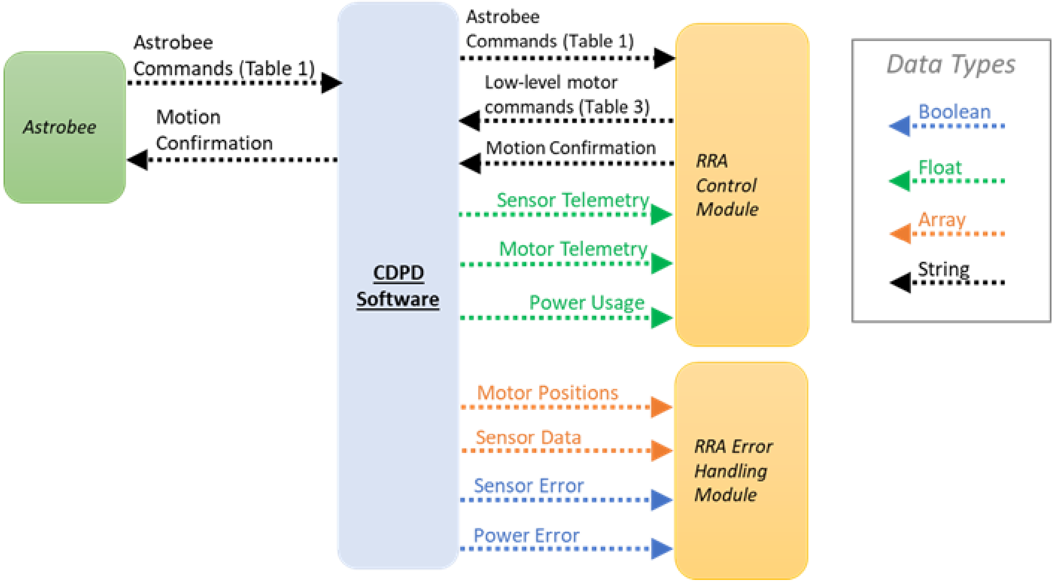

Figure 2 - CDPD Software interfaces

3 Functional Requirements

This section details the functional requirements that the CDPD must meet. The CDPD must be able to (1) interact with and accommodate existing RRA software that manages motion planning; (2) accommodate and read eight sensors; (3) drive six motors and sense motor current; (4) communicate with Astrobee, RRA sensors and RRA motors.

3.1 Motion Requirements

R1 Motion Requests from Astrobee: CDPD shall be able to receive any of 4 high-level motion commands from Astrobee and send them to the RRA software. The details of these commands are described in detail in Section 4.1 (C8).

R2 Motor Commands from RRA: CDPD shall be able to receive low-level motor commands from RRA Control Software. Specific motor control commands from the RRA Control Software are summarized in columns 4 &5 of Table 1 below.

R3 Motor control: CDPD shall be able to drive a specified RRA motor when sent a low-level command from the RRA Control Software.

R4 Type of motor: CDPD shall be able to drive the 6 motors in the RRA. An example of the kind of motor CDPD will be required to control is included in Section 4 (C9). Please use this motor as your basis for design.

R5 Number of active motors: CDPD shall be able to drive at least 3 motors simultaneously.

3.2 Motor Control Requirements

The format of all data and electrical interfaces to Astrobee and the RRA motors and sensors are specified in Section 4 below. This section describes how CDPD should be able to control the RRA motors and sensors.

R6 Motor control modes: CDPD shall be able to move a motor in four ways –, as described in Table 1 below.

R7 Position accuracy: CDPD shall control motor position and speed to levels described in Table 2, relative to the commanded position or speed.

Table 1 – Motor modes and commands

Motor Commands	Description	Inputs	Command format	Example for actuator, M1
Open loop velocity mode	Drives actuator at specified velocity	Direction (CW, CCW)*; Speed (deg/s);	MX_olv_(direction, speed)	M1_olv_(CCW, 1.3)
Timed velocity mode	Drives actuator at specified velocity for a specified time	Direction (CW, CCW)*; Speed (deg/s); Total time (s);	MX_tv_(direction, speed, total time)	M1_tv_(CCW, 1.3, 65)
Position mode	Drives actuator to a specified position at a specified velocity	Position (deg); Velocity (deg/s);	MX_pos_(position, velocity)	M1_pos_(42, 0.7)
Stop	Stops motor	none	MX_stop	M1_stop

*CW= Clockwise; CCW = Counter Clockwise

Table 2 - Motor position and speed accuracy

Motor	Relative Position Uncertainty	Required Speed at Motor Output	Speed Uncertainty
M1	±500 deg	0 – 400 rpm	±60 deg/s
M2	±500 deg	0 – 400 rpm	±60 deg/s
M3	±500 deg	0 – 400 rpm	±60deg/s
M4	±500 deg	0 – 600 rpm	±30 deg/s
M5	±500 deg	0 – 600 rpm	±30 deg/s
M6	N/A*	M6 minimum	N/A*

* M6 is expected to be driven in open loop velocity mode, at the low end of its speed range.

3.3 Sensing

R8 CDPD shall be able to sense motor current for each motor.

R9 CDPD shall be able to accommodate 2 dedicated sensors, in addition to a position sensor for each of the 6 motors. See Section 4.2 (C9) for examples of each kind of sensor.

R10 CDPD shall be able to produce streams of all motor and sensor data to RRA Motion Software at the given rates seen in Table 3.

Table 3 – Motor and Sensor Data

Data Stream Name	Description	Units/Format	Rate (Hz)
A1_pos	Actuator #1 output position	degrees	100
A1_spd	Actuator #1, speed	deg/s	100
A1_dir	Actuator #1, direction	CW or CCW	100
A1_power	Actuator #1, power	W	50
S7_force	Force Sensor output	N	200
S8_contact	Contact Switch position	On or Off	200

3.4 Resource Requirements

3.4.1 Power Requirements

All power and data are transmitted through the connector described in interface constraints described in Section 4.1.

R11 Steady State Current: Dedicated CDPD hardware shall not draw more than 0.3 A at steady state. All RRA motors and sensors are designed to operate successfully with less than this, so only worry about current drawn from your CDPD hardware.

3.5 Safety Requirements

R12 The CDPD shall have no sharp edges, defined as a radius of 3 mm, for astronaut safety.

R13 The CDPD shall have no loops of material greater than 25.4 mm in diameter for astronaut safety and unsupported or unattached for more than 40 mm from the structure of the CDPD.

R14 The CDPD shall not damage itself through normal operations.

R15 The CDPD shall be able to return to its normal operations if power is momentarily lost.

3.6 Environmental Requirements

R16 The CDPD shall operate in the ISS zero gravity environment.

R17 The CDPD, when unpowered, shall not be damaged by electrostatic discharge <4,000V.

R18 The CDPD shall operate in an atmosphere comparable to that of Earth. Assume temperature of 21 °C [70 °F], and pressure of 101 kPa [1 atm], and relative humidity that is 40% - 70%.

R19 The CDPD shall not contribute any particulates (e.g. dust) to the ISS atmosphere.

R20 The CDPD shall enclose all lubricated components to prevent lubricants from leaking into the atmosphere of the ISS.

4 Interface Requirements

The CDPD has electrical, mechanical and data interfaces to Astrobee. The section describes constraints imposed by those interfaces.

4.1 CDPD-Astrobee Interface

4.1.1 Mechanical Interface

C1 Constraint 1 (C1) Volume Constraint: Two volumes are available for your CDPD design. All of your design must fit within these two volumes except for hardware connecting components within these volumes to the RRA Motors and Sensors (C9). However, you should include all hardware mass in your mass estimate as outlined by the submission guidelines.

- C1.1 The primary volume (Volume #1) is fixed within the payload bay and contains the electrical interface to Astrobee.
- C1.2 An additional, smaller volume (Volume #2) sized 30mm x 30 mm x 30mm is available for your CDPD design. Volume #2 is closer to the motors and sensors, as described in C9 and Table 5.

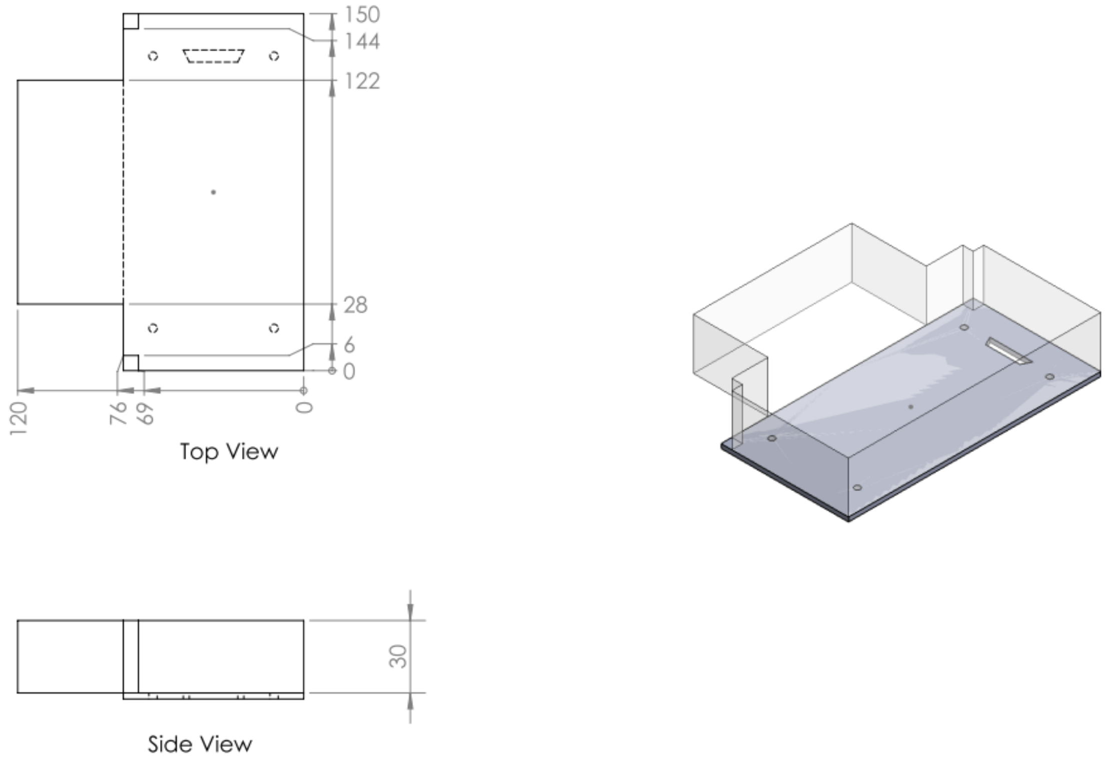

Figure 3 –Available Volume #1 (all dimensions in mm)

C2 Astrobee Interface Plate: The CDPD shall mechanically mount to a flat metal plate show in Figure 5. There are four available threaded holes (#8-32) for your mounting.

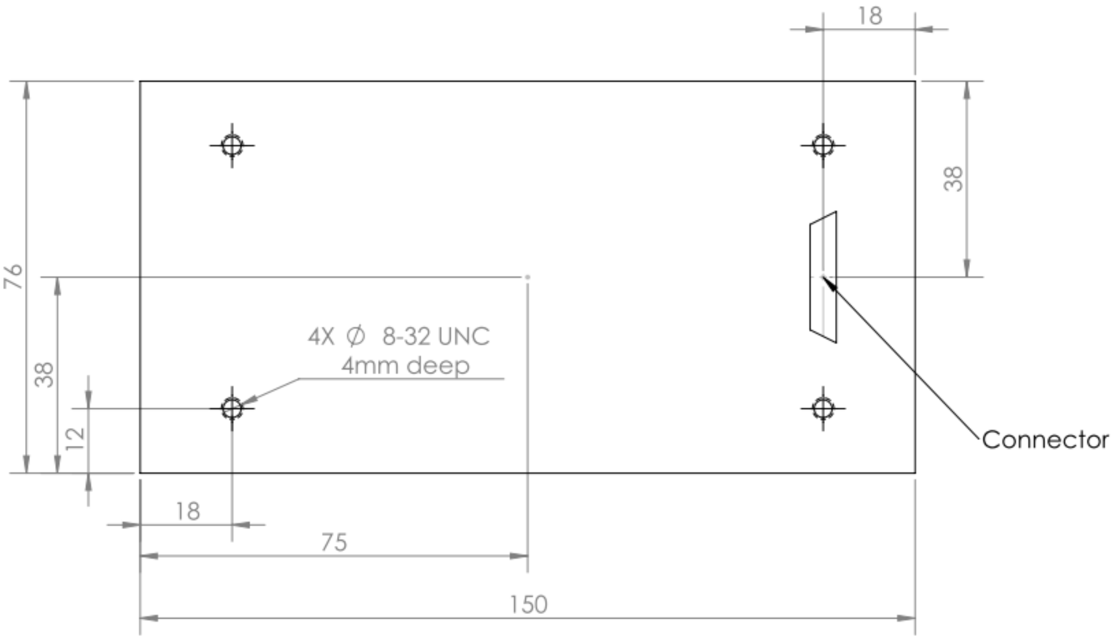

Figure 4 - Astrobee Interface Plate (all dimensions in mm)

4.1.2 Electrical Interface:

C3 The CDPD shall connect to Astrobee through a defined power and data connector - a 31-pin payload Glenair M83513/03-E03N. The location of this connector is described above in Figure 4

C4 Supply Voltage: Available electrical power from Astrobee is normally at 14.4 V DC, but can vary between 11 to 17 V DC.

C5 Pinout is as follows: Pin 4 is bus voltage. Pin 1 is ground.

4.1.3 Data Interface:

C6 All commands will be received and transmitted as part of a serial command using the second and third pins of the 31-pin connector. The second pin is the positive serial command line, and the third pin is the negative serial command line.

C7 CDPD shall be able to receive and transmit commands formatted in ASCII using the RS-232 protocol once.

C8 The serial messages from Astrobee that CDPD needs to process are specified in Table 4, along with actions associated with each of these commands.

Table 4 – Astrobee Communication

Command format	Action
Motion Requests sent to CDPD by Astrobee	
"attach(x,y,z)"	Pass to RRA Control Software
"pan(θ_x)"	Pass to RRA Control Software
"tilt(θ_y)"	Pass to RRA Control Software
"stow"	Pass to RRA Control Software
Motion Confirmations sent by CDPD to Astrobee	
"attachconfirmed"	Send when confirmation comes from RRA Control Software
"panconfirmed"	Send when confirmation comes from RRA Control Software
"tiltconfirmed"	Send when confirmation comes from RRA Control Software
"stowconfirmed"	Send when confirmation comes from RRA Control Software

4.2 CDPD-RRA Interface

C9 RRA Motors and Sensors: CDPD shall accommodate the motors and sensors like those identified in Table 5.

Table 5: RRA Motor and Sensor List, with distances from available CDPD Volumes

ID	Description	Example component specification	Distance from Volume #1	Distance from Volume #2
M1<br>M2<br>M3<br>M4<br>M5<br>M6	Brushless rotary motor for each Motor	Maxon EC 32 flat motor, part # 339271;<br>Catalog page:<br>https://www.maxonmotor.com/medias/sys_master/root/8830904565790/2018EN-262.pdf	150 mm	75 mm
S1<br>S2<br>S3<br>S4<br>S5<br>S6	Rotary encoder	CUI Inc incremental encoder, part # MES3-100P:<br>https://www.cui.com/product/resource/mes3-series.pdf	150 mm	75 mm
S7	Contact Switch	Honeywell microswitch, 311SM6-T:<br>https://sensing.honeywell.com/honeywell-sensing-micro-switch-sm-basic-switch-product-sheet-004959-3-en.pdf	200 mm	100 mm
S8	Piezoresistive force sensor	Tekscan force sensor:<br>https://www.tekscan.com/products-solutions/force-sensors/a101	200 mm	100 mm

C10 RRA Control Software: Your CDPD hardware design must be capable of running a real time operating system (RTOS) in order to support the RRA high-level software that utilizes a primary control loop with input data at a maximum of 200Hz.

C11 The inertia of the load being driven by each RRA motors is bounded by a load-to-motor inertia ratio between 4:1 and 6:1.
