# Supplementary material for: Linking solver characteristics, solving processes and solution attributes: A data explainer for an open innovation generated robotic design dataset
Source: Data Brief. 2023 Sep 6;50:109547. doi: 10.1016/j.dib.2023.109547 (PMC10518673; doi:10.1016/j.dib.2023.109547)
Supplement: Supplementary file 1 [file mmc1.zip › Release/Process/Challenge Rules/D4-CDPD/CDPD Blurb.docx]

# Command, Data, and Power Distribution System – (CDPD)

In this challenge, you are asked to design a Command, Data, and Power Distribution System (CDPD) that will control and power the elements of a separately designed robotic arm. This robotic arm has been designed to grab a handrail on the International Space Station, and move a camera on the “Astrobee” Free Flying robot in two directions. The CDPD receives all power and high-level commands through its electrical interface to Astrobee, and uses them to accomplish its primary functions.

***How it works:*** The CDPD’s primary job is to process electrical signals to and from Astrobee, read sensors in the robotic arm, and drive motors as directed by the robotic arm's high-level motion planning software. For this contest, you will be given a Reference Robotic Arm (RRA) design, with specific sensors and motors to read and control. The RRA design also includes the high-level control software that would run on your CDPD hardware, converting high-level commands from Astrobee into low-level, motor commands. Your CDPD then turns the low-level motor commands into appropriate power profiles to drive the RRA motors. Your CDPD must also be capable of converting raw signals from the sensors and motors into usable data streams for the RRA high-level control software.

***Challenge rules***: A prize of **$1,500** will be awarded for the most **efficient**, **technically feasible** solution that is submitted by **March 8^th^, 2019**. For this contest, efficiency is defined as the **minimizing mass and power**. No working prototype or simulation is required for submission, but the design must be sufficiently detailed to allow experts to assess the feasibility of your design (i.e., comply with all requirements) and the credibility of your mass and power estimates. Only complete submission packages will be evaluated.

**Attachments:**

CDPD_ProblemDescription.pdf

CDPD_SubmissionGuidelines.pdf

Templates:

CDPDMassTemplate [.xlsx .ods]

CDPDPowerTemplate [.xlsx .ods]
