# Supplementary material for: Linking solver characteristics, solving processes and solution attributes: A data explainer for an open innovation generated robotic design dataset
Source: Data Brief. 2023 Sep 6;50:109547. doi: 10.1016/j.dib.2023.109547 (PMC10518673; doi:10.1016/j.dib.2023.109547)
Supplement: Supplementary file 1 [file mmc1.zip › Release/Process/Challenge Rules/D4-CDPD/CDPD Submission Guidelines.pdf]

Submission Guidelines for the Command, Data, and Power Distribution System

In this contest, you were asked to design a Command, Data, and Power Distribution System that will control and power the elements of a separately-designed robotic arm that has been designed to grab a handrail on the ISS and move a camera on the "Astrobee" Free Flying robot in two directions.

This document provides detailed guidelines on how you must describe and present each aspect of your design to be considered for the prize. This document looks long but very little text is required. Your submission document must include each of the sections detailed below and all of the information requested in each. Several templates and examples are provided to clarify what constitutes a complete solution.

Use the exact section and subsection header words, shown below.

1 FUNCTIONAL DESCRIPTION 2

1.1 NARRATIVE (WORD) DESCRIPTION OF DESIGN 2

1.2 FUNCTIONAL ANALYSIS 2

2 MASS SUMMARY AND COMPONENT LIST 3

3 SYSTEM LAYOUT 4

4 DESIGN DRAWINGS 6

5 SOFTWARE DESCRIPTION 7

6 POWER USAGE DESCRIPTION 8

7 EXIT SURVEY 9

1 Functional Description

1.1 Narrative (word) description of design

In this section, describe how your CDPD design works. In a few sentences, please describe how your solution does each of the following:

1) Driving Motors: How does your CDPD drive the specified motors after being sent a command from Astrobee? Where does the interpretation of the command occur? Which parts of your CDPD design are involved with driving motors? What telemetry (sensor) data is used to control motors?
2) Sensor Accommodation: Where and how are the sensor signals transformed into something a computer could read?
3) Describe how the RRA motors and sensors are physically connected to your CDPD hardware (e.g. connector types, number/length of wire bundles, wire size/insulation, …).
4) How does your design deal with sensor signal quality, noise or other electrical interference?
5) How did you select computing elements (the components that interpret and react to input signals) for your design?
6) How could programming be loaded on to your CDPD?
7) What programming language(s) has your system been designed to use?

Although it is not required, you may embed images of sketches, models, storyboards or other illustrations in your written descriptions to help describe your CDPD design.

Minimum content requirement: Text response to each of the above questions.

1.2 Functional Analysis

In this section, describe your logic and/or analysis for the following aspects of your CDPD design. Including equations and mathematics is acceptable if it helps clarify the logic behind your design, but please ensure that it will be understood by our reviewers by annotating your process or describing the math being done and why.

1) What data is stored for low-level motor control, and how is it stored? How much storage does your design have in total?
2) What is the minimum controllable step for moving Motor #6?
3) Assuming three motors operating simultaneously, requiring a total of 1.5A, how long could your CDPD components operate without getting too hot?
4) If the motor inertia ratio were twice as high for Motor #1, explain how your design would perform.

Minimum content requirement: Text responding to each of the above questions. Some responses must include a numerical value.

2 Mass Summary and Component List

In this section, list all the elements of your CDPD solution using the template provided [CDPDMassTemplate.xlsx, .odt]. For each component/piece/part, include an estimate of its mass and a brief explanation of where the estimate came from. Please be sure to include the reasons supporting your mass estimate for each element since they will be part of the evaluation of the credibility of your CDPD mass estimate.

Table 1 provides an example of how the template should be filled in.

		Is this a powered component?	Estimated Mass per Unit (kg)	Quantity (# units)	Mass (kg)	Basis of Estimate
**1.0**	**Subsystem #1**				**1.88**	
	Electronics Box A	Yes	0.200	1	0.20	Weighed a prototype I built
	Switch #1 & 2	Yes	0.030	2	0.06	Called some former coworker who builds these, and asked for a typical masses
	Mechanism #1	No	0.800	1	0.80	Made a CAD model, assumed SS316, to obtain this mass
	Attachment hardware	No	0.040	8	0.32	Typical mass of component that I use all the time in design of systemX.

Table 1 - Mass Summary and Components List example

Minimum content requirement: Paste your filled table into this section of the document. No additional text is required.

3 System Layout

In this section, provide a diagram(s) identifying all the physical components/pieces/parts of your CDPD design and how components physically connect, power, or exchange information with each other.

Please use the names of components from the Mass Summary and Component List described in section 2. You may use a combination of different diagrams, but please be sure to include each electrical and mechanical component of your design in at least one diagram.

If you represent mechanical and electrical together, the system layout may be presented as a block diagram. A Block Diagram shows how each of the components connect to one another. Each component is represented as a box and the lines that connect the blocks identify what is being transferred/passed or supported between the blocks. Be sure to identify how your design connects to the RRA. Figure 1 provides an example of a block diagram.

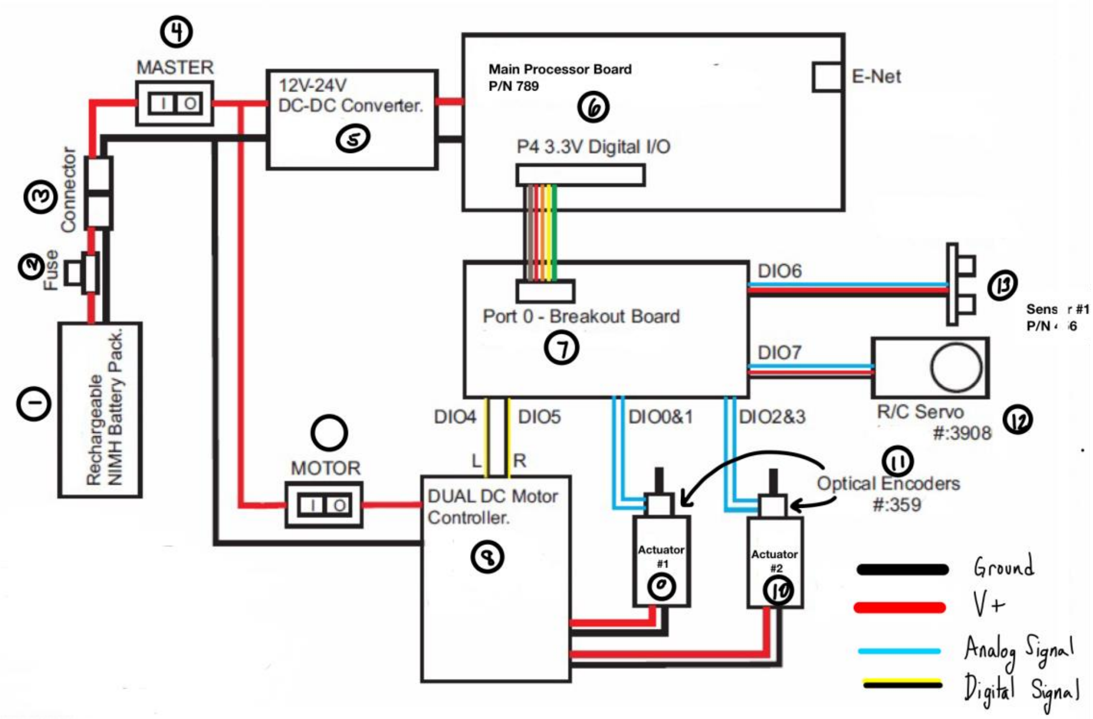

Figure 1 - Block Diagram - example

If you include your mechanical layout separately, you could also use a schematic similar to a "Wiring Diagram." See Figure 3 for an example. This schematic must identify all the direct[1] electrical connections among all the electrical (e.g. sensors, amplifiers, receivers, ICs), electro-mechanical (motors) components of your solution, as well as wiring and connectors between components. Make sure to identify any connection between your electrical system and the connector in the interface plate.

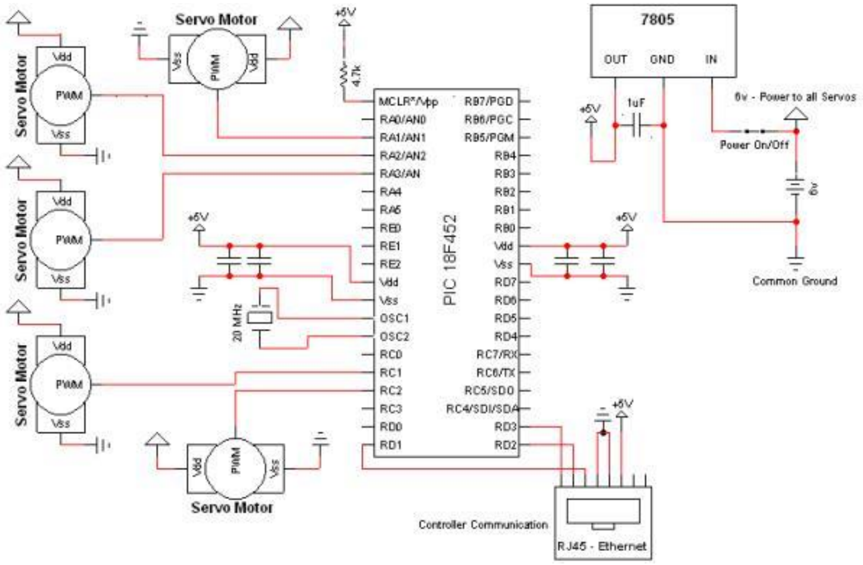

Figure 2 - Wiring Diagram – example

Minimum content requirement: Either a a) block diagram showing all elements in the Mass and Component List, or b) both a mechanical and electrical description

---

[1] Only detail the direct electrical connections. For example, if you have a gearbox connected to an electrical motor which has electrical leads coming off of it, this diagram would only show the electric motor and the electrical leads as well as those lead's connection to the larger system. The diagram would not need to show the gearbox, as it is separated from the electrical system by the electric motor.

4 Design Drawings

In this section, include drawings showing your solution's physical configuration to show us that your design fits within the available volumes, and could be mounted to the Astrobee interface plate.

You could use a schematic similar to an "exploded view" of your design. See Figure 2 for an example. The defining feature of an exploded view is that it conveys information about how all the mechanical pieces connect to one another. If you do provide and exploded view of the mechanical elements of your design, please also include a view of the full system in its assembled configuration as well.

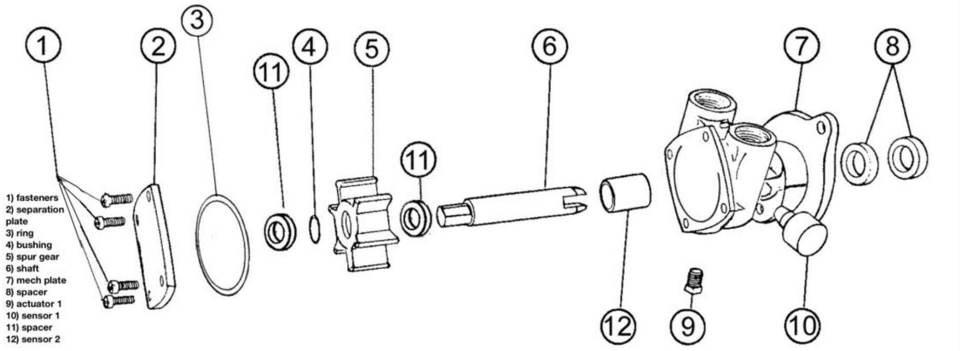

Figure 3 - Mechanical (exploded view) schematic - example

You can use any CAD software you like, sketch them by hand or photograph a prototype, but accurate dimensions of the whole system are required (we'd much rather have proof that your design can fit in the available volume than the specific location of each bolt). For each view, please provide at least one off-angle view to show perspective. Please label as many elements and subassemblies as possible. Use the names specified in your Mass Summary and Component List (section 2).

Minimum content requirement: Design drawing of your CDPD. Figures must be clearly labeled and dimensioned.

5 Software Description

In this section, describe at a high-level your CDPD software design. Include descriptions of how your design uses software to accomplish all necessary tasks, such as motor control, data handling and communication. You do not need to provide executable code, but please include a sufficiently detailed description of how your CDPD software elements would work. Be sure to include how each element or subassembly identified in the Component List is being controlled, and how your software uses any feedback from (e.g., sensors). Your software description should be complete enough that an experienced programmer could implement the algorithm.

Present your software's control flow description using an Activity/Control Flow Diagram, also known as a Flowchart. An Activity Flow Diagram use squares to represent processes, diamonds to represent true/false case structures, ovals to indicate start and stops of programs, and arrows to designate program flow and sequence. Your Activity Flow Diagram must show every process and outcome, and a description of the steps taken to get there. See Figure 4 for an example.

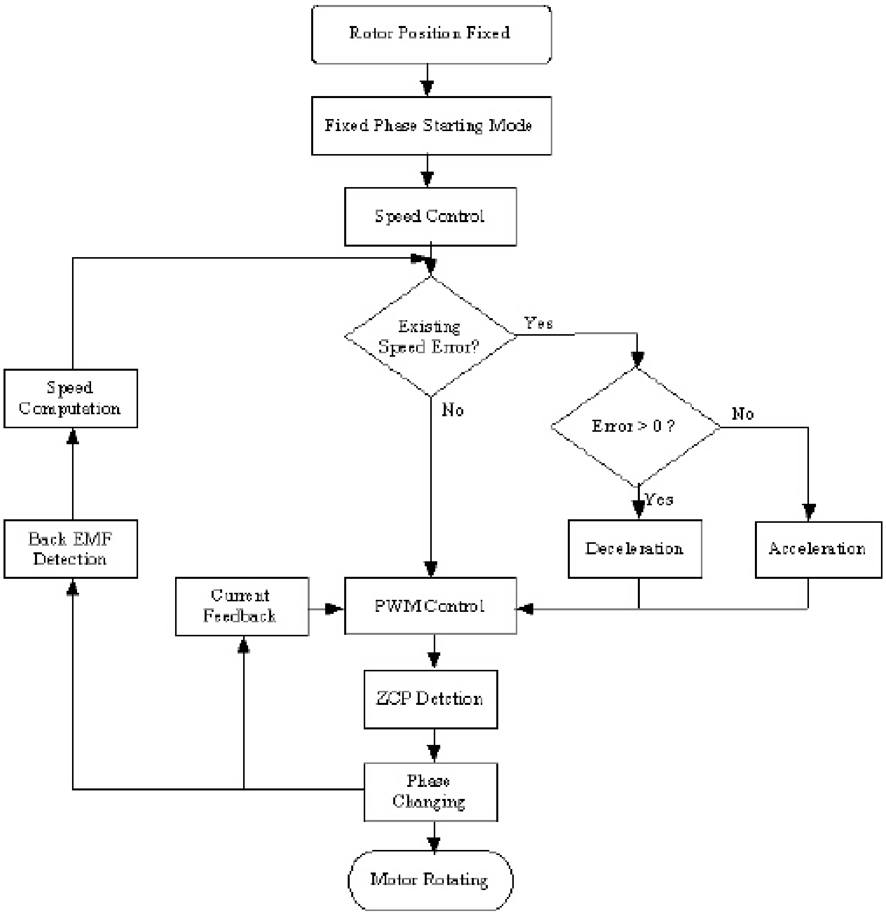

Figure 4 - Software Flow Diagram - example

Minimum content requirement: A flowchart that shows how your software would work. Standard symbols should be used.

6 Power Usage Description

In this section, describe your design's power usage by populating the attached Power Profile template [CDPDPowerProfileTemplate.xlsx, .ods]. For each component/part that uses power, you must estimate its power usage during expected operation. The expected operational sequence for power usage estimation is:

(1) Receive a message from Astrobee
(2) Drive any 3 motors & all sensors simultaneously for 15 min
(3) Wait for a message from Astrobee (60 min)
(4) Drive Motor #1 (M1) & Sensor #1 (S1) for 20 min
(5) Send a message to Astrobee

Table 2 shows an example of a filled in power profile.

Step 1: Description of power modes

	Power Mode Description
**Mode 1**	Only Board A on
**Mode 2**	Boards A, D & C on
**Mode 3**	Boards A & B on

**Step 2: List of Powered Elements**		***Mode 1*** Current (A)	***Mode 2*** Current (A)	***Mode 3*** Current (A)	***Mode 4*** Current (A)	***Mode 5*** Current (A)
**1.0**	**Element #1**					
	Board A	0.1	0.1	0.1		0.1
	Board B			0.2		
	Component #1				0.01	

Step 3: Power Profile for Typical Operations

	Which Power Mode	Time On (s)	Energy (W)
**(1) Receive and process "attach" command from Astrobee**			
Action #1 - to prepare for processing command by	Mode 1	30	0.01
Action #2 - process command from Astrobee by ...	Mode 5	10	0.01

Table 2 - Power Profile Example

Please continue to use the same names of components used in your Component List.

Minimum content requirement: Paste a figure of your filled in tables for the Power Profile into this document. No additional text is required.

7 Exit Survey

To complete your submission, please take the Exit Survey by going to this webpage: https://seasgwu.qualtrics.com/jfe/form/SV_2r9DaeSlh48uMcZ

At the end of the survey you will receive a unique code. In your submission include this section and the text: Exit Survey for Freelancer <<insert Freelancer username>> complete per completion code: <<insert completion code>>.

To be complete, your submission must include the following text: Exit Survey for Freelancer <<insert Freelancer username>> complete per completion code: <<insert completion code>>.
