# Supplementary material for: Linking solver characteristics, solving processes and solution attributes: A data explainer for an open innovation generated robotic design dataset
Source: Data Brief. 2023 Sep 6;50:109547. doi: 10.1016/j.dib.2023.109547 (PMC10518673; doi:10.1016/j.dib.2023.109547)
Supplement: Supplementary file 1 [file mmc1.zip › Release/Process/Challenge Rules/D5-SDM/SDM Blurb.docx]

Simple Deployment Mechanism (SDM)

In this challenge, you are asked to design a Simple Deployment Mechanism (SDM). This challenge is focused on the actuator selection and mechanism design and does not require detailed consideration of mounting, housing, control or electronics.

How it works: The SDM must be capable of performing two high-level actions: 1) displace a point mass from it’s starting point (origin) to a set destination 2) return that point mass back to its origin.

*Click on the links below to see detailed design instructions, constraints and solution templates for this problem.*

Challenge rules: A prize of **$250** will be awarded for the **lowest mass, technically feasible** solution, submitted by **June 14^th^ 2018**. No working prototype is required for submission, but the design must be sufficiently detailed to allow experts to assess the feasibility of your design (i.e., comply with all requirements) and the credibility of your mass estimate. Only complete submission packages will be evaluated.

Attachments:

SDMProblemDescription.pdf

SDMSubmissionGuidelines.pdf

Templates

- SDMMassTemplate [.xlsx, odt, [google docs](https://docs.google.com/spreadsheets/d/1w2PsqO7JIBbBMrGJ3Om6uEazsEzVssahdN57TwaJbHE/edit?usp=sharing)]

Steve, if you need the full link text. Here’s mass: <https://docs.google.com/spreadsheets/d/1w2PsqO7JIBbBMrGJ3Om6uEazsEzVssahdN57TwaJbHE/edit?usp=sharing>

Actual Final Text Pasted from Freelancer Description:

Design a Simple Deployment Mechanism (SDM)

In this challenge, you are asked to design a Simple Deployment Mechanism (SDM). This challenge is focused on the actuator selection and mechanism design and does not require detailed consideration of mounting, housing, control or electronics. 

How it works: The SDM must be capable of performing two high-level actions: 
1) displace a point mass from it’s starting point (origin) to a set destination 
2) return that point mass back to its origin. 

Click on the links below to see detailed design instructions, constraints and solution templates for this problem. 
The SDMMassTemplate may be submitted in any of the 3 formats provided: XLSX, ODS, or Google Sheets (<https://docs.google.com/spreadsheets/d/1w2PsqO7JIBbBMrGJ3Om6uEazsEzVssahdN57TwaJbHE/edit?usp=sharing>)

Challenge rules: 
The prize will be awarded for the lowest mass, technically feasible solution, submitted by the contest deadline. 
No working prototype is required for submission, but the design must be sufficiently detailed to allow experts to assess the feasibility of your design (i.e., comply with all requirements) and the credibility of your mass estimate. 
Only complete submission packages will be evaluated (see attachments).

NASA may select multiple winners or provide additional prize compensation on entries that are particularly novel or innovative.

NASA will be available to respond to clarifying questions, but feedback on quality is otherwise limited. 
All complete submissions will be confirmed with 3-star ratings. 
Note that final judgement of quality and winners will only happen after the submission deadline.
