# Supplementary material for: Linking solver characteristics, solving processes and solution attributes: A data explainer for an open innovation generated robotic design dataset
Source: Data Brief. 2023 Sep 6;50:109547. doi: 10.1016/j.dib.2023.109547 (PMC10518673; doi:10.1016/j.dib.2023.109547)
Supplement: Supplementary file 1 [file mmc1.zip › Release/Process/Challenge Rules/D5-SDM/SDM Problem Description.pdf]

1 Contest Description

In this challenge, you are asked to design a Simple Deployment Mechanism (SDM). **This challenge is focused on the actuator selection and mechanism design and does not require detailed consideration of mounting, housing, control or electronics.** The installation and linkage to the rest of the system will be handled separately. The SDM receives an electrical control signal to move an actuator, back and forth, between a stowed and deployed position. The below specification details how the SDM will work, its functional requirements, and interface constraints/assumptions. A separate document provides detailed guidelines on how your design must be presented and submitted.

A prize of $250 will be awarded for the lowest mass, technically feasible solution, submitted before 13:00 GMT on June 14th 2018.

2 Concept of Operations – How the SDM needs to work

The SDM must be capable of performing two high-level operations: 1) displace a point mass from it's starting point (*origin*) to a set *destination* 2) return that point mass back to its origin.

The coordinate system used is illustrated in Figure 1. The requirements for each operation (underlined) and intermediate state (italicized) are detailed in section 3.

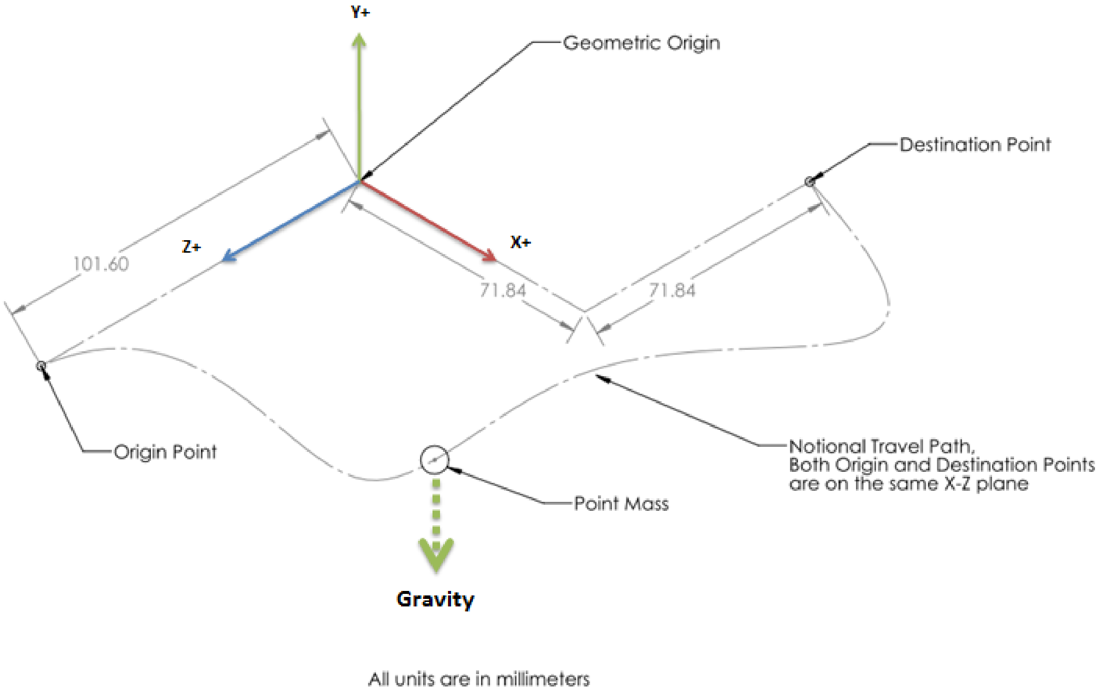

Figure 1 – SDM Concept of Operations

3 Functional Requirements

This section details all of the requirements that the SDM must meet.

3.1 Motion Requirements

R1 Displace: The SDM shall be able to move a point mass from an *origin point* to a fixed *destination point* as seen in Figure 1. The path shown in the figure is notional; the actual path is up to you. Assume that a link between the actuator output and the point mass will be designed separately (by someone else) and will match the needs of your design.

- R1.1 The point mass and its mass properties are defined in section 4.4
- R1.2 The origin point is (X, Y, Z) = (0 mm, 0 mm, 101.6 mm) or [0”, 0”, 4”].
- R1.3 The destination point is (X, Y, Z) = (71.84 mm, 0 mm, -71.84 mm) or [2.82”, 0”, -2.82”]
- R1.4 The point mass displacement must be accurate to within +/- 2.5mm [0.1”] in every Cartesian direction.

R2 Return: The SDM shall be able to move the point mass from the *destination point* back to the *origin point*. Accuracy requirements are equivalent to those specified in displace (see R1)

3.2 Resource Requirements

3.2.1 Timing Requirements

R3 Time to Displace:

- R3.1 The SDM shall be able to displace in no more than 30 seconds
- R3.2 The SDM shall be able to displace in no less than 5 seconds.

R4 Time to Return:

- R4.1 The SDM shall be able to return in no more than 30 seconds
- R4.2 The SDM shall be able to return in no less than 5 seconds.

3.2.2 Power Requirements

You may assume that power is applied to all active elements as needed to drive them. The details of the interface and bus voltage are described in C2.3.

R5 Energy Budget: The SDM shall not use more than 1.5 Watt-hours to support all operations. Assume a maximum of 1 minute of operations.

3.3 Environmental Requirements

R6 The SDM shall operate in an atmosphere comparable to that of Earth. Assume 21 degrees centigrade [70 degrees Fahrenheit], with low humidity, and pressurized to 100 kPA [750 mm Hg, 14.5 psi].

R7 The SDM shall not contribute any particulates (e.g. dust) to the ISS atmosphere.

R8 The SDM shall enclose all lubricated components to prevent lubricants from leaking into the atmosphere of the ISS.

4 Interface Requirements

The SDM you design will be integrated into a separately designed casing. You are not responsible for designing the interface, but your design must meet the constraints defined below.

4.1 SDM Volume

C1 Constraint 1 (C1) SDM Volume constraint: The SDM shall fully fit within the volume defined in Figure 2 at all times (i.e., any stroke length or other moving parts must remain within that cube).

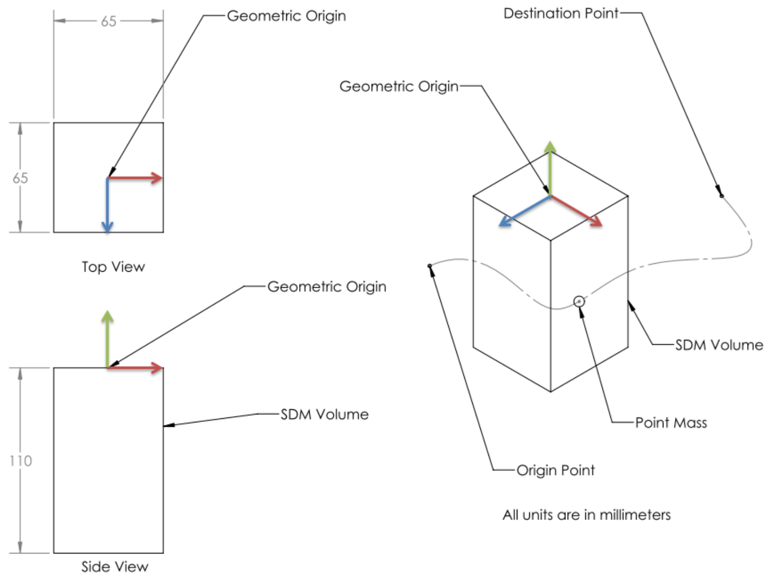

Figure 2 – SDM Volume

4.2 Electrical Power Interface:

C2 Available bus voltage is 14 +/- 0.1 Volts DC

C3 Max current: The SDM shall not draw more than 3 Amps peak.

C4 Steady State Current: The SDM shall not draw more than 2 Amps at steady state.

C5 There is a common ground for the system.

4.3 Control Interface:

C6 Astrobee can power any actuator. Here, actuators are defined as any device that moves a design element (e.g., solenoid, hydraulic or electrical motor, piezo-motor, shape memory alloy or polymer, compressed spring etc.).

C7 You must specify how the actuator should be activated (e.g., wiring diagram for electrically driven actuators).

4.4 Point Mass

C8 Point Mass Definition: The Point Mass is an abstract representation of the robotic arm that the SDM will be integrated with. Treat it as a point mass of 3.6 Kg being moved perpendicularly to normal gravity (9.81 m/s^2). In other words, gravity creates a cross-axial moment on your actuator, but normal motion does not need to work against it.

C9 The Point Mass will be rigidly connected to the Active Element of the SDM.
