# Supplementary material for: Linking solver characteristics, solving processes and solution attributes: A data explainer for an open innovation generated robotic design dataset
Source: Data Brief. 2023 Sep 6;50:109547. doi: 10.1016/j.dib.2023.109547 (PMC10518673; doi:10.1016/j.dib.2023.109547)
Supplement: Supplementary file 1 [file mmc1.zip › Release/Process/Challenge Rules/D5-SDM/SDM Submission Guidelines.pdf]

Submission Guidelines for the Simple Deployment Mechanism (SDM)

In this contest, you were asked to design a Simple Deployment Mechanism (SDM). This document provides detailed guidelines on how you must describe and present each aspect of your design in order to be considered for the prize. This document looks long but very little text is required. It's mostly pasting in figures and tables in a structured way. Your submission document must include each of the sections detailed below and all of the information requested in each. Several templates and examples are provided to clarify what constitutes a complete solution.

Use the exact section and subsection header words, shown below.

1 FUNCTIONAL DESCRIPTION 2

1.1 NARRATIVE (WORD) DESCRIPTION OF DESIGN 2
1.2 FUNCTIONAL ANALYSIS 2

2 PHYSICAL DESIGN 3

2.1 MASS SUMMARY AND COMPONENT LIST 3
2.2 DESIGN LAYOUT 3
2.3 WHAT WE NEED TO KNOW TO OPERATE YOUR SDM 3

3 EXIT SURVEY 4

1 Functional Description

1.1 Narrative (word) description of design

In this section, describe how your design for the Simple Deployment Mechanism (SDM) works. In a few sentences, please describe how your solution does each of the following:

1) Displace: How does your SDM move the point mass (through the separately designed linkage) from the origin point to the destination point?
2) Return: How does your SDM move the point mass (through the separately designed linkage) back to the origin point?

Although it is not required, you may embed images with sketches, models, storyboards or other illustrations in your written descriptions to help explain how your DSM design accomplishes these high-level operations.

Minimum content requirement: Text reponse to each of the above questions.

1.2 Functional Analysis

In this section, describe your logic and/or analysis for the following aspects of your SDM design. Including equations and mathematics is acceptable if it helps clarify the logic behind your design, but please ensure that it will be understood by our reviewers by annotating your process or describing the math being done and why.

(1) What torque does your actuator need to generate to move the point mass?
(2) How does your SDM design achieve the +/- 2.5mm position accuracy required per R1.4?
(3) How does your SDM handle the cross-axial moment induced by gravity?
(4) How long does it take your SDM to move the point mass from the origin point to the destination point (R3)?

Mimumum content requirement: Text responding to each of the above questions.

2 Physical Design

2.1 Mass Summary and Component List

In this section, list all the elements of your SDM solution using the template provided [SDMMassTemplate available as google doc, .odt, .xlsx]. For each component/piece/part, include an estimate of its mass and a brief explanation of where the estimate came from. Please be sure to include the reasons supporting your mass estimate for each element since they will be part of the evaluation of the credibility of your SDM mass estimate.

The below table provides an example of how the template should be filled in.

			Is this a powered component?	Estimated Mass per Unit (kg)	Quantity (# units)	Mass (kg)	Basis of Estimate
**1.0**	**Subsystem #1**					**1.18**	
	Switch #1 & 2		Yes	0.030	2	0.06	Called some former coworker who builds these, and asked for a typical masses
	Mechanism #1		No	0.800	1	0.80	Made a CAD model, assumed SS316, to obtain this mass
	Attachment hardware		No	0.040	8	0.32	Typical mass of component that I use all the time in design of systemX.
**2.0**	**Subsystem #2**					**0.91**	
	2.01 Assembly #1					0.49	
		Actuator #1	Yes	0.150	1	0.15	Weighed a prototype I built

Minimum content requirement: Paste your filled table into this section of the document. No additional text is required.

2.2 Design Layout

In this section, include an engineering-style drawing showing that your solution can fit in the volume constraint (C1). You should show your SDM at the point when a component or moving part of the SDM is the closest to exceeding the volume constraint (C1); this might be at one of the origin or destination points, or at some point on your chosen path between them. Your drawing should specify how all the components listed in your Component List (section 2.1) connect to one another. You can use any CAD software you like, sketch by hand, or photograph a prototype, but accurate dimensions of the whole SDM are required.

Minimum content requirement: One design drawing. The figure must be clearly labeled and dimensioned.

2.3 What we need to know to operate your SDM

In this section, tell us what we need to know to operate your design and integrate it with the rest of Astrobee. For each actuator or device that moves an element (e.g., solenoid, hydraulic or electrical motor, piezo-motor, shape memory alloy or polymer, compressed spring etc.), tell us what kind of device it is and what is required to operate it. If it uses an off-the-shelf actuator driver, simply list it. Otherwise, tell us exactly what is required (e.g., the precise electrical signals) to generate and control motion in both directions.

Minimum content requirement: Text describing 1) the type of actuator; 2) how the actuator is operated (e.g., list off-the-shelf driver)

3 Exit Survey

To complete your submission, please take the Exit Survey by going to this webpage: https://seasgwu.qualtrics.com/jfe/form/SV_2r9DaeSlh48uMcZ

At the end of the survey you will receive a unique code. In your submission include this section and the text: Exit Survey for Freelancer <<insert Freelancer username>> complete per completion code: <<insert completion code>>.

To be complete, your submission must include the following text: Exit Survey for Freelancer <<insert Freelancer username>> complete per completion code: <<insert completion code>>.
