# Supplementary material for: Linking solver characteristics, solving processes and solution attributes: A data explainer for an open innovation generated robotic design dataset
Source: Data Brief. 2023 Sep 6;50:109547. doi: 10.1016/j.dib.2023.109547 (PMC10518673; doi:10.1016/j.dib.2023.109547)
Supplement: Supplementary file 1 [file mmc1.zip › Release/Process/Challenge Rules/D4-PSA/PSA Problem Description.pdf]

1 Contest Description

In this challenge, you are asked to design the Positioning Software Architecture (PSA) that will control a Robotic Arm (RA) that has been separately designed to attach the Astrobee Robotic Free Flyer to a Handrail within the International Space Station. The PSA will receive a high-level positioning command from Astrobee and implement it through the RA's control electronics that will drive the RA's motion. The PSA should include the motion-planning algorithms necessary for the RA to (1) move from Astrobee's payload bay and move to a commanded location, and (2) stow back into the payload bay, without making contact with any parts of Astrobee or the ISS.

Section 2.1 describes how your PSA is expected to operate. Sections 2.2 and 4 below describe a reference design of the RA and its control electronics (such as locations of joints, ranges of motion and low-level motion control modes for all Robotic Arm actuators). Section 3 provides details on specific required functionality of your PSA. A separate document provides guidelines on how your PSA design must be presented and submitted.

Note that no executable code is required, but your proposed software architecture must be sufficiently descriptive to allow experts to assess its feasibility (i.e., comply with all constraints and requirements) and follow the prescribed format.

A prize of **$250** will be awarded for the **lowest cyclomatic complexity, technically feasible** solution, **submitted before 21:00 GMT on July 16th 2018.**

2 Concept of Operations – How the PSA needs to work

The PSA will be used to develop software that moves a Robotic Arm to a precise location when given a high-level motion command by Astrobee, while avoiding contact with objects in its proximity. The two specific, high-level motion commands your PSA must receive and execute are described in detail below (Section 2.1).

The Robotic Arm has 6 independent degrees of freedom, each driven by its own rotary actuator. You may assume the RA and all its associated control electronics hardware have been already designed. Relevant details on the design and performance of the RA and its control electronics are included in Sections 2.2 and 4.

The Robotic Arm's control electronics suite contains the processing components on which your software will run in realtime. All low-level, hardware-dependent driver software are also designed elsewhere, and a description of the software interface to the actuator driver is included in Section 4.1. Hardware-dependent software includes all drivers for controlling each actuator, as well as for sensors that provide knowledge of actuator state (such as position, speed or torque).

2.1 Normal Operations

The PSA must be able to control the robotic arm to perform two motion operations under normal conditions when commanded by Astrobee:

(1) MoveTool(x,y,z): This command is intended to move the Robotic Arm from a stowed configuration in Astrobee's payoad bay to an extended position that places the free end (the "Tool") of the RA at a specific point without passing into the two designated "keepout zones" – one to prevent contact with other Astrobee components, and the second to prevent contact with other parts of the ISS.

(2) StowTool: This command is intended to move the Robotic Arm back to a stowed position within the Astrobee Payload bay, while avoiding the "keepout zones" described above.

Figure 1 illustrates the high-level operations the PSA will be responsible for.

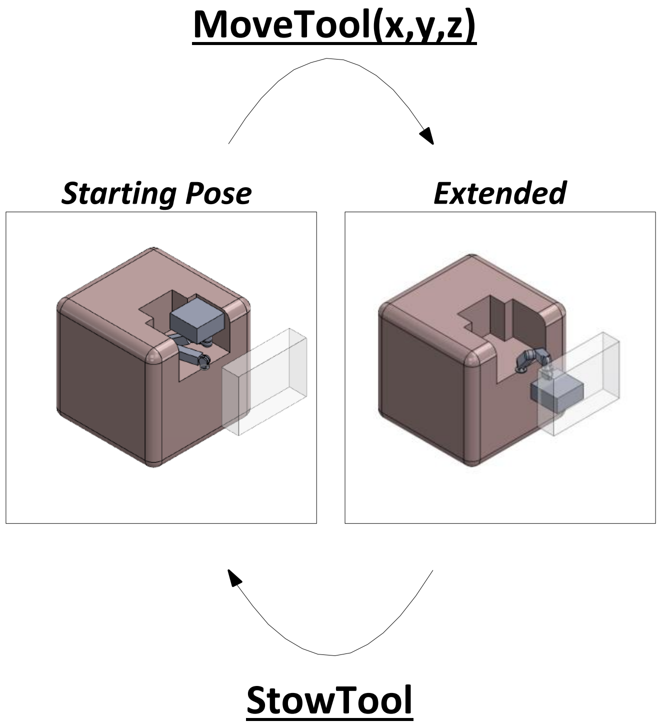

Figure 1 – Concept of Operations

2.2 Robotic Arm System Mechanical Description

The Robotic Arm has 6 independent joints, each driven by a rotary actuator (A1 – A6). Each actuator is connected by 5 rigid links. Figure 2 and Figure 3 show the orientation of each actuator and link. A1 is mounted directly to the Astrobee Payload bay, and is at the origin of the fixed coordinate frame for all RA motion. This location is indicated as "**O**" in Figure 3. The output of the last actuator in the series, A6, is fixed to a volume representing the Tool that is attached to the Robotic Arm.

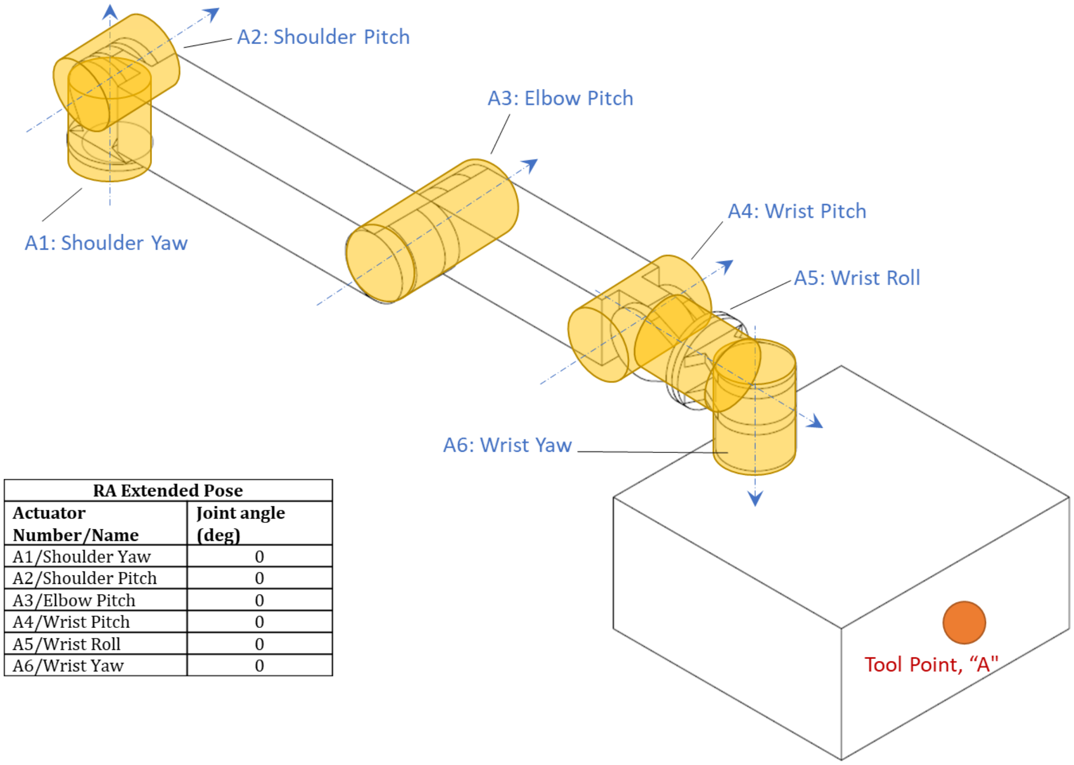

RA Extended Pose	
**Actuator Number/Name**	**Joint angle (deg)**
A1/Shoulder Yaw	0
A2/Shoulder Pitch	0
A3/Elbow Pitch	0
A4/Wrist Pitch	0
A5/Wrist Roll	0
A6/Wrist Yaw	0

Figure 2 – Robotic Arm (RA) Actuator Layout

Note that we are following the "right-hand rule" convention for positive rotation of each actuator (blue arrows in Figure 2). This assumes a counter clockwise rotation about the blue arrow is a positive change in angular position; and a clockwise rotation corresponds to a negative change in angular position.

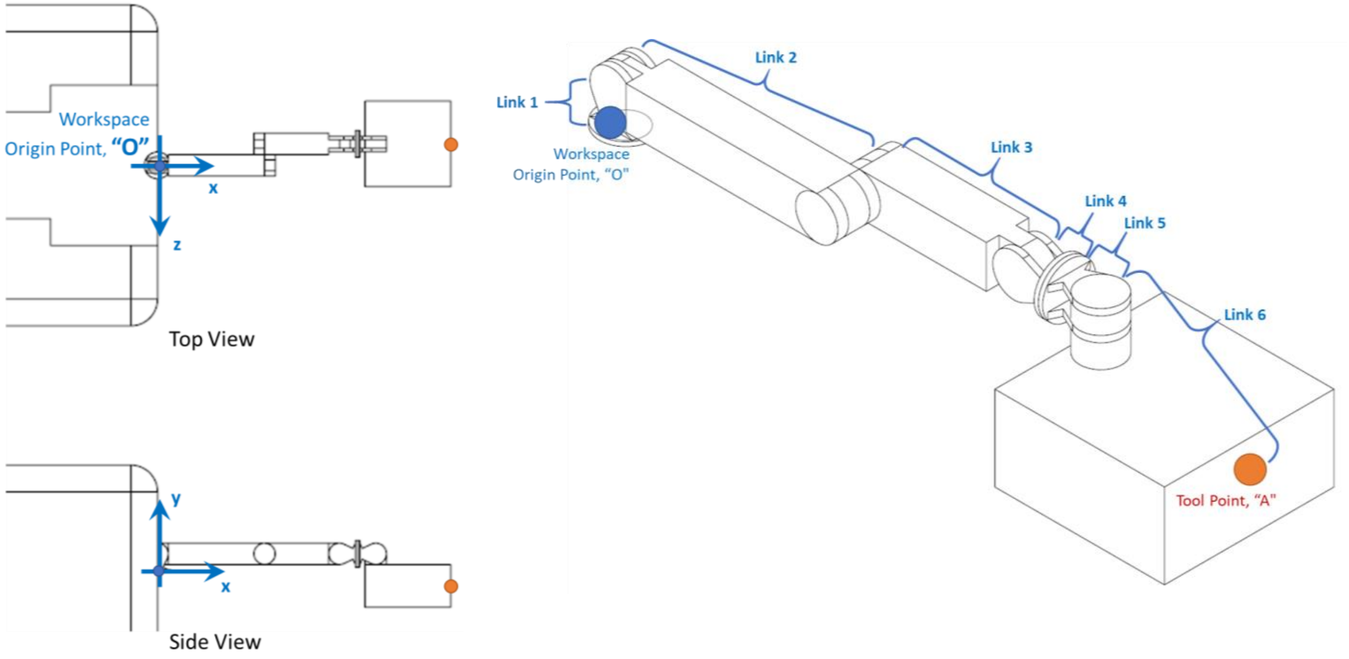

Figure 3 - Robotic Arm (RA) Link Layout

Table 1 - Link Lengths in Workspace Coordinate Frame (Figure 3)

Link #	Δx (mm)	Δy (mm)	Δz (mm)
Link 1	0	17	0
Link 2	100	0	-20
Link 3	70	0	0
Link 4	17	0	0
Link 5	17	0	0
Tool (Point "A")	70	-38	0

3 Functional Requirements

This section details the functional requirements that the PSA must meet.

3.1 Motion Requirements

R1 MoveTool(**x,y,z**): The PSA shall be able to control the RA to move the "Tool" (defined by point "A" in Figure3) from a Starting Pose, where the RA is stowed in the Astrobee payload bay, to the **x,y,z** position in the workspace without contacting with Astrobee or the mounting structure of the ISS Handrail.

R1.1 Starting Pose: The RA will start in the following pose (illustrated in Figure 4). Specific actuator positions for this RA configuration are included in Table 2.

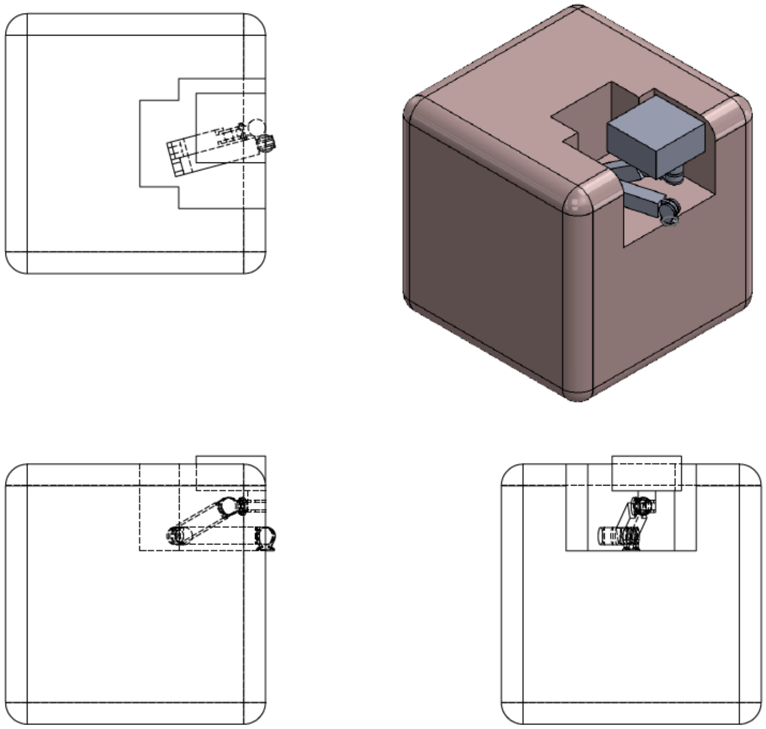

Figure 4 – Robotic Arm in Starting Pose

Table 2 – Robotic Arm Starting Pose Actuator Positions

Actuator Number/Name	Joint angle (degrees)
A1/Shoulder Yaw	15
A2/Shoulder Pitch	-180
A3/Elbow Pitch	150
A4/Wrist Pitch	30
A5/Wrist Roll	0
A6/Wrist Yaw	-15

R1.1 Tool Workspace (range for **x,y,z**): The RA will be given a command to place the Tool (defined by point "A" in Figure 2) in a region as enveloped by:

150 mm $\leq$ **x** $\leq$ 200 mm

-25 $\leq$ **y** $\leq$ 125 mm

-100 $\leq$ **z** $\leq$ 100 mm

The coordinate frame for this Workspace is coincident with where the RA mounts to the Astrobee payload bay, at point "O" in Figure 3. This is also the location of the rotational frame of reference for A1, Shoulder Yaw actuator in Figure 2.

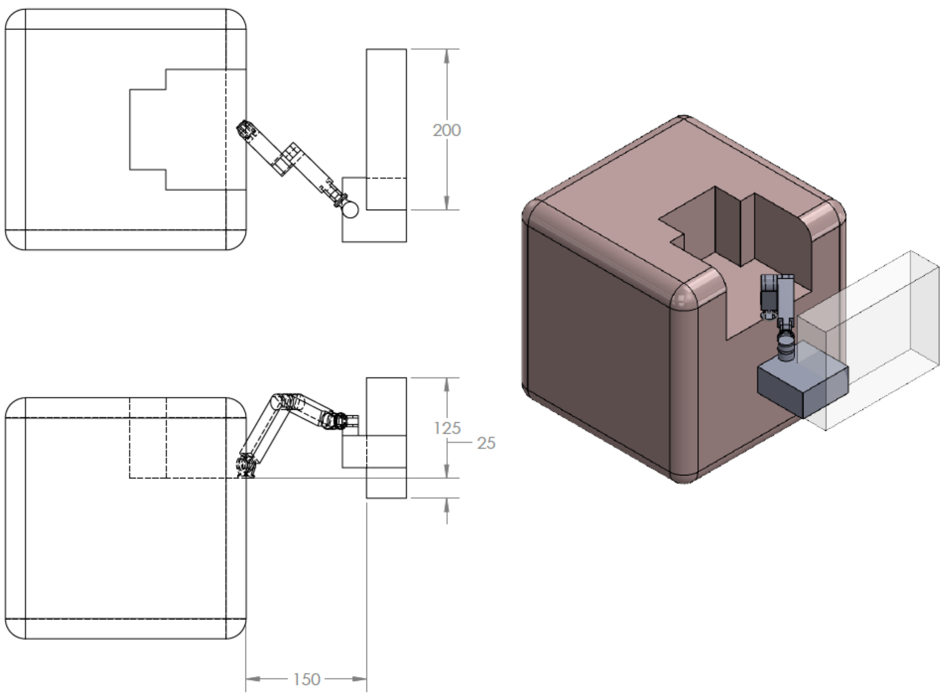

Figure 5 – Tool Workspace with extended RA (dimensions in mm)

R1.2 Tool position accuracy: The RA should be able to place the Tool (free end of the RA) within 6 mm of the specified location, i.e. (x ± 6, y ± 6, z ± 6).

R1.3 Tool orientation: The RA should be able to place the Tool at the specified location, (**x**,**y**,**z**), with an orientation of the Tool such that the plane with Point A on it is parallel to the front face of Astrobee, as illustrated in Figure 5.

R1.4 Maximum Tool Speed: For all motion planned for the RA, the Tool (defined by point "A" in Figure 3) should never exceed a speed of 1.6 cm/s relative to the Astrobee

R1.5 Maximum time to move to location: The PSA shall plan MoveTool motion such that the move is completed within 15 min.

R1.6 Motion Confirmation: The PSA shall send a confirmation of successful completion of motion when MoveTool is complete. Specific format is defined in section 4.1 (C6).

R2 StowTool: The PSA shall be able to command the RA to move from any position within the Tool Workspace back to the Starting Pose, stowed within the Astrobee Payload bay.

R2.1 Clearance to Payload Bay: When placing the RA back into the Payload bay, planned motion must maintain clearance of at least 8 mm in between any element of the RA (including the Tool) and any interior wall of the Payload Bay.

R2.2 Maximum time to move to stow: The PSA shall plan StowTool motion such that the RA is stowed into the Payload bay within 15 min.

R3 Collision avoidance: The RA must not make contact with any objects in the workspace while moving. This is a critical aspect of the PSA, and your approach to meeting this requirement should be clearly described in your submission.

R3.1 RA Volume: The physical dimensions of the Robotic Arm components are found in Figure 7.

R3.2 Keepout Zones: Two keep out zones are defined in Figure 6. They represent the Astrobee (Zone 1) and areas close to the ISS Handrail (Zone 2).

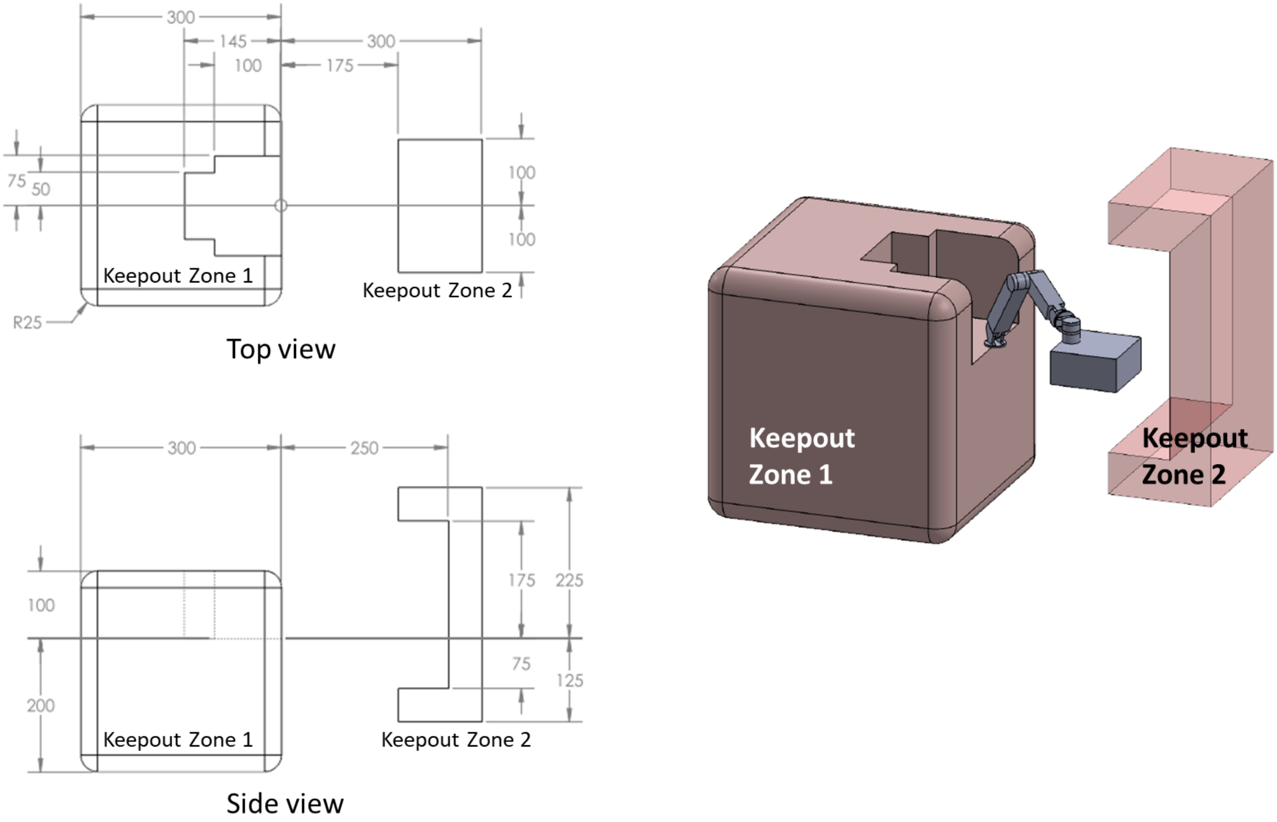

Figure 6 – Keepout Zones: (1) Near Astrobee; (2) Near Handrail

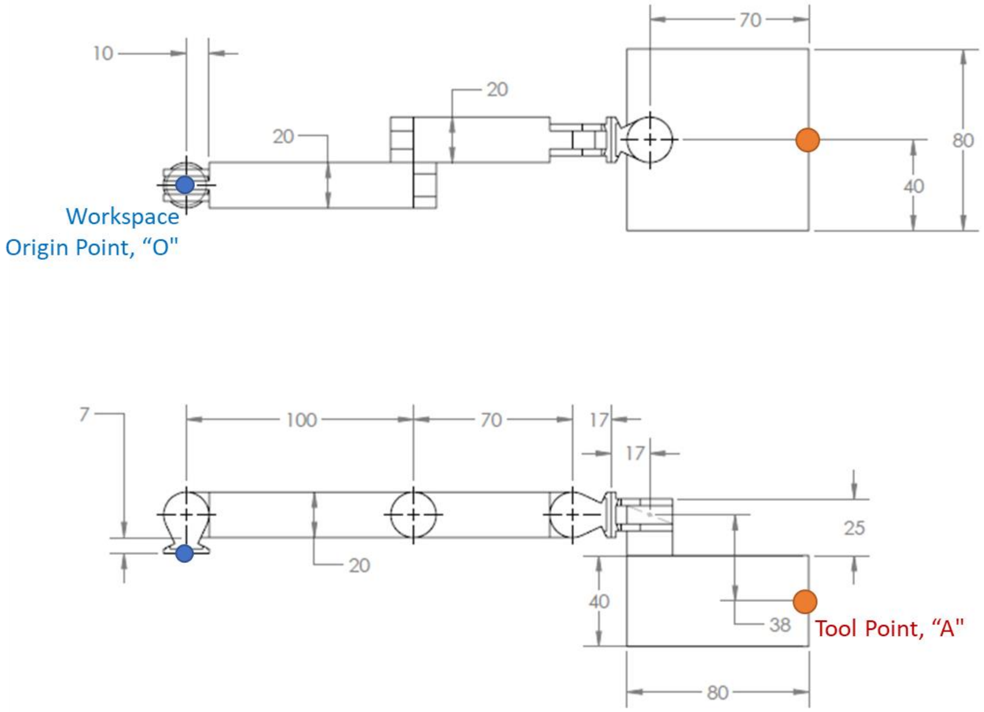

Figure 7 - Physical Dimensions of Robotic Arm

R4 Energy Limit: The PSA shall not command the RA to perform any motion operation (MoveTool or StowTool) that would use more than 12 watt-hours. Specific power constraints for each motion control mode for each RA actuator are included in the Interface Requirements (Section 4).

4 Interface Requirements

The PSA has interfaces to a separately designed robotic arm, through a control electronics hardware suite, as well as the with Astrobee. The section describes all constraints imposed by those interfaces.

4.1 System Power and Data Block Diagram

As described above, for your PSA design, you may assume a generic suite of motion control electronics hardware. This control suite also contains the software drivers for lower-level, hardware-specific motion control elements such as joint motors and position sensors. Figure 8, below, captures the primary elements of the control electronics suite that may be used by PSA for Robotic Arm motion planning. And specifically, we expect your PSA to run on the RPU.

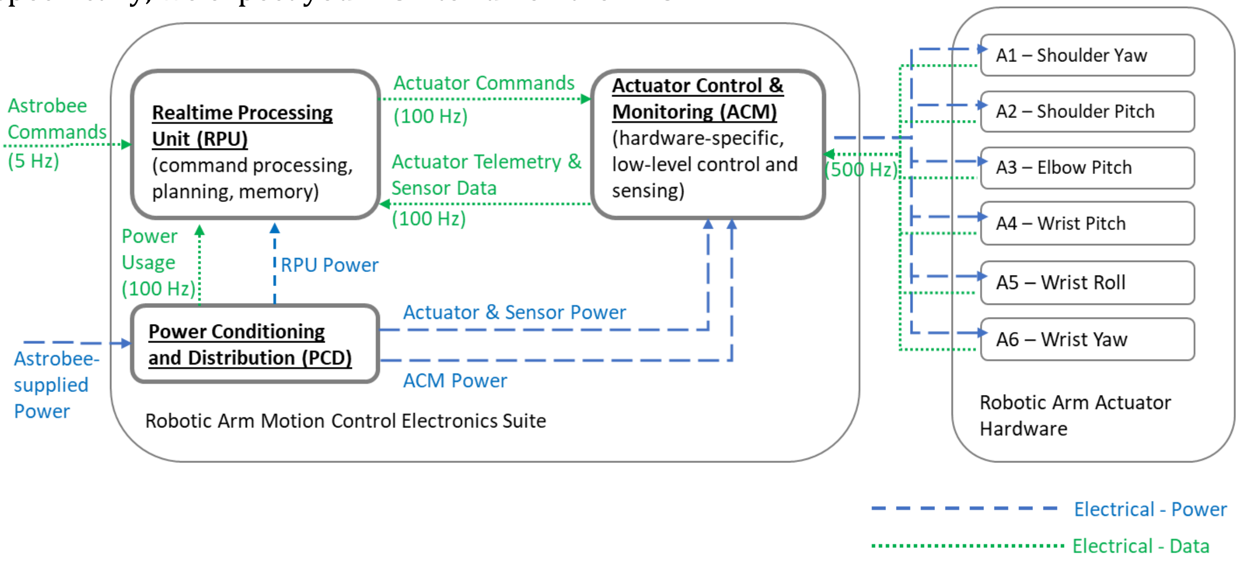

Figure 8 -. RA Motion Control Electronics Suite

We are not providing a particular API for the individual components of the RA motion control electronics suite, however we provide software interface information below. This includes specific commands, telemetry streams, function libraries, etc.

4.2 Actuator Control

This section describes the performance limits of each actuator and functional modes available to drive each of the 6 actuators in the RA through the ACM.

C1 RA actuator control properties – Range of Motion (or permissible actuator positions), acceptable speeds, and control uncertainties are summarized in Table 3.

Table 3 - Actuator Control Details

Actuator Number/Name	Position Range	Position Uncertainty	Speed Range	Speed Uncertainty	Average Power
A1/Shoulder Yaw	-90° ↔90°	±1.5 deg	0 – 3 rpm	±0.5 deg/s	0.5 W
A2/Shoulder Pitch	-180° ↔ 90°	±1.5 deg	0 – 3 rpm	±0.5 deg/s	0.5 W
A3/Elbow Pitch	-180° ↔ 180°	±1.5 deg	0 – 3 rpm	±0.5 deg/s	0.5 W
A4/Wrist Pitch	-165° ↔ 165°	±1.5 deg	0 – 5 rpm	±0.25 deg/s	0.3 W
A5/Wrist Roll	-180° ↔ 180°	±1.5 deg	0 – 5 rpm	±0.25 deg/s	0.3 W
A6/Wrist Yaw	-180° ↔ 180°	±1.5 deg	0 – 5 rpm	±0.25 deg/s	0.3 W

C2 Table 4 summarizes the single-actuator modes of operation available to PSA for control of the RA. These low-level actuator control modes do not return any values indicating success or failure. For your PSA design, you may assume faults or errors from the ACM, PCD or Astrobee are handled elsewhere.

Table 4 - Actuator Motion Control Modes

Actuator Control Mode	Description	Inputs to ACM	Format for ACM input	Example for actuator, A1
Velocity mode	Drives actuator at specified velocity for a specified time	Direction (CW, CCW)*; Speed (deg/s); Total time (s);	AX_vel_(direction, speed, total time)	A1_vel_(CCW, 1.3, 65)
Position mode	Drives actuator to a specified position at a specified velocity	Position (deg); Velocity (deg/s);	AX_pos_(position, velocity)	A1_pos_(42, 0.7)

*CW= Clockwise; CCW = Counter Clockwise

C3 Multi-actuator, or simultaneous motion is permitted, within the power limitations (power limit listed in C4). Note that average power associated with driving each actuator (column #6 in Table 3) includes power associated with all the necessary sensors and electronics. Note that you may assume average actuator power only, and ignore larger transients, such as start-up current spikes.

4.3 Power Interface

C4 Max Power: The PSA shall not draw more than 2 W average power while performing either moveTool (R1) or stowTool (R2) operations.

4.4 Data Interfaces

Data types identified in Power & Data Block Diagram above (Figure 8) are described in more detail in this section.

C5 Data available for your PSA design from all actuators and dedicated sensors in the ACM and PCD at 100 Hz is summarized below in Table 5. In the table, details are given for Actuator 1 as an example; data and format for all other actuators is available for your PSA design with a change in the actuator number. For example, the data stream name for the position for Actuator 4 would be, “A4_pos” .

Table 5 - Actuator Telemetry, Sensor & Power Usage Data

Data Stream Name	Description	Units/Format	Source
A1_pos	Actuator #1 output position	degrees	ACM
A1_spd	Actuator #1, speed	deg/s	ACM
A1_dir	Actuator #1, direction	CW or CCW	ACM
A1_power	Actuator #1, power	W	PCD
ACM_power	ACM module power	W	PCD
RPU_power	RPU module power	W	PCD
PCD_power	PCD module power	W	PCD

C6 Commands (received and transmitted) to and from Astrobee shall be serial and formatted in ASCII using the RS-232 protocol. They are specified in Table 6.

Table 6 – Format for commands to/from Astrobee

Command format	Action
Received from Astrobee to PSA	
"moveTool(x,yz)"	Moves RA to location, (x,y,z) (R1)
"stowTool"	Return Robotic Arm to Starting Pose in Payload bay (R2)
Sent from PSA to Astrobee	
"MoveToolConfirmed"	Confirm moveTool operation has completed
"stowToolConfirmed"	Confirms stowTool operation has completed
