# Supplementary material for: Linking solver characteristics, solving processes and solution attributes: A data explainer for an open innovation generated robotic design dataset
Source: Data Brief. 2023 Sep 6;50:109547. doi: 10.1016/j.dib.2023.109547 (PMC10518673; doi:10.1016/j.dib.2023.109547)
Supplement: Supplementary file 1 [file mmc1.zip › Release/Process/Challenge Rules/D4-PSA/PSA Blurb.docx]

# Positioning Software Architecture (PSA)

In this challenge, you are asked to design the Positioning Software Architecture (PSA) that will control a Robotic Arm (RA) that has been separately designed to attach the Astrobee Robotic Free Flyer to a Handrail within the International Space Station. This challenge is focused on software and software architectures, and no executable code is required.

How it works: The PSA will receive a positioning command from Astrobee and implement it through the RA’s control electronics that will drive the RA’s motion. The PSA should include the high-level motion-planning algorithms necessary for the RA to (1) move from Astrobee’s payload bay and move to a commanded location, and (2) stow back into the payload bay, without making contact with any parts of Astrobee or the ISS.

*Click on the links below to see detailed design instructions, constraints and solution templates for this problem.*

Challenge Rules: A prize of **$250** will be awarded for the **lowest cyclomatic complexity, technically feasible** solution, **submitted before 21:00 GMT on July 16^th^ 2018.** No working code is required for submission, but the software architecture design must be sufficiently detailed to allow experts to assess the feasibility of your design (i.e., comply with all requirements). Only complete submission packages will be evaluated.

# Attachments:

PSA Problem Description.pdf

PSA Submission Guidelines.pdf
