# Supplementary material for: Linking solver characteristics, solving processes and solution attributes: A data explainer for an open innovation generated robotic design dataset
Source: Data Brief. 2023 Sep 6;50:109547. doi: 10.1016/j.dib.2023.109547 (PMC10518673; doi:10.1016/j.dib.2023.109547)
Supplement: Supplementary file 1 [file mmc1.zip › Release/Process/Challenge Rules/D2-SFA/SFA Blurb.docx]

# Smart Fine Positioning Arm (SFA)

In this contest, you are asked to design a “Smart” Fine-positioning Arm (SFA) that will be mounted to Astrobee and move an attached tool to a specific location and command that tool to attach to an ISS Handrail. The SFA receives all power and high-level commands from Astrobee, but implements the following functions autonomously: stowing and deploying from a payload volume, positioning its free end at a specified location near an Handrail and orienting Astrobee by panning and tilting it.

How it works: initially, the SFA will be packed in a stowed configuration. When powered and commanded by the Astrobee, The SFA must be able to autonomously perform three high-level functions. Each function requires a combination of performing operations itself and controlling the operations of the attached tool, as described below:

**Attach**, starting with Deploy, which involves the SFA unpacking from its *stowed* configuration in Astrobee’s payload bay and moving to a self-determined location in *freespace*; commanding the tool to “unpack” and standing by for a confirmation signal; place, which involves the SFA placing its free end (and the attached tool) at a specified *pre-attach* location near a Handrail; and then commanding the tool to “close” and standing by for a confirmation signal; and relaying that “attachconfirm” signal to Astrobee.

**Orient**, which involves the SFA panning and tilting Astrobee, as commanded, while the tool *secures* it to the Handrail; and sending a confirmation when each pan and tilt completes.

**Stow**, starting with commanding the tool to “release” and standing by for a confirmation signal; withdraw, which involves the SFA withdrawing the tool from the *pre-attached* configuration and moving it to *freespace*; commanding the tool to “pack” and standing by for a confirmation signal; and then stow, which involves the SFA repacking in Astrobee’s payload bay (*stowed configuration*); and sending a confirmation of stow to Astrobee.

*Click on the links below to see detailed design instructions, constraints and solution templates for this problem.*

Challenge Rules: A prize of **$4000** will be awarded for the **lowest mass, technically feasible** solution submitted by September 5^th^, 2018. No working prototype is required for submission, but the design must be sufficiently detailed to allow experts to assess the feasibility of your design (i.e., comply with all requirements) and the credibility of your mass estimate. Only complete submission packages will be evaluated.

Attachments:

SFAProblemDescription.pdf

SFASubmissionGuidelines.pdf

Templates

SFAMassTemplate [.xlsx .ods]

SFAPowerTemplate [.xlsx .ods]
