# Supplementary material for: Linking solver characteristics, solving processes and solution attributes: A data explainer for an open innovation generated robotic design dataset
Source: Data Brief. 2023 Sep 6;50:109547. doi: 10.1016/j.dib.2023.109547 (PMC10518673; doi:10.1016/j.dib.2023.109547)
Supplement: Supplementary file 1 [file mmc1.zip › Release/Process/Challenge Rules/D2-SFA/SFA Problem Description.pdf]

1 Contest Description

In this contest, you are asked to design a "Smart" Fine-positioning Arm (SFA) that will be mounted to Astrobee. A separately designed "Tool" will be mounted to the other (free) end of the SFA. This Tool acts like a hand, grasping the ISS Handrail ("Handrail") and holding on, when commanded. In designing the SFA this Tool should be considered as a black box with several defined volumes depending on what the SFA commands and specified power and control needs. After the SFA places the Tool and commands it to close, the SFA rotates the Astrobee in two directions.

The SFA receives all power and high-level commands from Astrobee, but implements the following functions autonomously: stowing and deploying from a payload volume, positioning its free end at a specified location near an Handrail, and orienting Astrobee by rotating it in two directions.

The below specification details how the SFA will work, its functional requirements and interface constraints/assumptions. A separate document provides detailed guidelines on how your design must be presented and submitted.

A prize of $4000 will be awarded for the lowest mass, technically feasible solution, submitted before 21:00 GMT on September 5th, 2018.

2 Concept of Operations – How the SFA needs to work

2.1 Normal Operations

The SFA must be able to autonomously perform three high-level functions through doing five operations, underlined in the below specification. Each function requires a combination of performing operations itself and controlling the operations of the attached Tool, as described below:

1) Attach:
   a. <u>Deploy</u>, which involves the SFA unpacking from its *stowed* configuration in Astrobee's payload bay and moving to a self-determined location in *freespace*;
   b. Commanding the Tool to "tool_unpack" and standing by for a confirmation signal;
   c. <u>Place,</u> which involves the SFA placing its free end (and the attached Tool) at a specified *pre-attach*;
   d. Commanding the Tool to "tool_close" and standing by for a confirmation signal; and relaying that "attachconfirm" signal to Astrobee.
2) Orient,
   a. <u>Pan</u>, <u>Tilt</u>: which involves the SFA independently rotating Astrobee in two directions as commanded by Astrobee.
3) Stow:

a. Commanding the Tool to "tool_release" and standing by for a confirmation signal;
b. <u>Withdraw</u>, which involves the SFA withdrawing the Tool from the *pre-attached* configuration and moving it to *freespace*;
c. Commanding the Tool to "tool_pack" and standing by for a confirmation signal;
d. <u>Stow</u>, which involves the SFA repacking in Astrobee's payload bay (*stowed configuration*); and sending a confirmation of stow to Astrobee.

<u>Standby</u> is a low powered waiting mode used between deploy and place, place and orient, orient and withdraw, and withdraw and stow.

The relationships among the configurations (italicized), operations (underlined), commands to and from the Astrobee (blue dashed arrows), and commands to and from the Tool (red solid arrows) are illustrated in Figure 1. The coordinate system and definitions of certain elements are seen in Figure 2. The requirements for each operation, configuration, and command are detailed in Section 3.

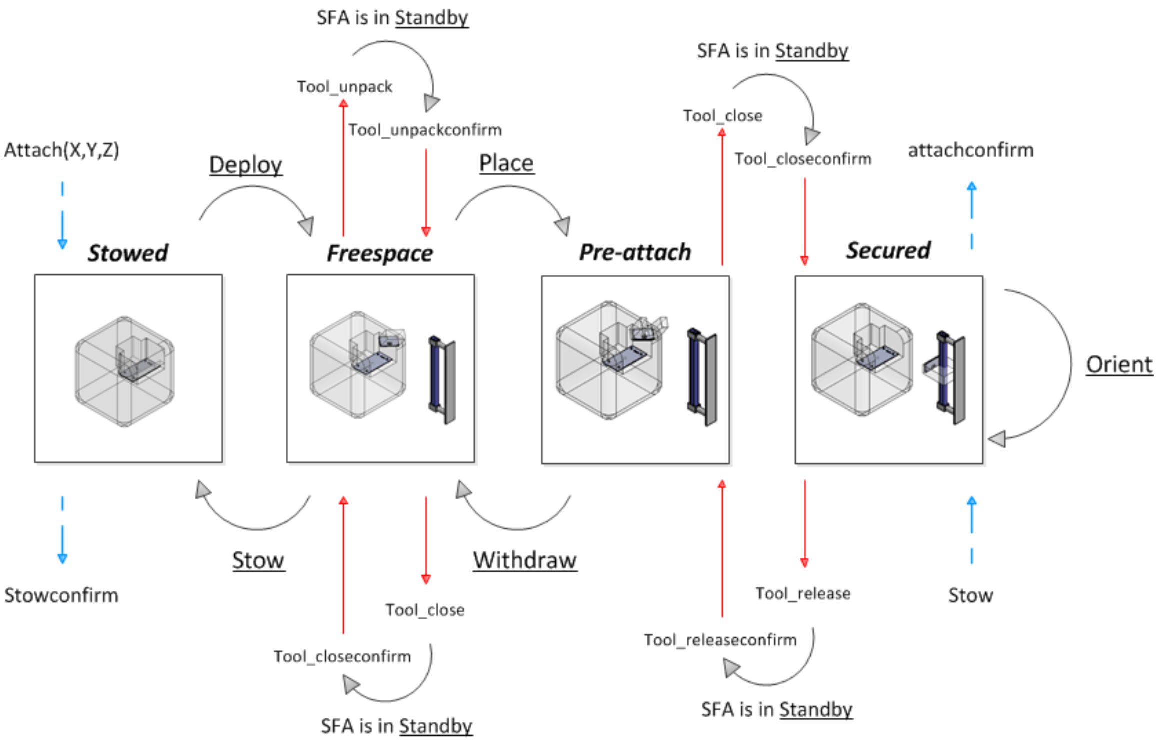

Figure 1 - SFA Concept of Operations

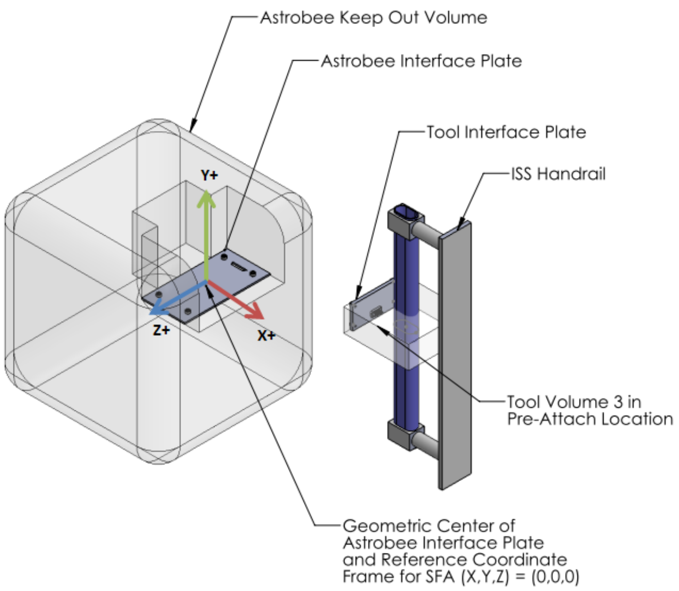

Figure 2 - Concept of Operations Workspace and Coordinate Frame

2.2 Contingency (Emergency) Operations

There are several scenarios when normal operations may be disrupted. The ranges of permissible responses are detailed in Section 3.6. This section summarizes those scenarios: 1) when the SFA experiences higher than expected loads during orienting (e.g., because an astronaut or other object bumps or contacts Astrobee) and 2) when an astronaut manually pulls on the SFA to remove the system from the handrail.

3 Functional Requirements

This section details all the requirements that the SFA must meet and the operations it must perform. To clearly define the motion involved in these operations, we have defined a coordinate reference frame that has its origin at the geometric center of the Astrobee Interface Plate (as seen in Figure 2). Since many of the configurations and volumes are formally defined in the interface requirements (section 4), their names and a brief description are listed here:

- *Tool Volume #1:* Occupied by the Tool that the SFA commands at the beginning of deploy and the smallest volume that the Tool might occupy (C11.1)
- *Tool Volume #2:* The maximum volume that the Tool might occupy while moving when either in freespace or while attaching to the ISS Handrail (C11.2)
- *Tool Volume #3:* Occupied by the Tool when it is ready to be placed at the Handrail (C11.3).
- *ISS Handrail*: The ISS Handrail found at location x,y,z as reported by the Astrobee with attachment hardware to the wall of the International Space Station(C21).

3.1 Motion Requirements

R1 Deploy: The SFA shall be able to move from the Astrobee's Payload Bay (C1.2) to a self-determined point in *freespace* configuration without itself, or the attached Tool Volume #1 (C11.1), contacting Astrobee or the ISS Handrail(C21).

R1.1 *Freespace* is a location in space that you select as part of your design for the free end of the SFA. This location is subject to the following constraints: it is a) outside Astrobee's Payload Bay and b) the attached Tool Volume #2 (C11.2)cannot contact any obstacles (as during the attach function, the Tool Volume will change from #1 to #3, never expanding larger than Tool Volume #2).

R1.2 *Obstacles* shall include a) Astrobee Keep Out Zone (C1.1) b) The ISS Handrail. It's attachment to the wall are defined in (C21). The location of the ISS Handrail is defined by the Cartesian coordinate (X, Y, Z), relative to the SFA baseplate, in the "attach" command issued by Astrobee (see R7).

R2 Place: The SFA shall be able to place the *Tool Position* of Tool Volume #3 (C11.3) to the *pre-attach* configuration (the Tool Position being located at a coordinate x,y,z) anywhere within the workspace defined below, without contacting the ISS Handrail at that location.

R2.1 Placing workspace: The SFAs workspace is defined by the Cartesian coordinates (x, y, z) centered at the geometric center of the Astrobee interface plate, such that:

- 265 mm $\leq x \leq$ 315 mm
- -25 mm $\leq y \leq$ 125 mm
- -100 mm $\leq z \leq$ 100 mm

Figure 3 defines the reference coordinate system. The origin lies at the center of the SFA baseplate (see C2). The destination is the Tool position relative to the Tool Interface Plate (Figure 4)

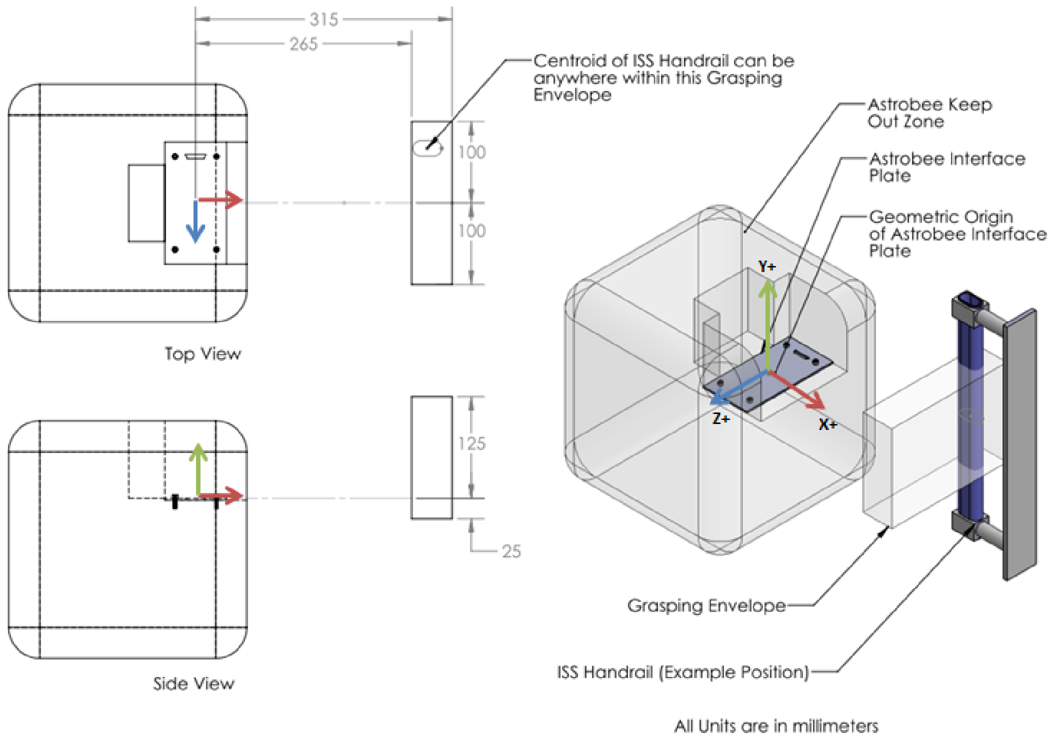

Figure 3 - Workspace Figure

R2.2 Tool Position and Error and Pre-Attach Configuration: When place ends in the pre-attach configuration, the Tool Position of Tool Volume #3 shall not be offset from the Handrail relative to the Tool Interface Plate's geometric center by no more than:
- x = 59 mm $\pm$ 6 mm
- y = 0 $\pm$ 6 mm
- z = 0 $\pm$ 6 mm
- θ_x = 0 $\pm$ 5 degrees
- θ_y = 0 $\pm$ 5 degrees
- θ_z = 0 $\pm$ 5 degrees

The coordinate system for these offsets is shown graphically in Figure 4 and is centered around the Tool Interface Plate geometry that you must provide (C12)

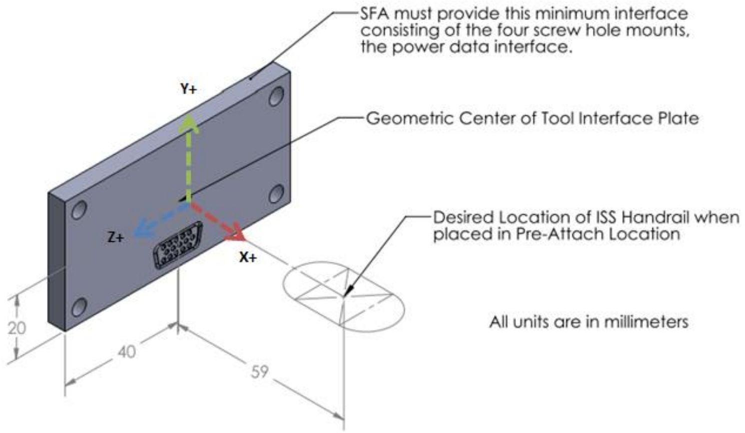

Figure 4 - Ideal location of ISS Handrail with Respect to the SFA- Provided Tool Interface Plate

R2.3 Collision avoidance: Tool Volume #3 shall not contact the Handrail or ISS wall while being placed.

R3 Orient: When commanded by Astrobee, the SFA shall be able to <u>Pan</u> and <u>Tilt</u> (rotate in two perpendicular directions) Astrobee about the geometric center of the Tool Interface Plate. Assume that the SFA is fixed to the Handrail through the Tool Interface (which could be anywhere within the grasping envelope R2.1).

R3.1 During orienting (after "attachconfirm" being sent to the Astrobee and before the stow command is received from the Astrobee), the SFA and Astrobee shall not contact the Tool Volume #3

R3.2 <u>Pan</u> and <u>Tilt</u> coordinates are measured with respect to the Tool Interface Plate coordinate system shown in Figure 5.

R3.3 Rotation for Pan and Tilt occurs about the center of the Tool Interface plate (Figure 13). Translation of the Astrobee is acceptable, along with rotation, if the Astrobee's final position is rotated about the center of the Tool Interface Plate.

R3.4 Minimum clearance between the moving components of the SFA, the Astrobee Keep Out Zone, and the Tool Volumes during these movements is 2 mm SFA and attached Tool and any element of the Astrobee.

R3.5 <u>Pan</u>: The SFA shall be able to pan through the range: -65° < θ_x < 65° , +/- 5° as detailed in Figure 5

R3.6 Tilt: the SFA shall be able to tilt through the range: 0° < θ_y < 90° , +/- 5° as detailed in Figure 5

R3.7 No simultaneous panning and tilting shall ever be requested.

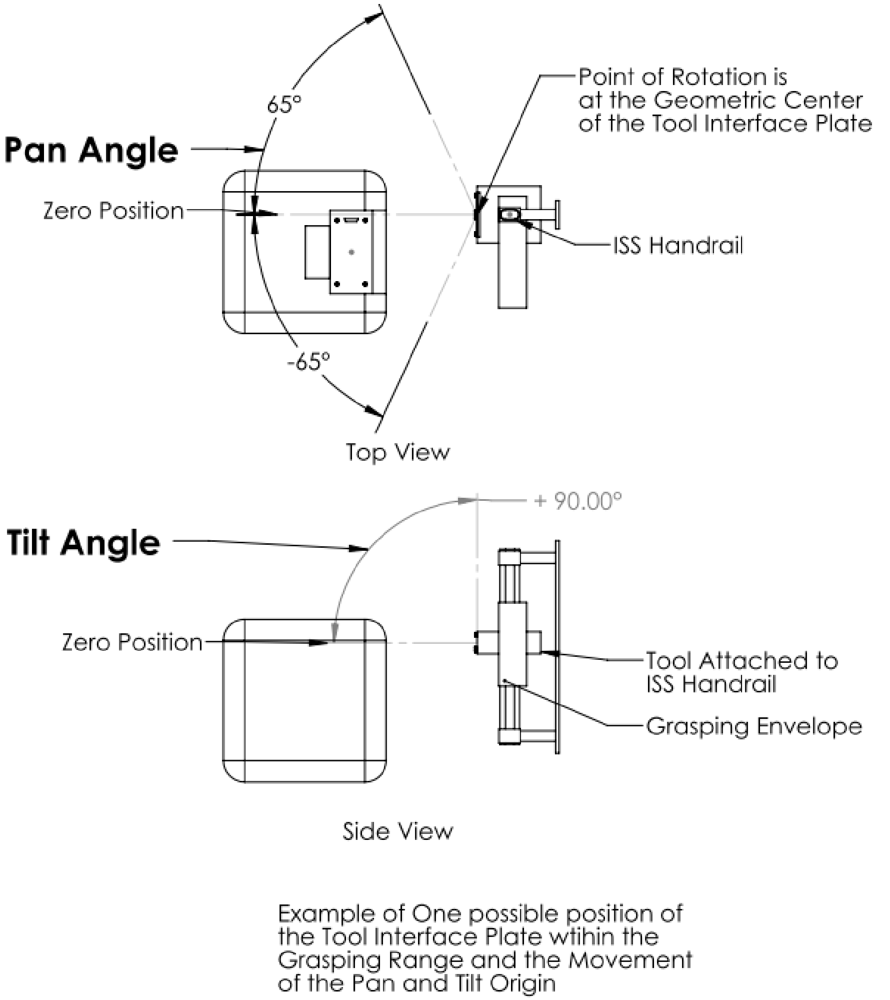

Figure 5 - Astrobee Tilting Angles

R4 Withdraw: The SFA shall be able to reverse the place operation, moving from the pre-attached configuration (see R2) to a self-defined position in *freespace* (R1).

R5 Stow: The SFA shall be able to return itself (with Tool Volume 1 attached – see C11.1) to the *stowed* configuration in Astrobee's payload bay (see C1.2).

R6 Standby: The SFA shall be able to enter a standby mode wherein it uses minimal power but can receive commands.

3.2 Control Requirements

The format of all commands sent by the Astrobee is specified in section C10. This section describes how the SFA shall be controlled. The SFA only receives four high-level commands from Astrobee and must execute the rest of its operations and interactions with the Tool autonomously. The SFA is also responsible to command the attached Tool to change its configurations and eventually attach (and release) from the ISS Handrail.

R7 When commanded to "attach(x,y,z)" the SFA shall perform the following sequence of operations and communications without further instruction from Astrobee:

1. The SFA shall deploy (R1).
2. Once in *freespace* (R1.1), the SFA shall:
    a. command the Tool Volume #1 to "tool_unpack" (C20) and
    b. enter standby (R6)
3. The SFA shall remain in standby until it receives an "tool_unpackconfirmed" signal from the Tool (C20) and then
4. Place (R2) the centroid of Tool Volume #3 at location X, Y, Z.
5. Once the place operation has completed:
    a. The SFA shall send an "tool_close" command to the Tool (C20)
    b. enter standby (R6)
6. The SFA shall remain in standby until it receives a "tool_closeconfirmed" signal from the Tool (C20) and then:
    a. The SFA shall send a "attachcomplete" signal to Astrobee and
    b. Enter standby (R6)

R8 When commanded to "pan(θ_x)"

1. The SFA shall complete the specified rotation θ_x
2. When panning is complete:
    a. the SFA shall send a "panconfirmed" signal to Astrobee and
    b. enter standby (R6)

R9 When commanded to "tilt(θ_y)"

1. The SFA shall complete the specified rotation θ_y
2. When tilting is complete:
    a. the SFA shall send a "tiltconfirmed" signal to Astrobee and
    b. enter standby (R6)

R10 When commanded to "stow," the SFA shall perform the following sequence without further instruction from Astrobee:

1. The SFA shall:
    a. Send a "tool_release" command to the Tool (C20) and
    b. enter standby (R6)
2. The SFA shall remain in standby until it receives a "tool_releaseconfirmed" signal from the Tool (C20) and then
3. The SFA shall withdraw (R4) to a self-defined position in *freespace (*R1.1*)*, where the SFA shall:
    a. Command the Tool to "tool_pack" (C20) and
    b. Enter standby (R6)
4. The SFA shall remain in standby until it receives a "tool_packconfirmed" signal from the Tool (C20) and then
5. The SFA shall:
    a. stow (R5) in Astrobee's payload bay and
    b. Send a "stowconfirmed" signal to Astrobee.

3.3 Resource Requirements

3.3.1 Timing Requirements

R11 Time to deploy and place: Combined, deploy and place operations shall not exceed 13 minutes and shall not take less than 1.5 seconds. The timing for deploy is measured from Astrobee's command to "deploy" to the SFAs command to "tool_unpack." The timing for place is measured from the SFA's reception of the signal of "tool_unpackconfimed" to sending the command "tool_close" combined with the receiving of "tool_closeconfirm" to the SFAs signal of "attachconfirmed."

R12 Time to orient:

c. The SFA shall be able to Pan 90 degrees in 15 seconds.
d. The SFA shall be able to Tilt 90 degrees in 15 seconds.
e. Combined pan and tilt operations shall not exceed 1 hour.

R13 Time to withdraw and stow: Combined, withdraw and stow operations shall not exceed 13 minutes and shall not take less than 1.5 seconds. The timing to withdraw is measured from Astrobee's command to "stow" to the SFAs command to "tool_release" combined with the time from receiving "tool_releaseconfirmed" to "tool_pack." The timing for stow is measured from the Tool's signal "tool_packconfirmed" to the SFAs signal "stowconfirmed."

R14 Time in standby: Shall not exceed four minutes during the attach operations (while SFA is waiting after deploying and place for the attached Tool) or the stow operations (while the SFA is waiting after withdraw and stow after waiting for the attached Tool to release and pack).

3.3.2 Energy Requirements

All power transmitted to the Tool is described in the interface Section 4.1.2.

R15 The SFA shall not use more than 30 Watt-hours of energy from all its operations. In calculating your energy budget, assume that pan and tilt are ongoing for 1 hr and Standby could be up to 8 minutes. The time for deploy, place, withdraw and stow are a function of your design.

3.4 Safety Requirements

R16 The SFA shall have no sharp edges, defined as a radius of 3 mm, for astronaut safety.

R17 The SFA shall have no loops of material greater than 25.4 mm in diameter for astronaut safety and unsupported or unattached for more than 40 mm from the structure of the SFA.

R18 The SFA shall not damage itself through normal operations.

R19 The SFA shall be able to return to its normal operations if power is shortly lost.

3.5 Environmental Requirements

R20 The SFA shall operate in the ISS zero gravity environment.

R21 The SFA, when unpowered, shall not be damaged by electrostatic discharge <4,000V.

R22 The SFA shall operate in an atmosphere comparable to that of Earth. Assume temperature of 21 °C [70 °F], and pressure of 101 kPa [1 atm], and relative humidity that is 40% - 70%.

R23 The SFA shall not contribute any particulates (e.g. dust) to the ISS atmosphere.

R24 The SFA shall enclose all lubricated components to prevent lubricants from leaking into the atmosphere of the ISS.

3.6 Contingency Requirements

R25 Excessive loads: This scenario may occur if an astronaut or piece of equipment contacts Astrobee while the SFA is secured at the handrail (including while experiencing normal operating loads per R2). The SFA shall maintain normal orient operations when subject to a force of up to 18N applied at the baseplate in the negative Y-direction and a simultaneous moment of 1.8 Nm about the Z-axis (see Figure 6a). This assumes that the Tool Interface Plate remains fixed in space.

R26 Astronaut intervention: Astrobee is required to be removable from the Handrail by an astronaut. Assume that for the SFA, this translates to a pull-away force of 35.6 N [8 lbf] in the negative X-direction (see Figure 6b) applied through the baseplate, with the Tool fixed in space. This will not occur during any other operation.

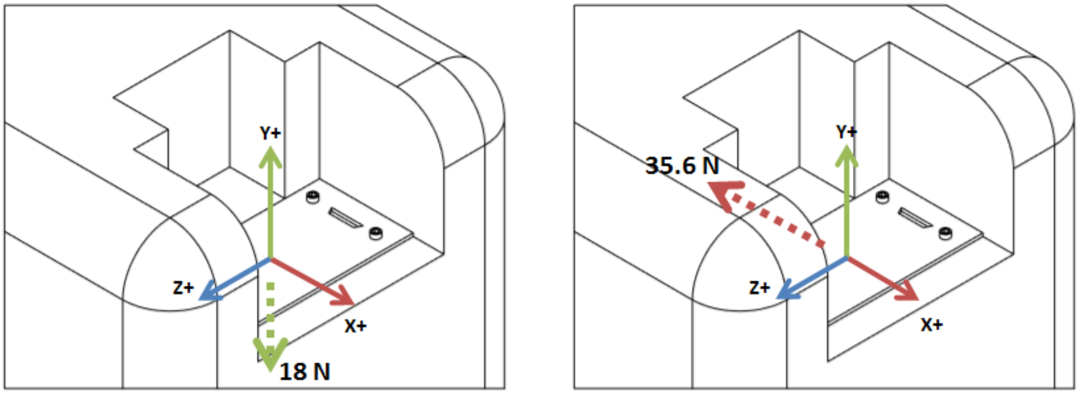

Figure 6 – Contingency Loads a) shows Excessive Loads; b) shows Astronaut intervention loads

4 Interface Requirements

The SFA has a fixed interface to Astrobee and the Tool. The section describes all constraints imposed by those interfaces.

4.1 SFA-Astrobee Interface

4.1.1 SFA-Astrobee Mechanical Interface

C1 Constraint 1 (C1) Volume Constraint: The SFA (and attached Tool) are stowed in Astrobee's payload bay in the *stowed* configuration. Figure 7 defines the payload bay with respect to Astrobee

- C1.1 Figure 8 defines the dimensions of the Astrobee volume that must not be contacted
- C1.2 Figure 9 defines the dimensions of the payload bay, which the SFA must stow within along with the attached Tool in Tool Volume 1 and the SFA is in the *stowed* configuration.

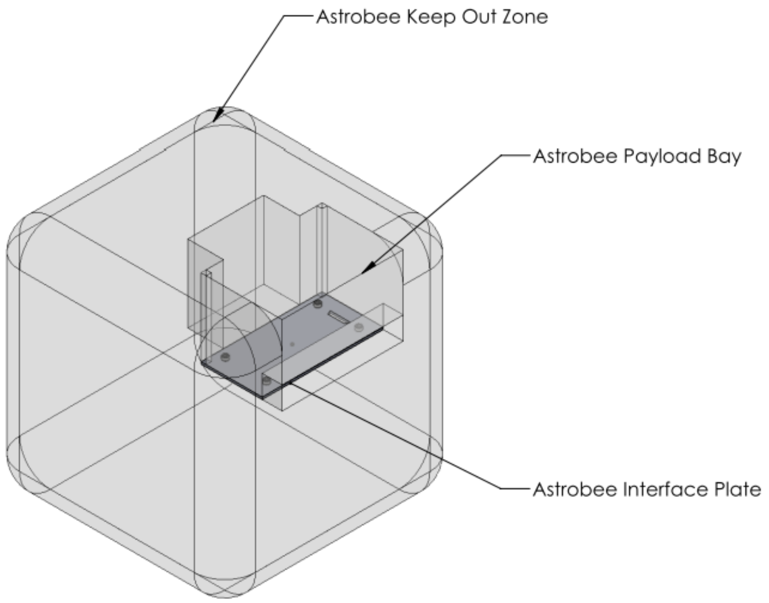

Figure 7 - Astrobee and Payload Bay

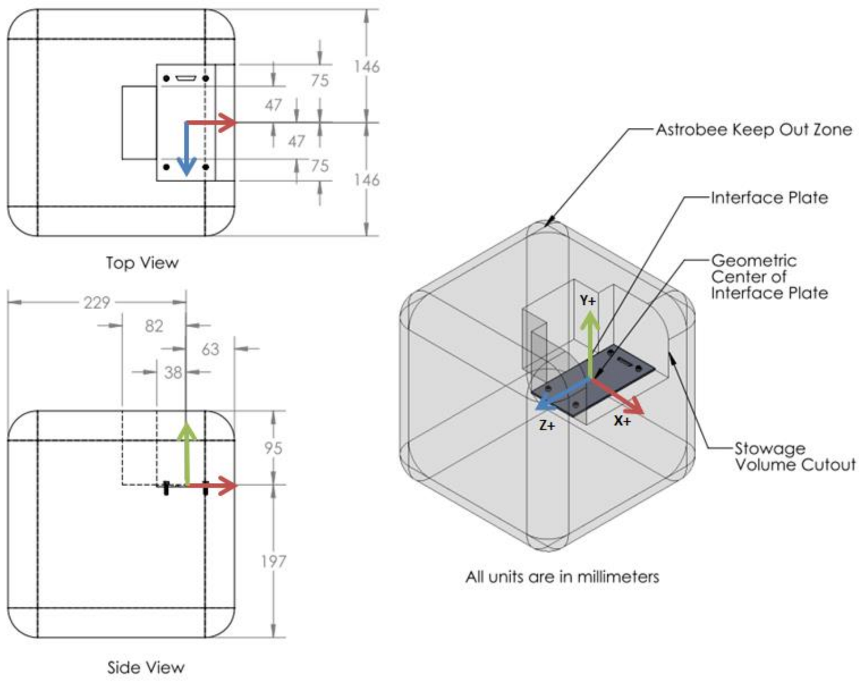

Figure 8 - Astrobee Keep-Out Zone and Adapter Plate

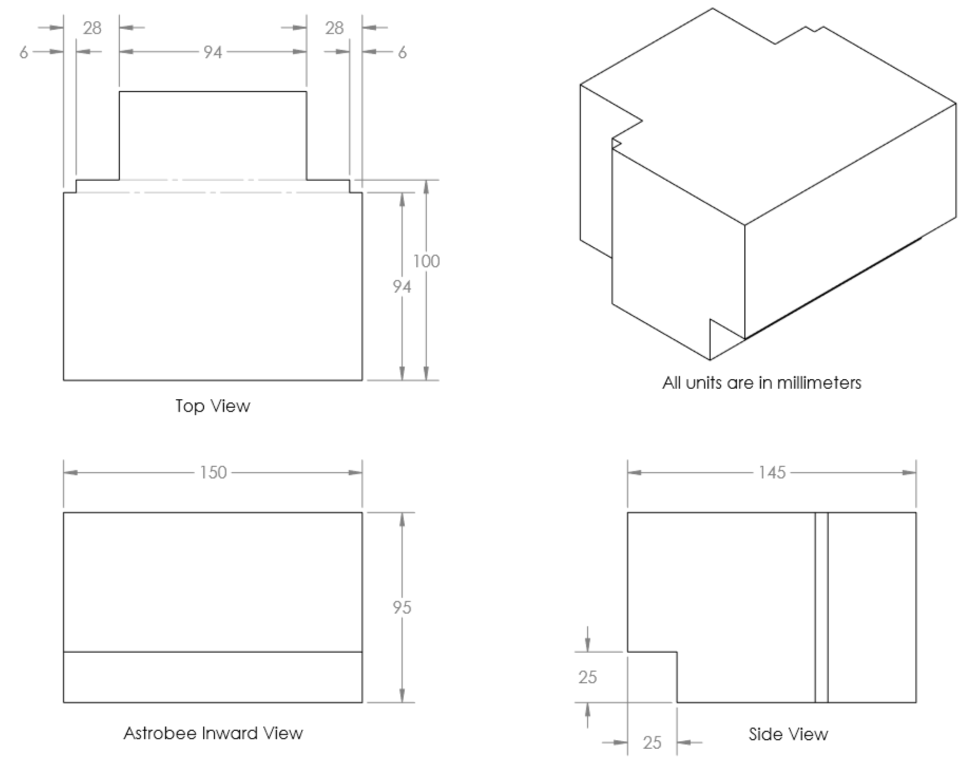

Figure 9 - Stowage Volume and Interface Plate

C2 Mounting interface: The SFA shall mechanically mount to a flat metal plate shown in Figure 10

- C2.1 All external loads are applied at the interface plate.
- C2.2 There are four available screw holes in the specified location.
- C2.3 Screws for your selected electrical connectors must only require hand tightening of locking screws of the adapter. Assume that no external loads are applied through the electrical connector.

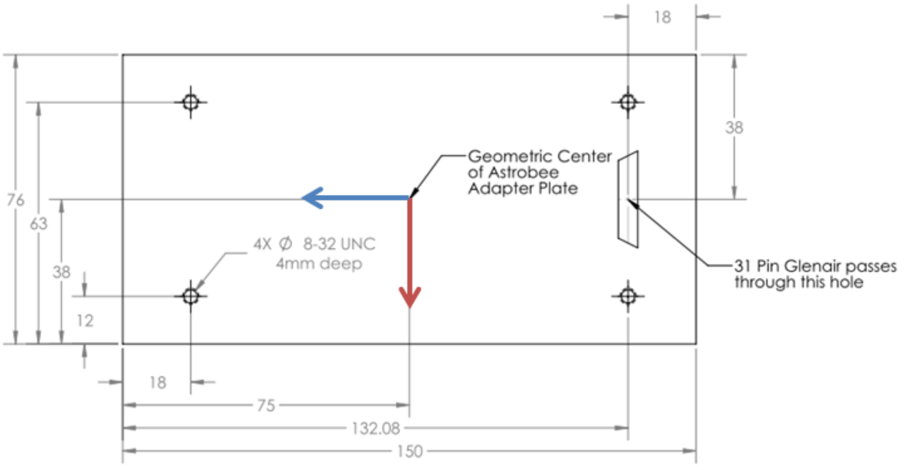

Figure 10 - SFA mounting interface

C3 Mass Properties: Treat Astrobee as mass of 6 Kg with center of mass of set (-83mm, -48 mm, 0 mm) relative to the SFA Coordinate Reference Frame as seen in Figure 2

4.1.2 Power Interface

C4 The SFA shall connect to the power and data connector shown in Figure 10. It is a 31-pin payload Glenair M83513/03-E03N connector located in the baseplate.

C5 Astrobee Bus Voltage + is normally 14.4 volts DC, but can vary between 11 to 17 volts DC.

C6 Max Current: The SFA shall not draw more than 3 A peak.

C7 Steady State Current: The SFA shall not draw more than 2 A at steady state.

C8 Pinout is as follows: Pin 4 is bus voltage. Pin 1 is ground.

4.1.3 Data Interface

C9 All commands will be received and transmitted as part of a serial command using the second and third pins of the 31-pin connector. The second pin is the positive serial command line, and the third pin is the negative serial command line.

C10 Commands (received and transmitted) shall be serial and formatted in ASCII using the RS-232 protocol once. They are specified in Table 1.

Table 1 – Command format

Command format	Action
Received from Astrobee to the SFA	
"attach(x,y,z)"	Initiate attach to a Handrail at specified location (R7)
"pan(α)"	Initiate pan for specified degrees (R8)
"tilt(β)"	Initiate tilt for specified degrees
"stow"	Initiate stow
Sent from SFA to Astrobee	
"attachconfirmed"	Confirm attach has completed (R7)
"panconfirmed"	Confirm pan has completed (R8)
"tiltconfirmed"	Confirm tilt has completed (R9)
"stowconfirmed"	Confirm stow has completed (R10)

4.2 SFA-Tool Interface

4.2.1 Mechanical SFA-Tool Interface

C11 Volume Assumptions: The Tool should be treated as a black box that can take on three specific envelopes associated with operations (defined in R1):

- C11.1 Tool Volume #1 (TV1) is defined in Figure 11 as 80 mm x 80 mm x 40 mm.
- C11.2 Tool Volume #2 (TV2) is defined in Figure 12 as 121 mm x 114 mm x 50 mm
- C11.3 Tool Volume #3 (TV3) is defined in Figure 13as 114 mm x 102 mm x 40 mm with a cutout to permit placement on the Handrail.

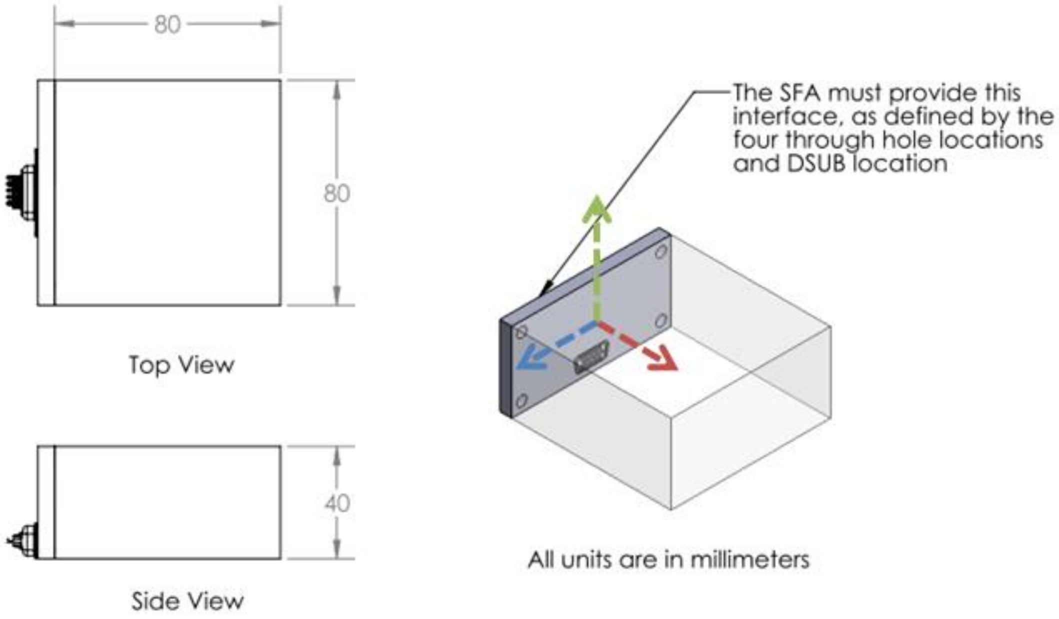

Figure 11 - Tool Volume 1, the Stowed Tool

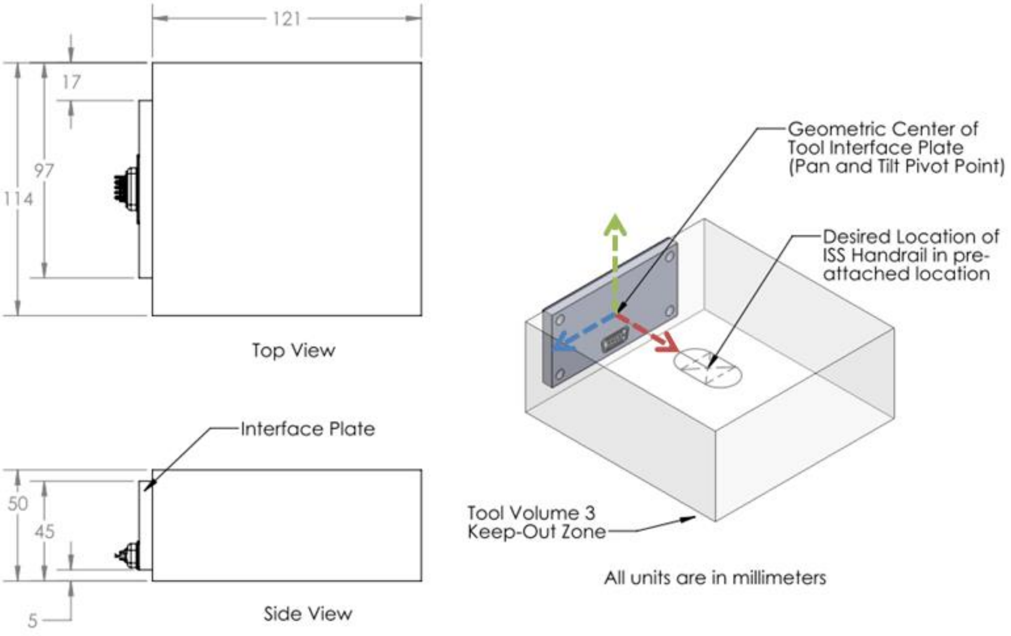

Figure 12 - Tool Volume 2, Dynamic Volume

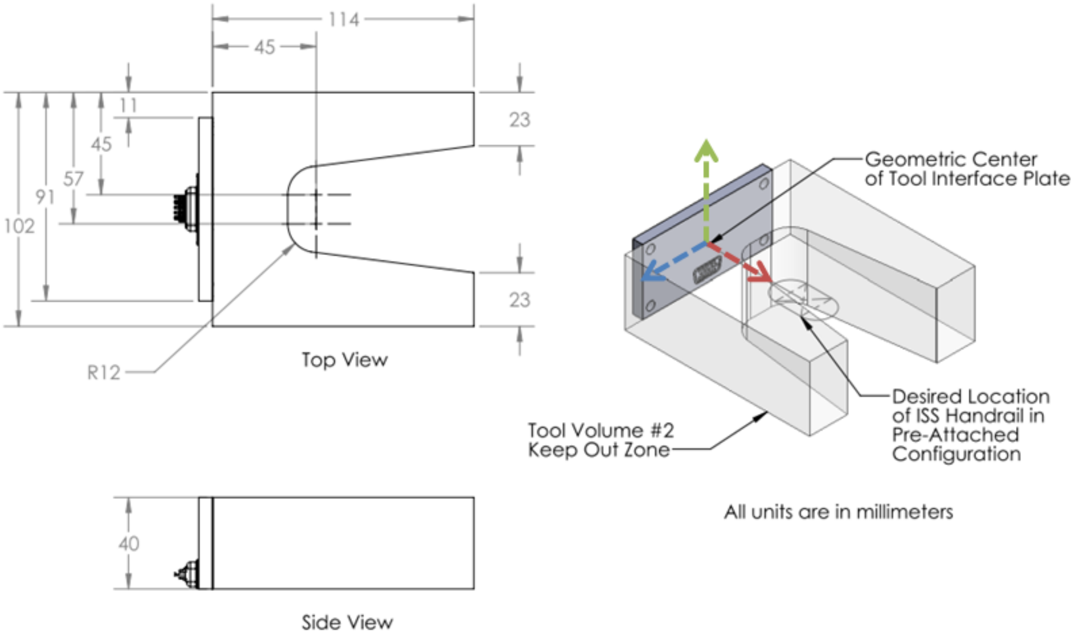

Figure 13 - Tool Volume 3, Open Volume

C12 Mounting interface: The free end of the SFA shall provide the interface pate shown in Figure 14.

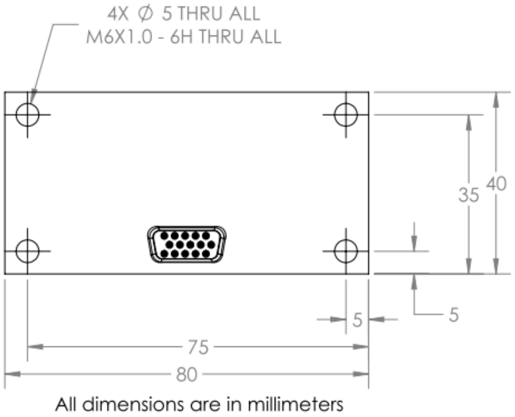

Figure 14 - Required Tool Interface Plate

C13 Mass Properties: Treat the Tool as mass of 400 grams with center of gravity offset 40 mm in x, and 0 mm in y and z from the interface plate centroid. You can assume the center of gravity is the same in all Tool Volumes

4.2.2 Power Interface

C14 The SFA shall power and control the Tool through the data connector shown in Figure 15.It is a standard D-Subminiature High Density 15 pin connector (DSUB-15). The receptacle is on the SFA side.

C15 The SFA shall provide a bus voltage+ of 14.4 volts DC. It can vary between 11 to 17 volts DC.

C16 Max current: The SFA shall accommodate a max current draw of up to 3 A peak from the Astrobee Bus

C17 Steady State Current: The SFA shall accommodate a steady state current draw of up to 2 A from the Astrobee Bus

C18 Pin out is as follows: Pin 4 is bus voltage. Pin 1 is ground.

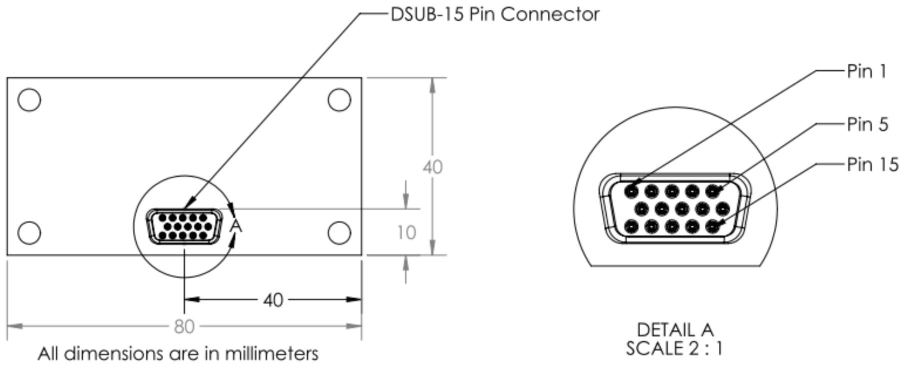

Figure 15 - Interface Plate Data and Electrical Connections

4.2.3 Data Interface

C19 All commands will be received and transmitted as part of a serial command using the second and third pins. The second pin is the positive serial command line, and the third pin is the negative serial command line.

C20 Commands (received and transmitted) shall be serial and formatted in ASCII using the RS-232 protocol once. They are specified in Table 2.

Table 2 – Command format

Command format	SFA Action
Sent from SFA to Tool	
"tool_unpack"	Enter standby and wait for response (R7)
"tool_close"	Enter standby and wait for response (R7)
"tool_release"	Enter standby and wait for response (R10)
"tool_pack"	Enter standby and wait for response (R10)
Received by SFA from Tool	
"tool_unpackconfirmed"	Initiate place (R7)
"tool_closeconfirmed"	Send an "attachconfirmed" signal to Astrobee (R7, C10) and enter standby
"tool_releaseconfirmed"	Initiate withdraw (R10)
"tool_packconfirmed"	Initiate stow (R10)

4.3 Handrail Interface

C21 The shape of a standard Handrail and associated wall attachment is defined in Figure 16. The "attach(x,y,z)" coordinate will always reference the centroid of the Handrail (in the blue region).

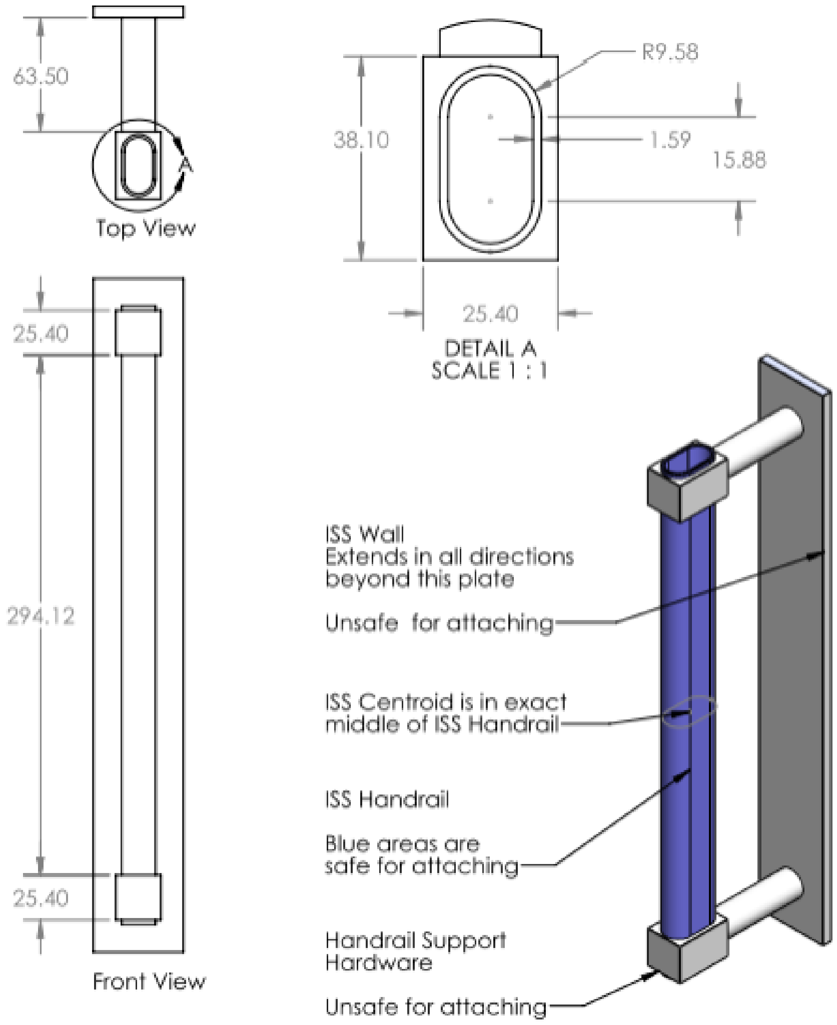

Figure 16 - ISS Handrail Definition and Safe Grasping Region
