# Supplementary material for: Linking solver characteristics, solving processes and solution attributes: A data explainer for an open innovation generated robotic design dataset
Source: Data Brief. 2023 Sep 6;50:109547. doi: 10.1016/j.dib.2023.109547 (PMC10518673; doi:10.1016/j.dib.2023.109547)
Supplement: Supplementary file 1 [file mmc1.zip › Release/Process/Challenge Rules/D3-SPAM/SPAM submission guidelines.pdf]

Submission Guidelines for the Smart Positioning and Attachment Mechanism

In this contest, you were asked to design a "smart" positioning and attachment mechanism (SPAM) that will be mounted to the free end of a separately designed robotic arm.

This document provides detailed guidelines on how you must describe and present each aspect of your design in order to be considered for the prize. This document looks long but very little text is required. Your submission document must include each of the sections detailed below and all of the information requested in each. Several templates and examples are provided to clarify what constitutes a complete solution.

Use the exact section and subsection header words, shown below.

1 FUNCTIONAL DESCRIPTION 2

1.1 NARRATIVE (WORD) DESCRIPTION OF DESIGN 2
1.2 FUNCTIONAL ANALYSIS 2

2 MASS SUMMARY AND COMPONENT LIST 3

3 SYSTEM LAYOUT 4

4 DESIGN DRAWINGS 6

5 SOFTWARE DESCRIPTION 7

6 POWER USAGE DESCRIPTION 8

7 EXIT SURVEY 9

1 Functional Description

1.1 Narrative (word) description of design

In this section, describe how your design for the Smart Positioning and Attachment Mechanism (SPAM) works. In a few sentences, please describe how your solution does each of the following:

1) Attach: How does your SPAM autonomously transition from the *Stowed* configuration to attached to a Handrail?
2) Retract: How does your SPAM autonomously return to a Stowed configuration from being attached to a Handrail?
3) Astronaut Pull Away: How does your SPAM release from the Handrail when the astronaut pull load is applied to the interface plate?

Although it is not required, you may embed images with sketches, models, storyboards or other illustrations in your written descriptions to help explain how your SPAM design accomplishes these high-level operations.

Minimum content requirement: Text reponse to each of the above questions.

1.2 Functional Analysis

In this section, describe your logic and/or analysis for the following aspects of your SPAM design. Including equations and mathematics is acceptable if it helps clarify the logic behind your design, but please ensure that it will be understood by our reviewers by annotating your process or describing the math being done and why.

(1) What is the typical force you expect the SPAM will exert on the Handrail while *attached (R3)*?
(2) When an astronaut applies a pull-away force (R27) what is the load the SPAM exerts on the Handrail?
(3) How does your SPAM design attach to a Handrail when the Handrail is at the maximum extent of its grasping region (per R1.2)?

Mimumum content requirement: Text responding to each of the above questions. Response must include numerical value.

2 Mass Summary and Component List

In this section, list all the elements of your SPAM solution using the template provided [SPAMMassTemplate, available as google doc, .odt, .xlsx]. For each component/piece/part, include an estimate of its mass and a brief explanation of where the estimate came from. Please be sure to include the reasons supporting your mass estimate for each element since they will be part of the evaluation of the credibility of your SPAM mass estimate.

Table 1 provides an example of how the template should be filled in.

		Is this a powered component?	Estimated Mass per Unit (kg)	Quantity (# units)	Mass (kg)	Basis of Estimate
**1.0**	**Subsystem #1**				**1.88**	
	Electronics Box A	Yes	0.200	1	0.20	Weighed a prototype I built
	Switch #1 & 2	Yes	0.030	2	0.06	Called some former coworker who builds these, and asked for a typical masses
	Mechanism #1	No	0.800	1	0.80	Made a CAD model, assumed SS316, to obtain this mass
	Attachment hardware	No	0.040	8	0.32	Typical mass of component that I use all the time in design of systemX.

Table 1 - Mass Summary and Components List example

Minimum content requirement: Paste your filled table into this section of the document. No additional text is required.

3 System Layout

In this section, provide a diagram(s) identifying all the physical components/pieces/parts of your SPAM design and how components connect to, move and/or, power each other.

Please use the names of components from the Mass Summary and Component List described in section 1. You may represent the electrical and mechanical aspects of your design together or separately.

If you represent mechanical and electrical together, the system layout should be presented as a block diagram. A Block Diagram shows how each of the components connect to one another. Each component is represented as a box and the lines that connect the blocks identify what is being transferred/passed or supported between the blocks. Be sure to identify how your design connects to the robotic arm interface. Figure 1 provides an example of a block diagram.

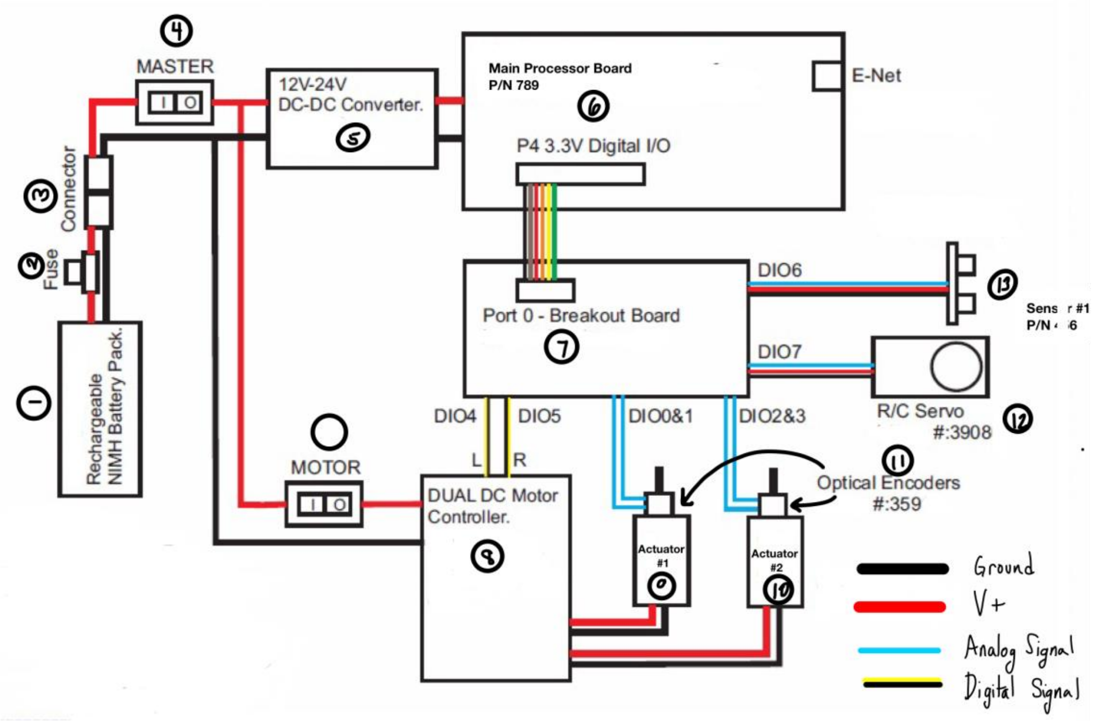

Figure 1 - Block Diagram - example

If you represent mechanical and electrical separately, you should use the following formats:

For mechanical, use a schematic similar to an "exploded view" of your design. See Figure 2 for an example. The defining feature of an exploded view is that it conveys information about how all the mechanical pieces connect to one another.

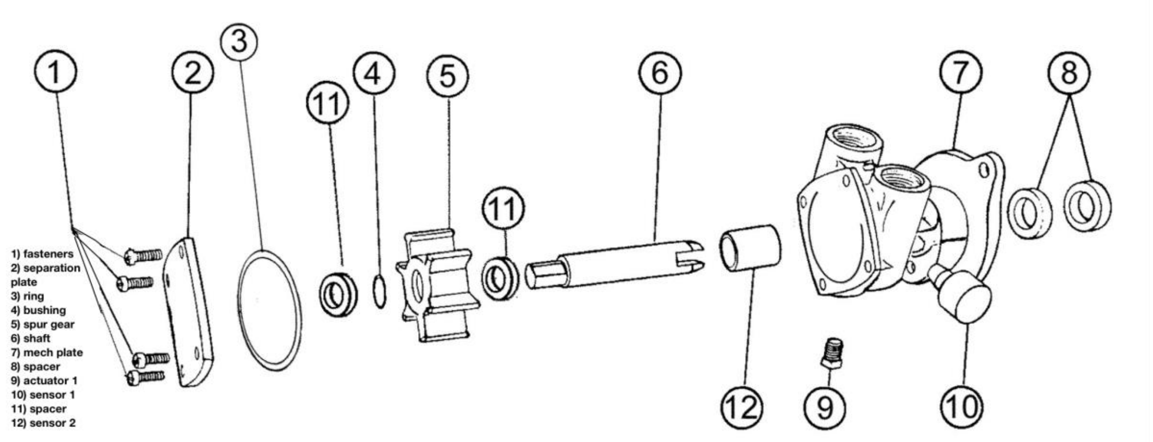

Figure 2 - Mechanical (exploded view) schematic - example

For electrical, use a schematic similar to a "Wiring Diagram" See Figure 3 for an example. The schematic must identify all the direct[1] electrical connections among all the electrical, electro-mechanical (such as actuators, motors, solenoids, etc.), and electro-computational (driver circuits, signal wires etc.) components of your solution. Make sure to identify any connection between your electrical system and the connector in the interface plate.

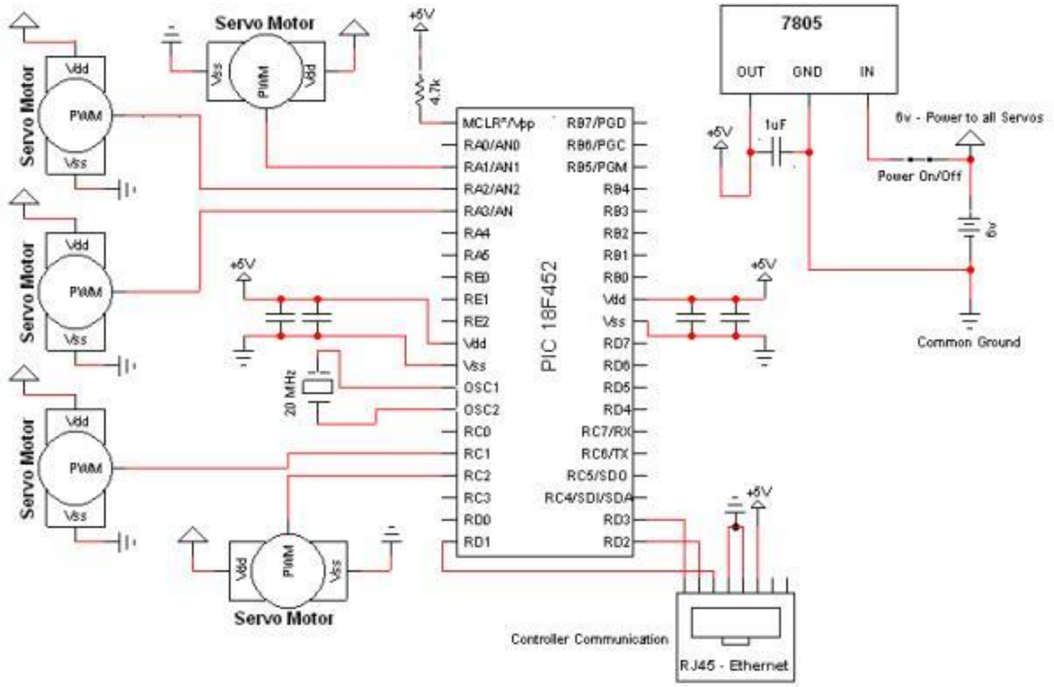

Figure 3 - Wiring Diagram - example

[1] Only detail the direct electrical connections. For example, if you have a gearbox connected to an electrical motor which has electrical leads coming off of it, this diagram would only show the electric motor and the electrical leads as well as those lead's connection to the larger system. The diagram would not need to show the gearbox, as it is separated from the electrical system by the electric motor.

4 Design Drawings

In this section, include engineering-style drawings showing your solution's physical configuration for each of the following.

1) *Stowed configuration (R1.1)*
2) *Attached to Handrail that is at the location, (160mm, 50mm, -95mm)*

You can use any CAD software you like, sketch them by hand or photograph a prototype, but accurate dimensions of the whole system are required (we'd much rather have proof that your deign can fit in its stowage volume than the specific location of each bolt). For each view, please provide at least one off-angle view to show perspective. Please label as many elements and subassemblies as possible. Use the names specified in your Mass Summary and Component List (section 1).

Minimum content requirement: Three design drawing figures – one for each configuration listed above. Figures must be clearly labeled and dimensioned.

5 Software Description

In this section, describe at a high-level how your SPAM design autonomously accomplishes the following reference sequence of operations. You do not need to specify exact code or strategies involved in your software, just generally what function might be used and how. Be sure to include how each element or subassembly identified in the Component List is being controlled, and how your software uses any feedback from (e.g., sensors). Your software description should be complete enough that an experienced programmer could implement the algorithm.

Reference sequence:

1) Commanded to <u>Attach</u>
2) Commanded to <u>Retract</u> after 60 minutes

Present your software's control flow description using an Activity/Control Flow Diagram, also known as a Flowchart. An Activity Flow Diagram use squares to represent processes, diamonds to represent true/false case structures, ovals to indicate start and stops of programs, and arrows to designate program flow and sequence. Your Activity Flow Diagram must show every outcome and what steps are taken to get there. See Figure 4 for an example.

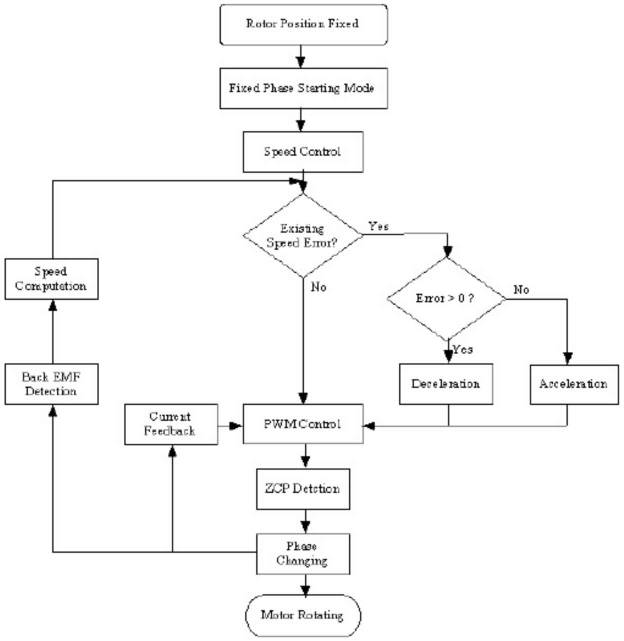

Figure 4 - Software Control Flow Diagram - example

Minimum content requirement: A flowchart that shows how your software would address the reference sequence. No text is required. Standard symbols must be used.

6 Power Usage Description

In this section, describe how your solution stays within the electrical power constraints (R15, C4, C5) by populating the attached Power Profile template [filename: SPAMPowerProfileTemplate.xlsx (.ods) or google sheet]For each component/part that uses power, you must estimate its usage (time and power level) during each expected operation. The operational sequence for power usage is:

1) Attach to a Handrail at (160, 50, -95)
2) Remain attached for 60 minutes
3) Retract to the *Stowed* configuration

Table 2 shows an example of a filled in power profile.

Step 1: Description of power modes

	Power Mode Description
**Mode 1**	Reading all sensors,
**Mode 2**	Running Actuators #1, Sensor #2, and Switches #1, #4-#6
**Mode 3**	Power draw while waiting (and attached to Handrail) for next operation

Step 2: List of Powered Elements

		Mode 1	***Mode 2***	***Mode 3***	***Mode 4***	***Mode 5***
		Current (A)	Current (A)	Current (A)	Current (A)	Current (A)
**1.0**	**Element #1**					
	Electronics Box A	0.1	0.1	0.1	0.1	0.1
	Switch #1		0.2			
	Switch #2					

Step 3: Power Profile for Sequence of Operations

		Which Power Mode	Time On (s)	Energy (W)
(1) Attach to Handrail				
	Attach command received			
	Action #1 - (operations in low power mode)	Mode 1	30	0.08
	Drive Element #2	Mode 4	90	0.49

Table 2 - Power Profile Example

Please continue to use the same names of components used in your Component List.

Minimum content requirement: Paste a figure of your filled in tables for the Power Profile into this document. No additional text is required.

7 Exit Survey

To complete your submission, please take the Exit Survey by going to this webpage: https://seasgwu.qualtrics.com/jfe/form/SV_2r9DaeSlh48uMcZ

At the end of the survey you will receive a unique code. In your submission include this section and the text: Exit Survey for Freelancer <<insert Freelancer username>> complete per completion code: <<insert completion code>>.

To be complete, your submission must include the following text: Exit Survey for Freelancer <<insert Freelancer username>> complete per completion code: <<insert completion code>>.
