# Supplementary material for: Linking solver characteristics, solving processes and solution attributes: A data explainer for an open innovation generated robotic design dataset
Source: Data Brief. 2023 Sep 6;50:109547. doi: 10.1016/j.dib.2023.109547 (PMC10518673; doi:10.1016/j.dib.2023.109547)
Supplement: Supplementary file 1 [file mmc1.zip › Release/Process/Challenge Rules/D3-SPAM/SPAM Problem Description.pdf]

1 Contest Description

In this challenge, you are asked to design a "smart" positioning and attachment mechanism (SPAM) that will be mounted to the free end of a separately-designed robotic arm. The SPAM receives all power and high-level commands through its interface to the robotic arm, but implements the following functions autonomously: stowing and deploying from a defined volume, moving to and attaching to an International Space Station (ISS) Handrail ("Handrail"), holding on and then retracting. The below specification details how the SPAM will work, it's functional requirements and interface constraints/assumptions. A separate document provides detailed guidelines on how your design must be presented and submitted.

A prize of $4000 will be awarded for the lowest mass, technically feasible solution, submitted before 13:00 GMT on July 12th 2018.

2 Concept of Operations – How the SPAM needs to work

2.1 Normal Operations

The SPAM must be able to autonomously perform two operations: 1) attach, which involves unpacking from it's *stowed* configuration, moving to a precise Handrail location and attaching securely to it for an extended period of time; and 2) retract, which involves releasing the Handrail and packing itself back in it's initial *stowed* configuration. For reference, Figure 1 shows the stowed configuration volume relative to the Astrobee keep-out zone and Handrail grasping range.

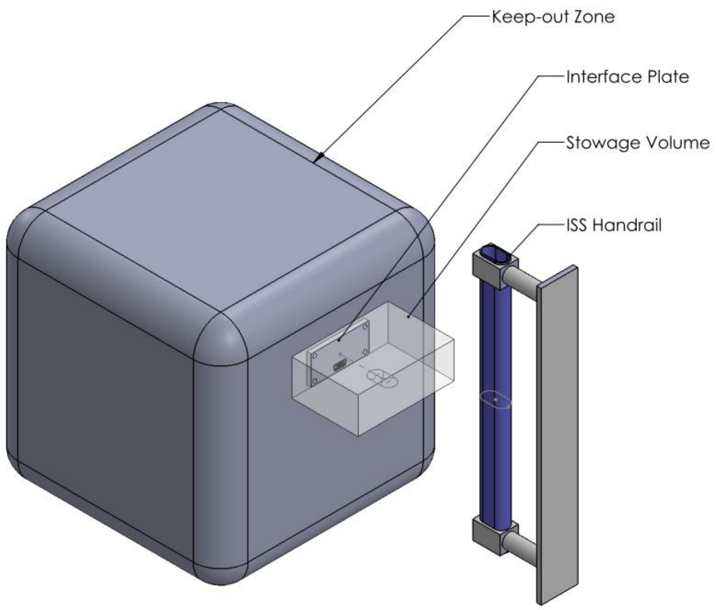

Figure 1- Concept of Operations

2.2 Contingency (Emergency) Operations

There are several scenarios when normal operations may be disrupted. The ranges of permissible responses are detailed in section R9. This section summarizes the scenarios: 1) when the SPAM is commanded to attach, but there is no handrail present; 2) when the SPAM experiences higher than expected loads while attached (e.g., because an astronaut or other object makes contact with Astrobee); and 3) when an astronaut manually removes the SPAM from the handrail.

3 Functional Requirements

This section details all of the functional requirements that the SPAM must meet.

3.1 Motion requirements

R1 Attach: The SPAM shall be able to move from the *stowed* configuration and attach to a specified Handrail located anywhere in the grasping region defined below, without contacting Astrobee or the ISS walls. You may assume that the robotic arm interface is fixed in space during attaching.

R1.1 Stowed configuration: the stowed configuration volume is defined in Figure 2 as 92 mm x 50 mm x 134 mm [3.6” x 2.0” x 5.3”]

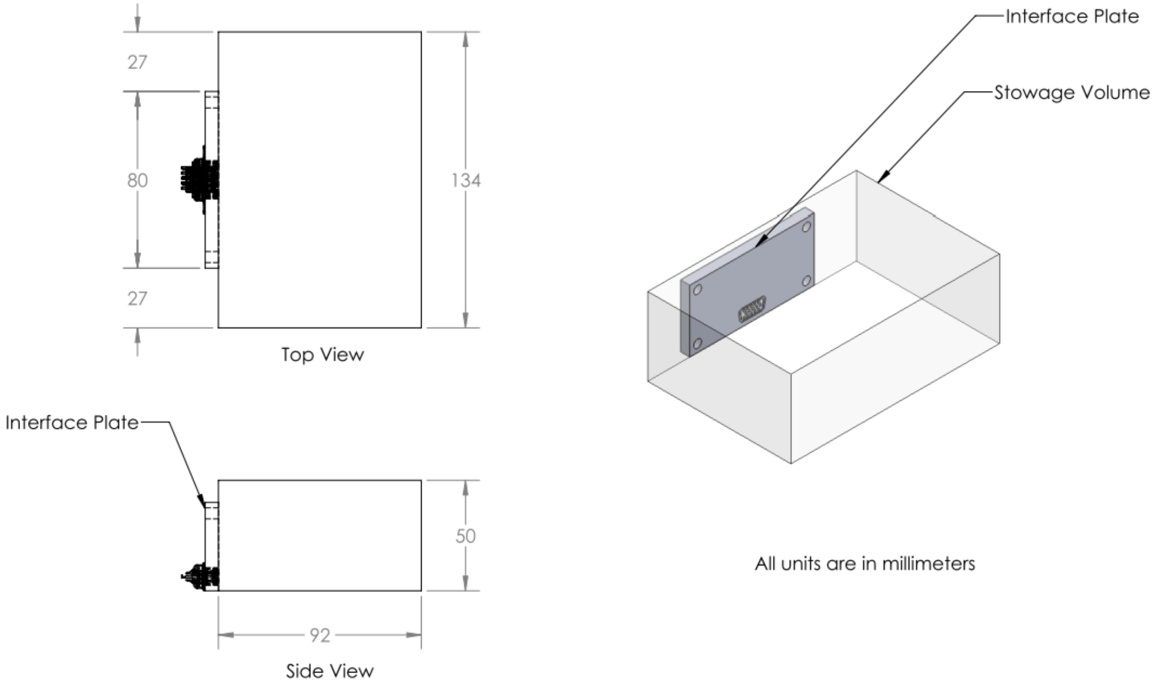

Figure 2 - Stowed Configuration

R1.2 The SPAM shall be able to attach to a Handrail located anywhere within the grasping region defined by the below volume:

1. 132 mm ≤ x ≤ 182 mm [5.2” ≤ x ≤ 7.2”]
2. -45 mm ≤ y ≤ 105 mm[-1.8” ≤ y ≤ 4.1”]
3. -100 mm ≤ z ≤ 100 mm[-3.9” ≤ z ≤ 3.9”]

Figure 3 the reference coordinate system. The origin lies at the center of the SPAM interface plate. The Cartesian coordinates (x, y, z) indicate the position of the centroid of the Handrail that the SPAM must attach too. The Handrail will never be rotated with respect to the orientation of the Interface Plate.

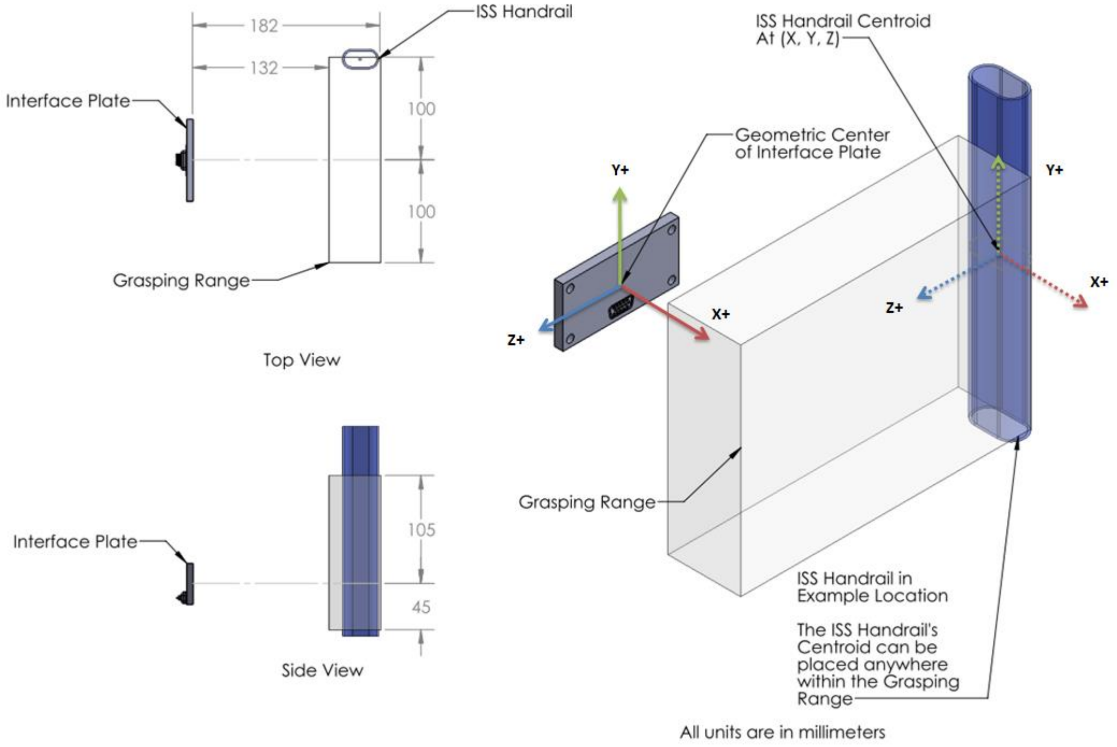

Figure 3 - Interface Plate and Workspace Definition

R1.3 The SPAM shall only ever be commanded to <u>attach</u> to a standard ISS Handrail as defined in interface constraint (C10).

R1.4 The SPAM shall not contact the rest of the system (see keep-out volume, C2) or the ISS wall during <u>attach</u> operations (see Handrail mounting, C10).

R1.5 The SPAM shall be considered *attached* when it if fixed to the Handrail. Fixed is defined as being able to resist slipping or twisting when subjected to normal operating loads of up to 3.5 Nm [2.6 ft-lbf] about either the Y-axis (M_y) or Z-axis (M_z) as seen in Figure 4.

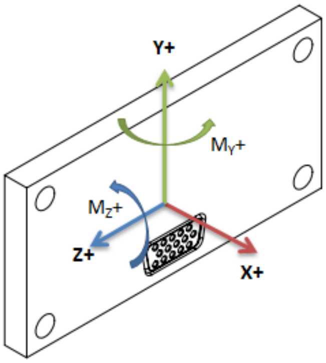

Figure 4 – SPAM-Robotic Arm Interface Plate and Moments

R2 Retract: The SPAM shall be able to detach from the Handrail and maneuver to return to its *stowed* configuration (R1.1).

3.2 Control Requirements

The format of all commands is specified in section 4.1.2 (C8 and C9). This section describes how the SPAM shall be controlled.

R3 Attach

- R3.1 When commanded to "attach(x,y,z)", the SPAM shall autonomously attach (R1).
- R3.2 When attaching is complete (R1.5), the SPAM shall send an "attachconfirmed" signal to the robotic arm and maintain a rigid hold on the Handrail until it receives further instructions.

R4 Retract

- R4.1 When commanded to "retract", the SPAM shall autonomously stow in its stowed configuration (R1.1).
- R4.2 When stowing is complete, the SPAM shall send a "retractconfirmed" signal to the robotic arm and await further commands.

3.3 Resource Requirements

3.3.1 Timing Requirements

R5 Time to Attach: Attach operations shall not exceed 7.5 minutes. The timing is measured based on the initiation and confirmation command structure specified in R3.

R6 Time Attached: After completing the Attach operation, SPAM shall be able to remain securely *attached* to the Handrail for up to 1 hr. *Attached* begins when the "attachconfirmed" command is sent and ends when the "retract" command is received.

R7 Time to Retract: Retract operations shall not exceed 7.5 minutes. The timing is measured based on the initiation and confirmation command structure specified in R4.

3.3.2 Power Requirements

All power is transmitted through the connector described in interface constraint C2.3.

R8 Energy: The SPAM shall not use more than 18 Watt-hours for all operations. Assume 1hr will be spent *attached*. The time to attach and retract is a determined by your design.

3.4 Safety Requirements

R9 The SPAM shall have no sharp edges, defined as a radius of 3 mm [.11"], for astronaut safety.

R10 The SPAM shall have no loops of material greater than 25.4 mm [1"] in diameter for astronaut safety.

R11 The SPAM shall not damage itself through normal operations.

R12 The SPAM shall be able to return to its normal operations if power is momentarily lost.

3.5 Environmental Requirements

R13 The SPAM shall operate in the ISS zero gravity environment.

R14 The SPAM, when unpowered, shall not be damaged by electrostatic discharge <4,000V.

R15 The SPAM shall operate in an atmosphere comparable to that of Earth. Assume 21 degrees centigrade [70 degrees Fahrenheit], with low humidity, and pressurized to 100 kPA [750 mm Hg, 14.5 psi].

R16 The SPAM shall not contribute any particulates (e.g. dust) to the ISS atmosphere.

R17 The SPAM shall enclose all lubricated components to prevent lubricants from leaking into the atmosphere of the ISS.

3.6 Contingency Requirements

R18 No Handrail: In some cases, a command to “attach(x,y,z)” may be sent to the SPAM, but there is no Handrail at the specified location. The SPAM need not recognize this type of error, but shall also not damage itself while trying to complete the operation.

R19 Excessive loads cases. This scenario may occur if an astronaut or piece of equipment contacts Astrobee while the SPAM is *attached* (including while experiencing normal operating loads per R1.5). The SPAM shall break away from the Handrail if it experiences a force of greater than 18N [4 lbf] applied at the SPAM interface in the negative Y-direction and a simultaneous moment of 2.7Nm [2 ft-lbs] about the Z-axis (see Figure 5a).

R20 Astronaut intervention: The SPAM shall be removable from the Handrail by an astronaut. Assume an astronaut can apply a pull-away force of 35.6 N [8 lbf] in the negative X-direction (see Figure 5b). This will not occur during any other operation.

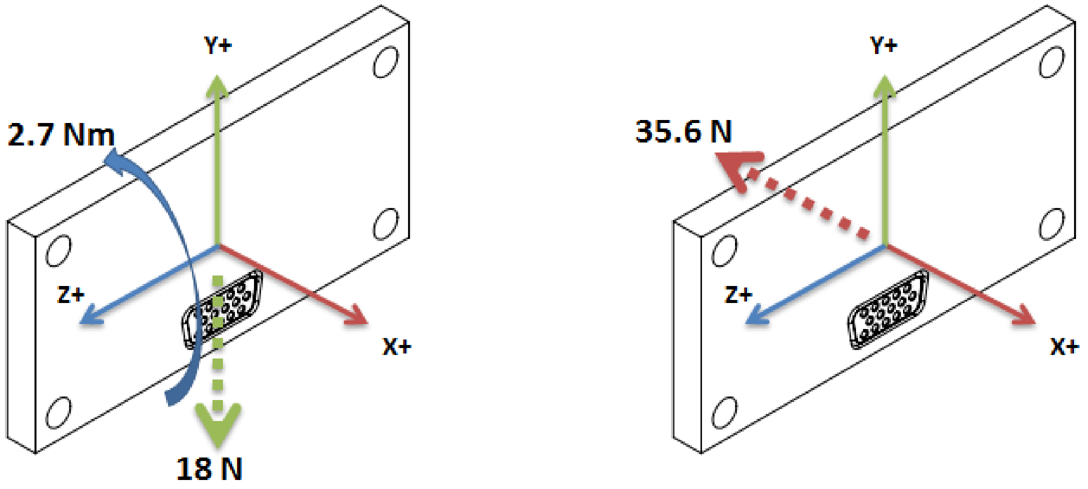

Figure 5 - Contingency loads: a) shows Excessive loads; b) shows Astronaut intervention loads

4 Interface Requirements

The SPAM has a fixed interface to a separately designed robotic arm and a dynamic interaction with ISS Handrails. The section describes all constraints imposed by those interfaces.

4.1 SPAM-Robotic Arm Interface

C1 Constraint (C1) Mounting Interface: The SPAM shall mechanically mount to a flat metal plate show in Figure 6. There are four available screw holes in the specified locations.

C1.1 All external loads are applied at the interface plate.

C1.2 Screws for your selected electrical connectors must only require hand tightening of locking screws. Assume that no external loads are applied through the electrical connector.

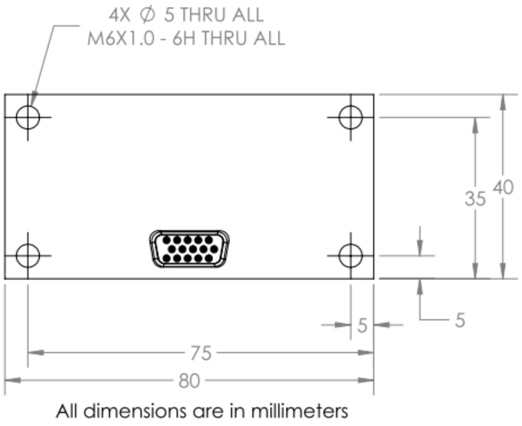

Figure 6 - SPAM-Robotic Arm Mounting Interface

C2 Keep-out zone: Figure 7 defines a keep-out zone that abstracts the physical space occupied by the remainder of the system (i.e., Astrobee and robotic arm). Use this volume in assessing collision avoidance (to support attach and retract operations).

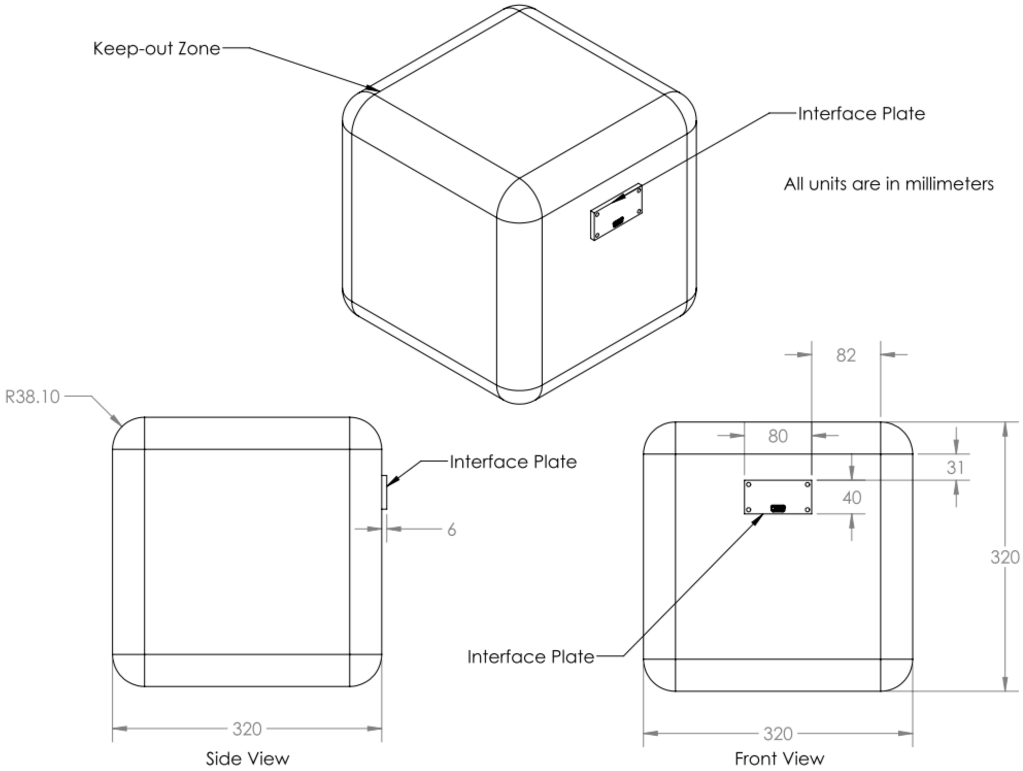

Figure 7 - Keep-out zone

4.1.1 Power Interface:

C3 The SPAM shall connect to the power and data connector shown in Figure 8. It is a standard D-Subminiature High Density 15pin connector (DSUB-15).

C4 Astrobee Bus Voltage+ is nominally 14.4 volts DC, but can vary between 11 to 17 volts DC.

C5 Max current: The SPAM shall not draw more than 3 Amps.

C6 Steady State Current: The SPAM shall not draw more than 2 Amps at steady state.

C7 Pin out is as follows: Pin 4 is bus voltage. Pin 1 is ground.

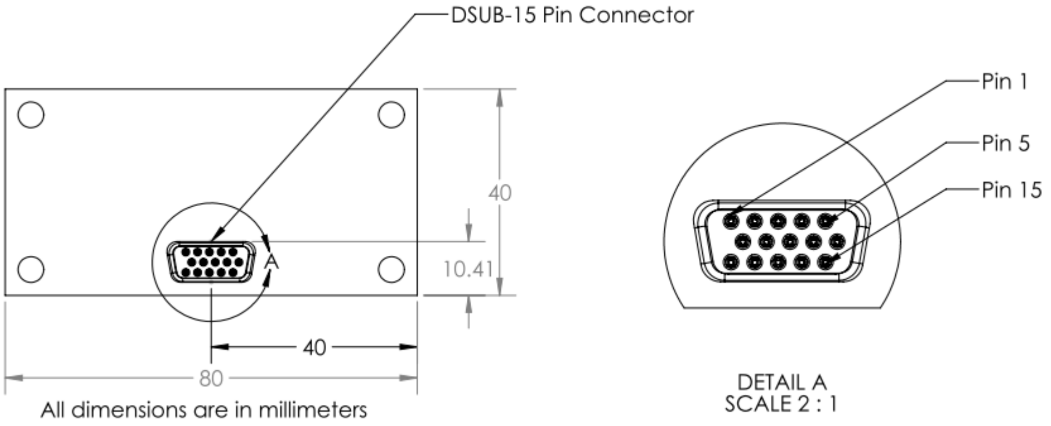

Figure 8 - Power/data connector location

4.1.2 Data Interface:

C8 All commands will be received and transmitted as part of a serial command using the second and third pins. The second pin is the positive serial command line, and the third pin is the negative serial command line.

C9 Commands (received and transmitted) shall be serial and formatted in ASCII using the RS-232 protocol once. They are specified in Table 1.

Table 1 – Command and Confirmation formats

Command	Action
**(1) Sent from robotic arm to SPAM**	
"attach(x, y, z)"	Initiate attach to Handrail at the specific location, (x,y,z) (R3.1)
"retract"	Initiate retract operation (R4.1)
**(2) Sent from SPAM to robotic arm**	
"attachConfirmed"	Confirm attach has completed (R3.2)
"retractConfirmed"	Confirm retract has completed (R4.2)

4.2 Handrail Interface

C10 Handrail definition:

C10.1 The shape of a standard Handrail is defined in Figure 9. The SPAM shall only attach to the Handrail in the blue region shown in the figure.

C10.2 The ISS Handrail is made of anodized aluminum. Assume the material is 6061 Aluminum of type T4 in terms of material properties and friction properties.

C10.3 The ISS Handrail is 1.59mm [1/16”] thick aluminum 6061 extrusion.

C11 The Handrail shall not be damaged during operations through excessive force (see R1.5, R19 and R20). Damage includes, but is not limited to: crushing, denting, or bending.

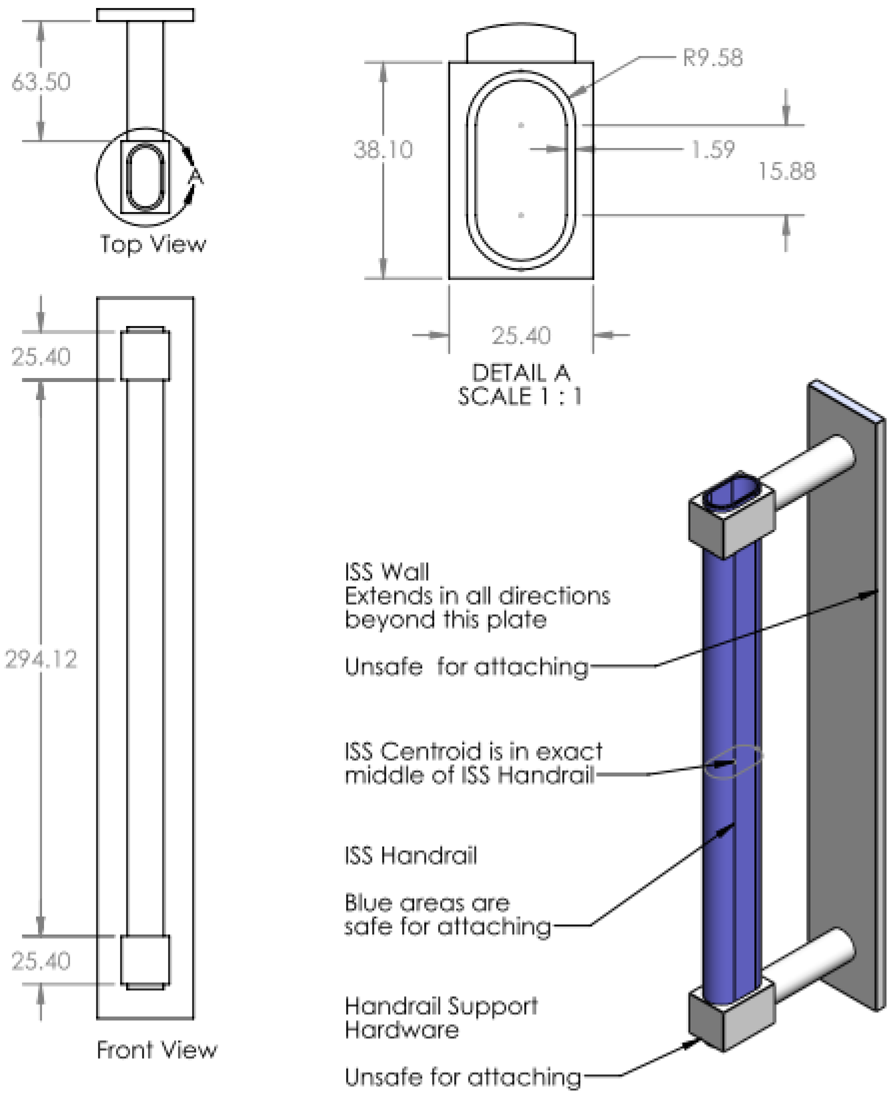

Figure 9- ISS Handrail Definition and Safe Grasping Region
