# Supplementary material for: Linking solver characteristics, solving processes and solution attributes: A data explainer for an open innovation generated robotic design dataset
Source: Data Brief. 2023 Sep 6;50:109547. doi: 10.1016/j.dib.2023.109547 (PMC10518673; doi:10.1016/j.dib.2023.109547)
Supplement: Supplementary file 1 [file mmc1.zip › Release/Process/Challenge Rules/D3-SPAM/SPAM Blurb.docx]

“Smart” Positioning and Attachment Mechanism (SPAM)

In this challenge, you are asked to design a “smart” positioning and attachment mechanism (SPAM) that will be mounted to the free end of a separately designed robotic arm. The SPAM receives all power and high-level commands through its interface to the robotic arm, but implements the below functions autonomously.

How it works: Initially, the SPAM will be packed in its stowed configuration. Once activated, the SPAM must be capable of autonomously performing three high-level actions: 1) unpack from its stowed configuration, move the remaining distance to a specified Handrail location, and then attach itself to the Handrail, 2) maintain its hold on the handrail for an extended period of time, 3) retract itself from the handrail and return to the initial stowed configuration.

*Click on the links below to see detailed design instructions, constraints and solution templates for this problem.*

Challenge rules: A prize of **$4,000** will be awarded for the **lowest mass, technically feasible** solution submitted by **July 12^th^ 2018**. No working prototype is required for submission, but the design must be sufficiently detailed to allow experts to assess the feasibility of your design (i.e., comply with all requirements) and the credibility of your mass estimate. Only complete submission packages will be evaluated.

Attachments:

SPAMProblemDescription.pdf

SPAMSubmissionGuidelines.pdf

Templates

- SPAMMassTemplate [.xlsx, odt, [google docs](https://docs.google.com/spreadsheets/d/1FhVIPpblxaseZQUPF0qSJNllVWs64r_-E_VfxB8EpAs/edit?usp=sharing)]
- SPAMPowerProfileTemplate [.xlsx, .odt, [google docs](https://docs.google.com/spreadsheets/d/1FhVIPpblxaseZQUPF0qSJNllVWs64r_-E_VfxB8EpAs/edit?usp=sharing)]

Steve, if you need the full link text. Here’s mass: <https://docs.google.com/spreadsheets/d/1FhVIPpblxaseZQUPF0qSJNllVWs64r_-E_VfxB8EpAs/edit?usp=sharing>

And power: <https://docs.google.com/spreadsheets/d/1bK3mXEFK9UZAS0PiWpN4JUX_-fy9k-uOhV5KCSRyveM/edit?usp=sharing>

Actual Text (pasted from Freelancer Contest Description Field):

Design a “Smart” Positioning and Attachment Mechanism (SPAM)

In this challenge, you are asked to design a “smart” positioning and attachment mechanism (SPAM) that will be mounted to the free end of a separately designed robotic arm. The SPAM receives all power and high-level commands through its interface to the robotic arm, but implements the below functions autonomously.

How it works: Initially, the SPAM will be packed in its stowed configuration. Once activated, the SPAM must be capable of autonomously performing three high-level actions: 
1) unpack from its stowed configuration, move the remaining distance to a specified Handrail location, and then attach itself to the Handrail, 
2) maintain its hold on the handrail for an extended period of time, 
3) retract itself from the handrail and return to the initial stowed configuration. 

Click on the links below to see detailed design instructions, constraints and solution templates for this problem. 
- The SPAMMassTemplate may be submitted in any of the 3 formats provided: XLSX, ODT, or Google Sheets (<https://docs.google.com/spreadsheets/d/1FhVIPpblxaseZQUPF0qSJNllVWs64r_-E_VfxB8EpAs/edit?usp=sharing>)
- The SPAMPowerProfileTemplate may be submitted in any of the 3 formats provided: XLSX, ODT, or Google Sheets (<https://docs.google.com/spreadsheets/d/1bK3mXEFK9UZAS0PiWpN4JUX_-fy9k-uOhV5KCSRyveM/edit?usp=sharing>)

Challenge rules: 
- The prize will be awarded for the lowest mass, technically feasible solution, submitted by the contest deadline. 
- No working prototype is required for submission, but the design must be sufficiently detailed to allow experts to assess the feasibility of your design (i.e., comply with all requirements) and the credibility of your mass estimate. 
- Only complete submission packages will be evaluated (see attachments).

NASA may select multiple winners or provide additional prize compensation on entries that are particularly novel or innovative.

NASA will be available to respond to clarifying questions, but feedback on quality is otherwise limited. 
All complete submissions will be confirmed with 3-star ratings. 
Note that final judgement of quality and winners will only happen after the submission deadline.
