# Supplementary material for: Linking solver characteristics, solving processes and solution attributes: A data explainer for an open innovation generated robotic design dataset
Source: Data Brief. 2023 Sep 6;50:109547. doi: 10.1016/j.dib.2023.109547 (PMC10518673; doi:10.1016/j.dib.2023.109547)
Supplement: Supplementary file 1 [file mmc1.zip › Release/Process/Challenge Rules/D5-MIS/MISSubmissionGuidelines.pdf]

Submission Guidelines for the Material Interface Surface (MIS)

In this contest, you were asked to design or select a material with properties that provide a non-slip interface between a Robotic Arm and a Handrail to which it is secured.

This document provides detailed guidelines on how you must describe and present each aspect of your design in order to be considered for the prize. Your submission document must include each of the sections detailed below and all of the information requested in each. Examples are provided where possible to clarify what constitutes a complete solution.

Use the exact section and subsection header words, shown below.

Submission Guidelines for the Material Interface Surface (MIS) 1

1 Design Description 1

1.1 Narrative (word) description of Material Interface Surface 1

1.2 Functional Analysis 1

2 Exit Survey 2

1 Design Description

1.1 Narrative (word) description of Material Interface Surface

Describe in words how your Material Interface Surface design meets the requirements of the is contest. What are its unique characteristics and features?

Minimum content requirement: Description of your material design or selection.

1.2 Functional Analysis

1) What is the estimated coefficient of friction between your MIS and the Handrail surface?
2) What is the minimum force that needs to be applied to the MIS to prevent slipping under normal operations? Please include detailed rationale for your estimate of minimum applied force. The credibility of your applied force estimate is based largely on your description here of how you arrived at the number.
3) Can your MIS design act as an electrical conductor, either with or without an applied normal force? If so, what is its estimated resistivity?

Minimum content requirement: Text responding to each of the above questions.

2 Exit Survey

To complete your submission, please take the Exit Survey by going to this webpage:

https://seasgwu.qualtrics.com/jfe/form/SV_2r9DaeSlh48uMcZ

At the end of the survey you will receive a unique code. In your submission, include this section and the text:

Exit Survey for Freelancer <<insert Freelancer username>> complete per completion code: <<insert completion code>>.
