# Supplementary material for: Linking solver characteristics, solving processes and solution attributes: A data explainer for an open innovation generated robotic design dataset
Source: Data Brief. 2023 Sep 6;50:109547. doi: 10.1016/j.dib.2023.109547 (PMC10518673; doi:10.1016/j.dib.2023.109547)
Supplement: Supplementary file 1 [file mmc1.zip › Release/Process/Challenge Rules/D5-MIS/MISProblemDescription.pdf]

1 Contest Description

In this challenge, you are asked to design the Material Interface Surface (MIS) for the contact between a Robotic Arm (RA) and Handrail for use on the International Space Station. The MIS will be attached to the free end of the Robotic Arm, but no consideration of how that attachment will occur is required for this challenge. When a force is applied to MIS, it must prevent slipping between the robotic arm and the Handrail during normal operations.

Challenge rules: A prize will be awarded for the **technically feasible** solution that requires the **lowest force to prevent slipping**. No working prototype is required for submission, but the design must be sufficiently detailed to allow experts to assess the feasibility of your design (i.e., comply with all requirements) and the credibility of your force estimate. Only complete submission packages will be evaluated.

2 Normal Operations - How the MIS needs to work

Your interface material needs to control or modify the interface with the Handrail surface such that when a uniformly distributed force ($F_{applied}$) is applied to the MIS, it can resist a "Pull" force. (Figure 1).

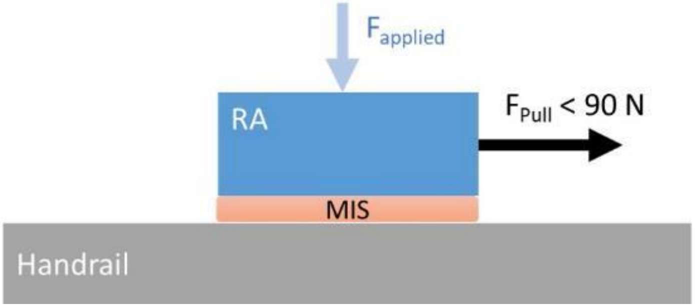

Figure 1. RA fixed to Handrail - no slip condition

When the applied force is removed, your interface material should allow the RA move freely across the surface of the Handrail.

3 Functional Requirements

This section details the functional requirements that the MIS must meet.

R1 <u>Applied force</u>: Force is applied perpendicular to the interface material, through the RA plate (represented in blue in Figure 1). This force is distributed evenly across the entire area of RA plate, and pushes the MIS interface material into the Handrail. The Handrail can be considered to be much larger than the RA plate to which the MIS is attached.

R2 Pull force: This is the maximum lateral force on the RA plate that, when applied, the two plates remain attached. F_{Pull}=90 N

4 Interfaces

The MIS has physically interfaces to the robotic arm by mechanical attachment that is designed separately from this contest.

C1 Material placement: Your interface material will be attached to a "plate" on the Robotic Arm, that is 1.5 cm x 1.5 cm in cross-section. This is shown in side view in Figure 1, above. Note that you are not responsible for defining how your material is secured to the RA in this contest.

C2 Handrail material: Handrail is made out of anodized aluminum (6061-T4).
